# Mechanistic differences between linear *vs.* spirocyclic dialkyldiazirine probes for photoaffinity labeling†

**DOI:** 10.1039/d4sc04238g

**Published:** 2024-08-13

**Authors:** Jessica G. K. O'Brien, Louis P. Conway, Paramesh K. Ramaraj, Appaso M. Jadhav, Jun Jin, Jason K. Dutra, Parrish Evers, Shadi S. Masoud, Manuel Schupp, Iakovos Saridakis, Yong Chen, Nuno Maulide, John P. Pezacki, Christopher W. am Ende, Christopher G. Parker, Joseph M. Fox

**Affiliations:** a Department of Chemistry, The Scripps Research Institute La Jolla California 92037 USA cparker@scripps.edu; b Pfizer Worldwide Research and Development Eastern Point Road, Groton Connecticut 06340 USA christopher.amende@pfizer.com; c Department of Chemistry and Biochemistry, University of Delaware Newark Delaware 19716 USA jmfox@udel.edu; d BioDuro-Sundia No.233 North FuTe Rd., WaiGaoQiao Free Trade Zone Shanghai 200131 P.R. China; e Department of Chemistry and Biomolecular Sciences, University of Ottawa Ottawa Ontario K1N 6N5 Canada; f Institute of Organic Chemistry, University of Vienna 1090 Vienna Austria

## Abstract

Dialkyldiazirines have emerged as a photo-reactive group of choice for interactome mapping in live cell experiments. Upon irradiation, ‘linear’ dialkyldiazirines produce dialkylcarbenes which are susceptible to both intramolecular reactions and unimolecular elimination processes, as well as diazoalkanes, which also participate in intermolecular labeling. Cyclobutylidene has a nonclassical bonding structure and is stable enough to be captured in bimolecular reactions. Cyclobutanediazirines have more recently been studied as photoaffinity probes based on cyclobutylidene, but the mechanism, especially with respect to the role of putative diazo intermediates, was not fully understood. Here, we show that photolysis (365 nm) of cyclobutanediazirines can produce cyclobutylidene intermediates as evidenced by formation of their expected bimolecular and unimolecular products, including methylenecyclopropane derivatives. Unlike linear diazirines, cyclobutanediazirine photolysis in the presence of tetramethylethylene produces a [2 + 1] cycloaddition adduct. By contrast, linear diazirines produce diazo compounds upon low temperature photolysis in THF, whereas diazo compounds are not detected in similar photolyses of cyclobutanediazirines. Diazocyclobutane, prepared by independent synthesis, is labile, reactive toward water and capable of protein alkylation. The rate of diazocyclobutane decomposition is not affected by 365 nm light, suggesting that the photochemical conversion of diazocyclobutane to cyclobutylidene is not an important pathway. Finally, chemical proteomic studies revealed that a likely consequence of this primary conversion to a highly reactive carbene is a marked decrease in labeling by cyclobutanediazirine-based probes relative to linear diazirine counterparts both at the individual protein and proteome-wide levels. Collectively, these observations are consistent with a mechanistic picture for cyclobutanediazirine photolysis that involves carbene chemistry with minimal formation of diazo intermediates, and contrasts with the photolyses of linear diazirines where alkylation by diazo intermediates plays a more significant role.

## Abstract 2

Linear diazirines can produce relatively long-lived diazoalkanes which are also capable of labeling proteins whereas cyclobutanediazirines are proposed to give minimal diazo product and labeling occurs predominantly *via* a carbene mechanism.

## Graphical abstract

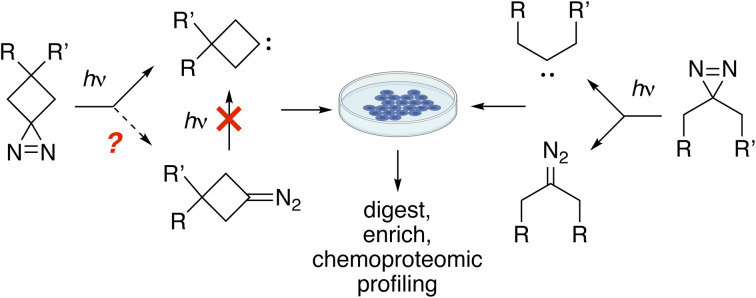

## Introduction

Photoaffinity labeling is a broadly used technique used to interrogate interactions between small molecule ligands and their biological targets in living cells.^1–4^ Photoaffinity probes are constructed by attaching a ligand to an enrichment handle (*e.g.* an alkyne or biotin) as well as a photoreactive group, which upon excitation generates a short-lived reactive intermediate capable of capturing proximal targets. Classically, aromatic diazirines, arylazides, and benzophenone analogs have employed for the construction of photoaffinity probes.^3,5^ More recent probes based on aryl carboxytetrazoles,^6^ pyrones,^7^ pyrimidones,^7^ and photoredox catalysis^8^ offer alternatives to conventional methods for photoaffinity labeling.

Ideally, a photoaffinity probe will display permeability and binding properties that are nearly identical to the parent probe, and upon photolysis will crosslink targets with high yield and extremely rapid kinetics, thereby enabling effective mapping of the target and interactome.^9^ Effective photoaffinity probes should also be capable of activating at wavelengths and operating under photolysis conditions that do not cause cytotoxicity or damage to the cellular targets under study. Additionally, the size and lipophilicity of the photoaffinity label should ideally be minimized in order to avoid negative effects on the function, solubility, permeability, and subcellular localization of the biological molecules under investigation.

In practice, it can be difficult to include all of the above-mentioned properties in a single probe. Classical probes based on benzophenones, aryl azides, and aryl diazirines display favorable crosslinking kinetics, but their large size and hydrophobicity can dominate the physicochemical properties of the probe.^3,5^ Because of their minimal size and favorable physicochemical properties, dialkyldiazirines have emerged as photoaffinity probes for a range of cellular interactions, including small molecule–protein interactions,^9,10^ including drugs,^11^ natural products,^12^ and fragments,^13^ as well as protein–protein interactions,^14^ nucleic acid–protein interactions,^15^ metabolic oligosaccharide engineering,^16^ lipid–protein interactions,^17^ and the study of biological membranes.^18^

The bimolecular capture of biological targets by diazirines during photoaffinity labeling is commonly attributed to carbene insertion processes. While this is the case for α-trifluoromethyl-α-phenyldiazirine,^19^ the situation is different for aliphatic diazirines. Diazirines produce both diazo compounds and carbene products upon irradiation.^19,20^ However, alkylcarbenes from aliphatic diazirines can undergo rapid intramolecular rearrangements with α-hydrogens, potentially limiting their ability to engage intermolecular targets.^21–27^ Meanwhile, aliphatic diazo compounds are reactive, and can undergo protonation at neutral pH to produce reactive diazonium compounds.^28^ The resulting diazonium salts are alkylating agents that can crosslink intermolecular targets,^29^ but with kinetics that are much slower than carbene crosslinking.^30,31^ Alkyldiazonium ions are therefore likely to have a substantially greater labeling radius than more reactive carbene intermediates in biological target identification investigations. Additionally, alkyldiazonium ions are likely to alkylate carboxylic acid residues when they do react with proteins to give ester bonds that could then be susceptible to hydrolysis by esterases in proteomic workflow and live cell assays.^29,32–34^

**Fig. 1:**
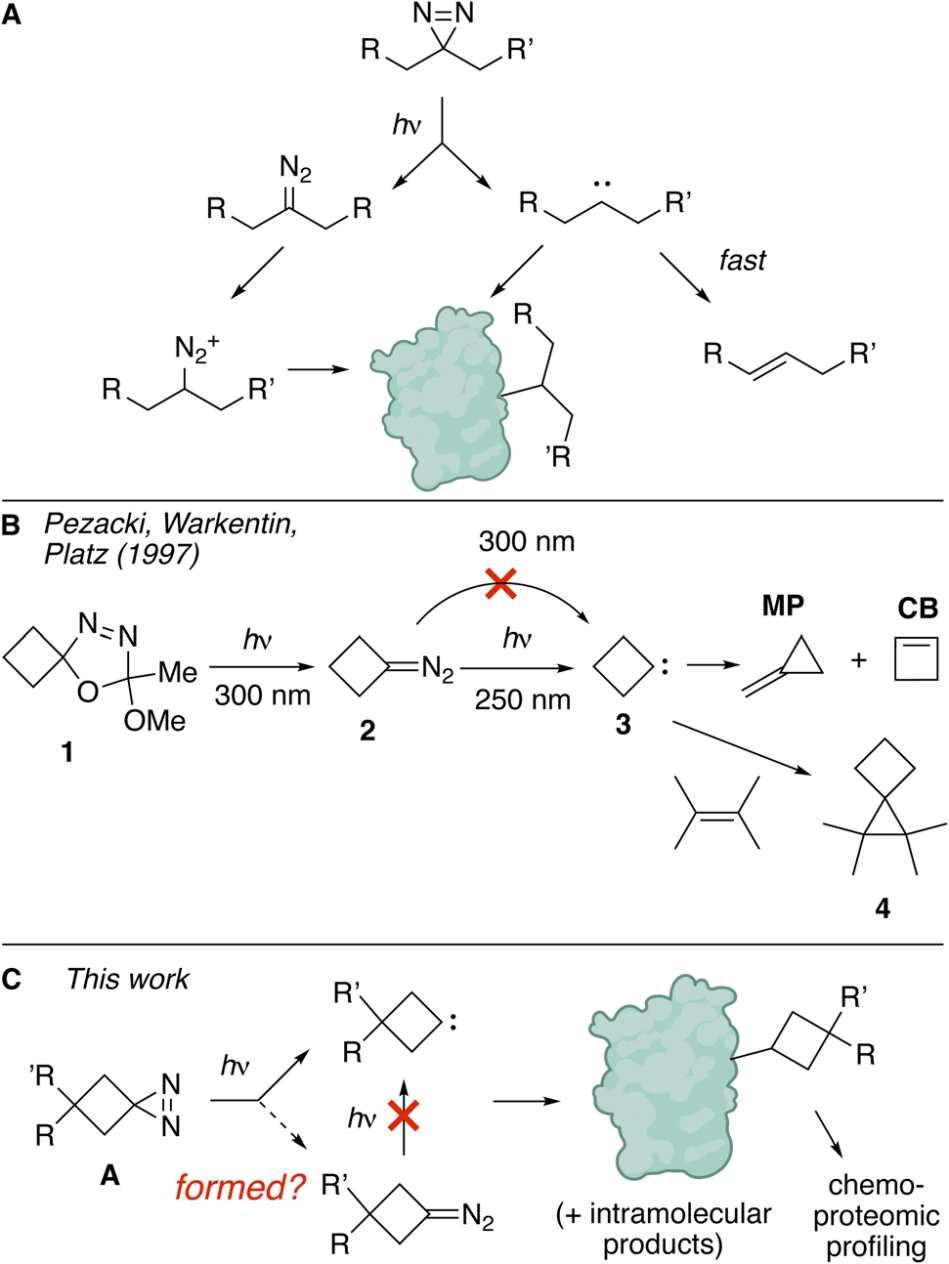
(A) ‘Linear’ diazirines produce both carbenes and diazo compounds upon photolysis. The carbene products are susceptible to intramolecular 1,2 H-shift. A primary labeling pathway involves protonation of the diazo compound and subsequent alkylation. (B) Prior studies showed spirocyclic oxadiazoline ketal 1 to be a precursor to diazocyclobutane 2, which can be used to produce cyclobutylidine 3 at 250 nm irradiation, but not at wavelengths longer than 300 nm with standard light sources. (C) We demonstrate that cyclobutanediazirines A produce carbene products upon photolysis. The diazocyclobutane is not observed, and does not serve as a photochemical precursor to the carbene.

In classic studies, Kirmse and Platz used competition experiments to understand the relative contributions of carbene and diazo intermediates in the photochemistry of diazirines fused to norbornane and bicyclo[2.1.1]hexane.^27^ Recently, we studied the photochemistry of linear dialkyldiazirines of the type commonly used in biological labeling.^35^ Deuterium labeling and diazo compound trapping experiments were employed to demonstrate that both carbene and diazo mechanisms operate, as well as a secondary alkylation process derived from a carbonyl ylide. It was shown that the carbene mechanism was primarily responsible for intramolecular alkene products, whereas bimolecular products are largely due in large part to alkylation chemistry (Fig. 1A). As discussed above, the mechanistic details have implications for chemical biology applications since carbene insertion reactions display different chemoselectivity and are substantially faster than alkylations of diazonium or oxocarbenium ions. Recently, Woo and coworkers evaluated the labeling preferences of alkyl and aryl diazirines.^36^ Consistent with the formation of diazo intermediates, they observed that alkyldiazirines exhibit preferential labeling of acidic amino acids in a pH-dependent manner and in live cells preferentially enrich highly acidic proteins or those embedded in membranes.

While linear dialkylcarbenes are susceptible to intramolecular rearrangement, observation of bimolecular reactions of alkylcarbenes in conformationally constrained systems is well documented.^23,37,38^ The smallest and perhaps simplest example of such a system is cyclobutylidene (3), which has been studied since 1960 ^37,39–42^ was first shown to undergo intermolecular 2 + 1 cycloaddition to give bicyclohexane products as early as 1984.^42^ Calculations predict that cyclobutylidene has a nonclassical carbene structure stablized *via* transannular interaction of the distal methylene group. Reflective of higher singlet stability in the nonclassical structure in cyclobutylidene is a computed singlet–triplet energy gap that favors the singlet excited state by 5.9 kcal mol^−1^ as compared to a singlet-triplet gap of only 1.3 kcal mol^−1^ for dimethylcarbene.^43^ Pezacki, Warkentin and Platz showed that cyclobutylidene could be generated from the laser flash photolysis (LFP, 308 nm) of oxadiazoline ketal 1.^37^ Unlike the 308 nm LFP result, conventional irradiation of 1 at 300 nm with UV-B bulbs was shown to produce diazocyclobutane 2 without the formation of carbene products. The difference was attributed to multiphoton excitation in the LFP method which does not occur in conventional photolysis. It was shown that diazocyclobutane 2 does not react thermally with tetramethylethylene to give 4,4,5,5-tetramethyl[2.3]spirohexane (4) *via* a 3 + 2/loss of nitrogen mechanism. However, carbene 3 can be generated *via* 250 nm irradiation and trapped by tetramethylethylene to give spirocyclohexane 4 in 21% yield. Also produced are methylenecyclopropane (MP) as the major intramolecular product and cyclobutene (CB) as a minor product.^37^MP is essentially the major product from carbene 3, whereas the excited state of diazocyclobutane 2 gives a 3.6 : 1 mixture of MP : CB. In agreement with experimental studies, computational investigations also predict that 1,2-carbon shift to give MP is an order of magnitude faster than 1,2-hydrogen migration to give CB, and three orders of magnitude faster than 1,3-hydrogen migration to give cyclobutane.^44^

Given these precedents, we hypothesized that spirocyclic diazirines A could serve as minimal probes capable of photoaffinity labeling through a ‘true’ carbene mechanism (Fig. 1C). Described in this study are demonstrations that these ‘cyclobutanediazirines’^45^ produce bimolecular and unimolecular products consistent with carbene intermediates.

Functionalized spirocyclic diazirines, including cyclobutanediazirine, were first reported in 2019 by Grygorenko and coworkers.^46^ In the course of our studies, Woo and coworkers described cyclobutanediazirines for applications in live cell photocrosslinking.^47^ They found that cyclobutanediazirines can label protein targets in cells and be utilized to map small molecule–protein binding sites, albeit with a significant reduction in labeling of targets. Mechanistically, cyclobutanediazirines were proposed to partition between diazocyclobutane and carbene intermediates. Cyclobutane diazirines displayed labeling that was less pH-dependent and a different reactivity profile relative to linear diazirine tags, and carbene labeling was proposed as the major bimolecular pathway. Diazocyclobutanes were proposed to undergo further photochemical transition to carbenes, rather than protonation/alkylation as has been described for linear carbenes. Only cyclobutene products were described as intramolecular byproducts of cyclobutanediazirine photolysis, whereas the methylenecyclopropane products expected from a carbene pathway were not described.

As discussed below, we propose a differing model of cyclobutanediazirine photolysis where the diazirine serves as the sole source of carbenes. Diazocyclobutanes are not observed in low temperature photolyses of a cyclobutanediazirine, whereas low temperature photolyses of linear diazirines do produce diazo compounds. Diazocyclobutane was prepared independently and shown to be thermally labile, and reactive toward water. Diazocyclobutanes are shown not to be photochemical precursors to carbenes upon illumination with UV-A (365 nm) light. Consistent with the formation of carbenes from cyclobutanediazirine photolysis, trapping with tetramethylethylene led to a cyclopropane product, and methylenecyclopropanes are observed as the major unimolecular rearrangement product of cyclobutyl diazirine photolyses along with the minor formation of cyclobutene products. Cell-based fluorescence and proteomics experiments demonstrated a marked reduction in labeling by the cyclobutyldiazirine-based probes compared to their linear diazirine counterparts, consistent with previous reports.^47^

## Results and discussion

### Synthesis

**Fig. 2:**
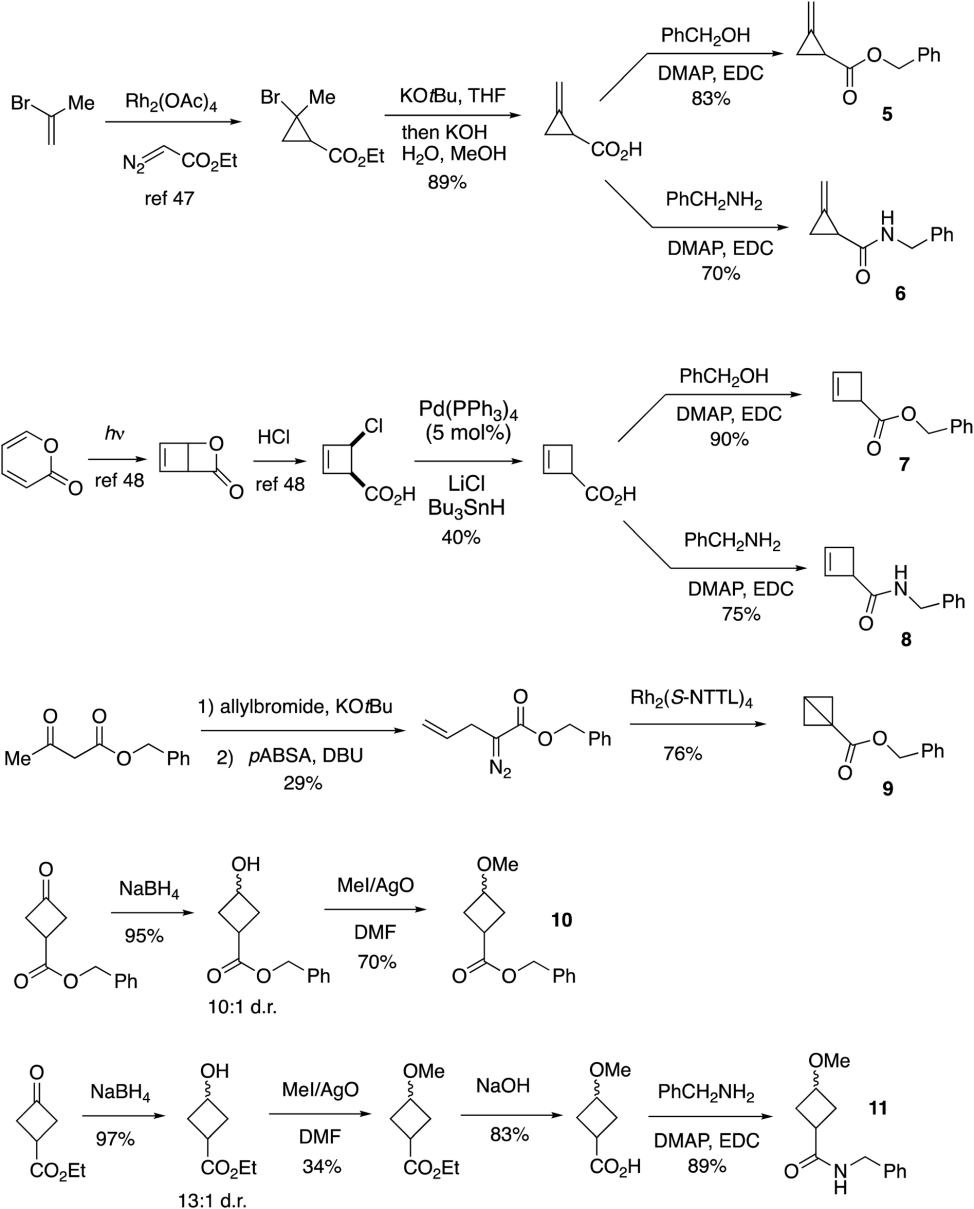
Synthesis of standards for mechanistic studies.

As standards for mechanistic studies with cyclobutanediazirines, we prepared benzylester and benzylamide analogs of methylenecyclopropanes, cyclobutenes, bicyclobutane, and cyclobutylmethylethers (Fig. 2). Cyclopropanation,^48^ dehydrohalogenation, and EDC/DMAP coupling provided access to methylenecyclopropanes 5 and 6. Cyclobut-2-ene carboxylic acid was prepared from *cis-*4-chloro-cyclobutenecarboxylic acid^49^ and served as precursor to ester 7 and amide 8. Benzyl bicyclo[1.1.0]butane-1-carboxylate 9 was prepared from benzyl acetoacetate by a sequence of allylation, diazo transfer, and intramolecular cyclopropanation.^50^ Cyclobutylmethylethers 10 and 11 were prepared as ≥10 : 1 syn : anti mixtures of diastereomers, using a sequence of NaBH_4_ and AgO/MeI mediated methylation as key steps (Fig. 2).

**Fig. 3:**
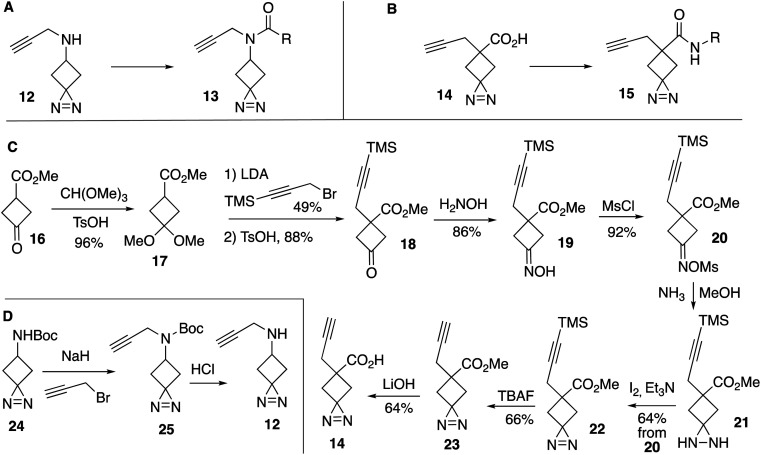
Synthesis of alkyne-functionalized cyclobutanediazirines. Probes based on (A) *N*-(propynyl)-1,2- diazaspiro[2.3]hex-1-en-5-amine and (B) 5-propynyl-1,2- diazaspiro[2.3]hex-1-ene-5-carboxylic acid. (C) Synthesis of 14. (D) Synthesis of 12 and 24**.**

As functionalized precursors to cyclobutylidienes, we synthesized a series of probes based on the alkyne-functionalized spirocyclic diazirines: *N*-(propynyl)-1,2-diazaspiro[2.3]hex-1-en-5-amine (13) and 5-propynyl-1,2-diazaspiro[2.3]hex-1-ene-5-carboxylic acid (15) (Fig. 3A and B). Straightforward acylation reactions converted 12 and 14 into structural analogs 13 and 15, respectively. Compound 14 was synthesized from methyl 3-oxocyclobutane-1-carboxylate (16) through conversion to ketal 17 in 96% yield (Fig. 3C) Introduction of a trimethylsilylpropargyl group followed by acid catalyzed deprotection gave 18 in 43% yield after 2 steps. A two-step sequence of oxime formation to give 19 and mesylation gave a 79% yield of 20, which could be converted to diazirine 22 in 64% yield through sequential treatment with ammonia to give 21 followed by oxidation with iodine. TBAF removed the silyl protecting group to give 22 in 66% yield, and saponification with LiOH gave carboxylic acid functionalized spirocyclic diazirine 23 in 64% yield. The amine-functionalized spirocyclic diazirine 12 was prepared by propargylation of commercially available 24 followed by Boc-deprotection (Fig. 3D).

### Evidence for carbene chemistry in photolyses of cyclobutanediazirines

While the chemistry of cyclobutylidene has been studied using a variety of precursors, including diazo compounds,^39^ tosylhydrazone salts,^41,42^ gem-dihalocyclobutanes,^41,42^ and oxadiazoline ketals,^37^ diazirine precursors had not been explored. As the nature of the precursor can have an effect on the relative contributions of carbene *vs.* precursor rearrangement chemistry, we sought to characterize the photolysis products of cyclobutanediazirine derivatives. The synthesis of a series of functionalized spirocyclic diazirines, including cyclobutanediazirine,^45^ had been reported by Grygorenko and coworkers.^46^

**Fig. 4:**
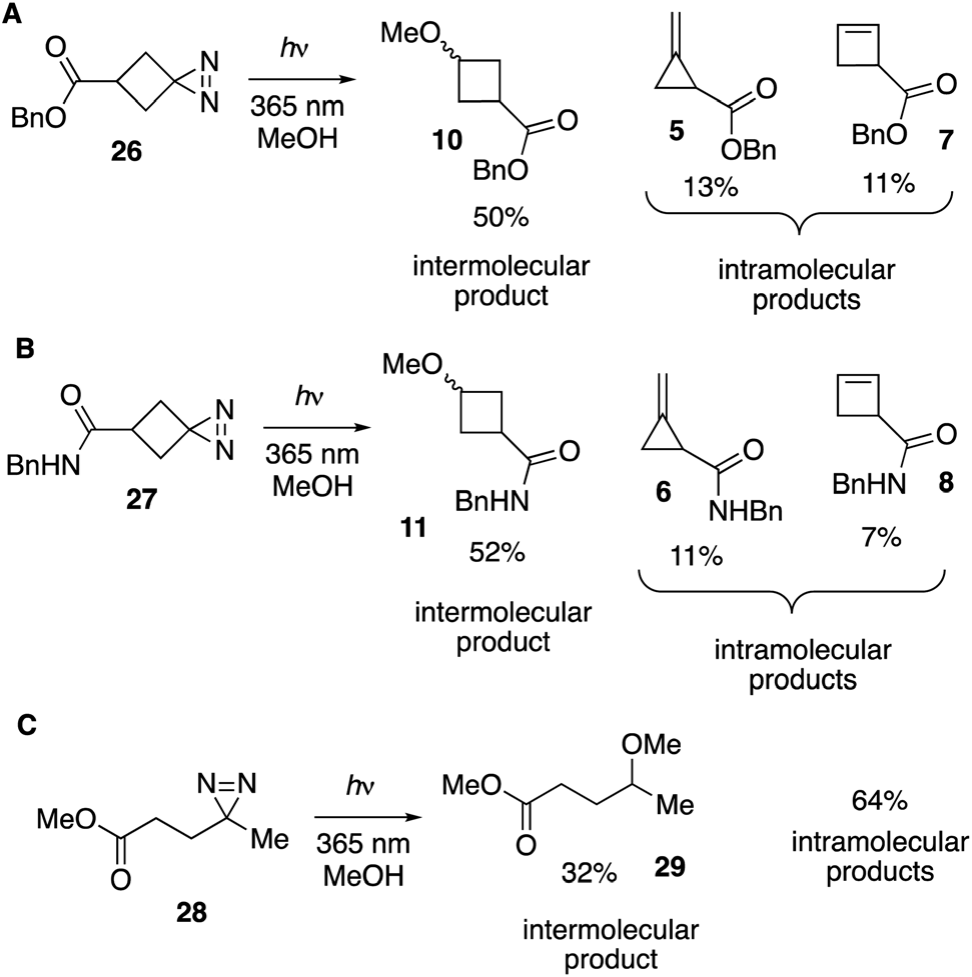
(A and B) Photolysis of spirocyclic diazirines in MeOH produces intermolecular products 10 and 11 in >50% yield, along with methylenecyclopropane and cyclobutene products of unimolecular rearrangement. (C) Photolysis of linear diazirine 28 produces intermolecular product 29 in 32% yield, along with 64% yield of alkene products of unimolecular rearrangement.

As shown in Fig. 4, the UV-A photolyses of spirocyclic diazirine 26 in methanol gave rise to 50% of the cyclopropylmethyl ether product 10 as well as methylenecyclopropane 5 (13%) and cyclobutene 7 (11%) (Fig. 4A). Benzyl bicyclo[1.1.0]butane-1-carboxylate (9, Fig. 2) was not observed. Similarly, amide analog 27 led to ether 11, methylenecyclopropane 6 and cyclobutene 8 in 52%, 11% and 7% yields, respectively (Fig. 4B). Yields were measured by ^1^H NMR analyses of the crude mixtures and structural assignments were made by comparison to standards that were independently synthesized (Fig. 2). Suggestive of contributing carbene chemistry, the yields of the bimolecular MeOH-adducts 10 (50%) and 11 (52%) are higher than the yield of MeOH-adduct 29 (32%) previously described for photolysis of a linear diazirine 28 (Fig. 4C).^35^ Also consistent with a contributing carbene mechanism is formation of methylenecyclopropanes as the major unimolecular products,^37,39–42^ while the observation of cyclobutene formation indicates that a non-carbene mechanism is also competitive.^37^

**Fig. 5:**
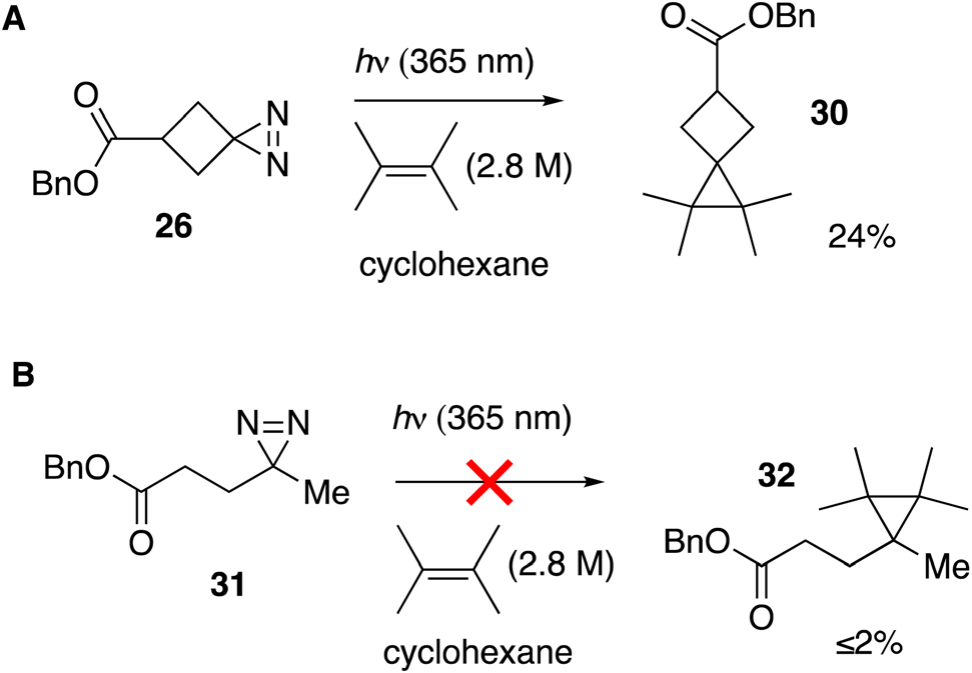
(A) Cyclopropanation with cyclobutanediazirine 26 succeeds, whereas (B) linear diazirine 28 does not give cyclopropane products.

We tested whether a carbene intermediate from photolysis of diazirine 26 could be intercepted *via* alkene cyclopropanation. Earlier studies have shown that 4,4,5,5-tetramethyl[2.3]spirohexane products are derived from the capture of cyclobutylidene, but not from diazocyclobutane.^37^ When 26 is irradiated in the presence of 2 M tetramethylethylene (TME) in cyclohexane with UV-A light for 120 minutes, carbene trapping adduct 30 was formed in 24% isolated yield (Fig. 5A). Our result is comparable to the 21% yield obtained by Pezacki *et al.* for carbene generated *via* 250 nm photolysis of an oxadiazolone ketal precursor to produce 4,4,5,5-tetramethyl[2.3]spirohexane 4.^37^ Also detected by GC along with 30 were intramolecular rearrangement products methylenecyclopropane 5 and cyclobutene 7. An analogous photolysis experiment with diazirine 31 in the presence of TME gave the expected intramolecular products but produced ≤2% of cyclopropane 32 by GC analysis (Fig. 5B).

### Decomposition of diazocyclobutane is accelerated by water, but not UV-A light

**Fig. 6:**
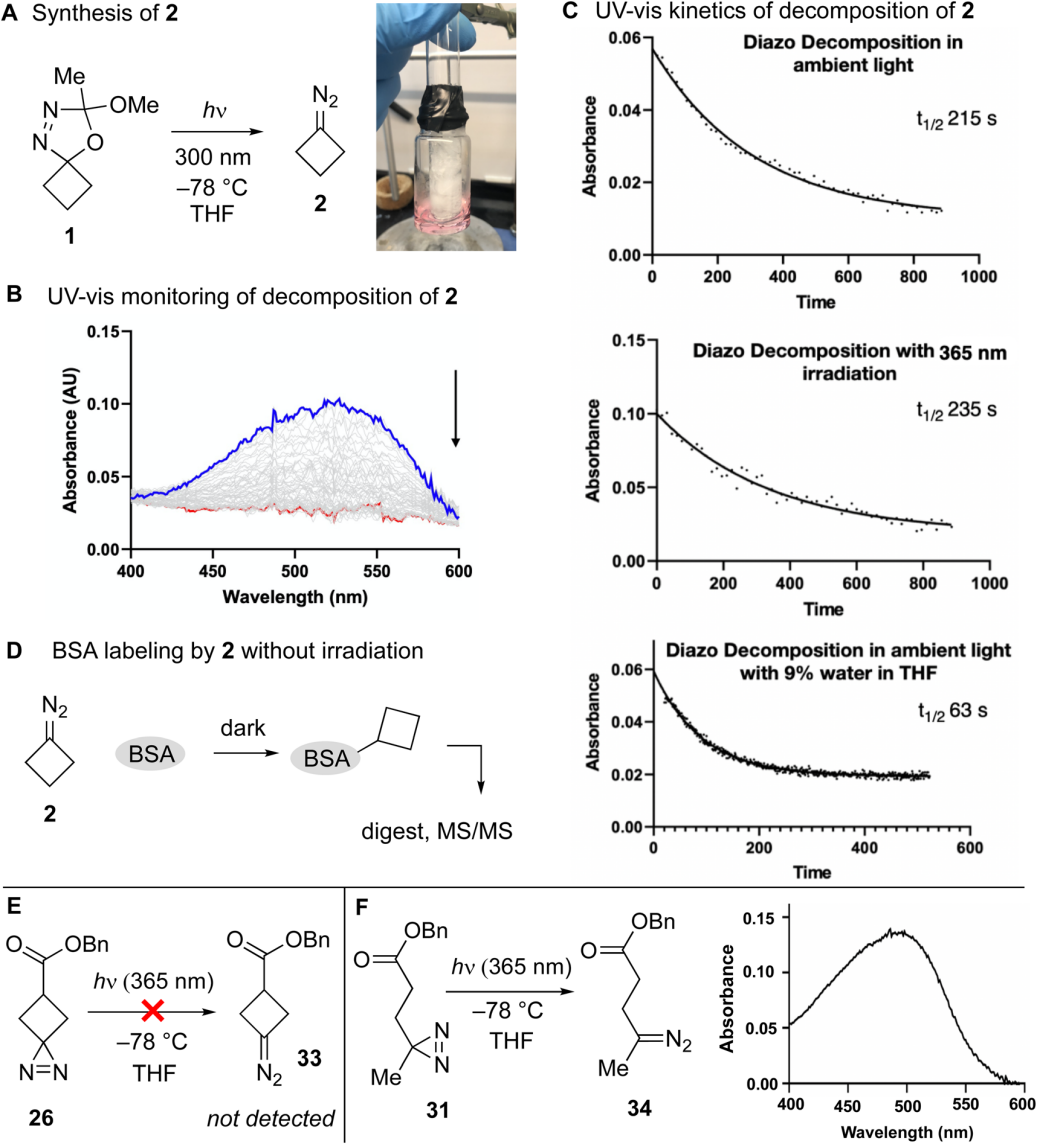
(A) Low temperature photolysis of 1 produces 2 as a pink solution. (B) Thermal decomposition of diazocyclobutane 2 can be monitored by UV-vis through time-course monitoring of disappearance of the characteristic absorbance at 525 nm. (C) The rate of decomposition of 2 is unaffected by irradiation with UV-A light centered at 365 nm, but is accelarated by the addition of water. (D) Diazocyclobutane 2 labels BSA without irradiation. (E) Irradiation of 26 in THF at −78 °C does not produce appreciable amounts of 33, whereas (F) similar irradation of 31 produces 34 as a pink solution with absorbance at 500 nm in the visible spectrum.

The irradiation of diazirines with UV-A (365 nm) light may give rise to diazo compounds as well as carbenes.^19,27,51^ To study whether a diazocyclobutane intermediate could lead to carbene formation, we revisited the Pezacki and Warkentin synthesis of diazocyclobutane (2). This was accomplished *via* UV-B (300 nm) irradiation of 0.1 M solutions of 1 for 15 min in anhydrous THF (Fig. 6A). The diazo compound is thermally sensitive and we found it was best prepared by low-temperature photolysis using a −78 °C cold finger. As expected, the diazo compound is bright pink in solution with a low-intensity absorption in the UV-vis at 525 nm.^52^

We measured the decay of the absorption at 525 nm associated with 2 with *in situ* spectroscopic monitoring at 24 °C in a thermostated cuvette (Fig. 6B). The diazo compound is labile and decays in anhydrous THF with an average half-life of 215 seconds (Fig. 6C, top right). By comparison, Platz reports the stability of a dichloromethane solution of acyclic dipropyldiazomethane to be more stable (*t*_1/2_ ∼ 1 hour).^51^ The half-life of 2 was insensitive to irradiation by UV-A light (365 nm, 20 mW cm^−2^) (Fig. 6C, middle). This result is consistent with the earlier observations of Pezacki and Warkentin, who found that diazocyclobutane 2 was not affected by 300 nm light, but gave carbene products when photolyzed with 250 nm light.^37^ By contrast, 2 is water sensitive. Upon mixing 0.5 mL of a THF solution of 2 with 0.5 mL water or PBS, the pink color disappeared rapidly. In an experiment conducted with *in situ* monitoring of diazo disappearance, 100 μL water was added to 1 mL of a THF solution of 2–4 (Fig. 6C, bottom). Relative to an anhydrous sample, the rate of decomposition increases >3-fold with an average half-life of 63 seconds. This fast reactivity of the diazocyclobutane is consistent with earlier studies on rates diazonium ion formation *via* diazocycloalkane protonation under anhydrous conditions, where strain-relief was proposed to explain the faster reactivity of diazocyclobutane relative to diazocyclopentane or diazocyclohexane.^53^

To investigate whether diazocyclobutane (2) is capable of non-photochemical protein alkylation, we investigated the reaction of 2 toward bovine serum albumin (BSA) followed by protein digestion and MS/MS analysis (Fig. 6D). For these experiments, a high concentration of 2 was utilized to mimic the conditions of proximity labeling. Thus, a 150 μM solution of BSA in ultrapure H_2_O was mixed with a solution of 2 in THF which was freshly prepared from 1 (10 mM) by low temperature photolysis as described above. The pink color of 2 quickly dissipated upon mixing accompanied by the evolution of nitrogen gas. After 1 hour, the protein was precipitated, pelleted and resuspended in 50 mM ammonium bicarbonate with 6 M urea. Samples were reduced with DTT, alkylated with iodoacetamide, trypsin digested and then processed for LC-MS/MS analysis. A mass shift of +54 Da corresponding to the addition of a cyclobutane with the loss of 1 proton from the nucleophile was screened for at lysine, cysteine, serine, histidine, aspartate, and glutamate residues. It was determined that 229 specific unique BSA amino acid side chains experienced the corresponding +54 Da addition: 59 lysine, 33 cysteine, 23 serine, 16 histidine, 40 aspartate, and 58 glutamate. In comparing this with the canonical BSA sequence containing 60 lysine, 35 cysteine, 32 serine, 17 histidine, 40 aspartate, and 59 glutamate residues, the majority of each residue type formed an adduct. The results suggest that, if cyclobutanediazirine photolysis produces diazocyclobutane intermediates, that non-photochemical alkylation of proteins by diazocyclobutane is possible.

We also considered whether photolysis of diazirine 26 in anhydrous THF would lead to the detectable production of diazocyclobutane 33 upon irradiation at 365 nm. Analogous to experiments above, UV-vis spectroscopy with monitoring at 500–525 nm was used to probe for the formation of diazo compound. Several light sources were tried, as was photolysis at r.t. or −78 °C, but in no case were we able to detect appreciable signal at >500 nm, and while reactions were carried out to completion and monitored at different time points, in no case did we observe the expected pink color associated with diazocyclobutane formation. By contrast, 365 nm irradiation of the linear diazirine 31 in THF produced a peach-colored solution with an absorption at 500 nm attributed to diazo compound 34 (Fig. 6F).

Together, these results indicate that 365 nm photolysis of cyclobutanediazirines can produce carbene intermediates as evidenced by formation of their expected bimolecular and unimolecular products. Diazocyclobutane (2), when synthesized from a different precursor, reacts rapidly with water and can serve as a protein alkylating agent. Diazocyclobutane 2 does not undergo photochemistry at 365 nm. Unlike linear diazirines, cyclobutanediazirine 26 does not give detectable amounts of diazo compound 33 upon low temperature photolysis in organic solvent, suggesting that 33 is either not formed or is especially labile.

### Comparative studies of linear and cyclobutanediazirine probes in live cells

**Fig. 7:**
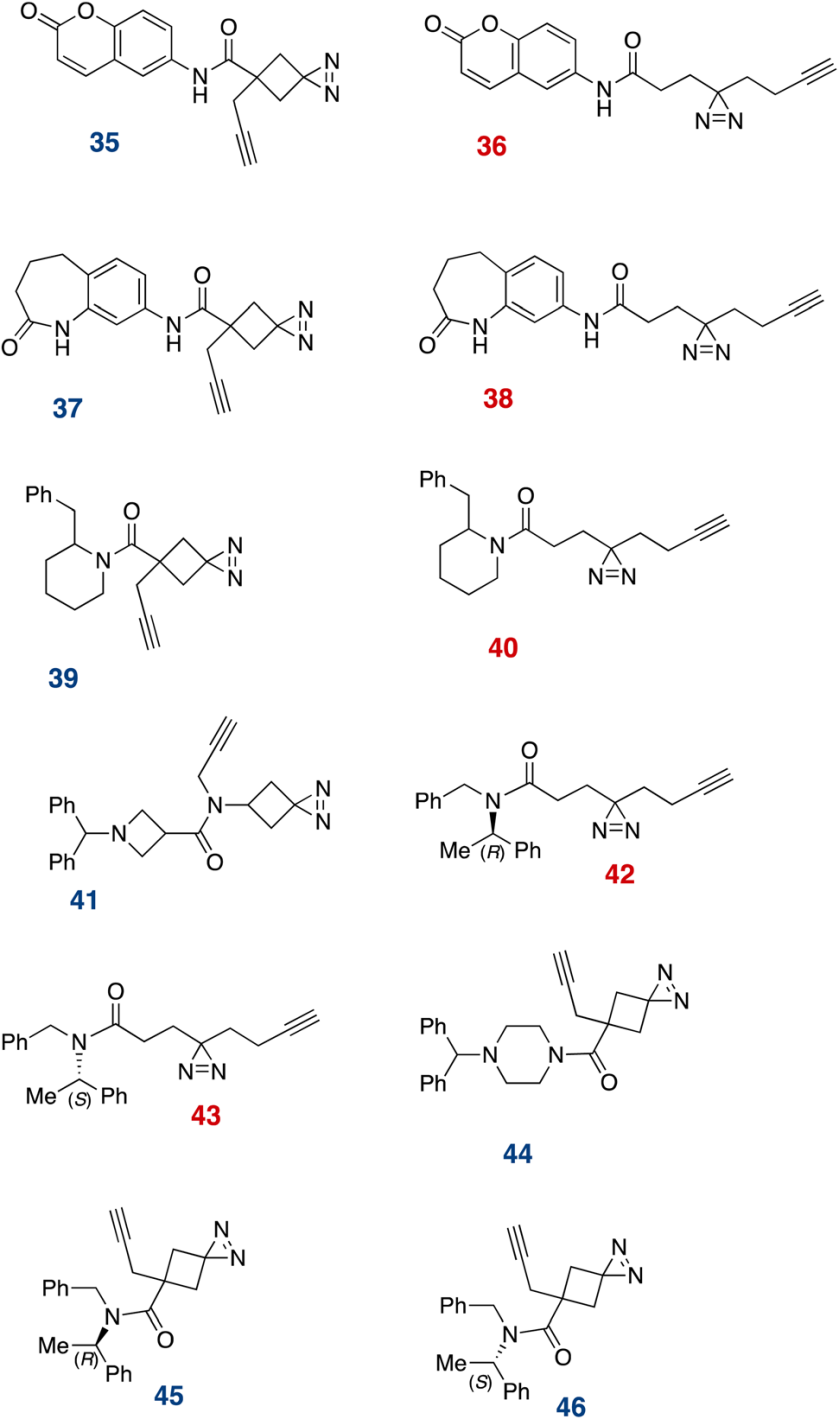
Diazirine probes used for live cell labeling.

**Fig. 8:**
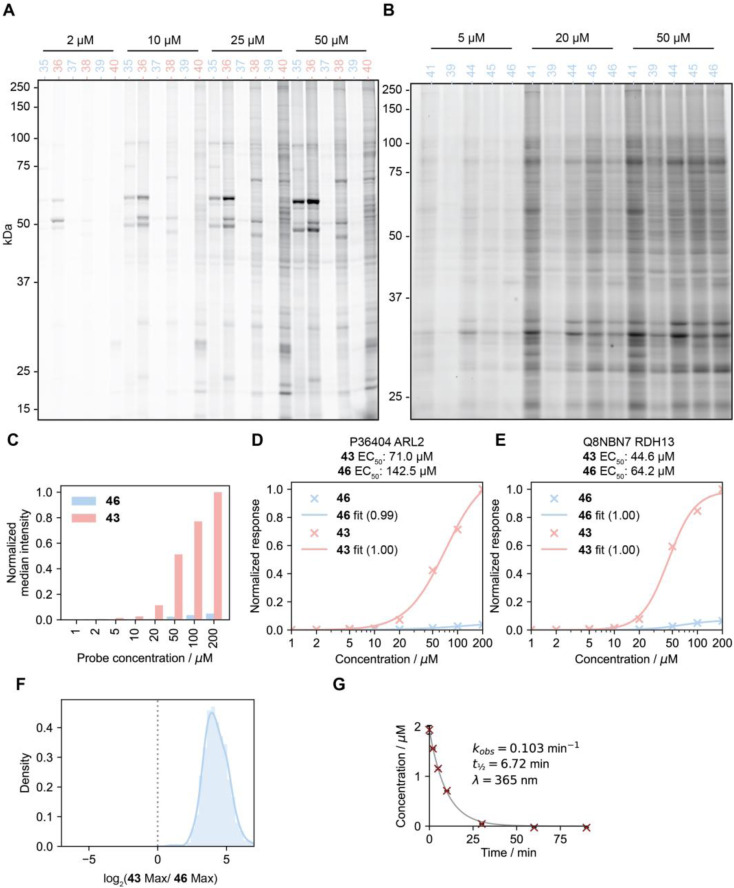
Cyclobutanediazirines exhibit reduced proteomic labeling relative to linear dialkyl diazirines. (A and B) Soluble lysate fraction derived from HEK293T cells treated with cyclobutyl (blue) and linear dialkyl diazirine probes (red) for 30 min followed by UV irradiation (365 nm for 20 min), conjugated to TAMRA-azide, and visualized by in-gel fluorescence. (C) Proteomic median abundances of targets enriched by 43 and 46 at different concentrations. (D and E) Proteomic abundances for representative proteins treated with different concentrations of probes 43 and 46. (F) Comparing the ratios of maximum intensity for proteins that possess similar 43 and 46 EC_50_ values (within 20%) shows that the linear dialkyl diazirine probes label proteins with approximately 16-fold greater efficiency. (G) First-order decay of 41 upon UV irradiation (365 nm). Error bars represent standard deviation, *n* = 3.

We next comparatively assessed the general proteomic profiles for a number of linear and cyclobutyl diazirine-based fragment probes^13,54^ derived from coumarin (35 and 36), tetrahydro-1-benzazepin-2-one (37 and 38), 2-benzylpiperadine (39 and 40), α,α-diphenylmethylazetidine-3-carboxamide (41), methyl benzylamine (42, 43, 45, 46), and benzhydrylpiperazine (44, Fig. 7). We first profiled the interactions of these probes *via* in-gel fluorescence imaging. Briefly, HEK293T cells were treated with the probe compounds at a range of concentrations for 30 min, before UV irradiation (365 nm, 4 °C, 20 min). The cells were then lysed, and the protein-bound probes were ligated to tetramethylrhodamine through a copper-catalyzed azide–alkyne 1,3-dipolar cycloaddition reaction. We found, in agreement with Woo and coworkers^47^ that the cyclobutanediazirines generally exhibited much less labeling than their linear counterparts (Fig. 8A and S7A†). However, when visualized separately, they do show dose- and target group-dependent protein labeling (Fig. 8B).

To further investigate these trends, we compared the profiles of linear and cyclobutyl diazirine *via* quantitative MS-based proteomics. Here, cells were treated as above and their interactions identified and quantified *via* TMT-based proteomics, as previously described.^54^ To quantify the relative enrichment of each matched probe, we treated cells with probe pairs at different probe concentrations, revealing a much lower median abundance in the samples treated with the corresponding cyclobutanediazirine probe compared to those treated with the linear dialkyl probe (Fig. 8C–E, S7B and C†), consistent with gel-based profiles. To determine if these disparate abundances were due to differences in protein affinities, rather than reactivity differences, we examined the enrichment profiles of proteins that display similar apparent binding affinities for the same molecular recognition group (within 20%). Here, we observed, once again, substantially higher signals for interactions captured by the corresponding linear dialkyl probe, which, in aggregate, produced a signal intensity an order of magnitude greater than that of the cyclobutanediazirine (Fig. 8F and S7D†) for proteins of similar apparent affinities. Finally, we note that the cyclobutanediazirine probes exhibited similar decomposition kinetics to the linear diazirine analog and are largely consumed within the timeframe of the labeling experiment (Fig. 8G). Collectively, these data indicate that cyclobutyl diazirine probes possess lower protein capture efficiency relative to their linear dialkyl counterparts in chemoproteomic experiments.

## Conclusions

These studies illustrate the differences in the photochemical behavior of linear diazirines and spirocyclic cyclobutanediazirines with the latter exhibiting a larger contribution from carbene chemistry and a reduced contribution of diazo chemistry. Suggestive of a contributing carbene mechanism, 365 nm irradiation of cyclobutanediazirines produces methylenecyclopropane products as has been observed in earlier studies of cyclobutylidene formed from non-diazirine precursors.^37,39–42^ Additionally, these photolyses produce relatively high (≥50%) yields of bimolecular adducts with MeOH solvent. Additional support for a trappable carbene intermediate was provided by the observation of [2 + 1] adduct 30 from the photolysis of 26 with tetramethylethylene.

If the irradiation of cyclobutanediazirines were to produce diazocyclobutane products, they would be rapidly protonated and could be capable of reacting with protein targets. Our experiments show that diazocyclobutane reacts rapidly with water and is capable of alkylating BSA. Our experiments further show that diazocyclobutane can be ruled out as a meaningful precursor to cyclobutylidene under 365 nm photolysis conditions. While irradiation of linear diazirines produces a diazoalkane product, attempts to produce a diazocyclobutane *via* low temperature irradiation of a cyclobutanediazirine were unsuccessful. The reason that carbene formation is favored may be connected to the stability afforded by the non-classical, transannular bonding in cyclobutylidene and the associated transition state for carbene formation.^43^ A model where carbene formation is favored over diazo formation under room temperature, aqueous conditions offers a straightforward explanation for the relatively low labeling yields that are observed in cellular experiments. Thus, linear diazirines can produce relatively long-lived diazoalkanes, which are also capable of labeling proteins, whereas cyclobutanediazirines are proposed to give minimal diazo product and labeling occurs predominantly *via* a carbene mechanism. This model is consistent with the observations and conclusions about carbene involvement by Woo and coworkers,^47^ while providing a more straightforward explanation for the observed reduction in overall labeling in cellular studies as well as the observed production of methylenecyclopropane product *in vitro*. For applications in proteomics, cyclobutanediazirines provide an alternate tool to conventional, linear dialkyldiazirines that, consistent with a carbene crosslinking mechanism, produce lower levels of overall labeling but also lower levels of background labeling. Given the high reactivity and short lifetime of cyclobutylidene intermediates, cyclobutanediazirines should prove especially useful in applications such as small molecule target identification where a small radius of labeling is an especially important consideration.

## Author contributions

Jessica O’Brien carried out mechanistic photolysis experiments and synthesized diazirine precursors and authentic standards of products. Louis Conway carried out whole cell proteomic studies and data analysis. Paramesh Ramaraj carried out low temperature photolyses and characterized kinetics of diazo compound stability under varied conditions. Appaso Jadhav synthesized synthesized diazirine probes, conducted gel electrophoreses, and studies kinetics of diazirine decomposition. Jun Jin synthesized alkyne-functionalized cyclobutanediazirines. Jason Dutra synthesized diazirine probes used for live cell labeling. Parrish Evers carried out labeling experiments of BSA and *in vitro* proteomic experiments. Shadi S. Masoud synthesized oxadiazoline ketal 1. Manuel Schupp contributed to the synthesis of cyclobut-2-ene carboxylic acid, used as a GC and NMR standard. Iakovos Saridakis contributed to the synthesis of cyclobut-2-ene carboxylic acid, used as a GC and NMR standard. Yong Chen contributed to the synthesis of cyclobut-2-ene carboxylic acid, used as a GC and NMR standard. Nuno Maulide designed the synthesis of synthesis of cyclobut-2-ene carboxylic acid. John P. Pezacki designed and oversaw BSA labeling and in vitro experiments with 1, and provided mechanistic insight. Christopher W. am Ende co-led the study. Christopher G. Parker co-led the study. Joseph M. Fox co-led the study.

## Conflicts of interest

The authors declare the following competing financial interest(s): J. O’B., J. K. D. and C. W. a. E. are employees of Pfizer Inc; J. J. is an employee of BioDuro-Sundia.

## Supplementary Material

SC-015-D4SC04238G-s001

SC-015-D4SC04238G-s002

SC-015-D4SC04238G-s003

## Data Availability

The data supporting this article have been included as part of the ESI.†
