# Supplementary material for: Mechanistic differences between linear vs. spirocyclic dialkyldiazirine probes for photoaffinity labeling
Source: Chem Sci. 2024 Aug 13;15(37):15463–73. doi: 10.1039/d4sc04238g (PMC11372447; doi:10.1039/d4sc04238g)

Supplementary Information (SI) for Chemical Science.
This journal is © The Royal Society of Chemistry 2024

Supporting Information

Mechanistic Differences between Linear vs Spirocyclic Dialkyldiazirine Probes for Photoaffinity Labeling

Jessica G. K. O'Brien,[#] Louis P. Conway,[#] Paramesh K. Ramaraj, Appaso M. Jadhav, Jun Jin, Jason K. Dutra, Parrish Evers, Shadi S. Masoud, Manuel Schupp, Iakovos Saridakis, Yong Chen, Nuno Maulide, John P. Pezacki, Christopher W. am Ende*, Christopher G. Parker*, Joseph M. Fox*

General Considerations	S2
Synthesis	S2
Photolysis Experiments	S29
Studies on Diazocyclobutane	S34
Studies of Diazo formation via Diazirine Photolysis	S36
BSA labeling by **2** and mass spectrometry	S37
Treatment of Live Cells with Fully-Functionalized Probes	S40
In-gel Fluorescence Experiments	S40
TMT-Labeled Streptavidin Enrichment Proteomics	S42
Mass Spectrometric Analysis and Data Analysis	S44
Decomposition Kinetics Studies	S44
References	S45
NMR spectra	S46

General Considerations

All syntheses were conducted under nitrogen in glassware that had been flame dried and cooled under nitrogen. Compounds were chromatographed on silica gel (ICN SiliTech 32-62D, 60A). Automated column chromatography was performed on a Teledyne Isco Combiflash. High-resolution mass spectra were obtained on a Thermo Q-Exactive Orbitrap using heated electrospray ionization (HESI). 1,2-Diazaspiro[2.3]hex-1-ene-5-carboxylic acid was purchased from Enamine. Preparations of **36**,[1] **40**[1], **42**,[2] and **43**[2] have been described. All other reagents were purchased from MilliporeSigma, Combi-Blocks, Acros Organics, Alfa Aesar, Oakwood Chemical, TCI Chemicals, and Frontier Scientific. All reagents were used without further purification. Solvents were purchased from Thermo Fisher Chemical, Acros Organics, Decon Laboratories Inc., Mediatech, Inc., and MilliporeSigma. Methanol labeling experiments were conducted with a 365 nm lamp with light intensity 19 mW/cm2 (Spectroline model XX15A longwave UV 365 nm 120 volts/60 hz/ 0.7 amps/84 watts). Light power density was measured using a ThorLabs PM125D Optical Power Meter Kit with 2 mW to 10 W Thermal Sensor (0.19 - 25µm). In-gel fluorescence, MS proteomics, and decomposition kinetics studies were performed as described previously.[3]

Synthesis

Benzyl 1,2-diazaspiro[2.3]hex-1-ene-5-carboxylate (26)

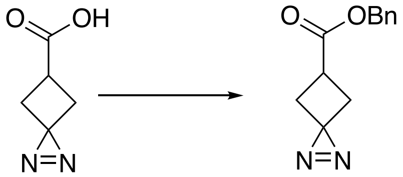

A flame-dried round bottom flask was charged with DMAP (26 mg, 0.21 mmol, 0.11 equiv.) and 1,2-diazaspiro[2.3]hex-1-ene-5-carboxylic acid (237 mg, 1.88 mmol) under N_2. Methylene chloride (8 mL) was added, followed by benzyl alcohol (0.24 g, 2.2 mmol, 1.2 equiv.). EDC (455 mg, 2.37 mmol, 1.26 equiv.) was added in one portion. The solution was allowed to stir at room temperature overnight. The next day, the reaction mixture was loaded directly on silica gel and chromatographed with an eluent of 20% ethyl acetate in hexanes. The title compound was obtained as a clear oil (400 mg, 1.85 mmol, 98% yield).

1H NMR (400 MHz, $CDCl_3$) δ 7.37-7.34 (m, 5H), 5.19 (s, 2H), 3.31-3.23 (m, 1H),

2.65-2.60 (m, 2H), 2.46-2.40 (m, 2H) ppm; 13C NMR (101 MHz, $CDCl_3$) δ 174.1 (C), 135.9 (C), 128.9 (CH), 128.6 (CH), 128.5 (CH), 67.1 (CH_2), 34.5 (CH_2), 30.4 (CH), 28.1 (C) ppm; HRMS (ESI+) [M+H]+ Calculated for $C_{12}H_{13}O_2N_2^+$ 217.0979; found 217.0968.

2-Methylenecyclopropane carboxylic acid

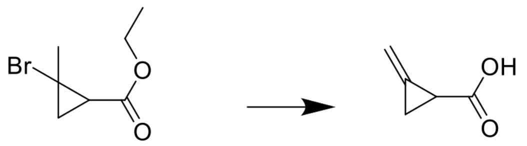

A flame-dried round bottom flask was charged with diasteromers of ethyl 2-bromo-2-methylcyclopropane carboxylate[4] (2.01 g, 9.71 mmol) and THF (30 ml) under N_2. The solution was chilled in an ice bath. Potassium *tert*-butoxide (1.64 g, 14.6 mmol) was added in three portions over 15 minutes. After the last addition of base, the solution was taken off the ice bath and allowed to stir at room temperature for one hour. The solution was then concentrated to reduce volume, followed by addition of water (15 ml) and methanol (20 ml). Sodium hydroxide (2.10 g, 52.5 mmol, 5.41 equiv.) was added and the solution turned orange over time. The reaction was left to stir overnight. The next day, the reaction mixture was extracted with methylene chloride and the aqueous phase was collected, acidified to pH 1 with concentrated hydrochloric acid, and extracted with methylene chloride (3x). The title compound was dried with sodium sulfate, filtered, and concentrated to obtain a clear orange oil (847 mg, 8.63 mmol, 89% yield).

^1H NMR (400 MHz, $CDCl_3$) δ 5.56 (s, 2H), 2.29-2.24 (m, 1H), 1.91-1.86 (m, 1H), 1.74-1.69 (m, 1H) ppm; ^{13}C NMR (101 MHz, $CDCl_3$) δ 178.8 (C), 130.0 (C), 105.3 (CH_2), 17.9 (CH), 12.4 (CH_2) ppm; HRMS (ESI+) $[M+H]^+$ Calculated for $C_5H_7O_2^+$ 99.0446; found 99.0445.

Benzyl 2-methylenecyclopropane carboxylate (5)

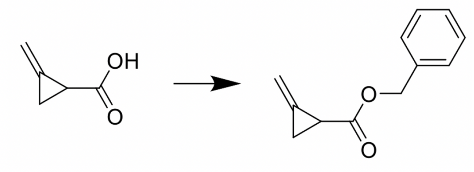

A flame-dried round bottom flask was charged with DMAP (74.9 mg, 0.613 mmol) and EDC (1.48 g, 7.72 mmol) under an atmosphere of N_2. Benzyl alcohol (742 mg, 6.86 mmol) was added, followed by 2-methylenecyclopropane carboxylic acid (612.8 mg, 6.247 mmol) dissolved in methylene chloride (14 mL). The solution was allowed to stir at room temperature overnight. The next day, the reaction mixture was loaded directly on silica gel and chromatographed with an eluent of 20% ethyl acetate in hexanes. The title compound was obtained as a clear oil (974 mg, 5.17 mmol, 83% yield).

^{1}H NMR (600 MHz, CD_3OD) δ 7.39-7.35 (m, 5H), 5.54-5.52 (m, 2H), 5.16 (s, 2H), 2.41-2.38 (m, 1H), 1.84-1.81 (m, 1H), 1.77-1.73 (m, 1H) ppm; ^{13}C NMR (101 MHz, $CDCl_3$) δ 172.2 (C), 136.1 (C), 130.3 (C), 128.7 (CH), 128.4 (CH), 128.3 (CH), 105.0 (CH_2), 66.6 (CH_2), 18.2 (CH), 11.9 (CH_2) ppm; HRMS (ESI+) $[M+H]^+$ Calculated for $C_{12}H_{13}O_2^+$ 189.0916; found 189.0908.

Benzyl 3-oxocyclobutane carboxylate

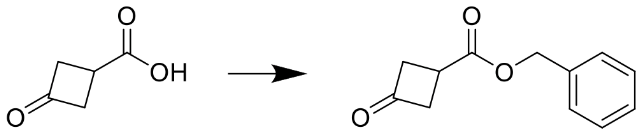

A flame-dried round bottom flask was charged with DMAP (169 mg, 1.38 mmol), EDC (3.19 g, 16.6 mmol), and 3-oxocyclobutane carboxylic acid (1.52 g, 13.321 mmol) under an atmosphere of N_2. Benzyl alcohol (1.57 g, 14.5 mmol) was added, followed by methylene chloride (30 mL). The solution was allowed to stir at room temperature overnight. The next day, the reaction mixture was loaded directly on a silica gel and chromatographed with an eluent of 30% ethyl acetate in hexanes. The title compound was obtained as a clear oil (2.60 g, 12.7 mmol, 96% yield). ^{1}H NMR and ^{13}C NMR data agreed with the spectra reported in the literature.[5]

Diastereomers of Benzyl 3-hydroxycyclobutane carboxylate, 10:1 d.r.

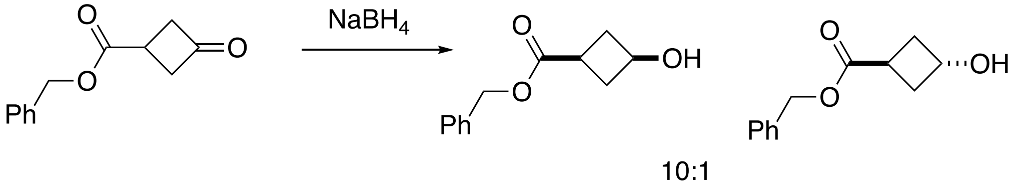

A flame-dried round bottom flask was charged with benzyl 3-oxocyclobutane carboxylate (2.60 g, 12.7 mmol), THF (33 mL), and methanol (1.6 mL) under N_2. The solution was chilled in an ice bath. Sodium borohydride (263 mg, 6.95 mmol) was added. The reaction was monitored by TLC and shown to be complete in 30 minutes. The reaction mixture was then diluted with ethyl acetate and extracted with aqueous sodium bicarbonate. The aqueous layer was extracted with ethyl acetate (2x). The organic layers were combined and extracted with brine, followed by drying with sodium sulfate. The solution was concentrated under vacuum and then chromatographed with an eluent of 60% ethyl acetate in hexanes. The title compound was obtained as a clear oil (2.49 g, 12.1 mmol, 95% yield) that was a 10:1 mixture of diastereomers as determined by ^{1}H NMR spectroscopy.

^{1}H NMR (400 MHz, $CDCl_3$) δ 7.39-7.30 (m, 5H), 5.12 (s, 2H), 4.23-4.15 (m, 1H), 2.69-2.56 (m, 3H), 2.23-2.15 (m, 2H), 2.0–1.8 (bs, 1H) ppm; ^{13}C NMR (101 MHz, CDCl3) 175.9 (C), 174.8 (C), 136.0 (C), 128.7 (CH), 128.4 (CH), 128.3 (CH), 66.7 (CH_2), 63.5 (CH), 37.2 (CH_2), 36.2 (CH_2), 29.1 (CH) ppm; additional peaks due to the minor diastereomer: 175.9 (C), 66.6 (CH_2), 65.8 (CH), 36.2 (CH_2), 31.3 (CH), HRMS (ESI+) [M+H]+ Calculated for $C_{12}H_{15}O_3^+$ 207.1021; found 207.1013.

Benzyl 3-methoxycyclobutanecarboxylate (10), 10:1 d.r.

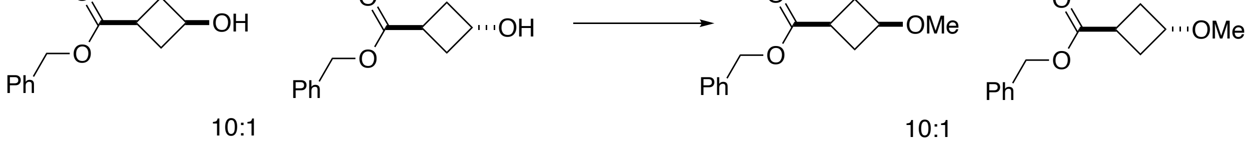

A flame-dried round bottom flask was charged with silver oxide (1.60 g, 7.49 mmol), benzyl 3-hydroxycyclobutane carboxylate (2.15 g, 10.4 mmol) and DMF (20 mL) under an atmosphere of

N_2. Methyl iodide (2.96 g, 20.9 mmol) was added, then the mixture was heated to 45 °C. After an hour, additional silver oxide (1.60 g, 7.49 mmol) and methyl iodide (2.96 g, 20.9 mmol) were added. The mixture was allowed to stir overnight. The next day, the mixture was filtered, concentrated, then extracted with water and ethyl acetate. The organic layer was dried with sodium sulfate and filtered, followed by chromatography with an eluent of 0-30% ethyl acetate in hexanes. The title compound was obtained as a clear oil (1.60 g, 7.26 mmol, 70% yield).

^{1}H NMR (600 MHz, CD_3OD) δ 7.38-7.35 (m, 5H), 5.16 (s, 2H), 3.89-3.84 (m, 1H), 3.26 (s, 3H), 2.82-2.76 (m, 1H), 2.57-2.53 (m, 2H), 2.16-2.10 (m, 2H) ppm; additional peaks due to the minor diastereomer were observed at 4.11-4.09 (m, 0.08H) and 2.28-2.23 (m, 0.18H) 13C NMR (101 MHz, $CDCl_3$) δ 174.4 (C), 136.1 (C), 128.7 (CH), 128.4 (CH), 128.3 (CH), 70.3 (CH), , 66.5 (CH_2), 55.3 (CH_3), 33.7 (CH_2), 33.0 (CH2, minor diastereomer), 29.2 (CH) ppm; additional peaks due to the minor diastereomer were observed at 73.2 (CH), 66.8 (CH_2), 33.0 (CH_2). HRMS (ESI+) $[M+H]^+$ Calculated for $C_{13}H_{17}O_3^+$ 221.1178; found 221.1168.

Benzyl 2-diazopent-4-enoate

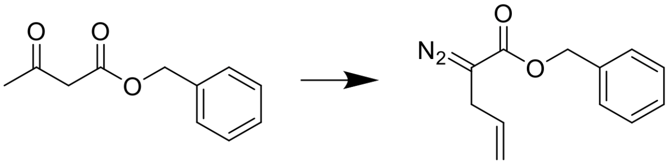

The procedure was adapted from the literature.[6] A flame-dried round bottom flask was charged with potassium *tert*-butoxide (1.95 g, 17.4 mmol, 1.20 equiv.) under an atmosphere of N_2. THF (50 mL) was added, and the solution was chilled in an ice bath. Benzyl acetoacetate (3.34 g, 17.4) was added, and then the solution was allowed to warm to room temperature and stir for 30 minutes. Allyl bromide (1.75 g, 14.4 mmol) was added and the reaction mixture was heated to reflux and allowed to stir overnight. The next day, the solution was allowed to cool to room temperature, then was diluted with ether and extracted with water. The aqueous phase was further extracted with ether (2x). The organic phase was pooled and dried with sodium sulfate, filtered, and concentrated under vacuum. The crude product was used directly in the next step.

To a flame-dried round bottom flask was added *p*-acetamidobenzenesulfonyl azide (4.17 g, 17.4 mmol) under N_2 atmosphere, followed by the addition of acetonitrile (60 mL). The crude ketoester product was dissolved in acetonitrile (10 mL) and added to the reaction mixture. 1,8-

diazabicyclo[5.4.0]undec-7-ene (3.30 g, 21.7 mmol) was added dropwise, and the reaction mixture was allowed to stir with cooling by an ice bath for 3 hours and then allowed to warm to room temperature overnight. The solution was diluted with ether and extracted with water. The aqueous phase was further extracted with ether (2x). The organic phase were combined and dried with sodium sulfate, filtered, and concentrated under vacuum, followed by chromatography on silica gel with 2% ethyl acetate in hexanes. The title compound was obtained as a clear yellow oil (908 mg, 4.20 mmol, 29% yield).

^{1}H NMR (400 MHz, CDCl3) δ 7.37-7.33 (m, 5H), 5.87-5.77 (m, 1H), 5.22 (s, 2H), 5.18-5.11 (m, 2H), 3.08-3.06 (m, 2H) ppm; ^{13}C NMR (101 MHz, CDCl3) δ 136.2 (C), 132.5 (CH), 128.7 (CH), 128.4 (CH), 128.2 (CH), 117.9 (CH_2), 66.6 (CH_2), 27.5 (CH_2) ppm; HRMS (ESI+) [M+H]+ Calculated for $C_{12}H_{13}O_2N_2^+$ 217.0977; found 217.0968.

Benzyl bicyclo[1.1.0]butane-1-carboxylate (9)

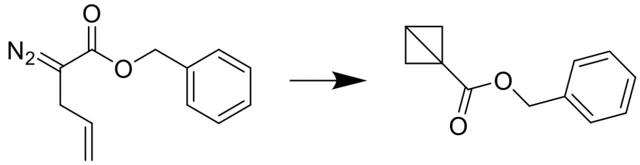

The procedure was adapted from the literature.[6] A flame-dried round bottom flask was charged with Rh_2(*S*-NTTL)$_4$ (4.0 mg, 0.0028 mmol) under N_2 atmosphere. Toluene (4 mL) was added and the solution was cooled in a dry ice/acetone bath at –78 °C. Benzyl 2-diazopent-4-enoate (106 mg, 0.490 mmol) was dissolved in toluene (4 mL) and added to the reaction mixture at a rate of 1 mL/h via syringe pump. After addition, the reaction mixture was allowed to warm to room temperature overnight and a color change from orange to a slight green tint occurred. The reaction mixture was concentrated, then chromatographed on silica gel. The title compound was obtained as a clear oil (70.3 mg, 0.373 mmol, 76% yield).

^{1}H NMR (600 MHz, $CDCl_3$) δ 7.35-7.33 (m, 5H), 5.15 (s, 2H), 2.40-2.39 (m, 2H), 2.12-2.09 (m, 1H), 1.18-1.17 (m, 2H) ppm; ^{13}C NMR (101 MHz, CDCl3) δ 173.2 (C), 136.4 (C), 128.7 (CH), 128.3 (CH), 128.1 (CH), 66.5 (CH_2), 35.8 (CH_2), 17.0 (CH), 9.3 (C) ppm; HRMS (ESI+) [M+H]+ Calculated for $C_{12}H_{13}O_2$+ 189.0916; found 189.0907.

Cyclobut-2-ene carboxylic acid

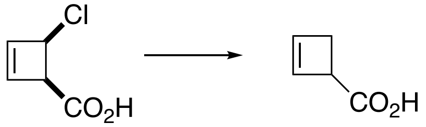

To a solution of *cis*-chlorocyclobutenecarboxylic acid[7] (331 mg, 2.50 mmol, 1.00 equiv.) in THF (0.15 M) were added $Pd(PPh_3)_4$ (144 mg, 0.125 mmol, 0.05 equiv.) and LiCl (318 mg, 7.50 mmol, 3.00 equiv.). The resulting mixture was degassed (freeze – pump – thaw, 3 cycles), before Bu_3SnH (0.807 mL, 3.00 mmol, 1.20 equiv.) was added. The reaction mixture was stirred at room temperature for 24 h, or until NMR analysis confirmed full consumption of the starting material. The reaction was terminated by the addition of 1M HCl and was subsequently diluted with EtOAc. The aqueous layer was extracted with EtOAc (3x) and the combined organic layers were dried over anhydrous Na_2SO_4. The solvents were evaporated (*nota bene: the temperature of the water bath must not exceed 30 °C, in order to prevent electrocyclic ring opening of the cyclobutene*) and the crude material was purified by flash column chromatography (SiO_2; 20% EtOAc + 3% AcOH in heptanes) to afford (*rac*)-cyclobutenecarboxylic acid (98.0 mg, 1.00 mmol, 40% yield) as a colorless powder. TLC: R_f (50% EtOAc in heptanes) = 0.35; $KMnO_4$ stain after heating. All analytical data with those reported in the literature.[7]

Benzyl cyclobut-2-ene carboxylate (7)

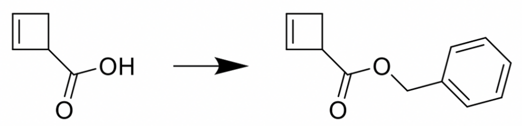

A flame-dried round bottom flask was charged with DMAP (5.4 mg, 0.044 mmol, 0.26 equiv.) and EDC (44.6 mg, 0.233 mmol) under N_2 atmosphere. Benzyl alcohol (20.9 mg, 0.193 mmol) was added, followed by cyclobut-2-ene carboxylic acid (16.4 mg, 0.167176 mmol, 1.00 equiv.) dissolved in methylene chloride (3 mL). The solution was allowed to stir at room temperature overnight. The next day, the reaction mixture was loaded directly on silica gel and chromatographed with an eluent of 40% ethyl acetate in hexanes. The title compound was obtained as a clear oil (27.7 mg, 0.147 mmol, 88% yield). ^{1}H NMR and ^{13}C NMR agreed with the spectra reported in the literature.[8]

N-benzyl-1,2-diazaspiro[2.3]hex-1-ene-5-carboxamide (27)

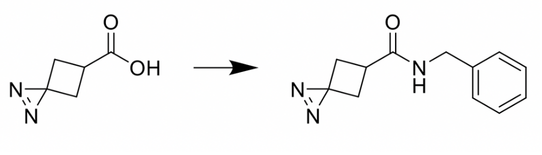

A flame-dried round bottom flask was charged with DMAP (23 mg, 0.19 mmol) and 1,2-diazaspiro[2.3]hex-1-ene-5-carboxylic acid (203 mg, 1.61 mmol) under N_2 atmosphere. Methylene chloride (5 mL) was added, followed by benzylamine (196 mg, 1.83 mmol, 1.14 equiv.). EDC (373 mg, 1.95 mmol, 1.21 equiv.) was added in one portion. The solution was allowed to stir at room temperature overnight. The next day, the reaction mixture was loaded directly on silica gel and chromatographed with an eluent of 50% ethyl acetate in hexanes. The title compound was obtained as a white flaky solid (285 mg, 1.32 mmol, 82% yield).

^{1}H NMR (400 MHz, CD_3OD) δ 7.32-7.22 (m, 5H), 4.37 (s, 2H), 3.23-3.18 (m, 1H), 2.53-2.48 (m, 2H), 2.35-2.29 (m, 2H) ppm; ^{13}C NMR (101 MHz, CD_3OD) δ 176.2 (C), 140.0 (C), 129.6 (CH), 128.6 (CH), 128.3 (CH), 44.3 (CH_2), 35.1 (CH_2), 32.1 (CH), 28.9 (C) ppm; HRMS (ESI+) $[M+H]^+$ Calculated for $C_{12}H_{14}ON_3^+$ 216.1137; found 216.1128.

***N*-benzylcyclobut-2-enecarboxamide (8)**

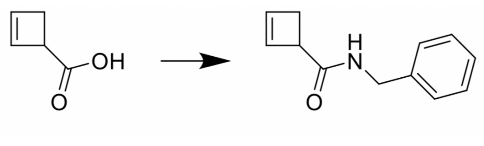

A flame-dried round bottom flask was charged with DMAP (3.9 mg, 0.032 mmol, 0.21 equiv.) and EDC (44.1 mg, 0.230 mmol, 1.50 equiv.) under N_2. Benzylamine (19.6 mg, 0.183 mmol) was added, followed by cyclobut-2-ene carboxylic acid (15.1 mg, 0.154 mmol, 1.00 equiv.) dissolved in methylene chloride (3 mL). The solution was allowed to stir at room temperature overnight. The next day, the reaction mixture was loaded directly on silica gel and chromatographed with an eluent of 40% ethyl acetate in hexanes. The title compound was obtained as a white solid (21.5 mg, 0.115 mmol, 75% yield).

^{1}H NMR (600 MHz, CD_3OD) δ 7.36-7.27 (m, 5H), 6.30 (m, 1H), 6.11-6.10 (m, 1H), 4.44-4.38 (m, 2H), 3.69-3.68 (m, 1H), 2.88-2.85 (m, 1H), 2.67-3.65 (m, 1H) ppm; ^{13}C NMR (150 MHz, CD_3OD) δ 175.7 (C), 140.4 (CH), 140.2 (C), 136.5 (CH), 129.5 (CH), 128.5 (CH), 128.2 (CH), 48.8 (CH), 44.0 (CH_2), 36.0 (CH_2) ppm; HRMS (ESI+) $[M+H]^+$ Calculated for $C_{12}H_{14}ON^+$ 188.1075; found 188.1072.

***N*-Benzyl-2-methylenecyclopropanecarboxamide (6)**

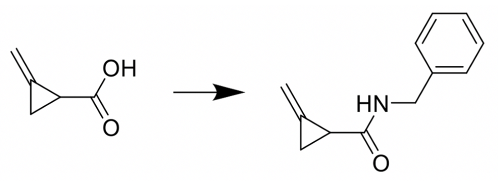

A flame-dried round bottom flask was charged with DMAP (39.3 mg, 0.322 mmol, 0.105 equiv.) and EDC (710. mg, 3.70 mmol) under N_2. Benzylamine (363 mg, 3.39 mmol) was added, followed by 2-methylenecyclopropane carboxylic acid (301 mg, 3.07 mmol) dissolved in methylene chloride (7 mL). The solution was allowed to stir at room temperature overnight. The next day, the reaction mixture was loaded directly on silica gel and chromatographed with an eluent of 50% ethyl acetate in hexanes. The title compound was obtained as a white solid (403 mg, 2.15 mmol, 70% yield).

^{1}H NMR (600 MHz, CD_3OD) δ 7.34-7.26 (m, 5H), 5.53-5.50 (m, 2H), 4.41 (s, 2H),

2.36-2.33 (m, 1H), 1.78-1.75 (m, 1H), 1.65-1.61 (m, 1H) ppm; 13C NMR (101 MHz, CD_3OD)δ 173.7 (C), 140.0 (C), 132.6 (C), 129.6 (CH), 128.6 (CH), 128.2 (CH), 104.5 (CH_2), 44.3 (CH_2), 20.4 (CH), 10.8 (CH_2) ppm; HRMS (ESI+) $[M+H]^+$ Calculated for $C_{12}H_{14}ON^+$ 188.1075; found 188.1066.

Ethyl 3-hydroxycyclobutane carboxylate, 13:1 dr

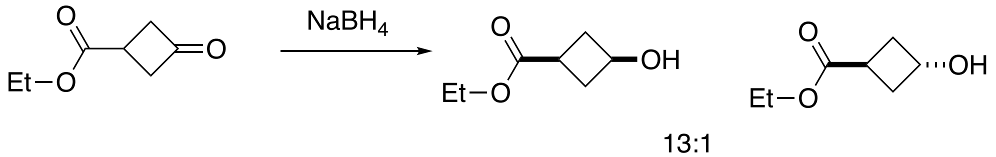

A flame-dried round bottom flask was charged with ethyl 3-oxocyclobutane carboxylate (1.75 g, 12.3 mmol), THF (32 mL), and methanol (1.6 mL) under N_2. The solution was chilled in an ice bath. Sodium borohydride (250 mg, 6.61 mmol) was added. The reaction was monitored by TLC and shown to be complete in 30 minutes. The reaction mixture was then diluted with ethyl acetate and extracted with aqueous sodium bicarbonate. The aqueous layer was extracted with ethyl acetate (2x). The organic layers were combined and extracted with brine, followed by drying with sodium sulfate. The solution was concentrated under vacuum and then chromatographed with an eluent of 50% ethyl acetate in hexanes. The title compound was obtained as a clear oil (1.72 g, 11.9 mmol, 97% yield) as a 13:1 mixture of diastereomers.

^{1}H NMR (400 MHz, $CDCl_3$) δ 4.21-4.11 (m, 3H), 2.61-2.59 (m, 3H), 2.18-2.15 (m, 2H), 1.26 (t, J = 7.6 Hz, 3H) ppm; ^{13}C NMR (101 MHz, CDCl3) δ 175.0 (C), 63.6 (CH), 60.8 (CH_2), 37.2 (CH_2), 29.2 (CH), 14.4 (CH_3) ppm; Peaks due to the minor diastereomer were observed at 65.9 (CH), 60.8 (CH_2), 36.2 (CH_2), 31.3 (CH) ppm. HRMS (ESI+) $[M+H]^+$ Calculated for $C_7H_{13}O_3^+$ 145.0865; found 145.0863.

Ethyl 3-methoxycyclobutane carboxylate

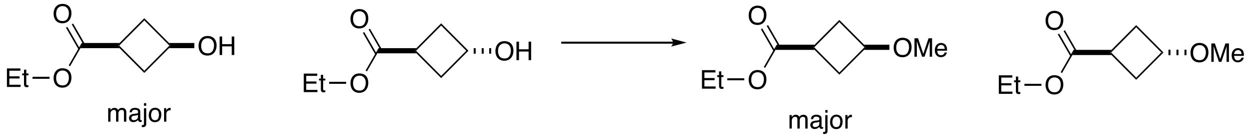

A flame-dried round bottom flask was charged with silver oxide (2.02 g, 8.72 mmol), ethyl 3-hydroxycyclobutane carboxylate (1.74 g, 12.1 mmol) and DMF (25 ml) under an atmosphere of N_2. Methyl iodide (3.42 g, 24.1 mmol) was added, then the mixture was heated to 45 °C. After an hour, additional silver oxide (2.05 g, 8.85 mmol, 0.733 equiv.) and methyl iodide (3.42 g, 24.1 mmol) was added. The mixture was allowed to stir overnight. The next day, the mixture was filtered, concentrated, then extracted with water and ethyl acetate. The organic layer was dried with sodium sulfate and filtered, followed by chromatography with an eluent of 0-30% ethyl acetate in hexanes. The title compound was obtained as a clear oil (649 mg, 4.10 mmol, 34% yield).

Peaks due to major diastereomer: ^{1}H NMR (600 MHz, CDCl3) δ 4.14 (q, J = 7.2 Hz, 2H), 3.81-3.76 (m, 1H), 3.23 (s, 3H), 2.63 2.59 (m, 1H), 2.51-2.48 (m, 2H), 2.20-2.15 (m, 2H), 1.25 (t, J = 7.1 Hz, 3H) ppm; ^{13}C NMR (150 MHz, $CDCl_3$) δ 174.6 (C), 70.4 (CH), 60.7 (CH2), 55.3 (CH3), 33.7 (CH_2, major diastereomer), 29.3 (CH), 14.4 (CH_3) ppm; HRMS (ESI+) [M+H]+ Calculated for C8H15O3+ 159.1021; found 159.1013.

3-Methoxycyclobutane carboxylic acid

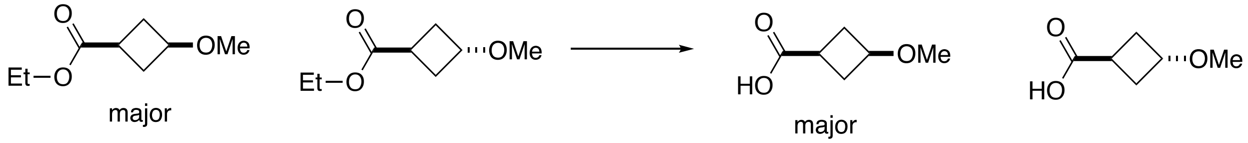

A flame-dried round bottom flask was charged with ethyl 3-methoxycyclobutane carboxylate (548 mg, 3.46 mmol). Sodium hydroxide (637 mg, 15.9 mmolwas added to 6 mL of a 1:1 solution of methanol and water. The basic solution was added to the round bottom flask containing ethyl 3-methoxycyclobutane carboxylate, followed by an additional 2 mL of methanol. The mixture was allowed to stir overnight. The next day, the reaction mixture was extracted with methylene chloride and the aqueous phase was collected, acidified to pH 1 with concentrated hydrochloric acid, and extracted with methylene chloride (3x). The combined

organics were dried with sodium sulfate, filtered, and concentrated to obtain the title compound as a clear oil (373 mg, 2.87 mmol, 83% yield).

^{1}H NMR (400 MHz, $CDCl_3$) δ 3.85-3.78 (m, 1H), 3.24 (s, 3H), 2.73-2.64 (m, 1H), 2.57-2.51 (m, 2H), 2.25-2.18 (m, 2H) ppm; ^{13}C NMR (101 MHz, $CDCl_3$) δ 180.4 (C), 70.2 (CH), 55.3 (CH_3), 33.5 (CH_2), 28.9 (CH) ppm; peaks due to minor diastereomer: 73.1 (CH), 55.5 (CH_3), 33.0 (CH_2), 31.6 (CH). HRMS (ESI$^+$) $[M+H]^+$ Calculated for $C_6H_{11}O_3^+$ 131.0708; found 131.0702.

N-Benzyl-3-methoxycyclobutane carboxamide (10)

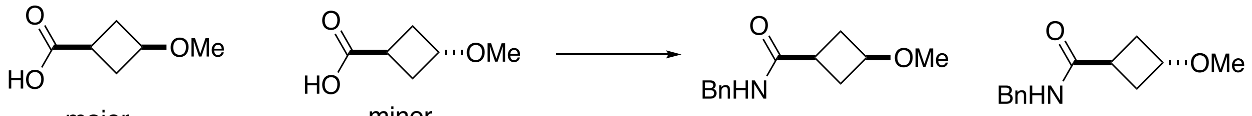

10 (major) **10** (minor)

A flame-dried round bottom flask was charged with DMAP (24.3 mg, 0.199 mmol) and EDC (387 mg, 2.02 mmol) under an atmosphere of N_2. Benzylamine (196 mg, 1.83 mmol) was added, followed by 3-methoxycyclobutane carboxylic acid (200 mg, 1.54 mmol) dissolved in methylene chloride (6 mL). The solution was allowed to stir at room temperature overnight. The next day, the reaction mixture was loaded directly on silica gel and chromatographed with an eluent of 30% ethyl acetate in hexanes. The title compound was obtained as a 20:1 mixture of diastereomers as a white solid (302 mg, 1.38 mmol, 89% yield).

^{1}H NMR (600 MHz, CD_3OD) δ 7.34-7.25 (m, 5H), 4.39 (s, 2H), 3.87-3.82 (m, 1H), 3.27 (s, 3H), 2.69-2.63 (m, 1H), 2.50-2.45 (m, 2H), 2.17-2.13 (m, 2H) ppm; peaks assigned to the minor diastereomer: 4.16-4.12 (m, 0.05H), 2.24-2.19 (m, 0.09H), ^{13}C NMR (101 MHz, $CDCl_3$) δ 173.7 (C), 138.4 (C), 128.9 (CH), 128.0 (CH), 127.7 (CH), 70.2 (CH), 55.2 (CH_3), 43.9 (CH_2), 33.6 (CH_2, major diastereomer), 33.2 (CH_2, minor diastereomer), 31.1 (CH) ppm; HRMS (ESI+) $[M+H]^+$ Calculated for $C_{13}H_{18}O_2N^+$ 220.1338; found 220.1329.

Benzyl 3-(3-methyl-3*H*-diazirin-3-yl)propanoate (31)

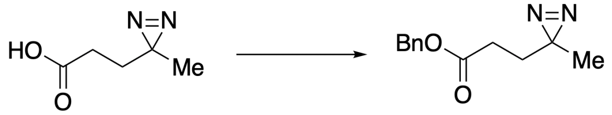

A flame-dried round bottom flask was charged with DMAP (33.8 mg, 0.264 mmol) and EDC (511 mg, 2.67 mmol) under N_2. Benzyl alcohol (261 mg, 2.41 mmol) was added, followed by 3-(3-methyl-3*H*-diazirin-3-yl)propanoic acid[9] (265 mg, 2.07 mmol) dissolved in methylene chloride (7 mL). The solution was allowed to stir at room temperature overnight. The reaction mixture was loaded directly on silica gel and chromatographed with an eluent of 10% ethyl acetate in hexanes. The title compound was obtained as a clear oil (383 mg, 1.75 mmol, 85% yield).

^{1}H NMR (400 MHz, $CDCl_3$) δ 7.39-7.35 (m, 5H), 5.12 (s, 2H), 2.24 (t, J = 7.2 Hz, 2H), 1.73 (t. J = 8.0, 2H), 1.02 (s, 3H) ppm; ^{13}C NMR (101 MHz, $CDCl_3$) δ 172.3 (C), 135.8 (C), 128.7 (CH), 128.5 (CH), 128.5 (CH), 66.7 (CH_2), 29.8 (CH_2), 28.9 (CH_2), 25.3 (C), 19.8 (CH_3) ppm; HRMS (ESI+) [M+H]+ Calculated for $C_{12}H_{15}O_2N_2$+ 219.1134; found 219.1135.

(*Z*)-Benzyl pent-3-enoate

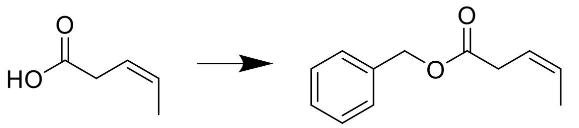

A flame-dried round bottom flask was charged with DMAP (26.9 mg, 0.220 mmol) and EDC (443 mg, 2.31 mmol) under N_2 atmosphere. Benzyl alcohol (219 mg, 2.03 mmol) was added, followed by (*Z*)- pent-3-enoic acid [10, 11] (173 mg, 1.73 mmol) dissolved in methylene chloride (5 mL). The solution was allowed to stir at room temperature overnight. The reaction mixture was loaded directly on silica gel and chromatographed with an eluent of 10% ethyl acetate in hexanes. The title compound was obtained as a clear oil (200 mg, 1.052 mmol, 61% yield).

^{1}H NMR (600 MHz, CD3OD) δ 7.38-7.35 (m, 5H), 5.71-5.66 (m, 1H), 5.61-5.56 (m, 1H), 5.15 (s, 2H), 3.19 (d, *J* = 7.4 Hz, 2H), 1.67 (d, *J* = 7.4 Hz, 3H) ppm; ^{13}C NMR (101 MHz, $CDCl_3$) δ

172.0 (C), 136.1 (C), 128.7 (CH), 128.4 (CH), 128.3 (CH), 127.9 (CH), 121.7 (CH), 66.6 (CH_2), 32.8 (CH_2), 13.1 (CH_3) ppm; HRMS (ESI^+) $[M+H]^+$ Calculated for $C_{12}H_{15}O_2^+$ 191.1072; found 191.1071.

(*E*)-Benzyl pent-3-enoate

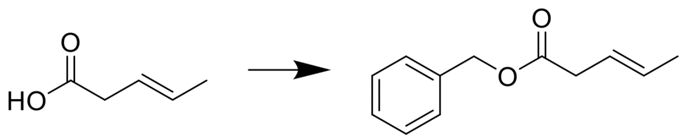

A flame-dried round bottom flask was charged with DMAP (26.1 mg, 0.214 mmol) and EDC (515 mg, 2.68 mmol) under an atmosphere of N_2. Benzyl alcohol (261 mg, 2.41 mmol, 1.19 equiv.) was added, followed by (*E*)- pent-3-enoic acid[12] (204 mg, 2.037 mmol) dissolved in methylene chloride (5 mL). The solution was allowed to stir at room temperature overnight. The next day, the reaction mixture was loaded directly on a silica gel and chromatographed with an eluent of 10% ethyl acetate in hexanes. The title compound was obtained as a clear oil (282 mg, 1.48 mmol, 73% yield).

^{1}H NMR (600 MHz, CD_3OD) δ 7.38-7.34 (m, 5H), 5.62-5.58 (m, 2H), 5.15 (s, 2H), 3.11 (d, J = 7.7 Hz, 2H), 1.72 (d, J = 6.4 Hz, 2H) ppm; ^{13}C NMR (101 MHz, $CDCl_3$) δ 172.2 (C), 136.1 (C), 129.8 (CH), 128.7 (CH), 128.4 (CH), 122.7 (CH), 66.5 (CH_2), 38.2 (CH_2), 18.1 (CH_3) ppm; HRMS (ESI^+) $[M+H]^+$ Calculated for $C_{12}H_{15}O_2^+$ 191.1072; found 191.1071.

Benzyl 4-methoxypentanoate

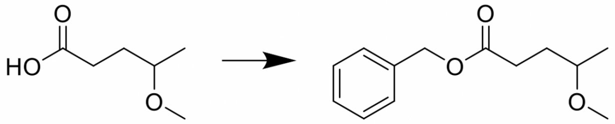

A flame-dried round bottom flask was charged with DMAP (32.5 mg, 0.266 mmol) and EDC (515.7 mg, 2.690 mmol) under an atmosphere of N_2. Benzyl alcohol (261 mg, 2.41 mmol) was added, followed by 4-methoxypentanoic acid[12] (269 mg, 2.04 mmol) dissolved in methylene chloride (5 mL). The solution was allowed to stir at room temperature overnight. The reaction

mixture was loaded directly on silica gel and chromatographed with an eluent of 10% ethyl acetate in hexanes. The title compound was obtained as a clear oil (409 mg, 1.84 mmol, 90% yield).

^{1}H NMR (600 MHz, CD_3OD) δ 7.40-7.35 (m, 5H), 5.15 (s, 2H), 3.39-3.34 (m, 4H), 2.46 (t, J = 8.5 Hz, 2H), 1.82-1.78 (m, 2H), 1.16 (d, J = 6.3 Hz, 3H) ppm; ^{13}C NMR (101 MHz, $CDCl_3$) δ 173.8 (C), 136.2 (C), 128.7 (CH), 128.3 (CH), 128.3 (CH), 75.9 (CH), 66.3 (CH_2), 56.3 (CH_3), 31.5 (CH_2), 30.4 (CH_2), 19.0 (CH_3) ppm; HRMS (ESI$^+$) $[M+H]^+$ Calculated for $C_{13}H_{19}O_3^+$ 223.1334; found 223.1336.

Benzyl pent-4-enoate

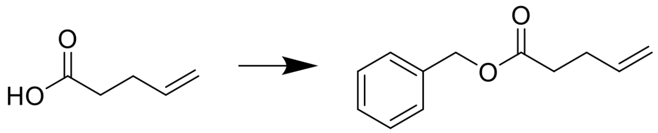

A flame-dried round bottom flask was charged with DMAP (27.1 mg, 0.222 mmol) and EDC (519 mg, 2.71 mmol) under an atmosphere of N_2. Benzyl alcohol (261 mg, 2.41 mmol) was added, followed by pent-4-enoic acid (206 mg, 2.06 mmol) dissolved in methylene chloride (5 mL). The solution was allowed to stir at room temperature overnight. The reaction mixture was loaded directly on silica gel and chromatographed with an eluent of 10% ethyl acetate in hexanes. The title compound was obtained as a clear oil (383 mg, 2.01 mmol, 98% yield).

1H NMR (600 MHz, CD_3OD) δ 7.39-7.35 (m, 5H), 5.89-5.83 (m, 1H), 5.15 (s, 2H), 5.09-5.06 (m, 1H), 5.02-4.99 (m, 1H), 2.51-2.49 (m, 2H), 2.40-2.39 (m, 2H) ppm; ^{13}C NMR (101 MHz, $CDCl_3$) δ 173.1 (C), 13cyclo.7 (CH), 136.1 (C), 128.7 (CH), 128.4 (CH), 115.7 (CH_2), 66.4 (CH_2), 33.7 (CH_2), 29.0 (CH_2) ppm; HRMS (ESI+) [M+H]+ Calculated for $C_{12}H_{15}O_2^+$ 191.1072; found 191.1073.

Methyl 3,3-dimethoxycyclobutane-1-carboxylate (17)

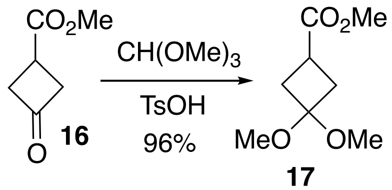

To a mixture of methyl 3-oxocyclobutane-1-carboxylate (10.00 g, 78.05 mmol) and trimethoxymethane (41.4 g, 390 mmol) in CH_2Cl_2 (100 mL)/MeOH (100 mL) was added TsOH (1.34 g, 7.80 mmol) at 0 °C, then the mixture was stirred at 25 °C for 20 hours. TLC showed starting material was consumed and a new spot was formed (Chromogenic agent: Iodine). The solvent was removed. Then the mixture was diluted with water (200 mL) and extracted with EtOAc (200 mL x 2). The combined organic layers were washed with brine (100 mL x 2), dried over anhydrous Na_2SO_4, filtered, and concentrated in vacuo. The residue was purified by flash column chromatography (Combi-Flash, 120 g silica gel, 0~8% EtOAc in Petroleum ether) to afford the title compound (13 g, 95.6%) as a colorless oil.

NMR data matched those in the literature[13]: ^{1}H NMR (400 MHz, $CDCl_3$) δ 3.70 (s, 3H), 3.17 (s, 3H), 3.15 (s, 3H), 2.93-2.85 (m, 1H), 2.47 - 2.36 (m, 4H).; ^{13}C NMR (100 MHz, $CDCl_3$) δ 175.21, 99.72, 51.87, 48.66, 48.39, 35.45, 28.57.

Methyl 3,3-dimethoxy-1-(3-(trimethylsilyl)prop-2-yn-1-yl)cyclobutane-1-carboxylate

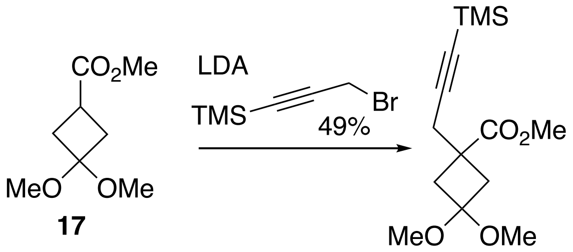

A mixture of methyl 3,3-dimethoxycyclobutane-1-carboxylate (13 g, 75 mmol) in dry THF (550 mL) was cooled to –78 °C under N_2 atmosphere, then LDA (48.5 mL, 97.0 mmol, 2 M in THF) was added dropwise at –78 °C over 15 mins. Afterwards, the reaction mixture was stirred at –78 °C for 30 mins. 3-Bromoprop-1-yn-1-yl)trimethylsilane (17.1 g, 89.6 mmol) in THF (100 mL) was slowly added dropwise. After the addition was completed, the reaction mixture was allowed to gradually warm to 0 °C and stirred for 4 hours. The mixture was quenched with sat. aq. NH_4Cl (300 mL) and then the mixture was extracted by ethyl acetate (200 mL). The organic layer was washed with brine (50 mL x 2) and dried over anhydrous Na_2SO_4, filtered, and concentrated in vacuo. The residue was purified by flash column chromatography (Combi-Flash, 120 g silica gel, 0~8% EtOAc in Petroleum ether) to afford the title compound (10.5 g, 49%) as a yellow oil.

^{1}H NMR (400 MHz, $CDCl_3$) δ 3.69 (s, 3H), 3.11 (s, 3H), 3.10 (s, 3H), 2.65 (s, 2H), 2.56 (d, J = 13.0 Hz, 2H), 2.20 (d, J = 13.1 Hz, 2H), 0.10 (s, 9H).; ^{13}C NMR (100 MHz, $CDCl_3$) δ 175.56, 102.97, 98.81, 86.96, 52.45, 48.68, 39.84, 38.68, 28.64, 0.20.

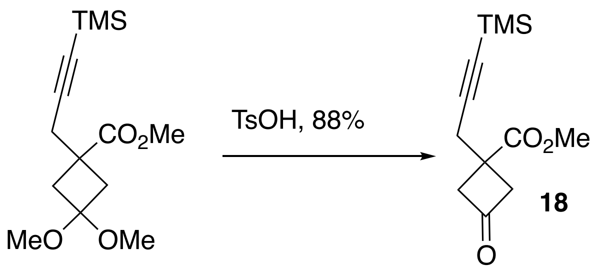

18

Methyl 3-oxo-1-(3-(trimethylsilyl)prop-2-yn-1-yl)cyclobutane-1-carboxylate (18)

To a mixture of methyl 3,3-dimethoxy-1-(3-(trimethylsilyl)prop-2-yn-1-yl)cyclobutane-1-carboxylate (10.5 g, 36.917 mmol) in acetone/H_2O (100 mL/50 mL), TsOH (1.27 g, 7.38 mmol) was added at room temperature, then the reaction mixture was stirred at 60 °C for 20 hours. TLC showed starting material was consumed and a new spot was formed. The mixture was quenched with water (100 mL) and extracted with MTBE (100 mL x 2). The combined organic layers were washed with brine (50 mL) and dried over anhydrous Na_2SO_4, filtered, and concentrated in vacuo. The residue was purified by flash column chromatography (Combi-Flash, 120 g silica gel, 0~8% EtOAc in Petroleum ether) to afford **18** (7.7 g, 87.5%) as a yellow oil.

^{1}H NMR (400 MHz, $CDCl_3$) δ 3.76 (s, 3H), 3.49 – 3.42 (m, 2H), 3.29 – 3.23 (m, 2H), 2.84 (s, 2H), 0.10 (s, 9H); ^{13}C NMR (100 MHz, $CDCl_3$) δ 203.01, 174.80, 101.50, 88.57, 55.51, 52.97, 36.73, 26.95, 0.03; HRMS (ESI+) [M+H]+ calculated for $C_{12}H_{18}O_3Si$ $[M + H]^+$ 239.1098, found 239.1098.

Methyl 3-(hydroxyimino)-1-(3-(trimethylsilyl)prop-2-yn-1-yl)cyclobutane-1-carboxylate (19)

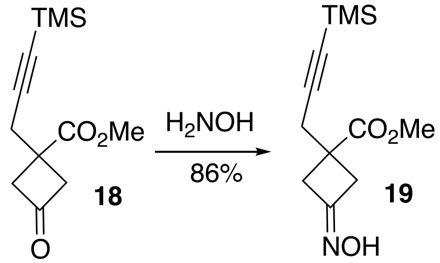

A mixture of methyl 3-oxo-1-(3-(trimethylsilyl)prop-2-yn-1-yl)cyclobutane-1-carboxylate (7.7 g, 32.30 mmol) and NH_2OH•HCl (2.47 g, 35.5 mmol) in pyridine (4 mL) and EtOH (80 mL) was stirred at 85 °C for 5 hours. TLC showed starting material was consumed and a new spot was formed. The mixture was quenched with water (100 mL) and the EtOH was removed in vacuo. Then the mixture was extracted with MTBE (100 mL x 2), and the combined organic layers were washed with 1 M HCl (30 mL) and brine (40 mL x 2), dried over anhydrous Na_2SO, filtered, and concentrated to afford the title compound (7.0 g, 86%) as a yellow oil.

^{1}H NMR (400 MHz, d^6-DMSO) δ 10.50 (s, 1H), 3.67 (s, 3H), 3.19 – 3.09 (m, 2H), 2.87 – 2.83 (m, 2H), 2.72 (s, 2H), 0.10 (s, 9H).; ^{13}C NMR (100 MHz, d^6-DMSO) δ 173.61, 148.74, 102.89, 85.97, 51.85, 39.89, 26.51, 0.51.; HRMS (ESI+) [M+H]+ calculated for $C_{12}H_{19}NO_3Si$ 254.1207, found 254.1209.

Methyl 3-(((methylsulfonyl)oxy)imino)-1-(3-(trimethylsilyl)prop-2-yn-1-yl)cyclobutane-1-carboxylate (20)

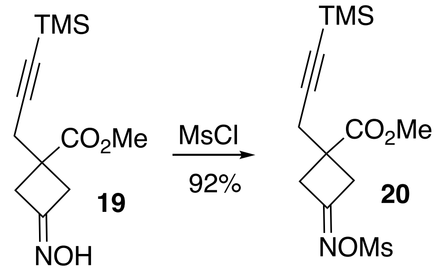

A mixture of methyl 3-(hydroxyimino)-1-(3-(trimethylsilyl)prop-2-yn-1-yl)cyclobutane-1-carboxylate (5.00 g, 19.7 mmol) and triethylamine (3.99 g, 39.5 mmol) in CH_2Cl_2 (120 mL) was cooled in a bath at 0 °C. MsCl (3.53 g, 30.8 mmol) in CH_2Cl_2 (20 mL) was slowly added dropwise with continued cooling at 0 °C. The reaction mixture was stirred at 0 °C for 4 hours.

TLC showed starting material was consumed and a new spot was formed. The mixture was quenched with water (120 mL) and extracted with CH_2Cl_2 (100 mL x 2). The combined organic layers were washed with brine (50 mL) and dried over anhydrous Na_2SO_4, filtered, and concentrated in vacuo to afford **20** (6.0 g, 92%) as a yellow oil, which was used directly without any further purification.

^{1}H NMR (400 MHz, $CDCl_3$) δ 3.77 (s, 3H), 3.46-3.41 (m,2H), 3.26 – 3.18 (m, 2H), 3.13 (s, 3H), 2.76 (s, 2H), 0.14 (s, 9H).; ^{13}C NMR (100 MHz, $CDCl_3$) δ 173.69, 164.39, 100.55, 88.80, 53.05, 40.41, 39.93, 39.72, 36.63, 27.21, 0.01.; HRMS (ESI+) [M+H]+ calculated for $C_{13}H_{21}NO_5SSi$ 332.0982, found 332.0982.

Methyl 5-(3-(trimethylsilyl)prop-2-yn-1-yl)-1,2-diazaspiro[2.3]hexane-5-carboxylate (21)

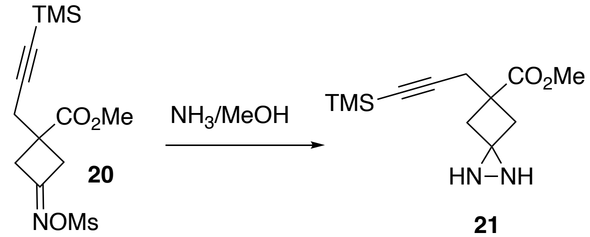

A solution of NH_3/MeOH (56 mL, 400 mmol, 7 M in MeOH) was added dropwise to a solution of methyl 3-(((methylsulfonyl)oxy)imino)-1-(3-(trimethylsilyl)prop-2-yn-1-yl)cyclobutane-1-carboxylate (6.00 g, 18.1 mmol) in CH_2Cl_2 (150 mL) at –78°C. The reaction mixture was stirred at –78 °C for 2 hours. The reaction temperature was allowed to warm to 25 °C and the mixture was stirred for 16 hours. TLC showed starting material was consumed and a new spot was formed. The reaction mixture was concentrated to afford the title compound (4 g, crude) as a yellow gum, which was used directly without further purification. ESI-MS: $[M + H]^+$ 253.2.

***M*ethyl 5-(3-(trimethylsilyl)prop-2-yn-1-yl)-1,2-diazaspiro[2.3]hex-1-ene-5-carboxylate (22)**

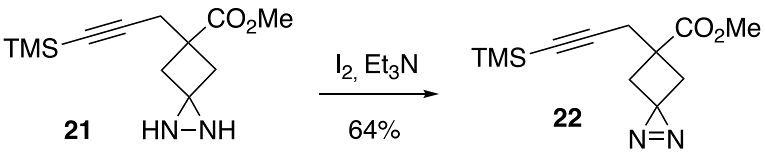

To a solution of methyl 5-(3-(trimethylsilyl)prop-2-yn-1-yl)-1,2-diazaspiro[2.3]hexane-5-carboxylate (4 g, crude) in CH_2Cl_2 (150 mL) was added triethylamine (2.40 g, 23.8 mmol) and I_2 (4.02 g, 15.8 mmol) at 0 °C, then the mixture was stirred at 25 °C for 2 hours. TLC showed starting material was consumed and a new spot was formed. The reaction mixture was quenched with aq. Na_2SO_3 (50 mL) and H_2O (60 mL), then extracted with CH_2Cl_2 (100 mL x 2). The combined organic layers were washed with brine, dried over anhydrous Na_2SO_4, filtered and concentrated in vacuo. The residue was purified by flash column chromatography (Combi-Flash, 120 g silica gel, 0~4% EtOAc in Petroleum ether) to afford **21** (2.5 g, 64% after 2 steps) as a yellow oil.

^{1}H NMR (400 MHz, $CDCl_3$) δ 3.79 (s, 3H), 2.78 (s, 2H), 2.68 (d, J = 14.9 Hz, 2H), 2.34 (d, J = 14.9 Hz, 2H), 0.20 (s, 9H).**;** ^{13}C NMR (100 MHz, $CDCl_3$) δ 174.90, 102.10, 87.78, 52.82, 39.94, 39.02, 28.06, 26.44, 0.21.**;** HRMS (ESI+) [M+H]+ calculated for $C_{12}H_{18}N_2O_2Si$ 251.1210, found 251.1212.

Methyl 5-(prop-2-yn-1-yl)-1,2-diazaspiro[2.3]hex-1-ene-5-carboxylate (23)

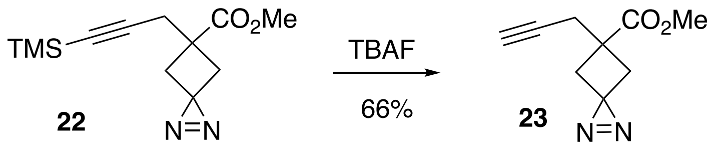

To a solution of methyl 5-(3-(trimethylsilyl)prop-2-yn-1-yl)-1,2-diazaspiro[2.3]hex-1-ene-5-carboxylate (1.50 g, 5.99 mmol) in THF (50 mL) was added TBAF (5.99 mL, 5.99 mmol, 1 M in THF) at 0 °C, then the mixture was stirred at 25 °C for 3 hours. TLC showed starting material was consumed and a new spot was formed. The reaction mixture was quenched with water (50 mL) and extracted with EtOAc (40 mL x 2). The combined organic layers were washed with water (20 mL x 2) and brine (20 mL x 2), and dried over anhydrous Na_2SO_4, filtered, and concentrated in vacuo. The residue was purified by flash column chromatography (Combi-Flash, 25 g silica gel, 0~4% EtOAc in Petroleum ether) to afford **27** (700 mg, 66%) as a light-yellow oil.

^{1}H NMR (400 MHz, $CDCl_3$) δ 3.81 (s, 3H), 2.79 (d, J = 2.6 Hz, 2H), 2.73 – 2.69 (m, 2H), 2.32 – 2.28 (m, 2H), 2.10 (t, J = 2.5 Hz, 1H).; ^{13}C NMR (100 MHz, $CDCl_3$) δ 174.47, 79.67, 70.64,

52.71, 39.81, 39.00, 26.73, 26.32.; HRMS (ESI+) [M+H]+ calculated for $C_9H_{10}N_2O_2$ 179.0815, found 179.0816.

5-(Prop-2-yn-1-yl)-1,2-diazaspiro[2.3]hex-1-ene-5-carboxylic acid (14)

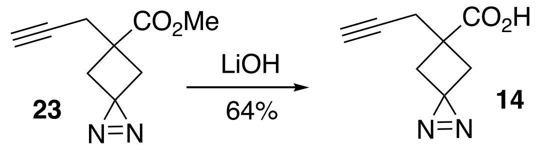

To a solution of methyl 5-(prop-2-yn-1-yl)-1,2-diazaspiro[2.3]hex-1-ene-5-carboxylate (630 mg, 3.54 mmol) in MeOH (15 mL) and H_2O (5 mL) was added LiOH (170 mg, 7.07 mmol) at 0 °C. Then the reaction mixture was stirred at 25 °C for 2 hours. TLC showed starting material was consumed and a new spot was formed. The reaction mixture was quenched with 0.5 M HCl (15 mL) and extracted with CH_2Cl_2 (15 mL x 2). The combined organic layers were washed with brine, dried over anhydrous Na_2SO_4, filtered, and concentrated in vacuo to afford **23** (370 mg, 64%) as a light yellow solid.

^{1}H NMR (400 MHz, $CDCl_3$) δ 2.84 (d, J = 2.5 Hz, 2H), 2.78 (d, J = 15.2 Hz, 2H), 2.38 (d, J = 15.3 Hz, 2H), 2.15 (t, J = 2.5 Hz, 1H); ^{13}C NMR (100 MHz, $CDCl_3$) δ 180.16, 79.39, 70.91, 39.74, 38.98, 26.33, 26.20.; HRMS (ESI+) [M+H]+ calculated for $C_8H_8N_2O_2$ 165.0659, found 165.0660.

tert-butyl prop-2-yn-1-yl(1,2-diazaspiro[2.3]hex-1-en-5-yl)carbamate (25)

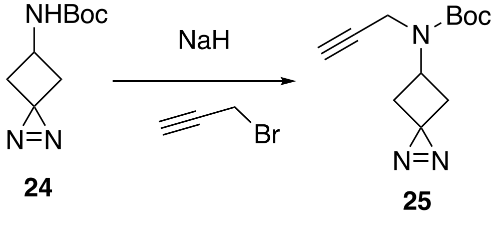

To a solution of tert-butyl (1,2-diazaspiro[2.3]hex-1-en-5-yl)carbamate (600 mg, 3.04 mmol) in THF (20 mL) was added NaH (150 mg, 3.8 mmol) at 25 °C in portions. After the addition was completed, the reaction mixture was stirred at 25 °C for 30 mins and then (3-bromoprop-1-yn-1-yl)trimethylsilane (700 mg, 3.66 mmol) and 18-crown-6 (50 mg, 0.19 mmol) were added. The reaction mixture was stirred at 40 °C for 2 hours. LC-MS showed the starting material was consumed and a peak with the desired mass was formed. The reaction was

quenched with sat. aq. NH_4Cl (50 mL) and extracted with ethyl acetate (25 mL x 2). The combined organic layers were washed with brine (25 mL x 2), and dried over anhydrous Na_2SO_4, filtered, and concentrated in vacuo. The residue was purified by flash column chromatography (Combi-Flash, 40 g silica gel, 0~10% EtOAc in Petroleum ether) to afford the title compound (650 mg, 90.8%) as a colorless oil.

^{1}H NMR (400 MHz, $CDCl_3$) δ 4.55 (br, 1H), 4.13 (d, J = 2.3 Hz, 2H), 2.73 – 2.67 (m, 2H), 2.40 – 2.34 (m, 2H), 2.23 (t, J = 2.4 Hz, 1H), 1.51 (s, 9H).; ^{13}C NMR (100 MHz, $CDCl_3$) δ 155.05, 81.28, 80.43, 71.59, 44.63, 37.40, 34.54, 28.61, 27.13.; HRMS (ESI+) [M+H]+ calculated for $C_{12}H_{17}N_3O_2$ 236.1394, find 236.1398.

***N*-(prop-2-yn-1-yl)-1,2-diazaspiro[2.3]hex-1-en-5-amine (12)**

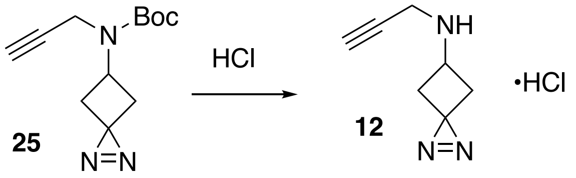

To a solution of tert-butyl prop-2-yn-1-yl(1,2-diazaspiro[2.3]hex-1-en-5-yl)carbamate (650 mg, 2.76 mmol) in CH_2Cl_2 (10 mL) was added HCl (6 mL, 4 M in dioxane, 24 mmol) at 0 °C. The mixture was stirred at 25 °C for 5 hours. LC-MS showed starting material was consumed and a peak with the desired mass was formed. The reaction mixture was concentrated to afford **17** (455 mg, 96%) as a white solid.

^{1}H NMR (400 MHz, d^6-DMSO) δ 10.34 (s, 2H), 4.02 – 3.95 (m, 1H), 3.89 (d, J = 2.5 Hz, 2H), 3.70 (t, J = 2.5 Hz, 1H), 2.68 (dd, J = 15.9, 5.8 Hz, 2H), 2.50 – 2.40 (m, 2H).; ^{13}C NMR (100 MHz, d^6-DMSO) δ 79.32, 74.86, 43.88, 34.48, 33.95, 27.01.; HRMS (ESI+) [M+H]+ calculated for $C_7H_9N_3$ 136.0869, found 136.0873.

General procedure for coupling reactions of 23

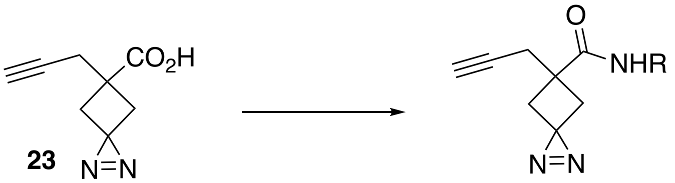

A 1-dram vial containing a stirbar was charged successively with carboxylic acid **23** (0.15 mmol), amine (0.165 mmol), and methylene chloride (1.5 mL, 0.10 M), followed by HATU (0.165 mmol). Diisopropylethylamine (DIPEA, 80 uL, 0.46 mmol) was added to the stirring mixture. The mixture was stirred at room temperature for 4 hours with monitoring via TLC (20-50% ethyl acetate in heptanes). Following completion of the reaction, the mixture was partitioned between CH_2Cl_2 (20 mL) and saturated aqueous NH_4Cl (10 mL). The organic extract was retained, then washed with brine (10 mL). The organic layer was dried over Na_2SO_4, then filtered and concentrated to afford the crude compounds which were purified by chromatography.

N-(2-oxo-2H-chromen-6-yl)-5-(prop-2-yn-1-yl)-1,2-diazaspiro[2.3]hex-1-ene-5-carboxamide (35)

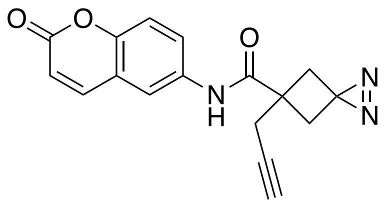

The general procedure was followed to provide 23.5 mg, solid, 51% yield; ^{1}H NMR (400 MHz, CD_3CN) δ 8.37 (s, 1H), 7.98 (d, J = 2.6 Hz, 1H), 7.85 (d, J = 9.6 Hz, 1H), 7.68 (dd, J = 8.9, 2.6 Hz, 1H), 7.30 (d, J = 8.9 Hz, 1H), 6.40 (d, J = 9.6 Hz, 1H), 2.94 (d, J = 2.7 Hz, 2H), 2.87 – 2.79 (m, 2H), 2.39 (t, J = 2.6 Hz, 1H), 2.33 – 2.25 (m, 2H); ^{13}C NMR (101 MHz, CD3CN) δ 173.32, 161.42, 151.45, 144.73, 136.08, 125.13, 120.07, 119.91, 117.80, 117.73, 80.81, 72.35, 42.57, 39.56, 28.64, 27.27; HRMS (ESI+) [M+H]+ calculated for $C_{17}H_{13}N_3O_3$ 308.1030, find 308.1025.

N-(2-oxo-2,3,4,5-tetrahydro-1H-benzo[b]azepin-8-yl)-5-(prop-2-yn-1-yl)-1,2-diazaspiro[2.3]hex-1-ene-5-carboxamide (37)

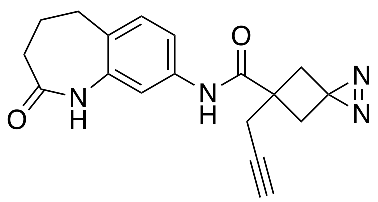

The general procedure was followed to provide 27.1 mg, solid, 56% yield; ^{1}H NMR (400 MHz, CD_3CN) δ 8.21 (s, 1H), 7.76 (s, 1H), 7.43 (d, J = 2.1 Hz, 1H), 7.26 (dd, J = 8.2, 2.2 Hz, 1H), 7.19 (d, J = 8.2 Hz, 1H), 2.92 (d, J = 2.6 Hz, 2H), 2.83 – 2.78 (m, 2H), 2.74 (t, J = 7.1 Hz, 2H), 2.38 (t, J = 2.6 Hz, 1H), 2.31 – 2.21 (m, 4H), 2.15 (pd, J = 7.1, 1.4 Hz, 2H); ^{13}C NMR (101 MHz, CD_3CN) δ 174.74, 173.09, 139.86, 138.89, 130.97, 130.85, 117.59, 114.36, 80.88, 72.29, 42.60, 39.56, 33.72, 30.48, 29.11, 28.65, 27.28; HRMS (ESI+) [M+H]+ calculated for $C_{18}H_{18}N_4O_2$ 323.1503, find 323.1507.

(2-benzylpiperidin-1-yl)(5-(prop-2-yn-1-yl)-1,2-diazaspiro[2.3]hex-1-en-5-yl)methanone (PF-91)

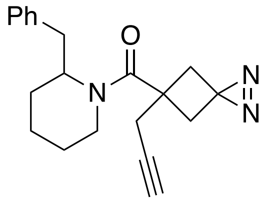

The general procedure was followed to provide 37.1 mg of an oil, 77% yield; ^{1}H NMR (400 MHz, CD_3CN) δ 7.33 – 7.21 (m, 5H), 4.97 (s, 1H), 3.34 – 3.10 (m, 2H), 3.02–2.82 (m, 2H), 2.70 – 2.50 (m, 4H), 2.35 (t, J = 2.7 Hz, 1H), 2.20 – 2.09 (m, 2H), 1.90–1.75 (m, 1H), 1.71 – 1.55 (m, 4H), 1.48 – 1.40 (m, 1H); ^{13}C NMR (101 MHz, CD_3CN) δ 172.43, 140.17, 130.25, 129.19, 127.15, 80.63, 72.14, 50.98, 42.76, 41.09, 40.24, 39.75, 36.30, 27.91, 27.35, 26.69, 19.58; HRMS (ESI+) [M+H]+ calculated for $C_{20}H_{23}N_3O$ 322.1914, found 322.1915.

(4-benzhydrylpiperazin-1-yl)(5-(prop-2-yn-1-yl)-1,2-diazaspiro[2.3]hex-1-en-5-yl)methanone (44)

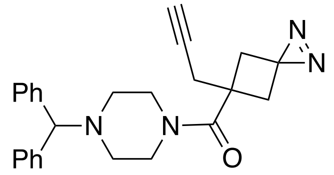

The general procedure was followed to provide 54.2 mg of a solid, 91% yield; ^{1}H NMR (400 MHz, CD_3CN) δ 7.51 – 7.42 (m, 4H), 7.35–7.25 (m, 4H), 7.25 – 7.16 (m, 2H), 4.30 (s, 1H), 3.45 (s, 4H), 2.85 – 2.76 (m, 2H), 2.73 (d, J = 2.7 Hz, 2H), 2.42 – 2.30 (m, 5H), 2.22 – 2.17 (m, 2H); ^{13}C NMR (101 MHz, CD_3CN) δ 172.02, 143.93, 129.64, 129.60, 128.66, 128.10, 80.60, 76.53, 72.14, 52.58, 47.11 (rotamer), 43.30 (rotamer), 40.97, 40.09, 28.27, 27.41; HRMS (ESI+) [M+H]+ calculated for $C_{25}H_{26}N_4O$ 399.2179, find 399.2182.

1-benzhydryl-*N*-(prop-2-yn-1-yl)-*N*-(1,2-diazaspiro[2.3]hex-1-en-5-yl)azetidine-3-carboxamide (41)

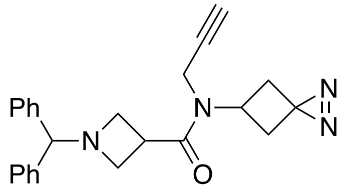

A 1-dram vial containing a stirbar was charged successively with carboxylic acid (40.1 mg, 0.15 mmol), amine (28.3 mg, 0.165 mmol), and dichloromethane (1.5 mL, 0.10 M), followed by HATU (62.7 mg, 0.165 mmol). DIPEA (80 uL, 0.46 mmol) was added to the stirring mixture. The reaction stirred at room temperature for 4 hours. The reaction was then partitioned between CH_2Cl_2 (20 mL) and saturated aqueous NH_4Cl (10 mL). The organic layer was extracted, separated, then washed with brine (10 mL). The organic phase was collected, then dried over Na_2SO_4. Na_2SO_4 was filtered and the filtrate was concentrated unto SiO_2 (5g). The dry loaded crude material was purified by FCC (4g SiO2, 35%-->50% EtOAc in heptanes gradient) to afford the title compound (41 mg, 71%) as a white solid.

^{1}H NMR (400 MHz, CD_3CN) δ 7.48 – 7.40 (m, 4H), 7.33 – 7.24 (m, 4H), 7.23 – 7.17 (m, 2H), 4.62 (m, 1H), 4.42 (s, 1H), 4.31 (s, 1H), 4.07 (dq, J = 11.5, 6.0, 5.0 Hz, 1H), 3.50 (s, 1H), 3.45 – 3.38 (m, 2H), 3.20 (s, 2H), 2.67 (d, J = 9.2 Hz, 2H), 2.52 (s, 1H), 2.40 – 2.30 (m, 2H); ^{13}C NMR

(101 MHz, CD_3CN) δ ^{13}C NMR (101 MHz, CD_3CN) δ 173.38, 172.62, 143.51, 129.49, 128.30, 128.14, 82.32, 80.98, 78.32, 78.27, 73.79, 72.00, 56.28, 56.24, 45.28, 44.94, 37.45, 36.99, 35.45, 33.80, 33.36, 31.55, 28.34, 27.55; HRMS (ESI+) [M+H]+ calculated for $C_{24}H_{24}N_4O$ 385.2023, find 385.2022.

(R)-N-Benzyl-N-(1-phenylethyl)-5-(prop-2-yn-1-yl)-1,2-diazaspiro[2.3]hex-1-ene-5-carboxamide (45)

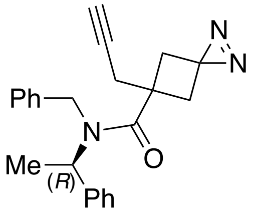

A 1-dram vial was charged with 2-chloro-1,3-dimethylimidazolinium chloride (29.2 mg, 0.173 mmol), carboxylic acid (24.6 mg, 0.150 mmol), and a stir bar. CH_2Cl_2 (0.75 mL) was added followed by N,N-diisopropylethylamine (45 mg, 0.34 mmol , 60 uL), resulting in fuming. The mixture was stirred at room temperature for 5 min, following addition of (R)-(-)-N-Benzyl-alpha-methylbenzylamine (39.7 mg, 0.188 mmol) in CH_2Cl_2 (0.75 mL). The reaction stirred at room temperature for 80 min. The reaction was diluted to 10 mL with CH_2Cl_2 then washed with water (2 mL), followed by sequential washing with $NaHCO_3$ (2 mL, aq), 0.1 N HCl (2mL), and brine (2mL). The organic layer was dried (Na_2SO_4), filtered, and concentrated in vacuo. Purification via flash column chromatography (4 g silica gel, eluted with 15% ethyl acetate in hexanes, isocratic) afforded **PF-95** (10.6 mg, 20%) as a colorless oil.

^1H NMR (400 MHz, CD_3CN) δ 7.45 – 7.14 (m, 10H), 5.29 – 4.68 (m, 2H), 4.20 – 3.75 (m, 1H), 3.08 – 2.96 (m, 1H), 2.90 (br. S, 2H), 2.89 – 2.84 (m, 1H), 2.40 (t, J = 2.7 Hz, 1H), 2.29 – 2.21 (m, 1H), 2.21 – 2.15 (m, 1H), 1.53 (d, J = 6.9 Hz, 3H). ^{13}C NMR (101 MHz, CD_3CN) δ 174.36, 141.90, 140.30, 129.71, 129.03, 128.31, 127.73, 127.54, 127.33, 80.64, 72.68, 56.77, 48.10, 42.08, 40.44, 28.91, 27.62, 19.88. HRMS (ESI+) [M+H]+ calculated for $C_{23}H_{23}N_3O$ 358.1914, find 358.1914.

(S)-N-benzyl-N-(1-phenylethyl)-5-(prop-2-yn-1-yl)-1,2-diazaspiro[2.3]hex-1-ene-5-carboxamide (46)

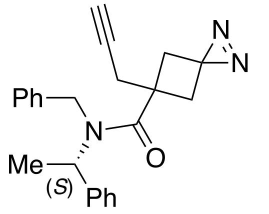

A 1-dram vial was charged with 2-chloro-1,3-dimethylimidazolinium chloride (29.2 mg, 0.173 mmol), carboxylic acid (24.6 mg, 0.150 mmol), and a stir bar. CH_2Cl_2 (0.75 mL) was added followed by N,N-diisopropylethylamine (45 mg, 0.34 mmol , 60 uL), resulting in fuming. The mixture was stirred at room temperature for 5 min, following addition of (S)-(+)-N-Benzyl-alpha-methylbenzylamine (38.1 mg, 0.180 mmol) in CH_2Cl_2 (0.75 mL). The reaction stirred at room temperature for 80 min. The reaction was diluted to 10 mL with CH_2Cl_2 then washed with water (2 mL), followed by sequential washing with $NaHCO_3$ (2 mL, aq), 0.1 N HCl (2mL), and brine (2mL). The organic layer was dried (Na_2SO_4), filtered, and concentrated in vacuo. Purification via flash column chromatography (4 g silica gel, gradient elution 0→25% ethyl acetate in hexanes) afforded **PF-96** (18.6 mg, 35%) as a colorless oil.

^{1}H NMR (400 MHz, CD_3CN) δ 7.49 – 7.10 (m, 10H), 5.31 – 4.65 (m, 2H), 4.00 (br. s, 1H), 3.02 (d, J = 13.9 Hz, 1H), 2.91 (br. s, 2H), 2.89 – 2.84 (m, 1H), 2.40 (t, J = 2.6 Hz, 1H), 2.30 – 2.21 (m, 1H), 2.21 – 2.14 (m, 1H), 1.53 (d, J = 6.9 Hz, 3H). ^{13}C NMR (101 MHz, CD_3CN) δ 174.35, 141.90, 140.30, 129.71, 129.03, 128.31, 127.73, 127.54, 127.33, 80.64, 72.68, 56.76, 48.10, 42.08, 40.43 (d, J = 3.8 Hz), 28.91, 27.62, 19.88. HRMS (ESI+) [M+H]+ calculated for $C_{23}H_{23}N_3O$ 358.1914, find 358.1930.

3-(3-(But-3-yn-1-yl)-3H-diazirin-3-yl)-N-(2-oxo-2,3,4,5-tetrahydro-1H-benzo[b]azepin-8-yl)propenamide (38)

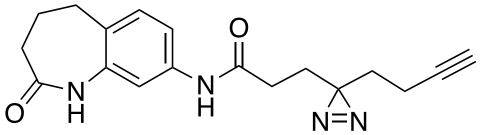

To a vial containing the corresponding amine intermediate (1 eq.) in dichloromethane (60 mM), commercially available 3-(3-(but-3-yn-1-yl)-3H-diazirin-3-yl)propanoic acid (1.1 eq.), N, N-diisopropylethylamine (3.0 eq.), EDC-HCl (1.5 eq.) and HOBt (1.5 eq.) were added and stirred at room temperature for 12 h. After completion (monitored by TLC) the crude mixture was diluted

with dichloromethane. Saturated aqueous NH_4Cl solution was added and extracted in dichloromethane. The combined extracts were washed with saturated aqueous $NaHCO_3$ solution, extracted in dichloromethane, dried over anhydrous Na_2SO_4, and volatiles removed by rotary evaporation. Crude products were purified by flash chromatography (Biotage®) with a 0 to 40 % ethyl acetate gradient in hexane to afford **AJ-25** as an off white solid (9 mg, 64%).^{1}H NMR (400 MHz, $CDCl_3$) δ 8.27 (s, 1H), 8.00 (s, 1H), 7.33 – 7.23 (m, 2H), 7.13 (d, *J* = 8.1 Hz, 1H), 2.73 (t, *J* = 7.2 Hz, 2H), 2.33 (t, *J* = 7.3 Hz, 2H), 2.26 – 2.09 (m, 4H), 2.06 – 1.98 (m, 3H), 1.95 – 1.90 (m, 2H), 1.67 (t, *J* = 7.3 Hz, 2H). ^{13}C NMR (101 MHz, $CDCl_3$) δ 175.59, 169.87, 138.14, 137.26, 130.21, 130.10, 117.02, 113.51, 82.74, 69.36, 32.89, 32.40, 31.20, 29.77, 28.50, 28.17, 27.92, 13.32. LCMS *calcd for* $C_{18}H_{21}N_4O_2$, 325.2 (M+H$^+$), *found*: 325.2.

Photolysis Experiments

Synthesis of Benzyl 1,1,2,2-tetramethylspiro[2.3]hexane-5-carboxylate (30)

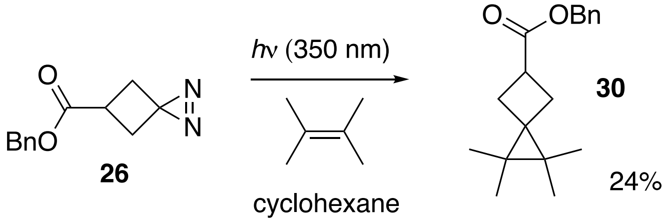

A quartz cuvette (pathlength = 1 cm^2) was charged with **26** (42 mg, 0.19 mmol), tetramethylethylene (531 mg, 6.31 mmol), and cyclohexane (2.25 mL). The cuvette was capped and irradiated with UV-B light for two hours in a Rayonet-type photobox outfitted with four 8-Watt RPR-3500A bulbs. The sample analyzed by GC (FID) and was then transferred to flask, concentrated, re-dissolved in methylene chloride, and chromatographed via preparatory TLC with 12% ethyl acetate in hexanes. The title compound was obtained as a clear oil (12.9 mg, 0.047 mmol, 24%).

1H NMR (600 MHz, CD_3OD) δ 7.40-7.35 (m, 5H), 5.17 (s, 2H), 3.13-3.10 (m, 1H), 2.21-2.14 (m, 4H), 0.93 (s, 6H), 0.88 (s, 6H) ppm; 13C NMR (150 MHz, CD_3OD) δ 177.4 (C), 137.8 (C), 129.5 (CH), 129.2 (CH), 129.2 (CH), 67.3 (CH2), 32.9 (CH), 26.8 (CH_2), 22.2 (C), 22.1 (C), 18.2 (CH_3), 18.1 (CH_3) ppm; HRMS (ESI+) $[M+H]^+$ Calculated for $C_{18}H_{25}O_2^+$ 273.1855; found 273.1847.

A

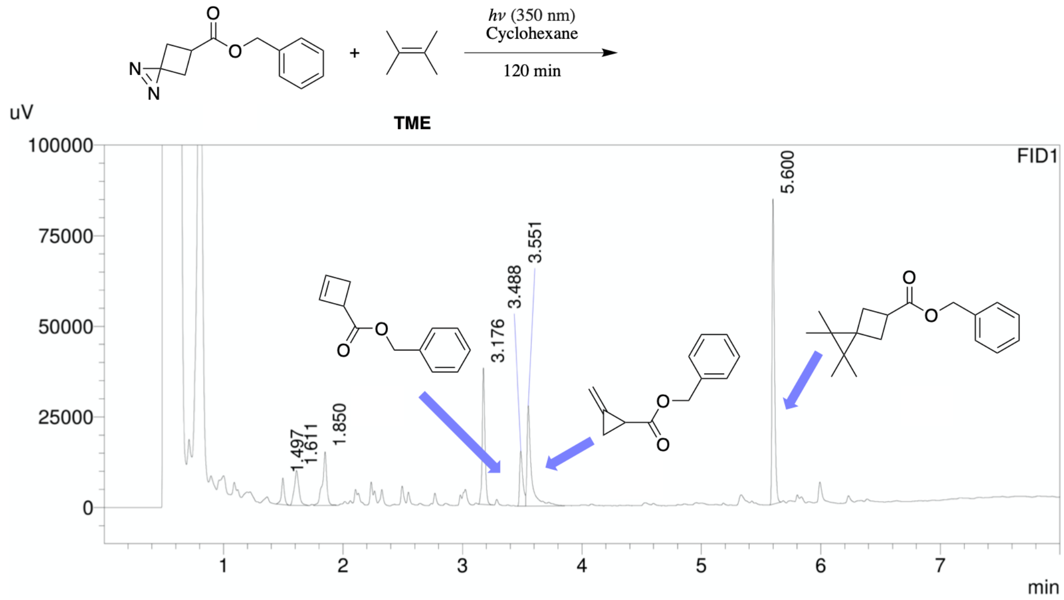

B

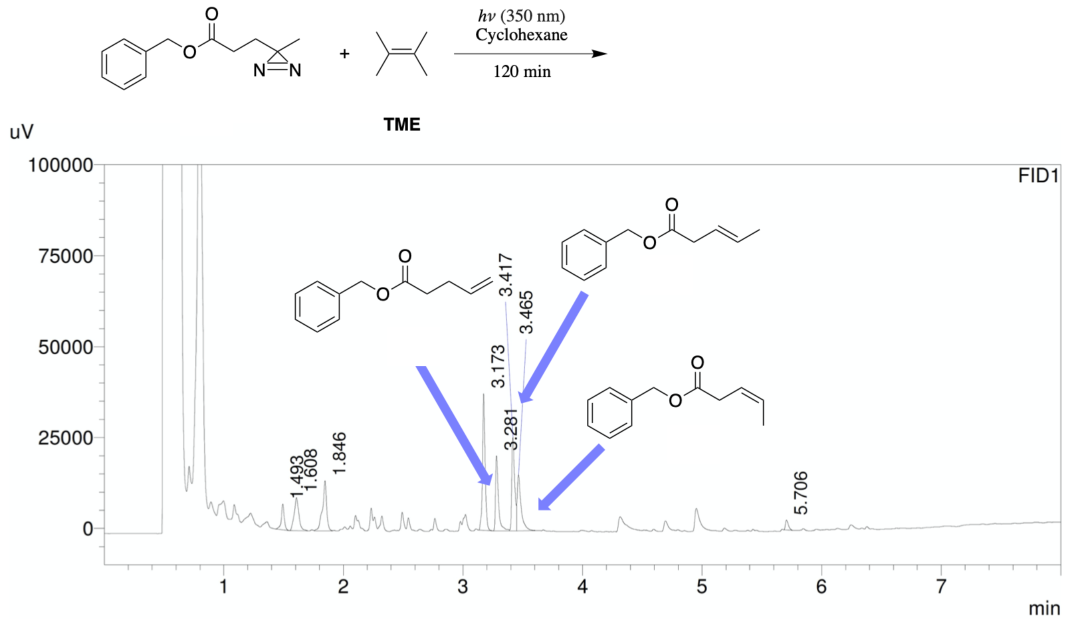

Figure S-1: (A) GC-FID of 350 nm irradiation of **26** in cyclohexane and 2 M TME for 120 min. Cycloadduct **30** is formed at retention time 5.60 min and is isolated in 24% yield. (B) GC-FID of 350 nm irradiation of linear diazirine in cyclohexane and 2 M TME for 120 min produces intramolecular alkene products. The peak at 5.71 min represents <2% of total integration of peaks eluting after 1.4 min.

Photolysis of Diazirines 26 and 27

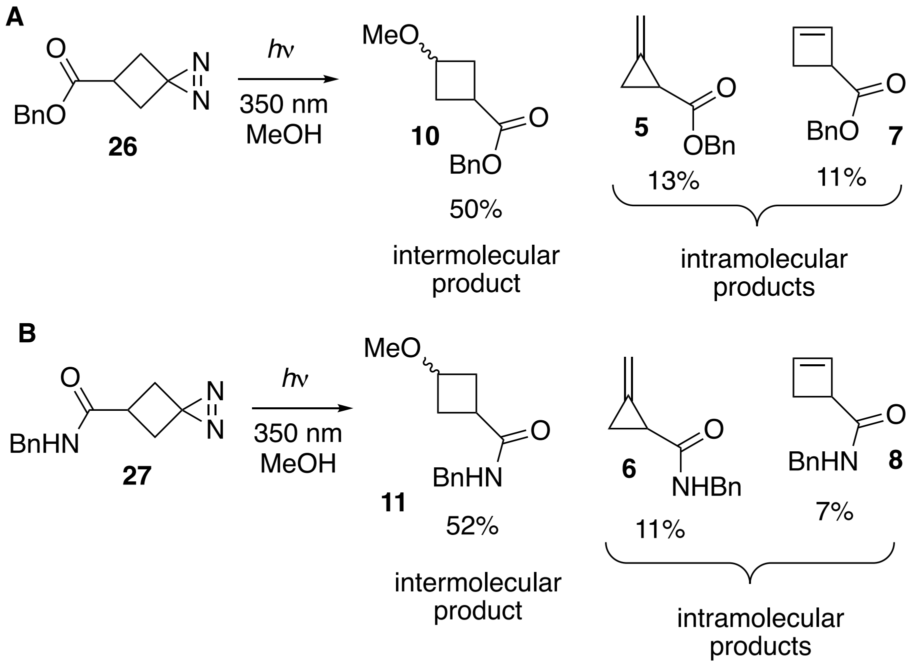

A quartz cuvette (pathlength = 1 cm^2) was charged with a solution of diazirine **26** (8 mM) or **27** (8 mM) in methanol. The resulting solution was irradiated with a 365 nm lamp (Spectroline model XX15A longwave) with light intensity 19 mW/cm^2 and irradiated at 365 nm 1 mm from the handheld light source for 15 minutes with monitoring of diazirine conversion via UV-Vis. The reaction was concentrated, then product yields were quantified by ^1H NMR on a Bruker 600 MHz spectrometer by comparing product peak integration to the integration of aromatic protons (5H) in the region 7.2–7.5 ppm as well as comparison to an external mesitylene standard. NMR spectra were compared to those of independently prepared samples.

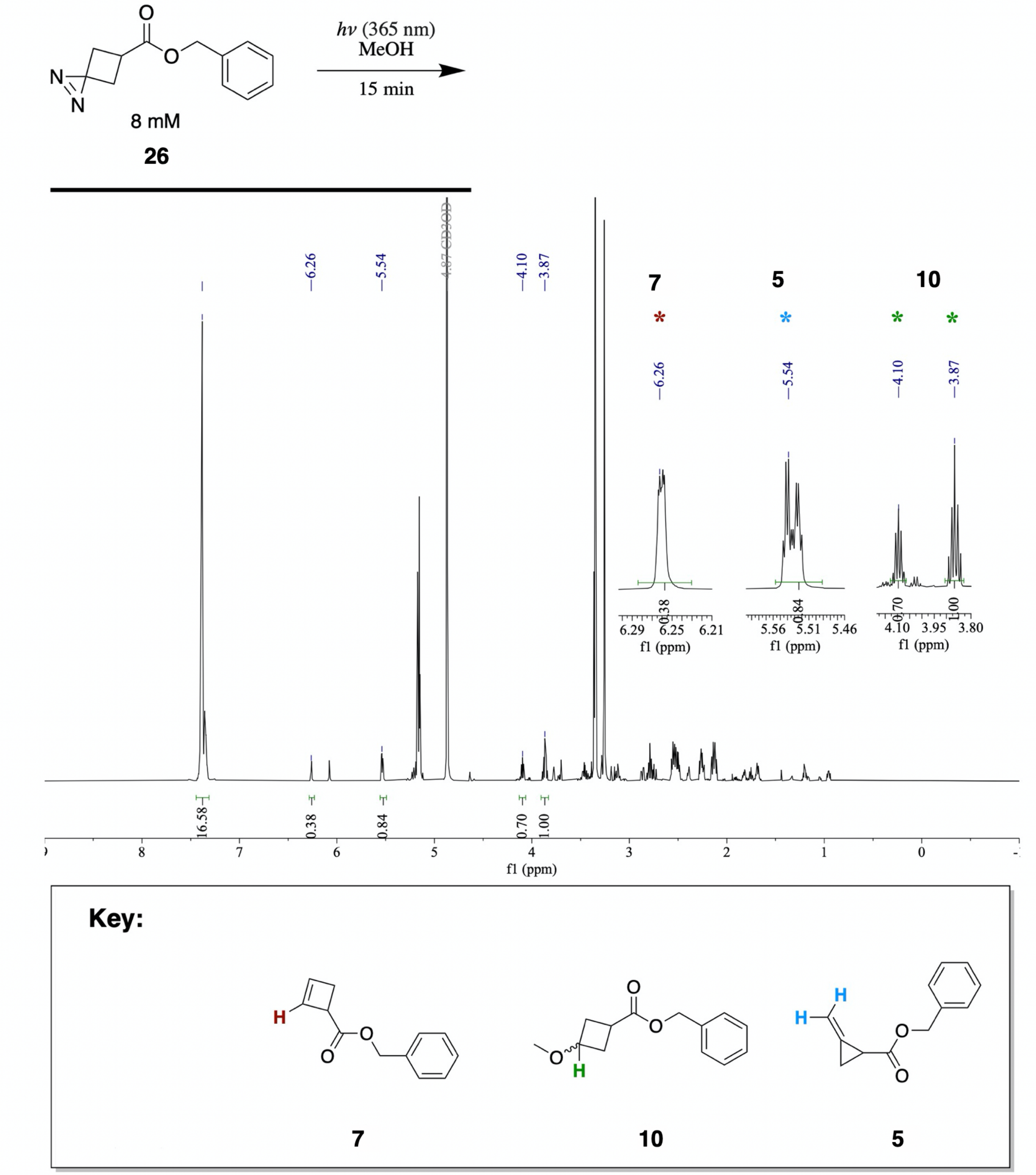

Figure S-2. 8 mM diazirine **26** was irradiated at 365 nm in methanol for 15 minutes to produce products **5**, **7** and **10**.

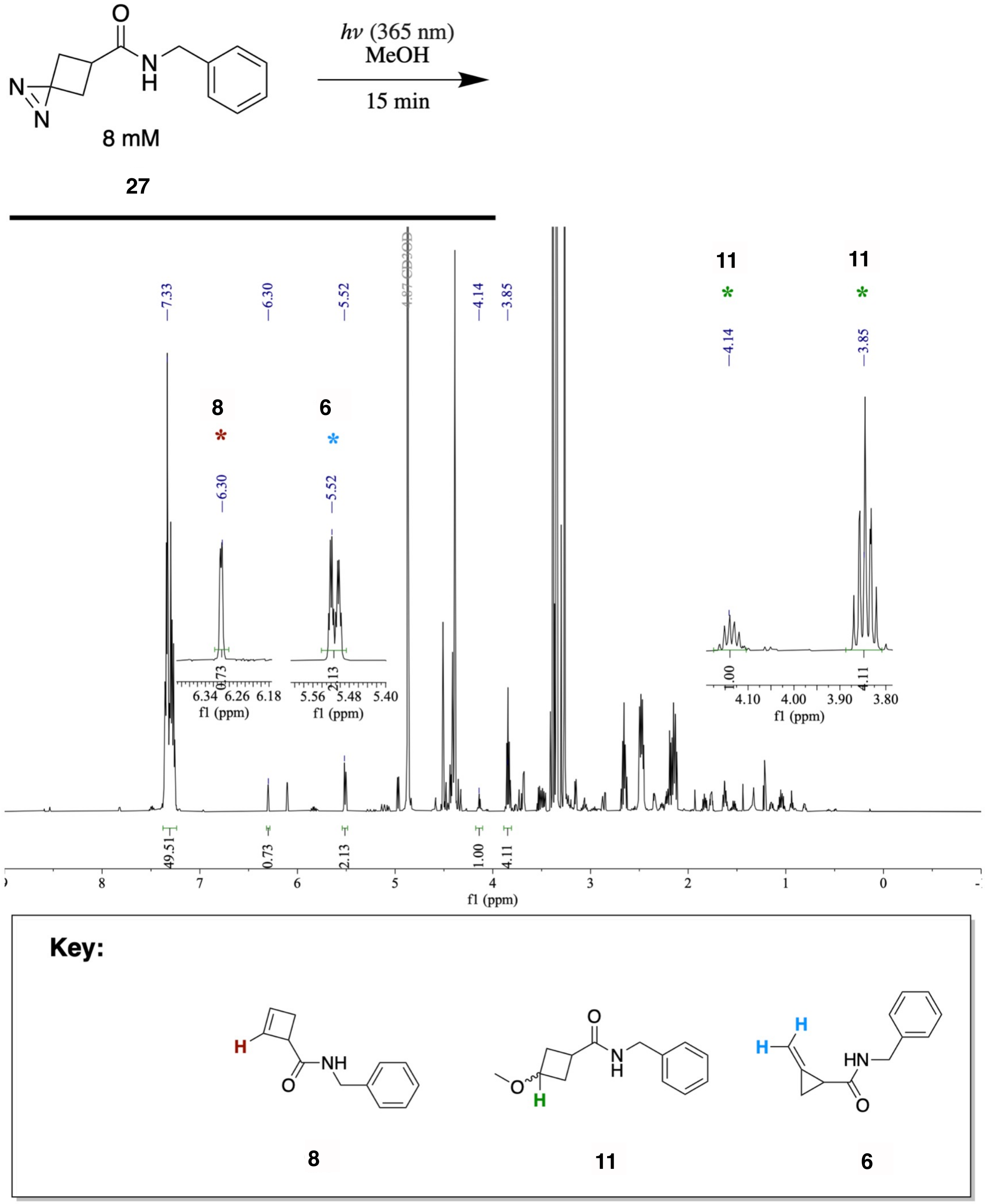

Figure S-3. 8 mM diazirine **9** was irradiated at 365 nm in methanol for 15 minutes to produce products **10–12**.

Photolysis of 1 to produce diazocyclobutane (2) and measurement of decay of 2 by UV-Vis

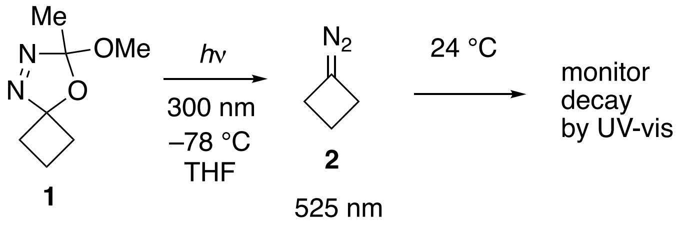

Warning: Diazo compounds should be treated as highly toxic compounds and should handled with appropriate personal protective equipment, including a well-ventilated fume hood. We advise that dialkyldiazo compound **2** should only be handled in small quantities and in dilute solution, as the energetic properties of the pure diazo compound are unknown.

A Rayonet RPR200 photoreactor was outfitted with eight (8) UVB bulbs (8 watt, Luzchem, LZC-UVB, 300 nm), and a rod and clamp that could hold a vial ~10 cm from the light source. A dry 20 mL Borosilicate vial (CG-4904-01) was charged with 3 mL of a 0.1 M solution of oxadiazoline **1**.[14] A test tube (16 x 150 mm) was inserted into the vial, and electrical tape was used to seal the interface. The test tube was filled with crushed dry ice and ~1 mL of acetone to cool the solution, which was irradiated. As needed, frost was wiped from the surface of the vial during the photolysis. After 15 min, the Rayonet was turned off, and the resulting pink solution was immediately transferred to a quartz cuvette (1 mL volume, 1 cm pathlength) which was capped and transferred to an Agilent 8453 UV-vis with a thermostatted cuvette holder maintained at 24 °C. The decomposition of **2** was monitored by the decrease in absorbance at 525 nm, and the kinetics of decay were fitted to a first-order rate law using GraphPad Prism software.

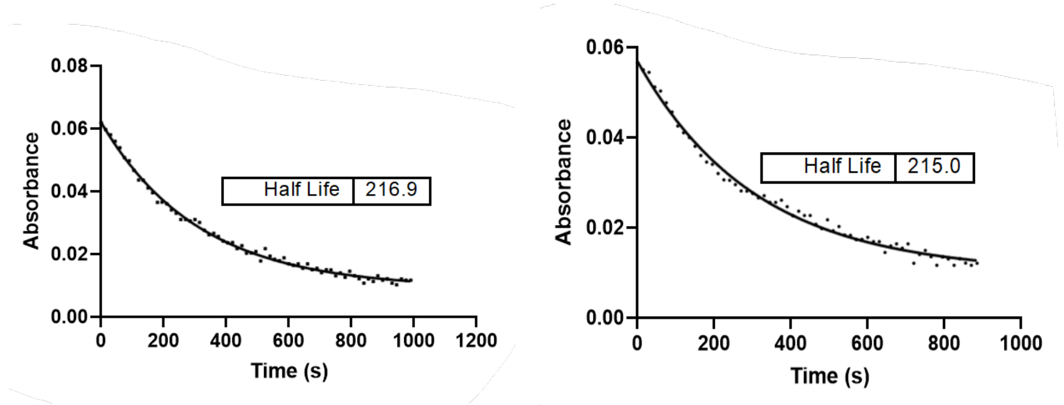

Figure S4. Decay rate of **2** fitted to first-order rate law. Duplicate runs displayed

Conversion of diazocyclobutane (2) is not accelerated by 350 nm irradiation

A solution of diazo **2** was prepared as described above and transferred to a quartz cuvette (1 mL volume, 1 cm pathlength) which was covered with a small plate of borosilicate glass and then transferred to an Agilent 8453 UV-vis with a thermostatted cuvette holder maintained at 24 °C. From the top, the cuvette was measured by a single UVA bulb (8 Watt, Rayonet RPR3500 Å bulb, Intensity = 20 mW/cm^2 measured at the distance of the bulb from the center of the cuvette). The decomposition of **2** was monitored by the decrease in absorbance at 525 nm, and the kinetics of decay were fitted to a first-order rate law using GraphPad Prism software.

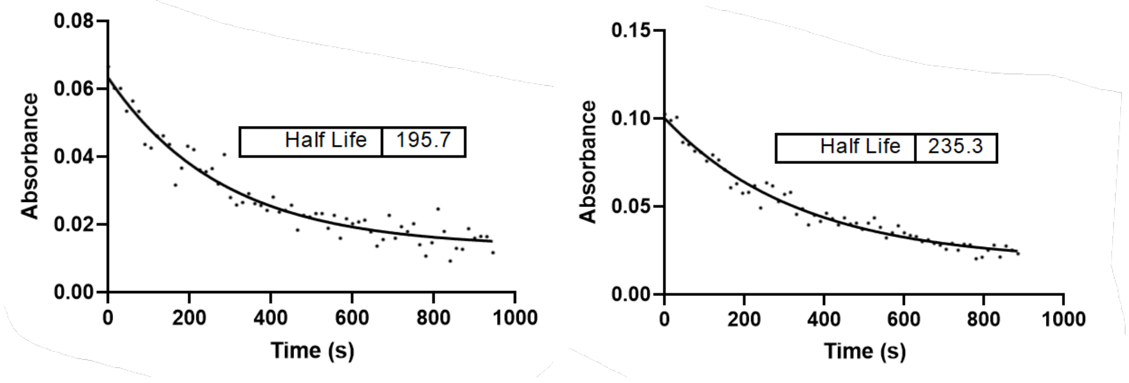

Figure S5. Decay rate of **2** while irradiated with 350 nm bulb, fitted to first-order rate law. Duplicate runs displayed.

Conversion of diazocyclobutane (2) is accelerated by addition of waterA solution of diazo **2** was prepared as described above. 1 mL of this solution was transferred to a quartz cuvette (1 mL

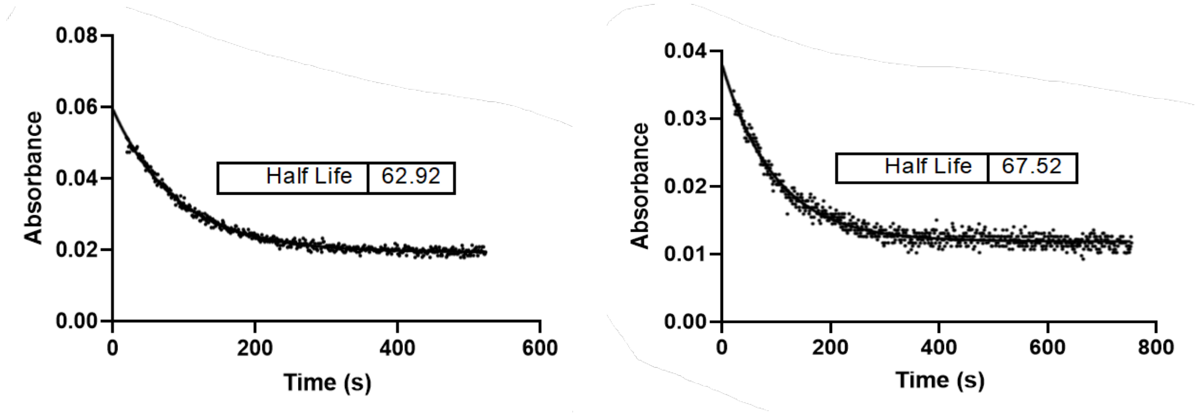

Figure S6. Decay rate of **2** with the addition of water, fitted to first-order rate law. Duplicate runs displayed

volume, 1 cm pathlength) which capped and transferred to an Agilent 8453 UV-vis with a thermostatted cuvette holder maintained at 24 °C. The decomposition of **2** was monitored by the decrease in absorbance at 525 nm. Water (0.1 mL) was added to the cuvette, which was mixed with a pipette. Reaction monitoring by UV-vis was resumed immediately. Kinetics of decay were fitted to a first-order rate law using GraphPad Prism software.

Photolyses of 31 to detect diazocyclobutane 34 by UV-Vis

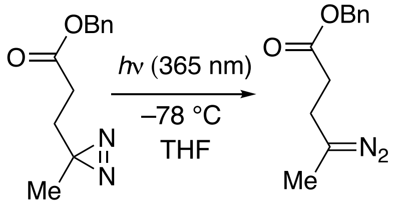

31 **34**

Warning: Diazo compounds should be treated as highly toxic compounds and should handled with appropriate personal protective equipment, including a well-ventilated fume hood. All diazo compounds should only be handled in small quantities and in dilute solution, as the energetic properties of the pure diazo compounds are unknown.

A dry 7 mL Borosilicate vial (CG-4904-01) was charged with 1 mL of a 0.16 M solution of diazirine **31**. The vial was capped and partially immersed in a beaker of dry ice/dichloromethane, and tilted at a ~45 ° angle. The vial was irradiated from the top with a Blak-ray long wave 365 nm ultraviolet lamp (model B 100 AP) lamp. Within 10 min, the solution became peach in color. After a total of 15 min irradation, and the resulting peach solution was warmed to r.t., transferred to a 1 mL quartz cuvette, and analyzed by UV-vis spectroscopy which revealed an absorption with λ_{max} 500 nm.

Photolyses of 26

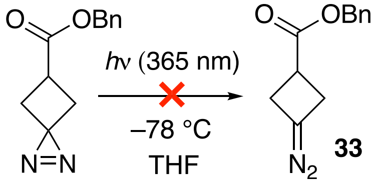

33

26 *not detected*

A dry 7 mL Borosilicate vial (CG-4904-01) was charged with 1 mL of a 0.16 M solution of diazirine **26**. The vial was capped and partially immersed in a beaker of dry

ice/dichloromethane, and tilted at a ~45 ° angle. The vial was irradiated with a Blak-ray long wave 365 nm ultraviolet lamp (model B 100 AP) lamp lamp for up to 30 min. At no time during the irradiation was a color change noted. Separate reactions were carried out for 15 or 30 min, and analyzed by UV-vis spectroscopy, but no absorption at ≥500 nm was noted. A similar irradiation experiment was carried out with a 0.10 M solution of **26**, but again no color change was noted, and no new absorption at ≥500 nm was noted after 30 min. An aliquot from this solution was diluted to 2 mM, and reanalyzed by UV-vis to show that diazirine **26** had been completely consumed as shown by disappearance of the diazirine absorption at 350 nm.

BSA labeling by 2

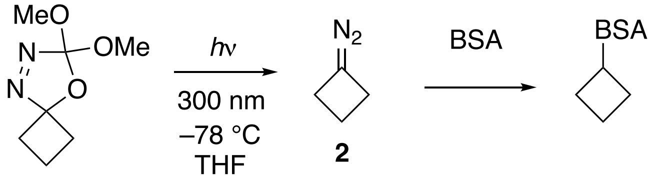

0.98 mL of 0.1 M oxadiazoline in dry THF (under argon) was injected into a thin-walled borosilicate NMR tube. The tube was quickly purged with argon and sealed after transfer. Alternating cycles of 5 minutes cooling at -78°C (dry ice) followed by 5 minutes irradiated with 302 nm UV light were carried out for a total of 50 minutes until maximum pink colour change was observed, indicating the formation of **2**. Bovine Serum Albumin (BSA, 0.2 mL of 10 mg/mL (150 μM) in double-distilled H_2O was then injected into the solution containing diazocyclobutane in THF. The pink colour rapidly dissipated after addition of aqueous BSA and bubbling continued for <1h as N_2 was liberated. When the bubbling of solution had stopped, the protein was precipitated by adding 5X volume of acetone and incubating at 80 °C overnight. A BSA (only) and THF solution was also prepared to serve as the negative control for labeling. This acetone precipitation step also served to remove unreacted oxadiazoline, and THF from the labeled protein. The samples were then centrifuged at 4 °C, maximum speed (20,800g) for 15 minutes. The supernatant was removed, and the resulting pellet was then dried for <5 minutes before being resuspended in 50 mM ammonium bicarbonate with 6 M urea. Samples were reduced with DTT to 25 mM final concentration and the sample was incubated at 65 °C for 15 minutes. To alkylate the samples, Iodoacetamide was then added to 25 mM final concentration and the samples were incubated, rotating, in the absence of light for 30 minutes at room

temperature. Following the incubation, 50 mM ammonium bicarbonate was added to dilute urea to <2 M, and LC-MS grade trypsin was added (final concentration of 0.5 mg/mL) before incubating overnight at 37 °C. The following morning, the samples were dried by vacuum centrifugation and resuspended in 100 mM triethylammonium bicarbonate before being acidified to pH <4 with 5% formic acid (verified via litmus paper). The samples were then desalted using Pierce C18 tips *(Thermofisher)* according to manufacturer protocol. Following desalting, samples were dried by vacuum centrifugation and submitted to the Holmes centre for mass spectrometry (University of Ottawa) for LC-MS/MS.

Mass spectrometry and data analysis for BSA labeling

LC-MS/MS was performed by Zoran Minic at the Holmes Mass Spectrometry Facility at University of Ottawa using an Orbitrap Fusion mass spectrometer (Thermo Fisher Scientific) coupled to an UltiMate 3000 nanoRSLC (Dionex; Thermo Fisher Scientific). An in-house packed column (Polymicro Technology) 15 cm × 70 µm ID, Luna C18(2), 3 µm, 100 Å (Phenomenex) with a water/acetonitrile/0.1% formic acid gradient was first used to separate the trypsinized peptides. To achieve this, a flow rate of 0.30 µL/min was applied for 105 mins with the following breakdown: 2% acetonitrile used for the first 7 mins, 2-38% acetonitrile (linear gradient) for the next 70 mins, 38-98% acetonitrile (linear gradient) for 9 mins, 98% acetonitrile for 10 mins, 98-2% acetonitrile (linear gradient) for 3 mins, and finally 2% acetonitrile for 10 mins. Positive electrospray ionization (ESI) with ion source temperature of 250°C and 2.1 kV was employed to inject the eluted peptides directly in the mass spectrometer. The mass spectrometer was run in top speed mode and the full-scan MS spectra of m/z 350-2000 was acquired at a resolution of 60,000. Monoisotopic precursor selection, charge sate (+2 to +7), and dynamic exclusion of 30s with a +/- 10 ppm window were all used to filter the precursor ions. For FTMS scans and MS/MS scans the automatic gain control settings were set at 5×10^5 and 1×10^4, respectively. Fragmentation was performed with collision-induced dissociation (CID) in the linear ion trap. A 2 m/z isolation window was used to isolate precursors, and these were fragmented with 35% normalized collision energy. Each sample was run in technical triplicates. Data analysis of LC-MS/MS data was performed by Nico Huettmann of the Holmes Mass Spectrometry facility at University of Ottawa. The raw MS files were analyzed using MaxQuant (version 2.4.0.0) and the Andromeda search engine. Peptides were searched against the NCBI

Reference Sequence NP_851335.1 for wild type bovine serum albumin and a contaminants database. Default parameters were used unless specified otherwise. N-terminal acetylation and methionine oxidation were set as variable modifications while cysteine carbamidomethylation was specified as a fixed modification. Cyclobutane was defined as a variable modification on K, C, S, H, D, and E residues represented by addition of C_4H_7 (and loss of nucleophilic residue H). To find potential PTMs with unexpected mass, the dependant peptides function was enabled. The minimum peptide length was set to 6 amino acids. The false discovery rate (FDR) was set to 0.01 for protein and peptides and was determined by searching against a reverse sequence database. The enzyme specificity was set to a maximum of 2 missed cleavages for C-terminal R and K. The initial precursor mass deviation was set to a maximum of 10 ppm with a fragment mass deviation equaling 0.5 Da. To increase peptide identification rate, the "match between runs" MaxQuant algorithm was applied between samples. All proteins and peptides that matched the reverse database were removed.

Results: A mass shift of +54 Da corresponding to the addition of a cyclobutane with the loss of 1 proton from the nucleophile was observed on 229 specific unique BSA amino acid side chains. BSA contains 60 lysine, 35 cysteine, 32 serine, 17 histidine, 40 aspartate, and 59 glutamate residues. These residues account for 243 of the 607 amino acids in BSA and represent nucleophilic residues likely to react with the diazocyclobutane; therefore, the addition of +54 Da was screened for and detected on each of these residues. Each of these residues displayed the 54 Da addition with the following percent of unique detected amino acid labeling to amino acid prevalence in BSA: 98% Lys, 94% Cys, 72% Ser, 94% His, 100% Asp, and 98% Glu. The results suggest that, if cyclobutanediazirine photolysis produces diazocyclobutane intermediates, that non-photochemical alkylation of proteins by diazocyclobutane is possible.
Therefore, the LC-MS/MS results indicate a high degree of labeling for all nucleophilic amino acid residues screened for with a slight bias against serine. This may be due to the acidity of the residues allowing for protonation of the cyclobutyldiazo followed by nucleophilic attack.

Treatment of Live Cells with Fully-Functionalized Probes HEK293T cells were grown to *circa* 80 % confluence in six-well plates (in-gel fluorescence experiments) or 10 cm plates (proteomics experiments) in Dulbecco's modified Eagle medium (DMEM) supplemented with fetal bovine serum (FBS, 10 % final volume), penicillin-streptomycin (100 U/ml final concentration), and glutamine. The growth medium was aspirated and replaced with a solution of the compound at the appropriate concentration in serum-free DMEM (1 ml / well or 3 ml / plate). The cells were incubated (37 ºC, 30 min, 5 % CO_2), then irradiated with UV light (Analytik Jena photocrosslinker equipped with 5 × G8T5E UV bulbs, 365 nm, 4 ºC, 20 min). The cells were scraped, centrifuged (500 g, 4 ºC, 3 min), and the supernatant was aspirated. The pellet was resuspended in DPBS, and again centrifuged and the supernatant aspirated. The pellet was stored at -80 ºC before the next steps in the procedure.

In-gel Fluorescence Experiments The cell pellets were lysed in DPBS (150 µl) supplemented with Halt™ protease inhibitor cocktail (1 ×, Thermo Scientific 78429) by sonication (Branson Sonifier probe sonicator, 15 ms on, 40 ms off, 15 % amplitude, 1 s total on, repeated once). The lysate was centrifuged (100,000 × g, 45 min). The pellet was resuspended in protease inhibitor-supplemented DPBS and the protein concentrations of the soluble and insoluble fractions were normalized (2 mg / ml, 50 µl) using a modified Lowry protein assay (Bio-Rad 5000113, 5000114). Rhodamine was conjugated to the protein-bound probes through a copper-catalyzed azide-alkyne cycloaddition by treating each lysate with a solution consisting of tris[(1-benzyl-1H-1,2,3-triazol-4-yl)methyl]amine (TBTA, 3 µl, 1.7mM in DMSO-tBuOH 1:4 *v*/*v*) tris(2-carboxyethyl)phosphine (TCEP, 1 µl, 50 mM in H_2O), tetramethyl rhodamine azide (1 µl, 1.25 mM in DMSO), and copper sulfate (1 µl, 50 mM in H_2O). The reaction was allowed to proceed at room temperature for 1 h with shaking. The samples were combined with 4× SDS gel loading buffer (17 µl) and vortexed. The proteins were resolved by SDS-PAGE (10 % acrylamide) and visualized by in-gel fluorescence.

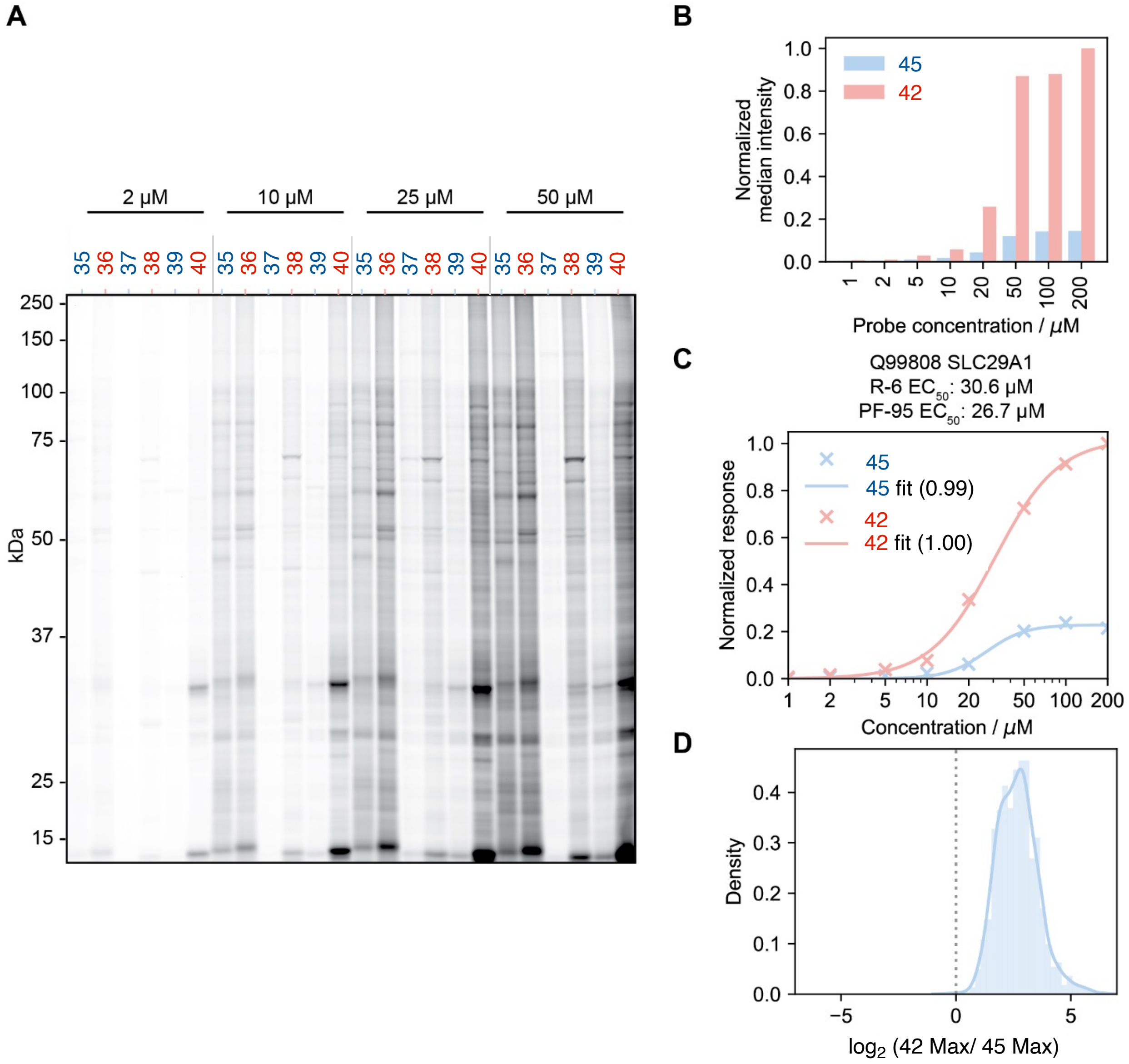

Figure S7. Cyclobutanediazirines exhibit reduced labeling relative to linear dialkyl diazirines. **A** Particulate lysate fraction derived from HEK293T cells treated with spirocyclic and linear dialkyl diazirine probes (30 min) followed by UV irradiation (365 nm for 20 min), conjugated to TAMRA-azide, and visualized by in-gel fluorescence. **B** LC-MS proteomic median abundances of proteins enriched by **45** and **42** at different concentrations. **C** LC-MS proteomic abundances for representative proteins treated with different concentrations of probes **45** and **42**. **D** Comparing the ratios of maximum intensity for proteins that possess similar **45** and **42** EC_{50} values (within 20 %) shows that the linear dialkyl diazirine probes label proteins with approximately eight to sixteenfold greater efficiency.

TMT-Labeled Streptavidin Enrichment Proteomics

The cell pellets were lysed in DPBS (500 µl) supplemented with Halt™ protease inhibitor cocktail (1 ×, Thermo Scientific 78429) by sonication (Branson Sonifier probe sonicator, 15 ms on, 40 ms off, 15 % amplitude, 1 s total on, repeated once). The lysate protein concentration was normalized as above (2 mg/ml, 500 µl). A solution consisting of TBTA (30 µl, 1.7 mM in DMSO/t-BuOH 1:4 *v/v*), TCEP (10 µl, 50 mM in H_2O), biotin-PEG3-azide (5 µl, 100 µM in DMSO), and copper sulfate (10 µl, 50 mM in H_2O) was added to each sample and shaken (1 h, rt). To each sample was added a solution of cold MeOH/$CHCl_3$ (2 ml, 4:1 v/v) followed by cold DPBS (1 ml). The samples were vortexed and centrifuged (4,700 x g, 10 min, 4 °C). The organic and aqueous layers were carefully removed to leave the protein interphase disc, which was then washed through the addition of cold MeOH/$CHCl_3$ (2 ml, 4:1 v/v) followed by resuspension using a water bath sonicator and centrifugation (4,700 x g, 10 min, 4 °C). This washing procedure was repeated two additional times. After removing the supernatant, urea solution (500 µl in DPBS, 6 M) and SDS (10 µl, 10 % *w/w* in H_2O) were added to the pellets, which were resuspended through probe sonication as above. A solution of (50 µl) of TCEP (100 mM) and K_2CO_3 (300 mM) in DPBS was added to each sample, and the samples were incubated with shaking (30 min, 37 °C). A solution of iodoacetamide (70 µl, 400 mM in DPBS) was then added and the samples were incubated in the absence of light (30 min, rt). SDS solution (130 µl, 10 % in DPBS *w/w*) was added to each sample, followed by DPBS (5.5 ml). Agarose resin-bound streptavidin (100 µl / sample, Thermo Scientific 20353) was washed with DPBS (3 × resuspension and centrifugation at 400 g, 2 min, 4 ºC), resuspended (100 µl final volume in DPBS), added to the sample, and rotated for 1.5 h. The samples were centrifuged (400 g, 2 min, 4 ºC) and the supernatant was aspirated. The remaining resin was washed with a solutions of SDS (0.2 % *w/w* in DPBS), DPBS × 2, water, and triethylammonium bicarbonate buffer (TEAB 100 mM, pH 8.5) through centrifugation, supernatant aspiration, and resuspension (400 g, 2 min, 5 ml / sample). A solution of TEAB (1 ml, 100 mM) was used to transfer the resin to low-protein binding centrifuge tubes (Eppendorf 022431081). The samples were centrifuged (400 g, 2 min, 4 ºC) and the supernatant removed. To each sample, sequencing grade modified trypsin (2 µg, Promega, V5111) in TEAB (200 µl, 100 mM, pH 8.5) was added, followed by calcium chloride

solution (20 µl, 100 mM). The resin was resuspended by vortexing and incubated (16 h, 37 ºC with shaking). The samples were centrifuged (400 g, 2 min, 4 ºC) and the supernatants were transferred to fresh low-binding centrifuge tubes. The beads were washed with a further volume of TEAB (100 µl) to ensure complete transfer and acetonitrile (120 µl) was added to each of the combined washings. An aliquot of the appropriate TMTpro™ 16plex reagent (Thermo Scientific™, A44520) was added to each sample and incubated (1 h, r.t., with shaking). Hydroxylamine solution (6 µl, 5 % *v*/*v*) was added to each sample and incubated for a further 15 min, followed by formic acid (4 µl). The samples were then dried by vacuum centrifugation. The residues were recombined into a solution of trifluoroacetic acid (300 µl, 0.1 % in H_2O) and fractionated into seven fractions using a high pH fractionation kit (Thermo Fisher Scientific 84868) according to the manufacturer's instructions. The fractions were dried by vacuum centrifugation and the peptidic residues were dissolved in formic acid solution (65 µl, 0.1 % in H_2O), centrifuged (10,000 g, 10 min), and a portion (50 µl) was transferred to vials in preparation for mass spectrometric analysis.

Mass Spectrometric Analysis A volume of each sample (10 µl) was loaded onto an Acclaim PepMap 100 precolumn (75 µm x 2 mm) and eluted on an Acclaim PepMap RSLC analytical column (75 µm x 15 cm) using the UltiMate 3000 RSLCnano system (Thermo Fisher Scientific). A gradient (flow rate 0.3 ml/min, 35 °C) of 98 % buffer A (0.1 % formic acid in H_2O) and 2 % buffer B (0.1 % formic acid in MeCN) was applied for 10 min, followed by an increase to 30 % buffer B over 192 min, to 60 % buffer B over 5 min, rising to 95 % buffer B over 1 min. After 5 min isocratic elution with 95 % buffer B, buffer B was decreased to 2 % over 1 min followed by re-equilibration at 2 % for 6 min. The eluted peptides were analyzed with a Thermo Fisher Scientific Orbitrap Fusion or Fusion Lumos mass spectrometer with a cycle time of 3 s and nano-LC electrospray ionization source applied voltage of 2.0 kV. MS1 spectra were recorded at a resolution of 120,000 with an automatic gain control (AGC) value of $1x10^6$ ions, maximum injection time of 50 ms (dynamic exclusion enabled, repeat count 1, duration 20 s). The scan range was specified from 375 to 1,500 *m/z*. Peptide fragmentation MS2 spectra were recorded via collision-induced diffusion (CID) and quadrupole ion trap analysis (AGC $1.8x10^4$, 30 % collision energy, maximum inject time 120 ms, isolation window 1.6). MS3 spectra were

generated by high-energy collision-induced dissociation (HCD) with collision energy of 65 %. Precursor selection included up to 10 MS2 ions for the MS3 spectrum.

Mass Spectrometric Data Analysis

Raw spectral files were processed using Proteome Discoverer 2.4 (Thermo Fisher Scientific) Peptides were identified using the SEQUEST HT algorithm searching against a *H. Sapiens* proteome database (42,358 sequences). Fragment tolerances were set to 0.6 Da, and precursor mass tolerances set to 10 ppm with one missed cleavage site allowed. Static modifications were defined for the carbamidomethyl group (C, +57.0215) and TMT-tag (K, N-terminal, +229.1629) while methionine oxidation (M, +15.9949) was set as a variable modification. Spectra were filtered using the Percolator algorithm with a false discovery rate of 1 %. MS3 peptide quantitation was performed with a mass tolerance of 20 ppm. Identified proteins were required to have at least two unique peptides. Quantitative data are listed in the supplementary proteomics data spreadsheet. The mass spectrometry proteomics data have been deposited to the ProteomeXchange Consortium via the PRIDE partner repository with the dataset identifier PXD052768: Perez-Riverol Y, Bai J, Bandla C, Hewapathirana S, García-Seisdedos D, Kamatchinathan S, Kundu D, Prakash A, Frericks-Zipper A, Eisenacher M, Walzer M, Wang S, Brazma A, Vizcaíno JA (2022). The PRIDE database resources in 2022: A Hub for mass spectrometry-based proteomics evidences. Nucleic Acids Res 50(D1):D543-D552 (PubMed ID: 34723319).

Decomposition Kinetics Studies Solutions of the probe compound (50 µl 2 µM in isopropanol) prepared in triplicate in a 96-well plate were exposed to UV light (365 nm, 4 ºC) for different periods of time. After the irradiation was complete, a solution of *p*-nitroaniline (50 µl, 400 µM) was added to each well. The contents of each well were transferred to vials for mass spectrometric analysis using a ThermoFisher UltiMate 3000 HPLC system with a Syncronis C18 column (Thermo Scientific , 100 mm × 2.1 mm, 3 µm) and coupled to a single quadrupole mass spectrometer (ThermoFisher ISQ EC). The analytical method was carried out with a flow rate of 0.5 ml/min, 40 °C column temperature and a gradient consisting of equilibration with 95 % buffer A (0.1 % formic acid in H_2O) and 5 % buffer B (0.1 % formic acid in MeCN) for 4 min, before injection of the sample. The proportion of buffer B was then increased to 95 % over 8

min, followed by isocratic elution for a further 4 min. The eluant was analyzed by mass spectrometry, selectively monitoring the $[M+H]^+$ signals of the probe compound and *p*-nitroaniline. The concentration of the probe compound in each sample was calculated by comparing the ratio of the probe / p-nitroaniline signals to a standard curve.

References

(1) Parker, C. G.; Galmozzi, A.; Wang, Y.; Correia, B. E.; Sasaki, K.; Joslyn, C. M.; Kim, A. S.; Cavallaro, C. L.; Lawrence, R. M.; Johnson, S. R.; Narvaiza, I.; Saez, E.; Cravatt, B. F. Ligand and Target Discovery by Fragment-Based Screening in Human Cells. *Cell* **2017**, *168*, 527-541.e529.
(2) Wang, Y.; Dix, M. M.; Bianco, G.; Remsberg, J. R.; Lee, H.-Y.; Kalocsay, M.; Gygi, S. P.; Forli, S.; Vite, G.; Lawrence, R. M.; Parker, C. G.; Cravatt, B. F. Expedited mapping of the ligandable proteome using fully functionalized enantiomeric probe pairs. *Nat. Chem.* **2019**, *11*, 1113-1123.
(3) Conway, L. P.; Jadhav, A. M.; Homan, R. A.; Li, W.; Rubiano, J. S.; Hawkins, R.; Lawrence, R. M.; Parker, C. G. Evaluation of fully-functionalized diazirine tags for chemical proteomic applications. *Chem. Sci.* **2021**, *12*, 7839-7847.
(4) Guan, H.-P.; Qiu, Y.-L.; Ksebati, M. B.; Kern, E. R.; Zemlicka, J. Synthesis of phosphonate derivatives of methylenecyclopropane nucleoside analogues by alkylation–elimination method and unusual opening of cyclopropane ring. *Tetrahedron* **2002**, *58*, 6047-6059.
(5) Poudel, P. P.; Arimitsu, K.; Yamamoto, K. Self-assembled ion-pair organocatalysis – asymmetric Baeyer–Villiger oxidation mediated by flavinium–cinchona alkaloid dimer. *Chem. Commun.* **2016**, *52*, 4163-4166.
(6) Panish, R.; Chintala, S. R.; Boruta, D. T.; Fang, Y.; Taylor, M. T.; Fox, J. M. Enantioselective Synthesis of Cyclobutanes via Sequential Rh-catalyzed Bicyclobutanation/Cu-catalyzed Homoconjugate Addition. *J. Am. Chem. Soc.* **2013**, *135*, 9283-9286.
(7) Primozic, J. J.; Ilgen, J.; Maibach, P.; Brauser, M.; Kind, J.; Thiele, C. M. Pd-Catalyzed Asymmetric Allylic Alkylation of Cyclobutenes: From Double Inversion to Double Retention. *J. Am. Chem. Soc.* **2023**, *145*, 15912-15923.
(8) Chen, Y.; Coussanes, G.; Souris, C.; Aillard, P.; Kaldre, D.; Runggatscher, K.; Kubicek, S.; Di Mauro, G.; Maryasin, B.; Maulide, N. A Domino 10-Step Total Synthesis of FR252921 and Its Analogues, Complex Macrocyclic Immunosuppressants. *J. Am. Chem. Soc.* **2019**, *141*, 13772-13777.
(9) Ahad, A. M.; Jensen, S. M.; Jewett, J. C. A Traceless Staudinger Reagent To Deliver Diazirines. *Organic Letters* **2013**, *15*, 5060-5063.
(10) Dobbs, A. P.; Guesne, S. J.; Parker, R. J.; Skidmore, J.; Stephenson, R. A.; Hursthouse, M. B. A detailed investigation of the aza-Prins reaction. *Org Biomol Chem* **2010**, *8*, 1064-1080.
(11) Wang, J.; Chen, J.; Kee, C. W.; Tan, C. H. Enantiodivergent and gamma-selective asymmetric allylic amination. *Angew Chem Int Ed Engl* **2012**, *51*, 2382-2386.
(12) O'Brien, J. G. K.; Jemas, A.; Asare-Okai, P. N.; am Ende, C. W.; Fox, J. M. Probing the Mechanism of Photoaffinity Labeling by Dialkyldiazirines through Bioorthogonal Capture of Diazoalkanes. *Organic Letters* **2020**, *22*, 9415-9420.
(13) Stevens, C.; De Kimpe, N. A New Entry into 2-Azabicyclo[2.1.1]hexanes via 3-(Chloromethyl)cyclobutanone. *J. Org. Chem.* **1996**, *61*, 2174-2178.

(14) Greb, A.; Poh, J.-S.; Greed, S.; Battilocchio, C.; Pasau, P.; Blakemore, D. C.; Ley, S. V. A Versatile Route to Unstable Diazo Compounds via Oxadiazolines and their Use in Aryl–Alkyl Cross-Coupling Reactions. *Angew. Chem. Int. Edit.* **2017**, *56*, 16602-16605.

^{1}H NMR spectrum of **26** (400 MHz, $CDCl_3$)

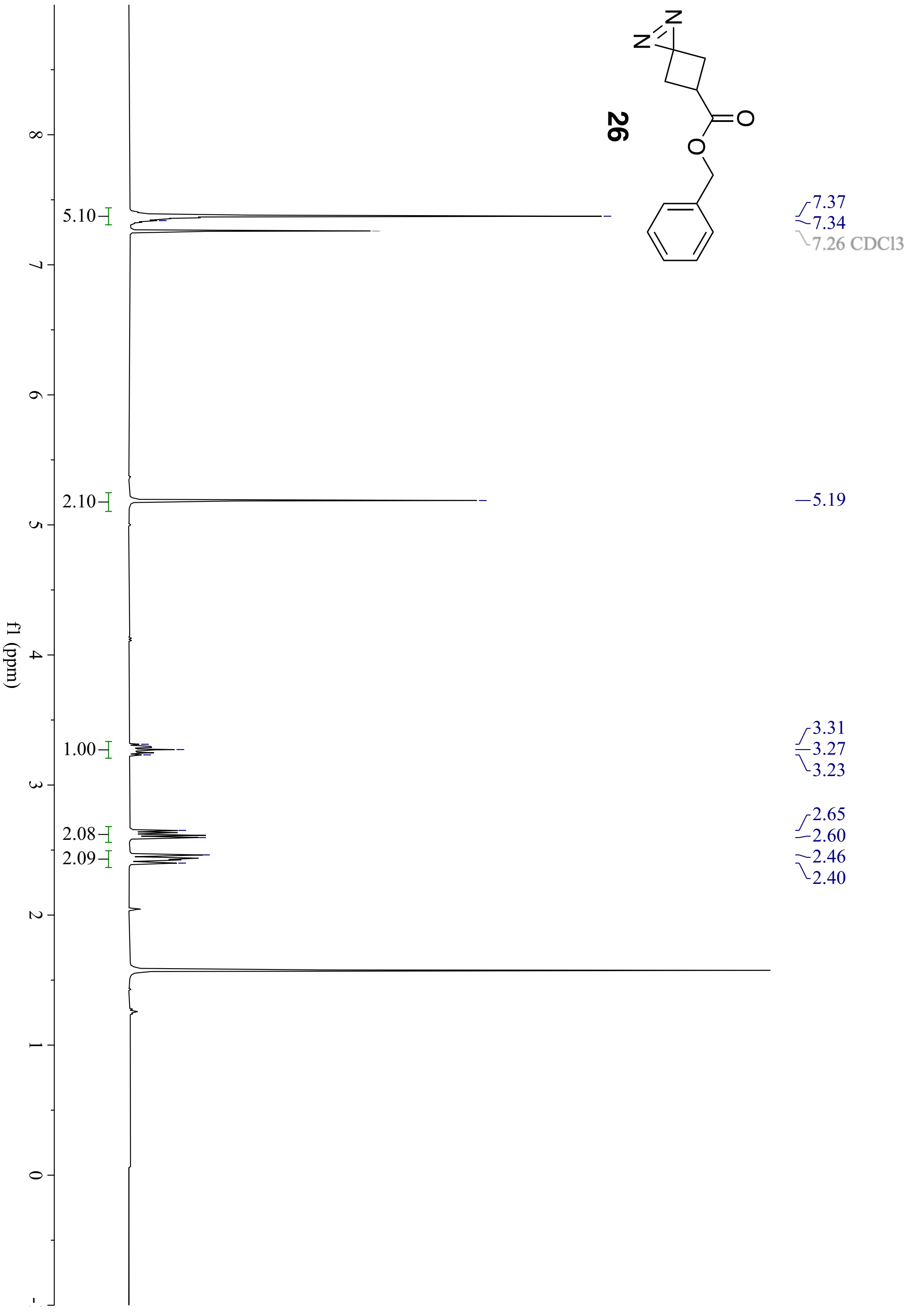

^{13}C NMR spectrum of **26** (101 MHz, $CDCl_3$)

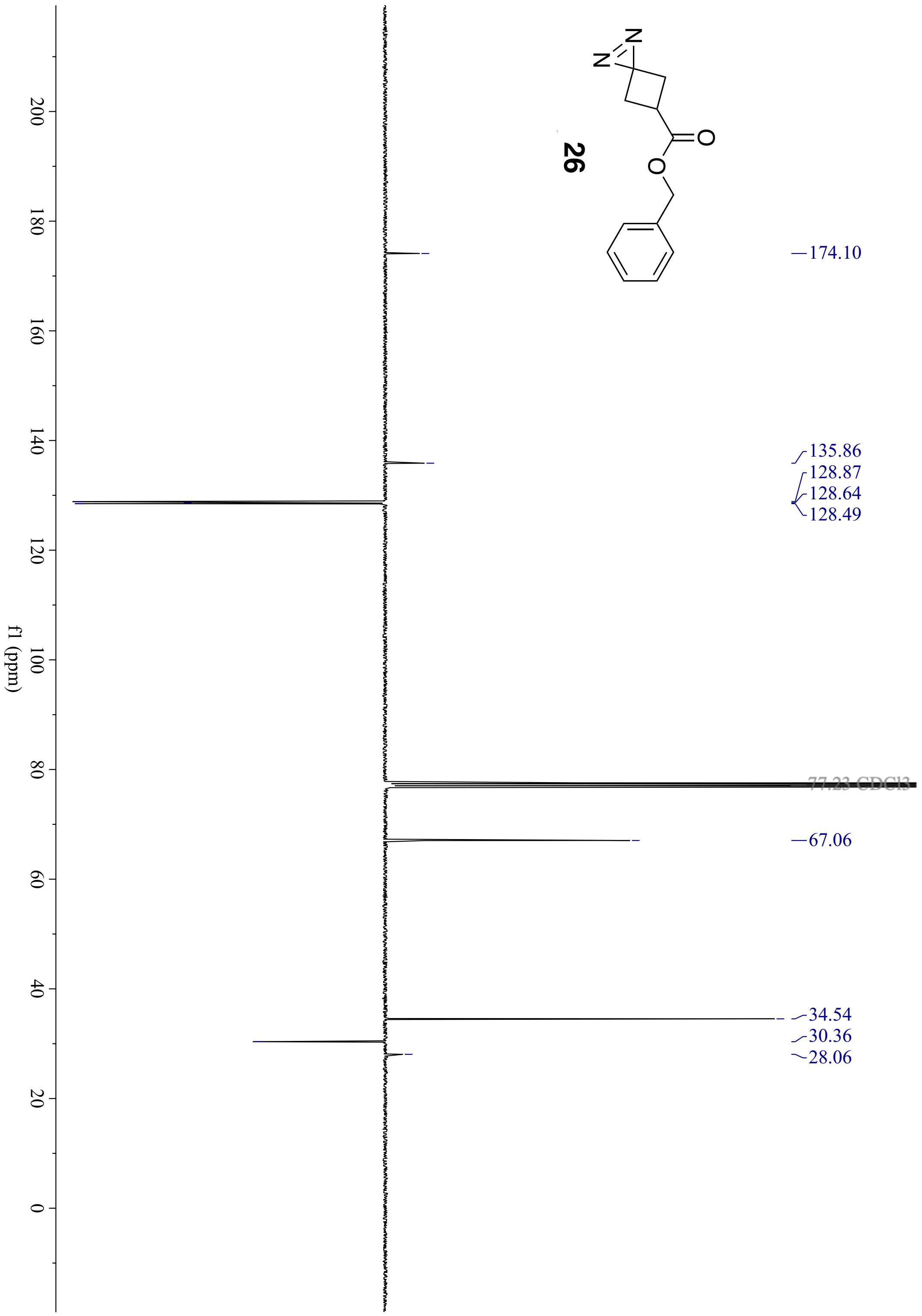

^{1}H NMR spectrum (400 MHz, $CDCl_3$)

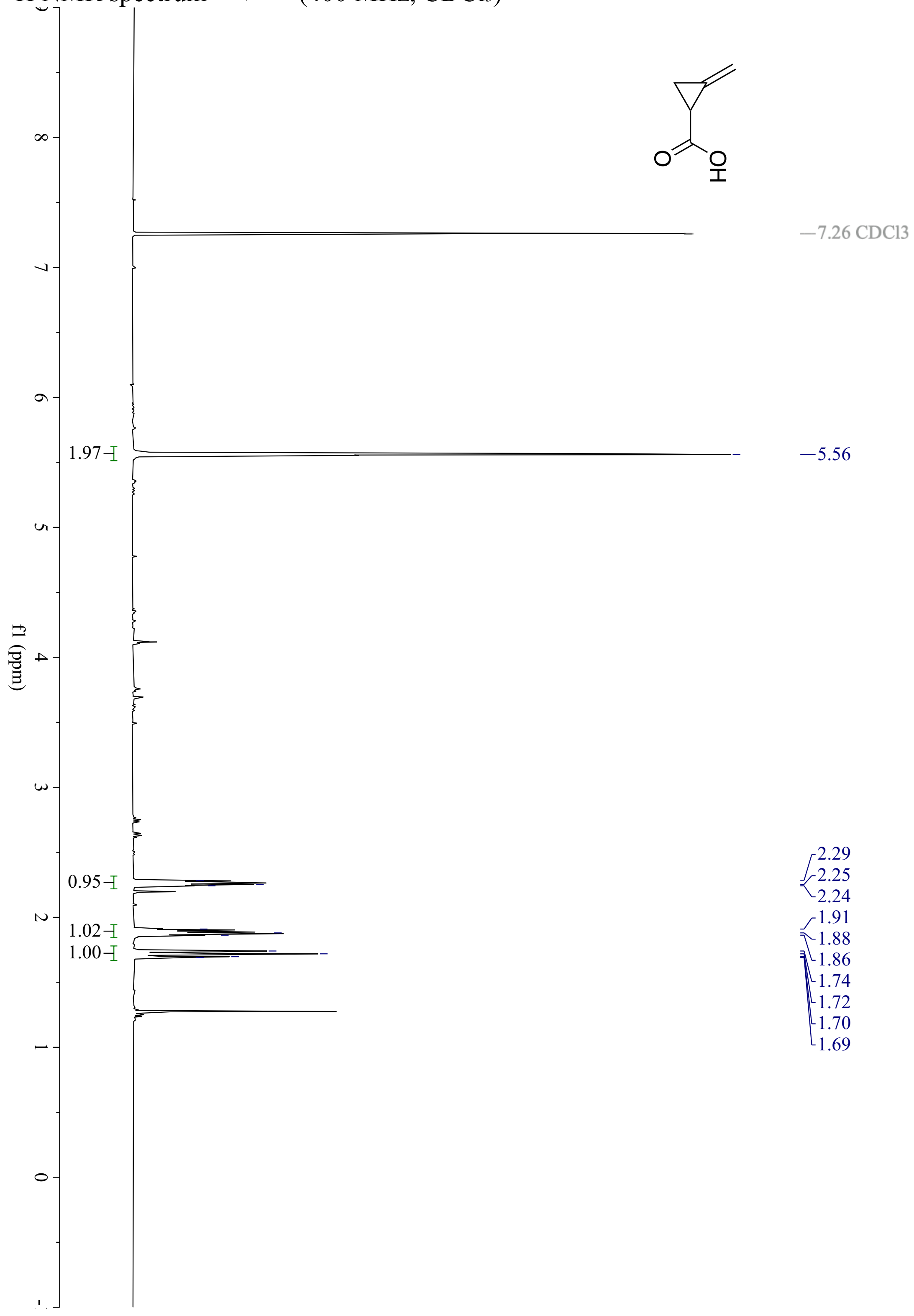

^{13}C NMR spectrum (101 MHz, $CDCl_3$)

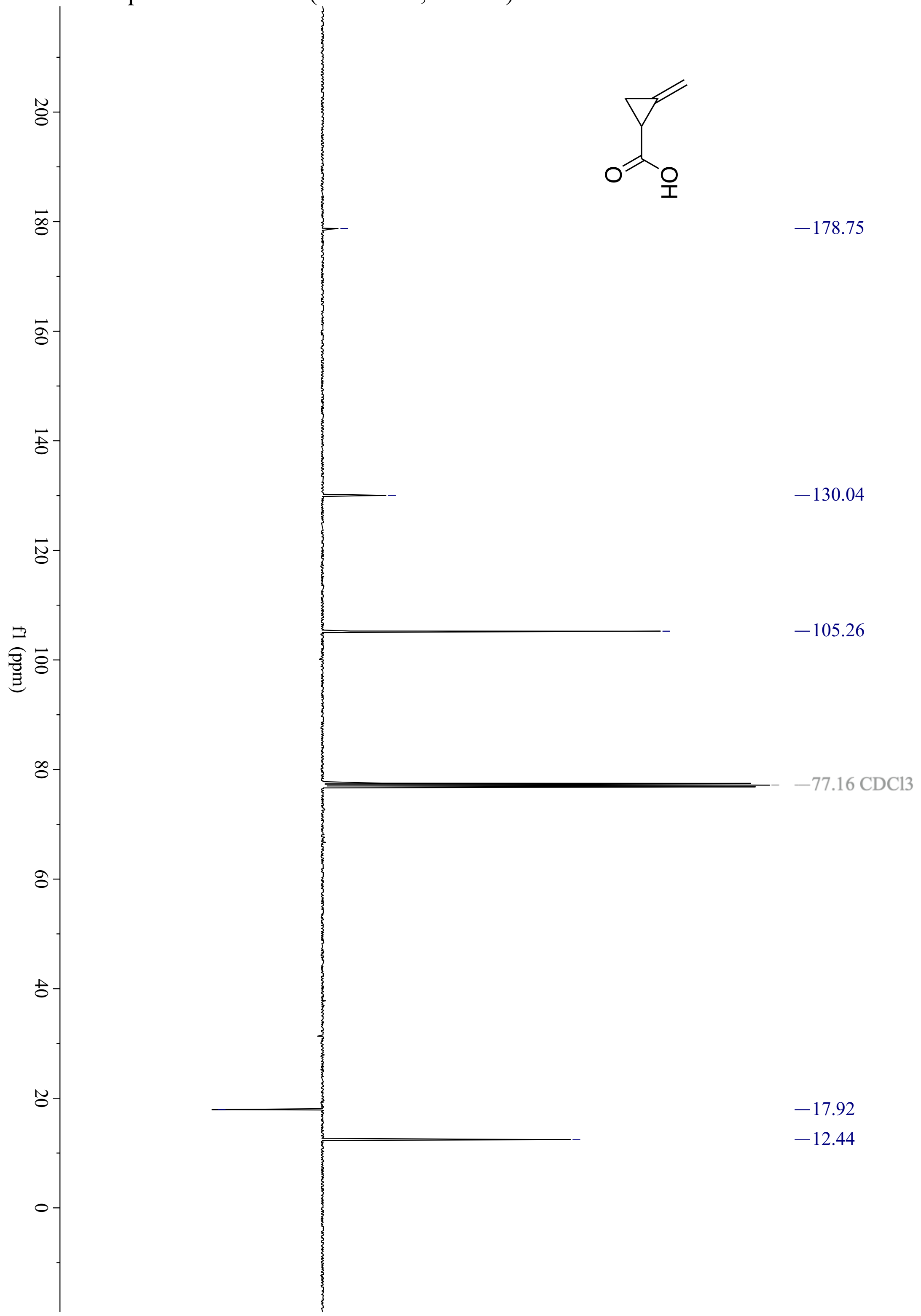

^{1}H NMR spectrum of **5** (600 MHz, CD_3OD)

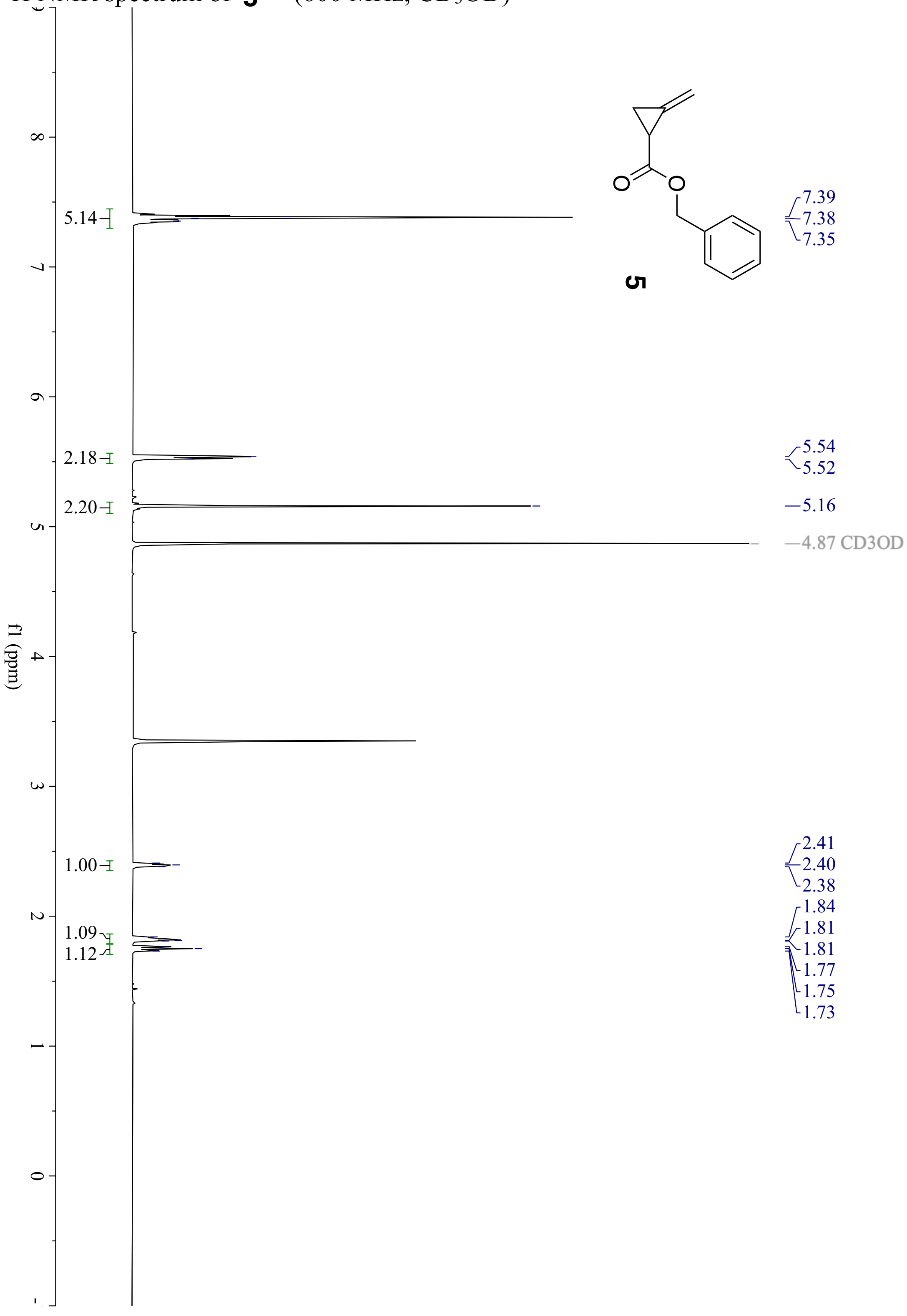

^{13}C NMR spectrum of **5** (101 MHz, $CDCl_3$)

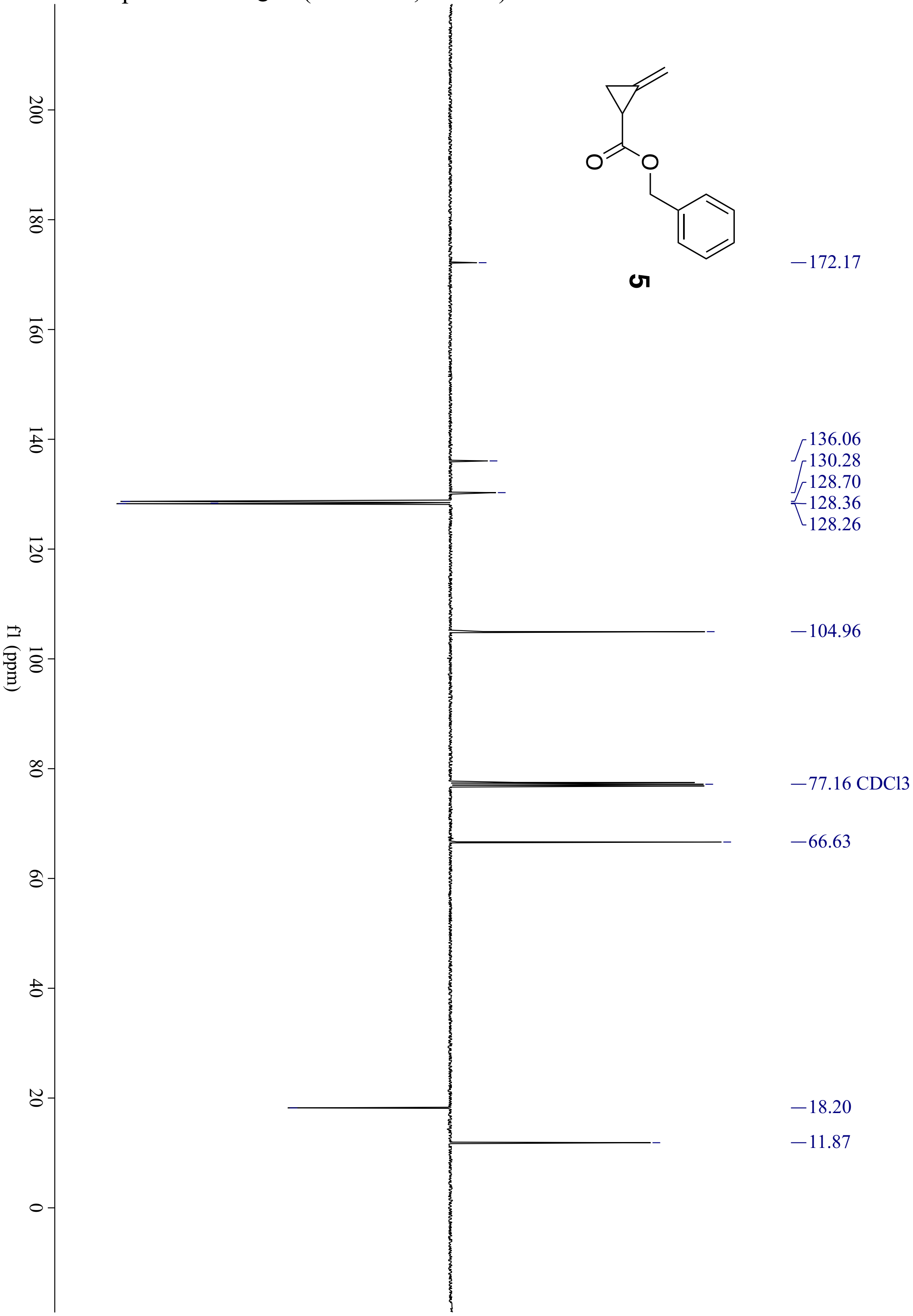

^{1}H NMR spectrum (400 MHz, $CDCl_3$)

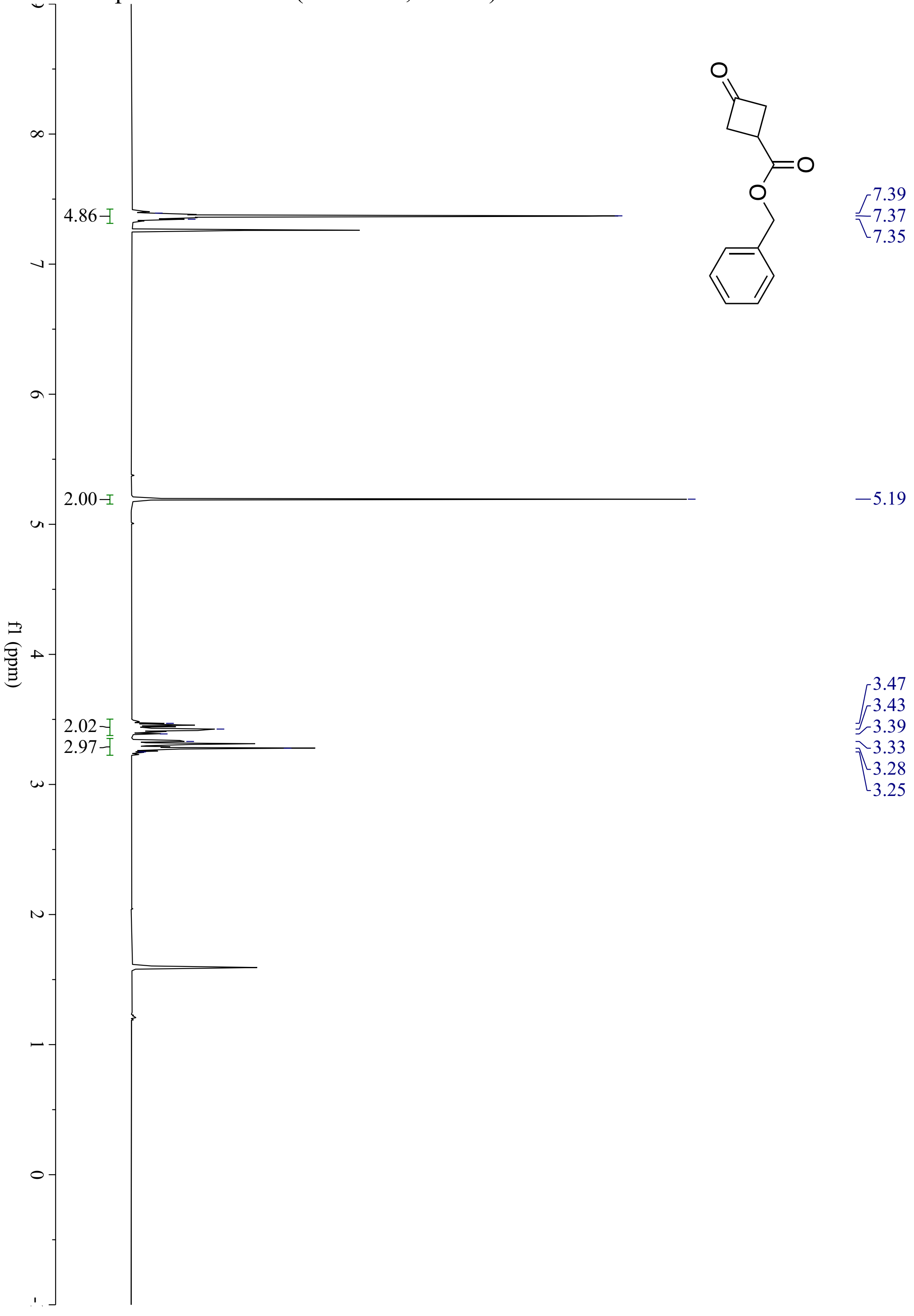

^{13}C NMR spectrum (101 MHz, $CDCl_3$)

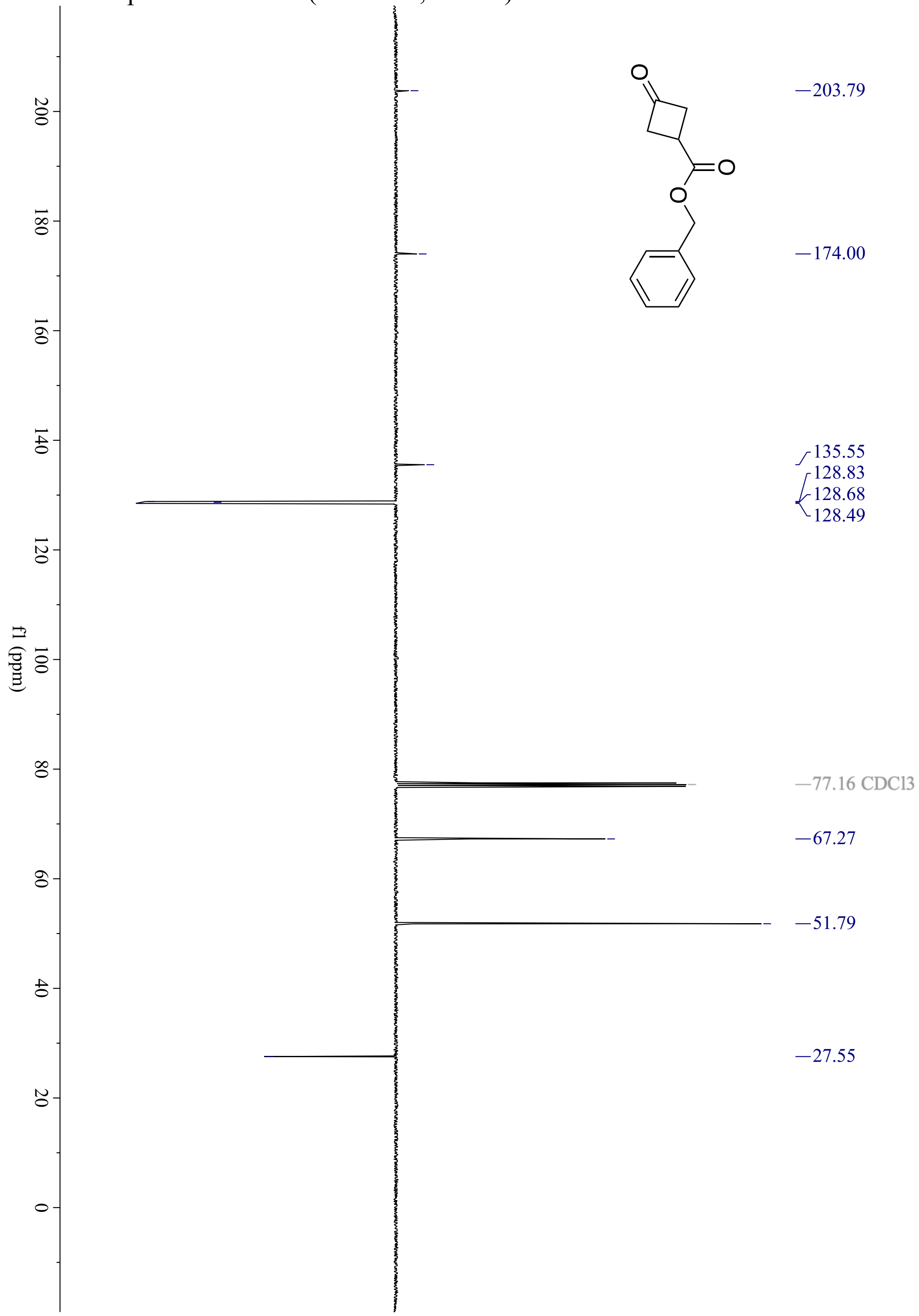

^{1}H NMR spectrum (400 MHz, $CDCl_3$)

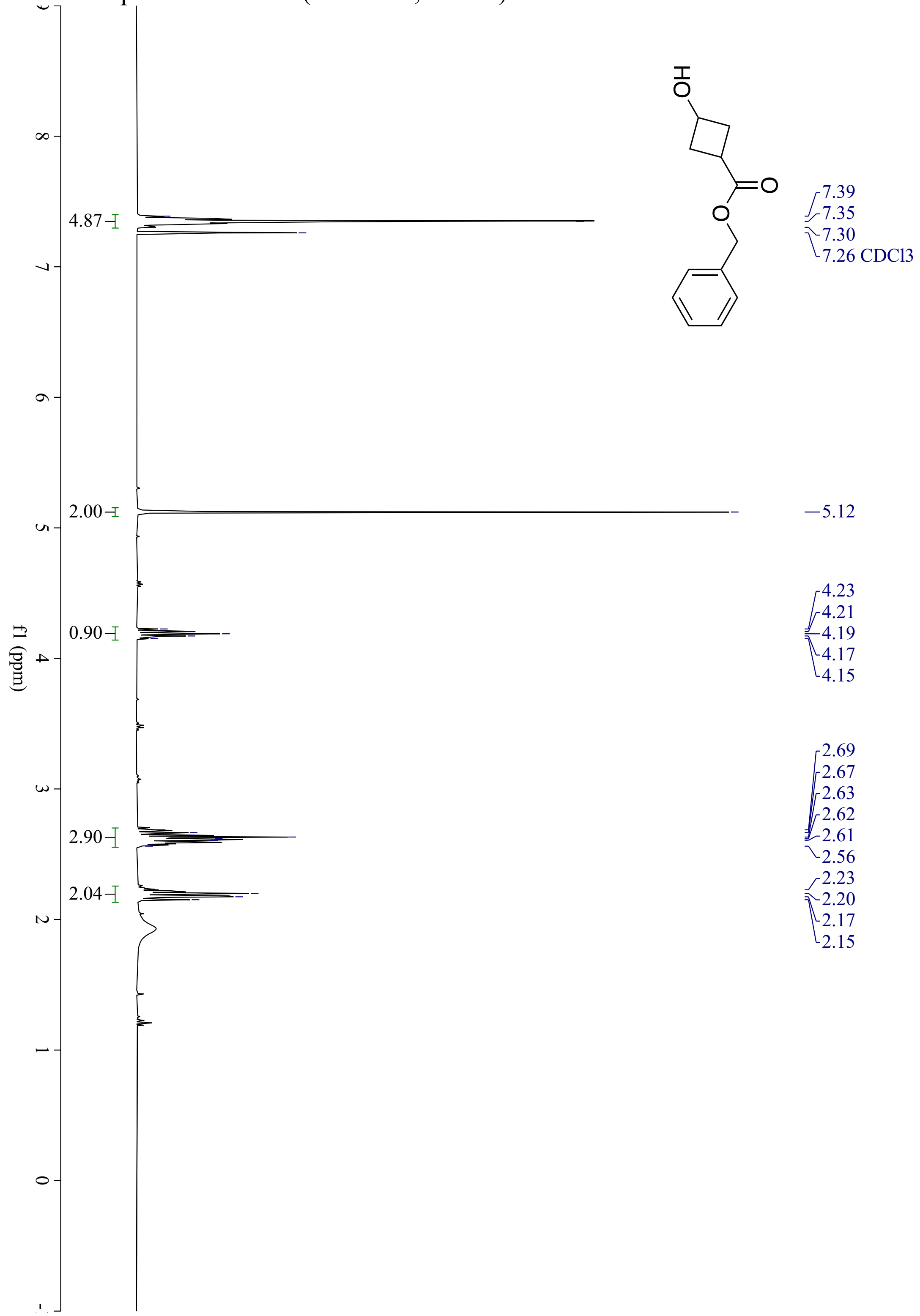

^{13}C NMR spectrum (101 MHz, $CDCl_3$)

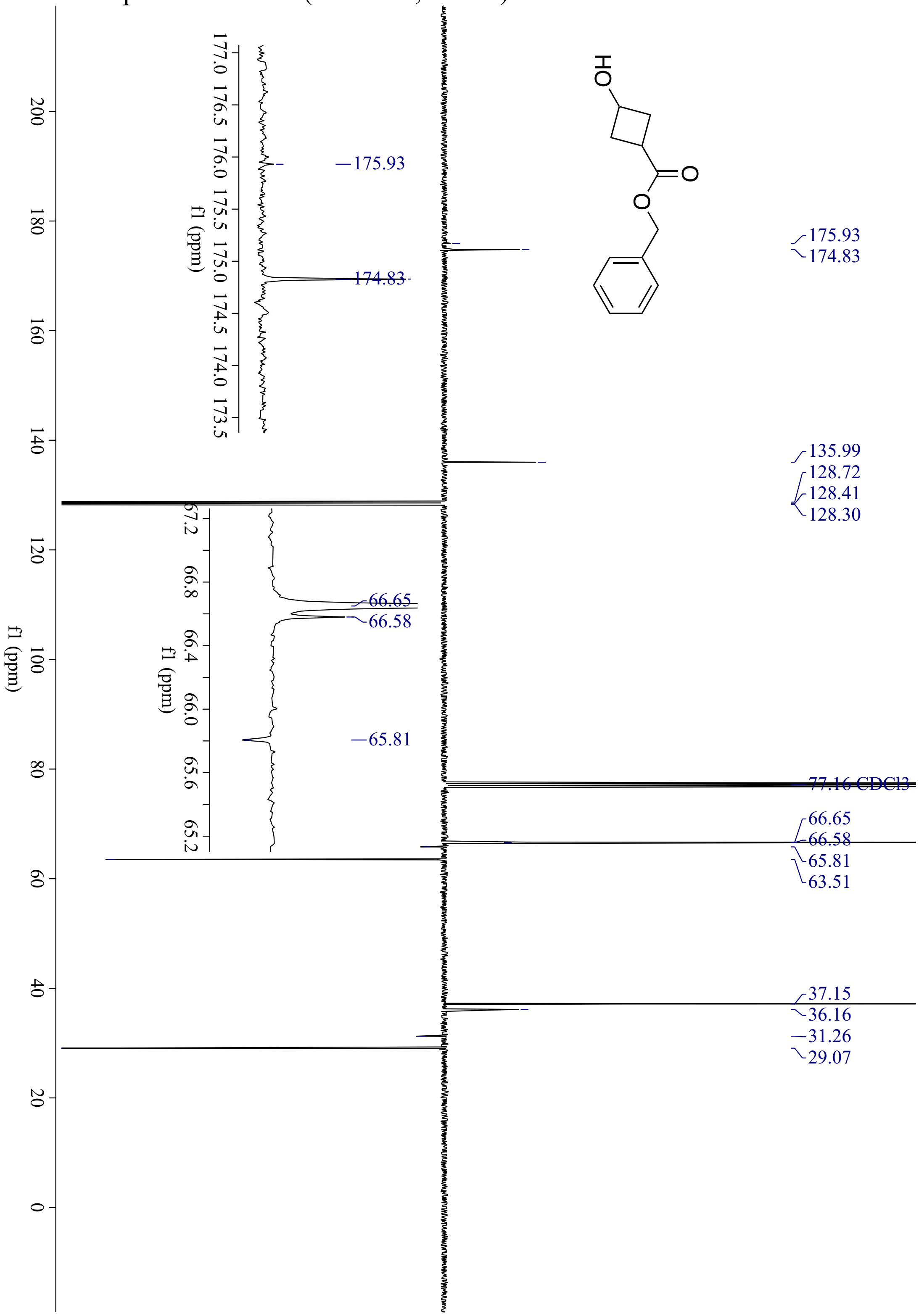

^{1}H NMR spectrum of **10** (600 MHz, CD_3OD)

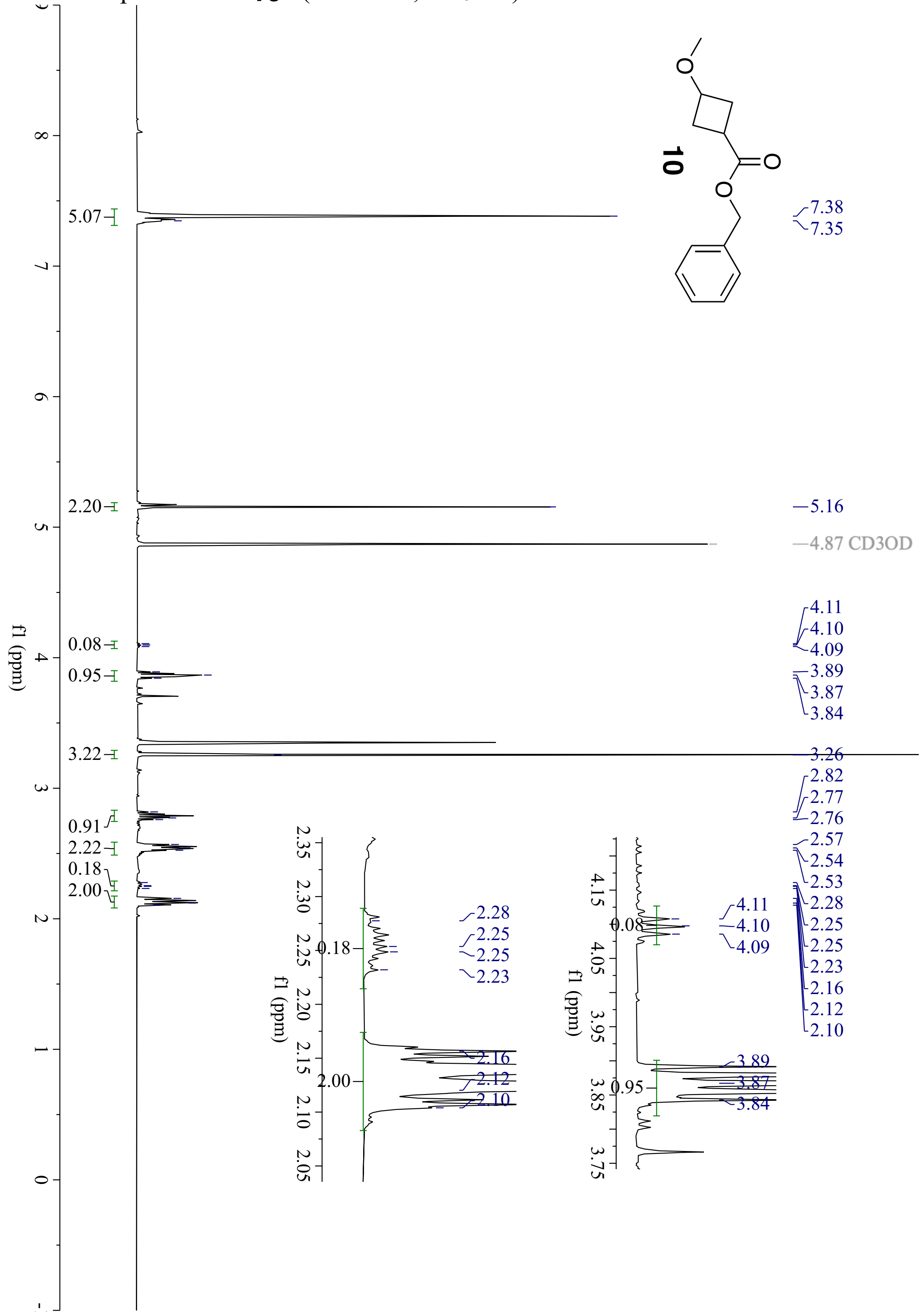

^{13}C NMR spectrum of **10** (101 MHz, $CDCl_3$)

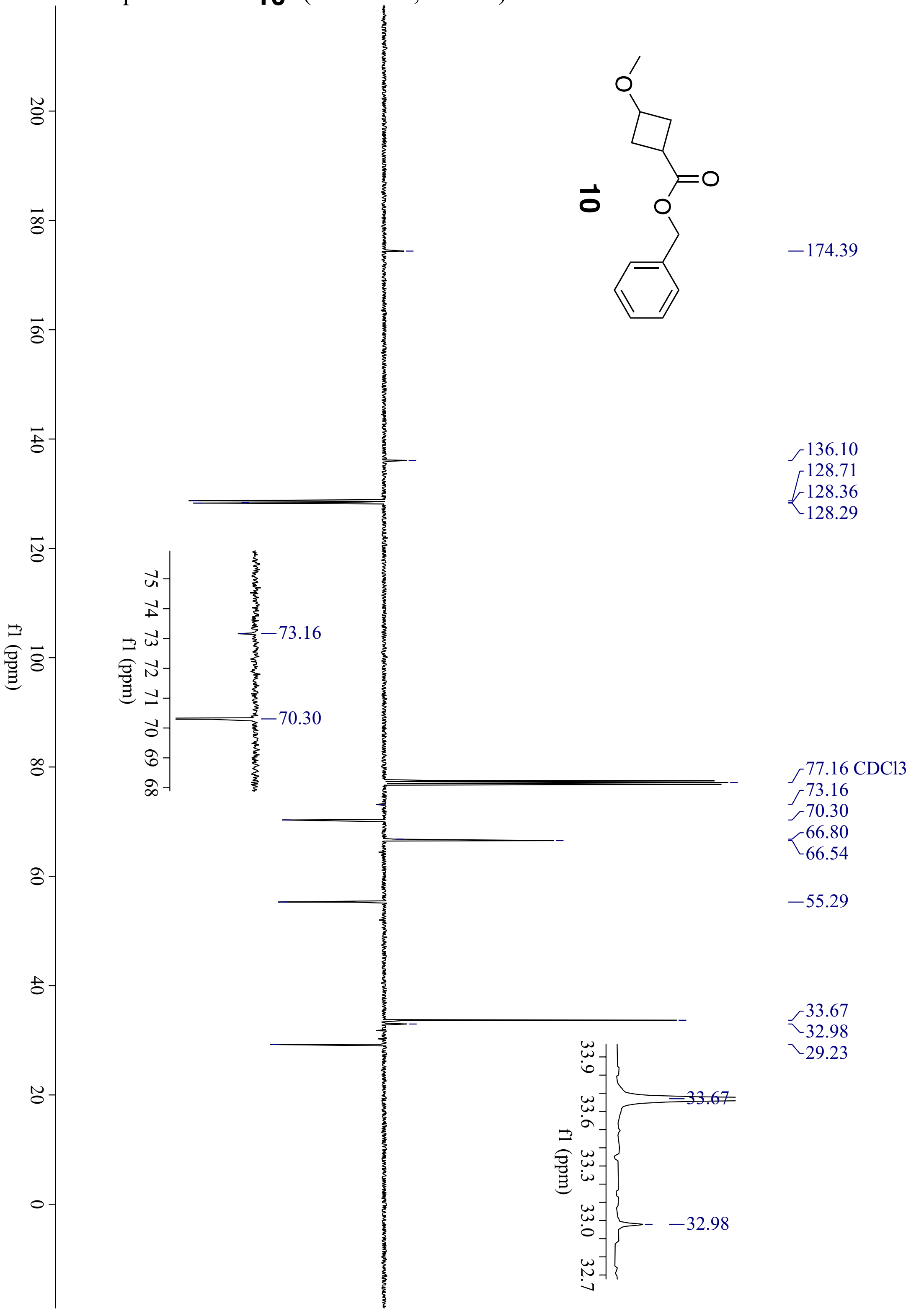

^{1}H NMR spectrum (400 MHz, $CDCl_3$)

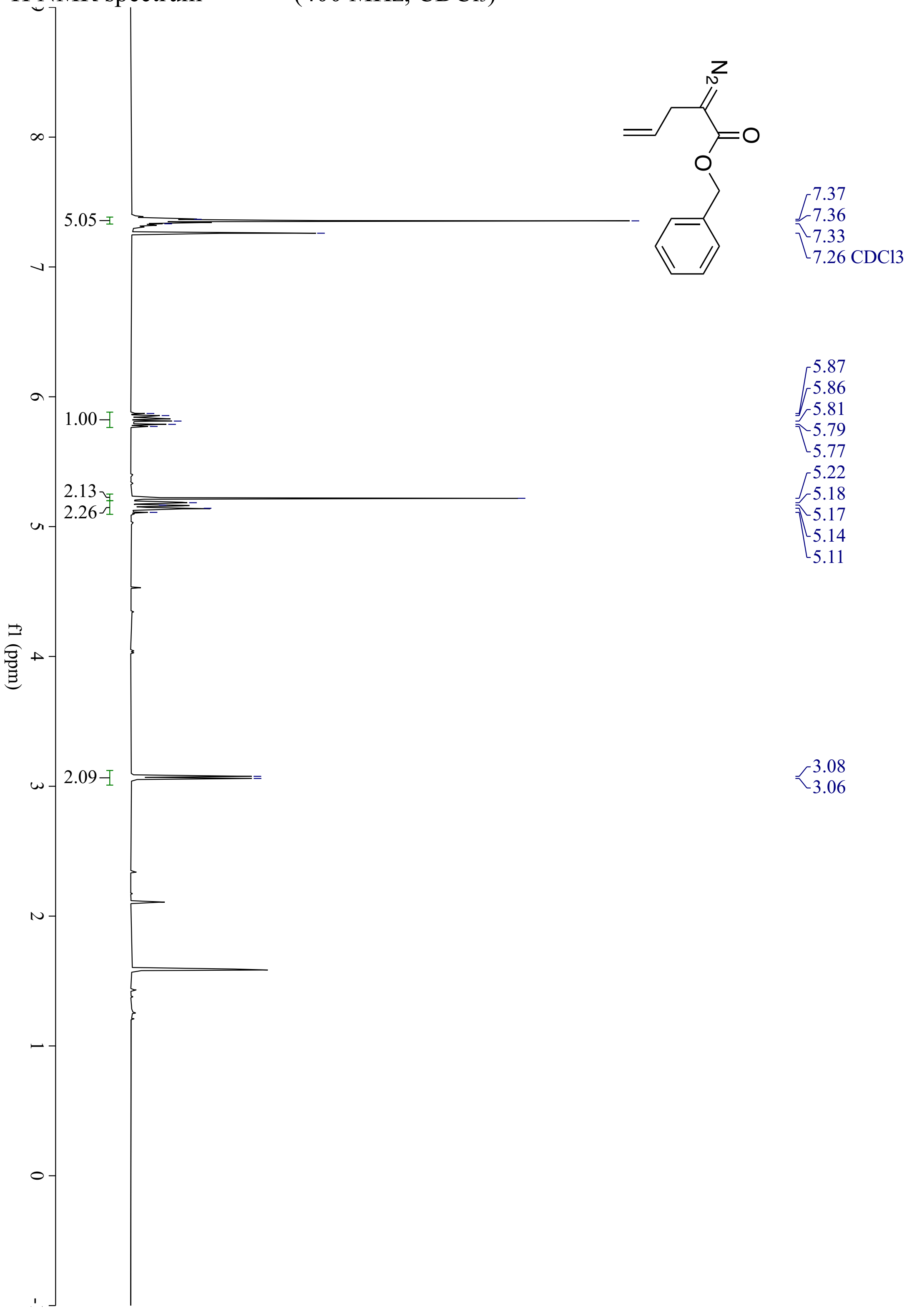

^{13}C NMR spectrum (101 MHz, $CDCl_3$)

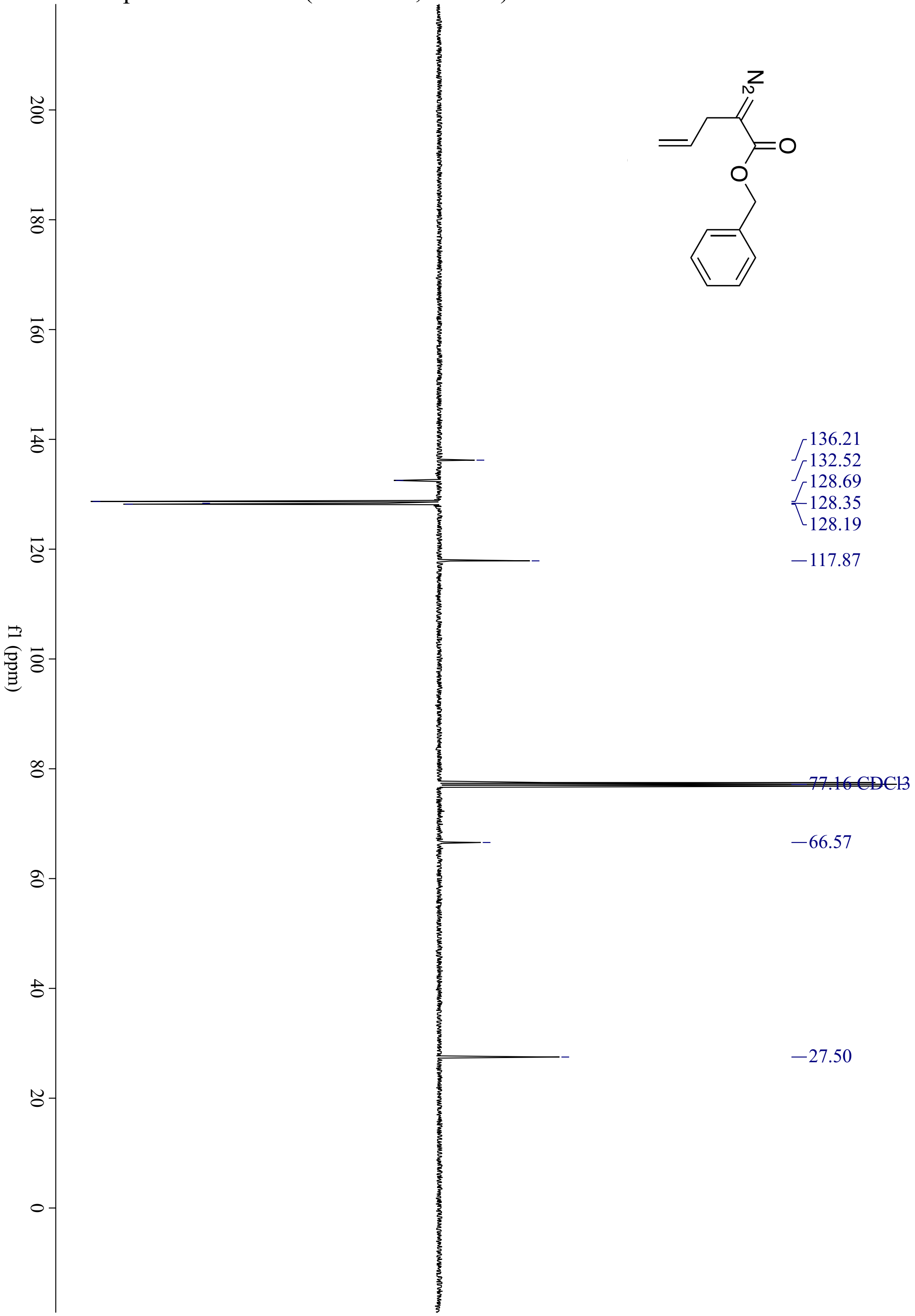

^1H NMR spectrum of **9** (600 MHz, CD_3OD)

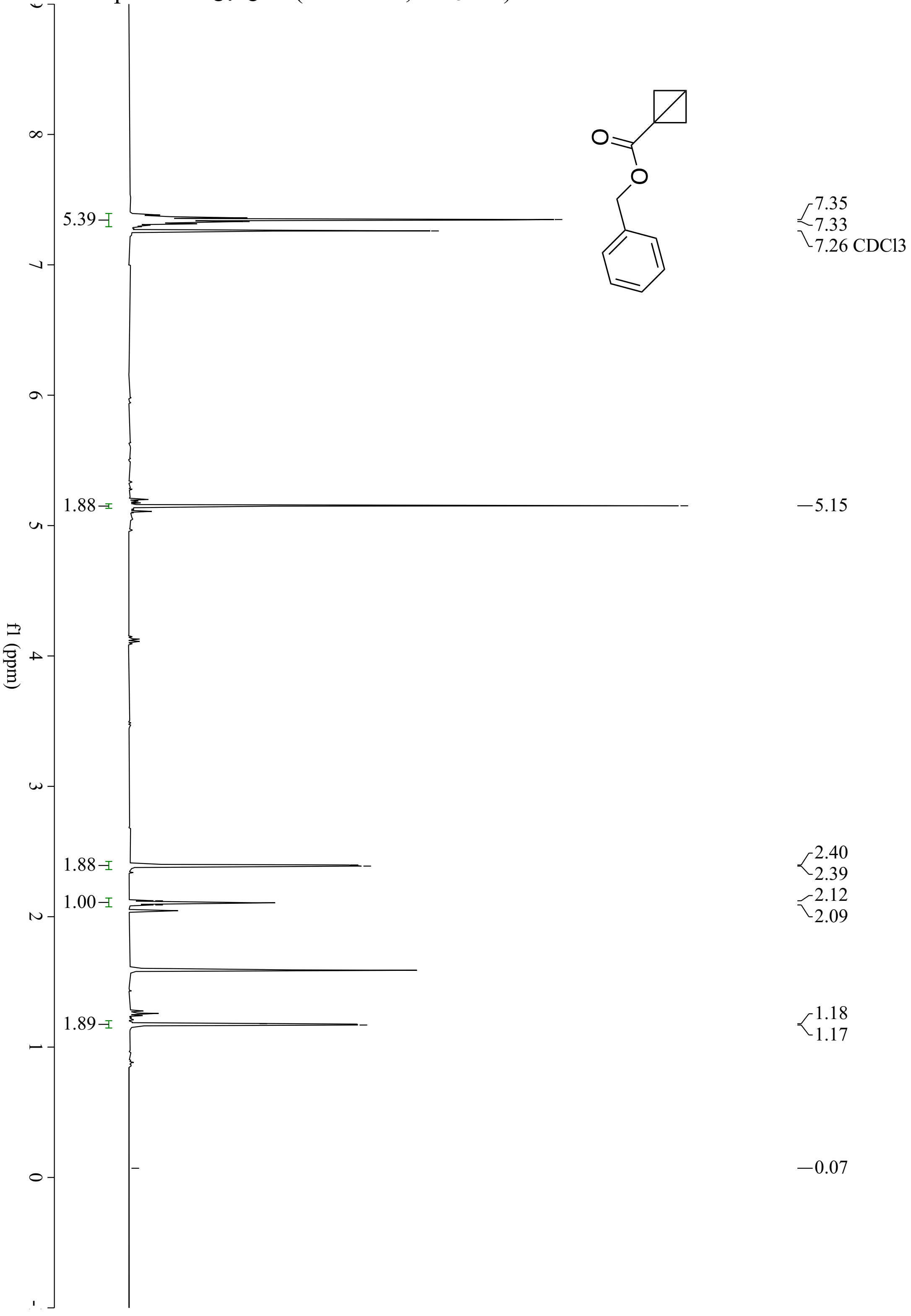

^{13}C NMR spectrum of **9** (101 MHz, $CDCl_3$)

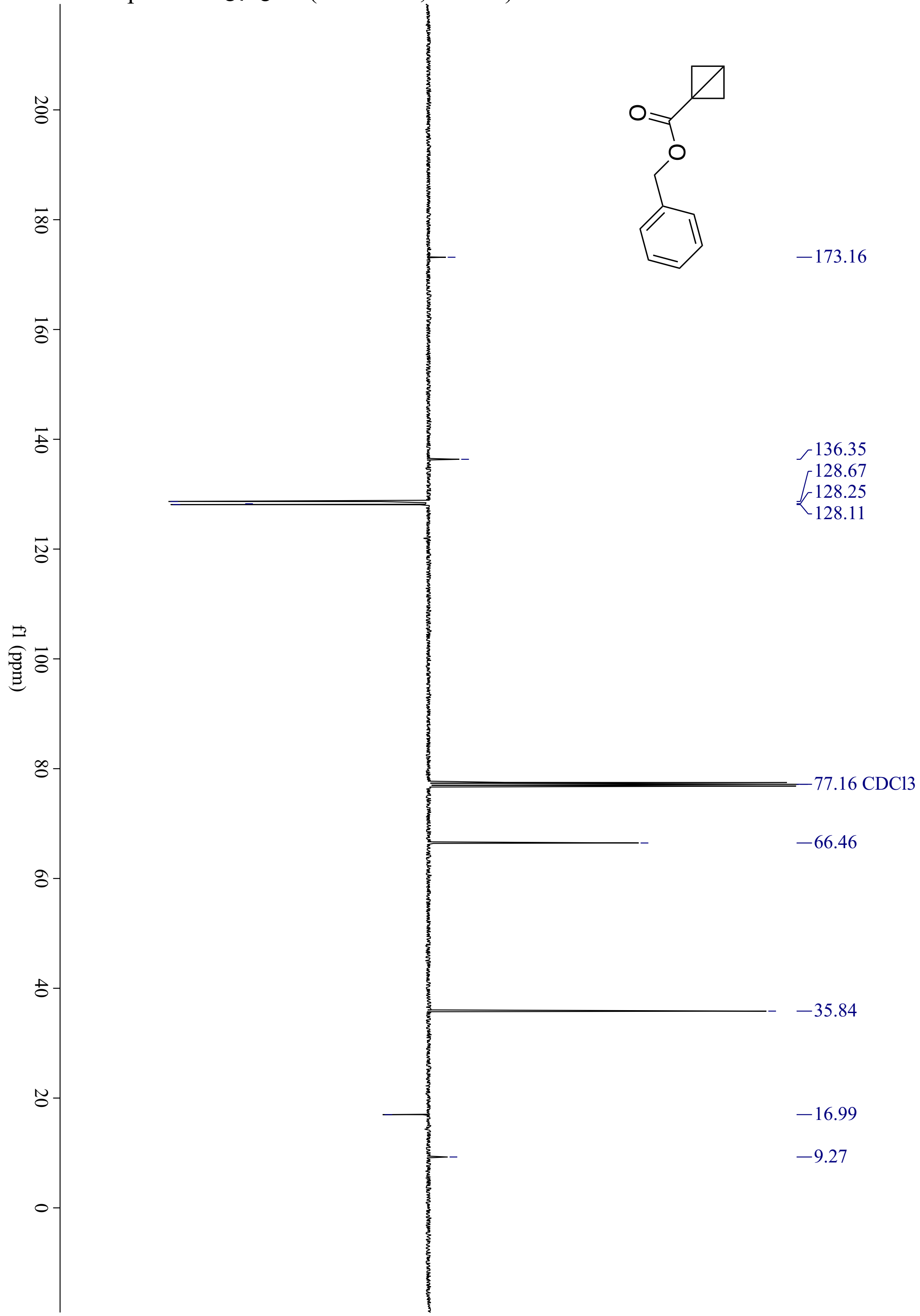

^{1}H NMR of cyclobut-2-ene carboxylic acid (600 MHz, $CDCl_3$)

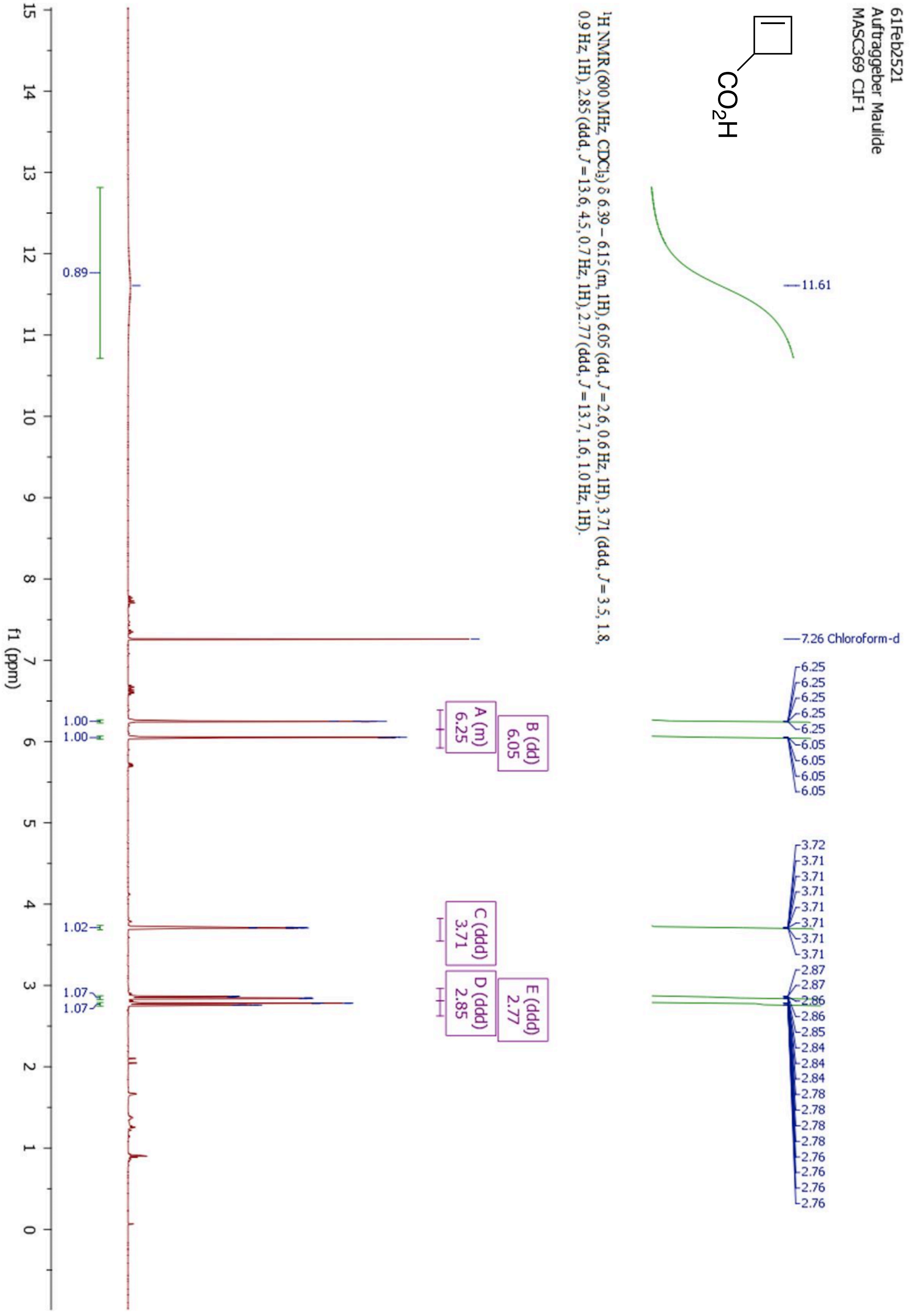

^{13}C NMR of cyclobut-2-ene carboxylic acid (125 MHz, $CDCl_3$)

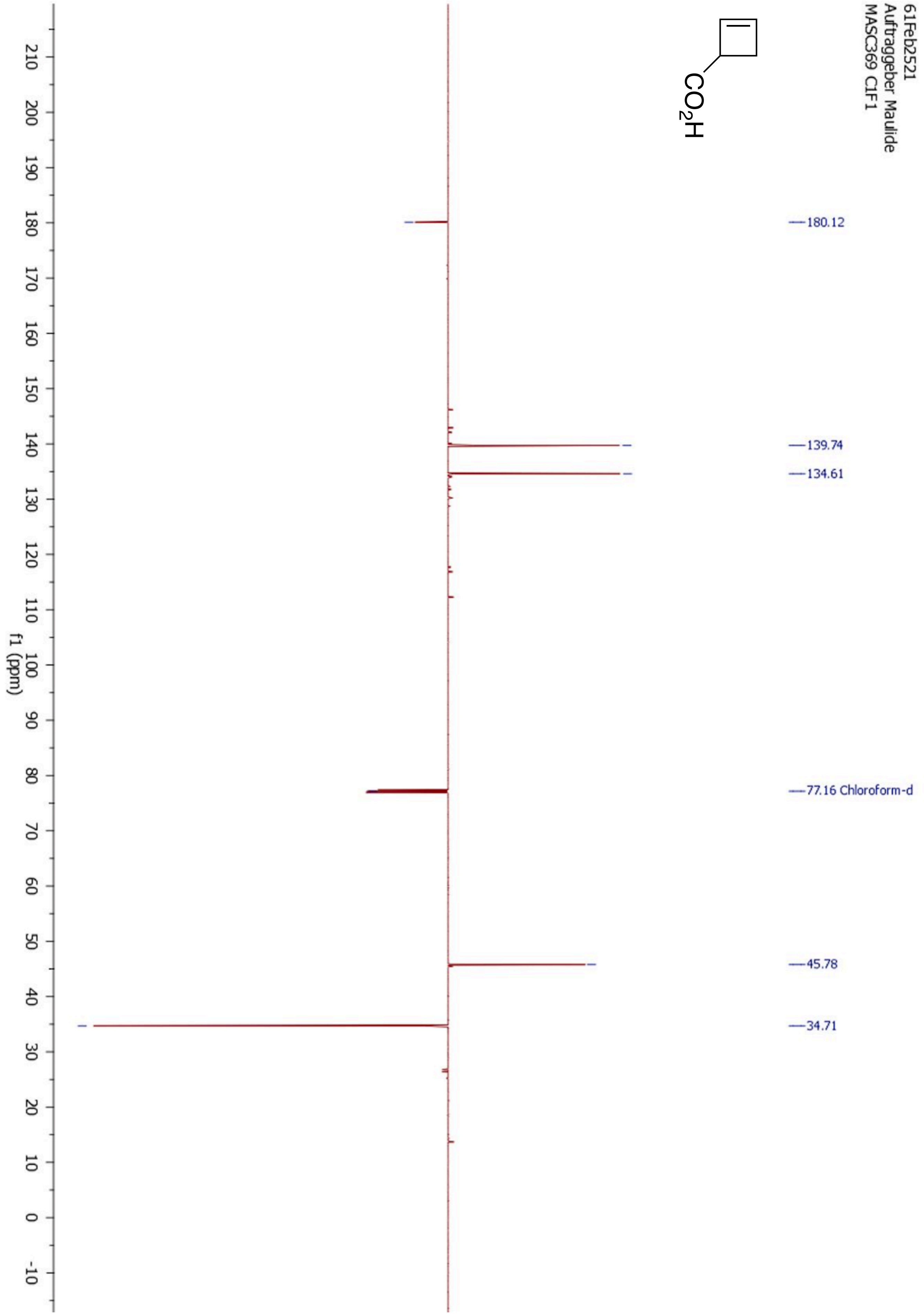

^{1}H NMR spectrum of **7** (600 MHz, CD_3OD)

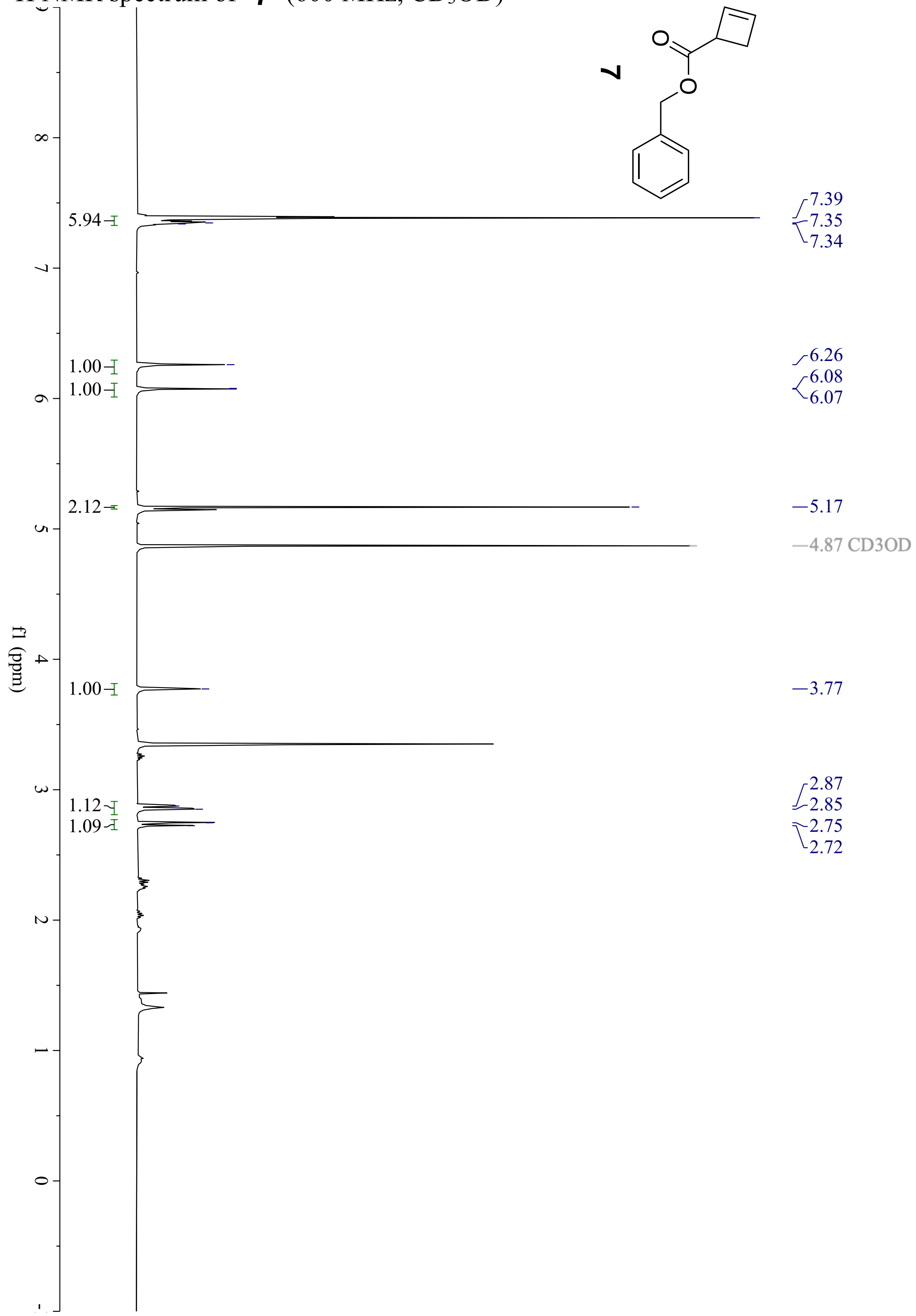

^{13}C NMR spectrum of **7** (150 MHz, CD_3OD)

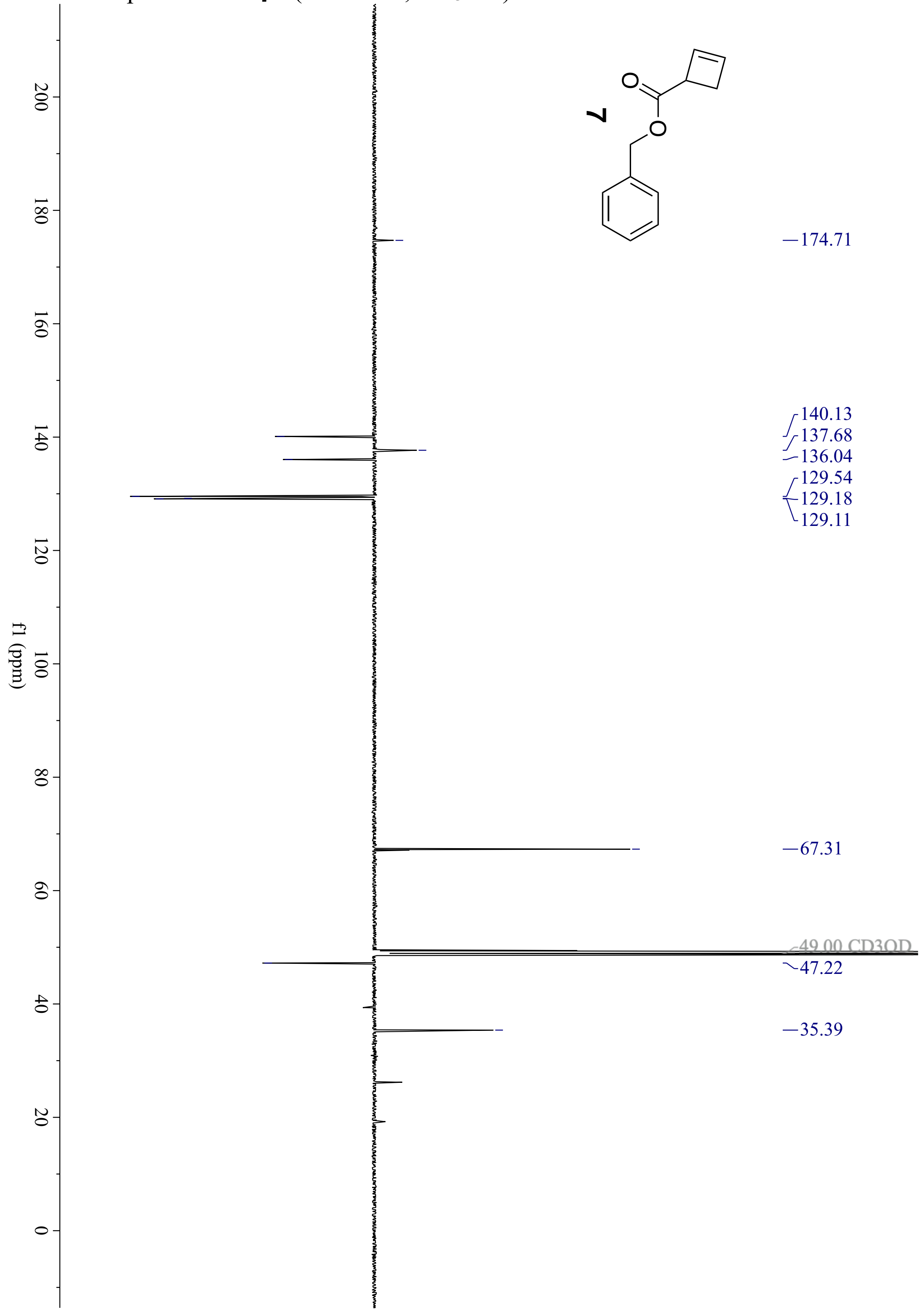

^{1}H NMR spectrum of **27** (400 MHz, CD_3OD)

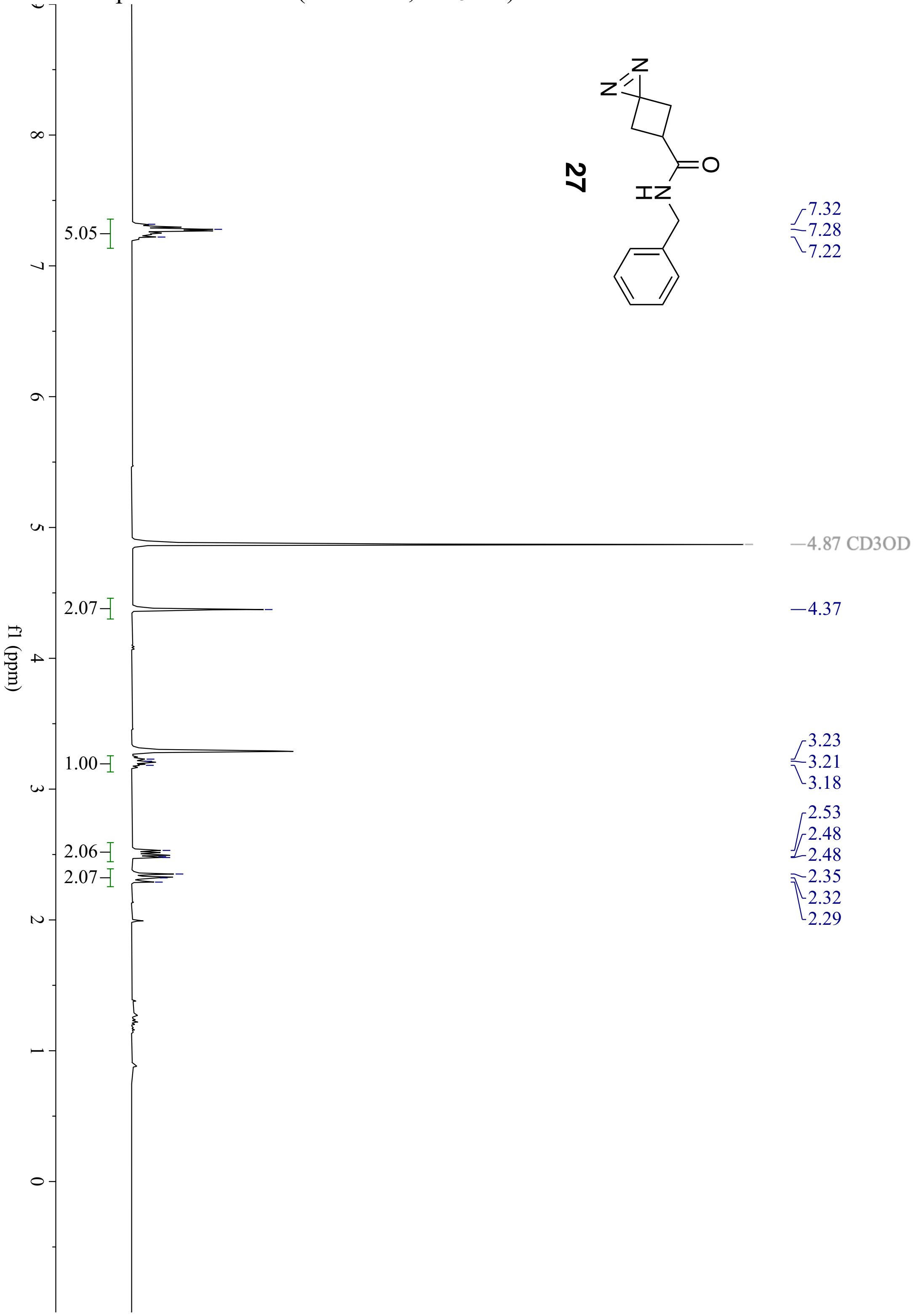

^{13}C NMR spectrum of **27** (101 MHz, CD_3OD)

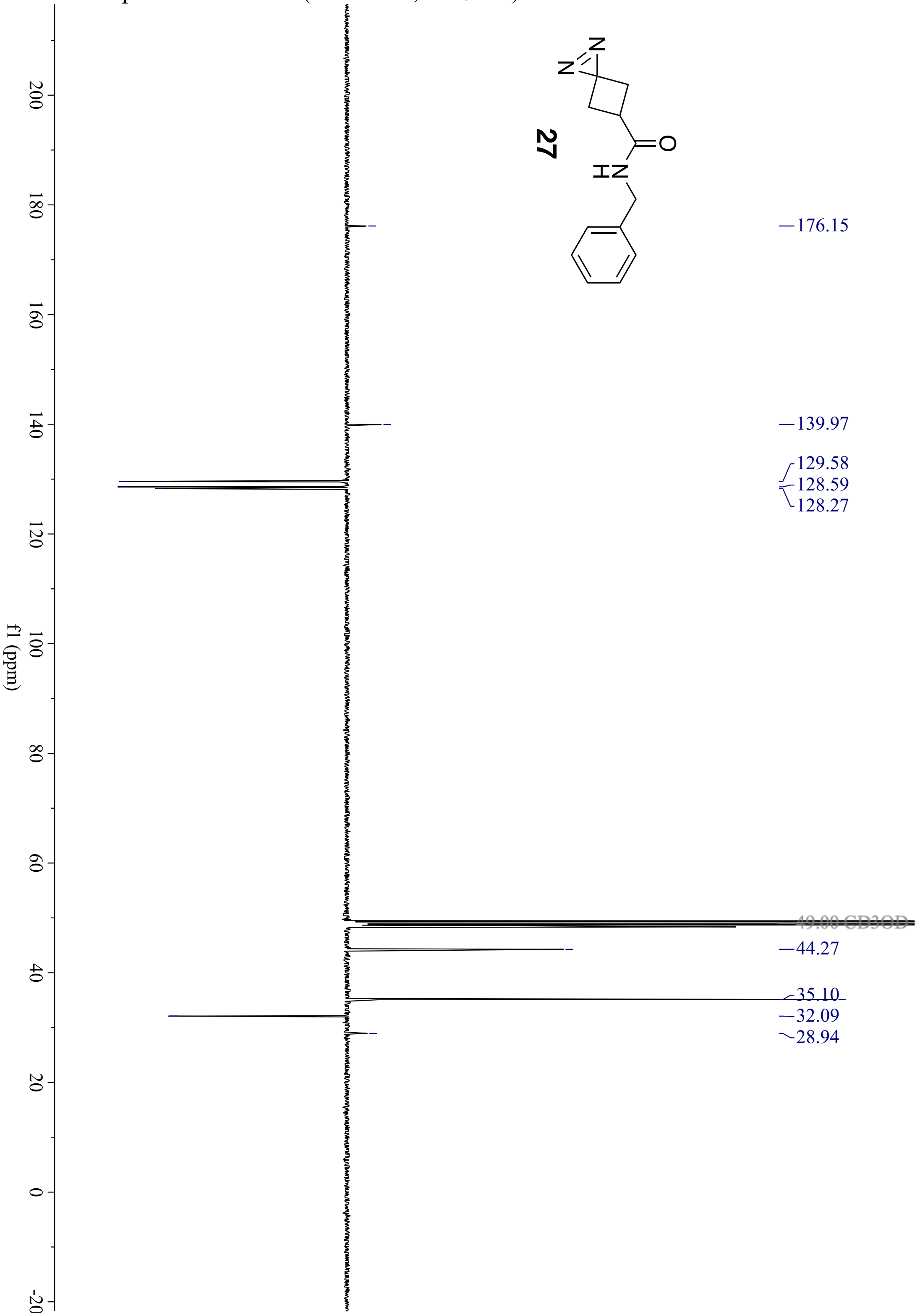

^{1}H NMR spectrum of **8** (600 MHz, CD_3OD)

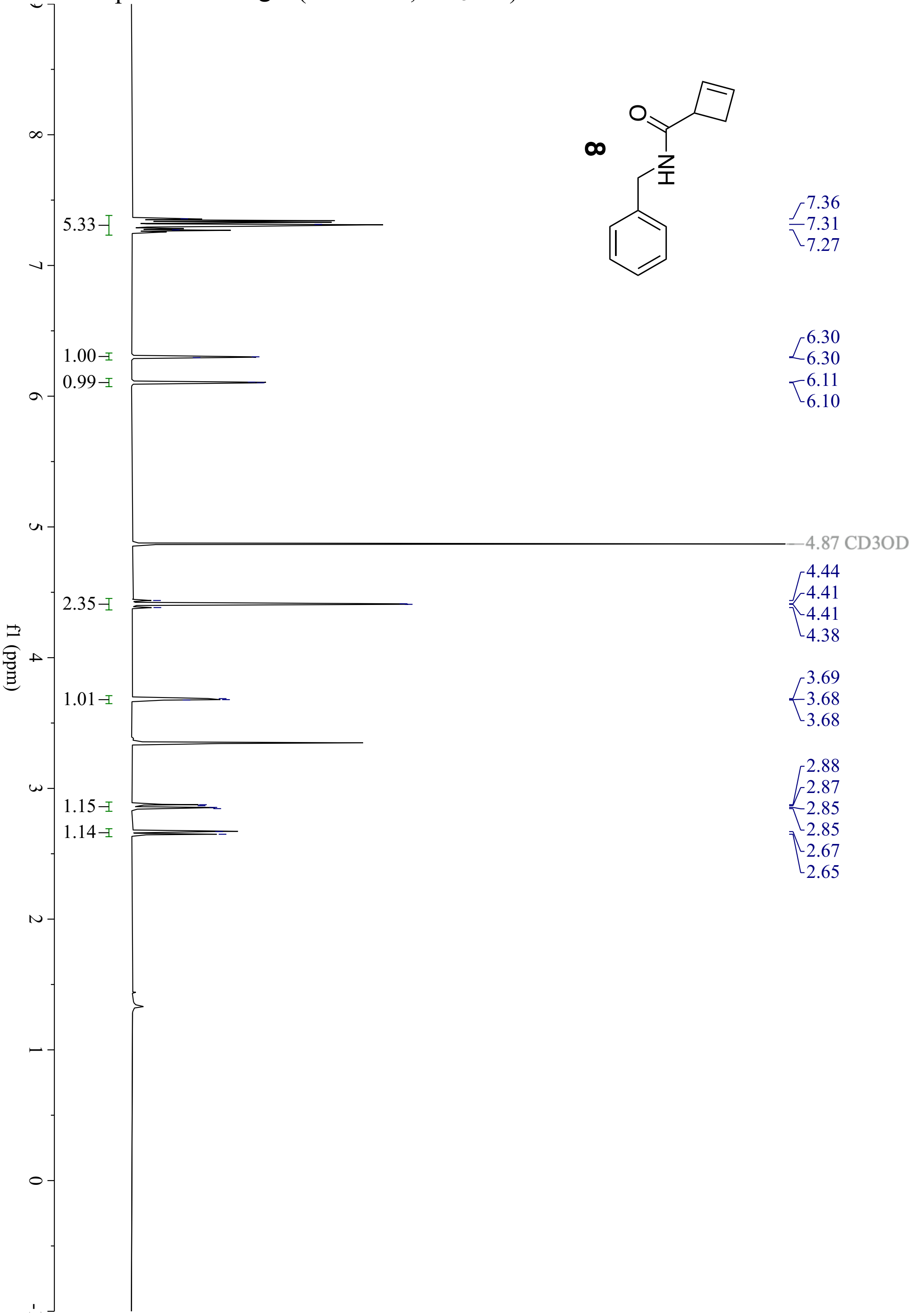

^{13}C NMR spectrum of **8** (150 MHz, CD_3OD)

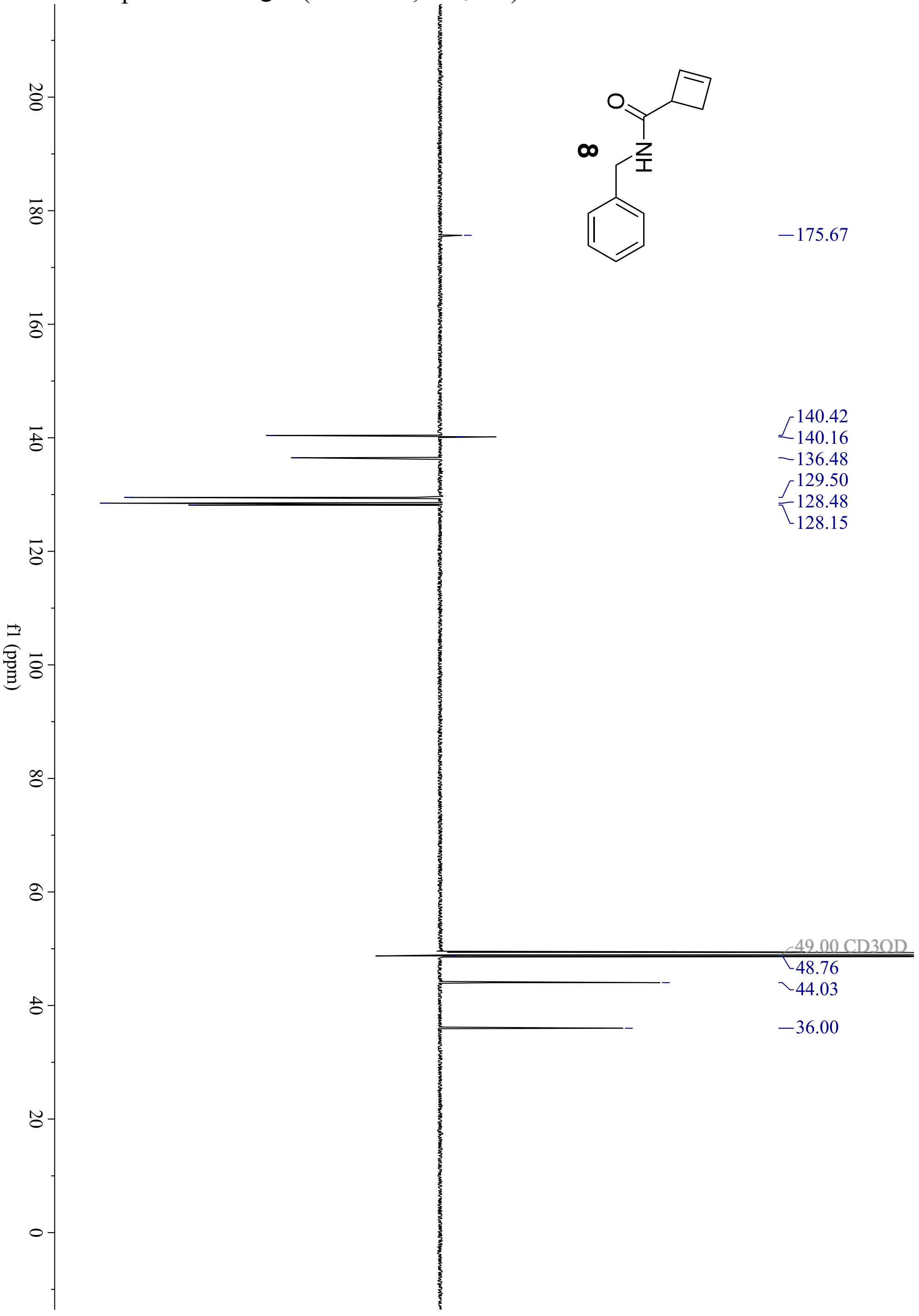

^{1}H NMR spectrum of **6** (600 MHz, CD_3OD)

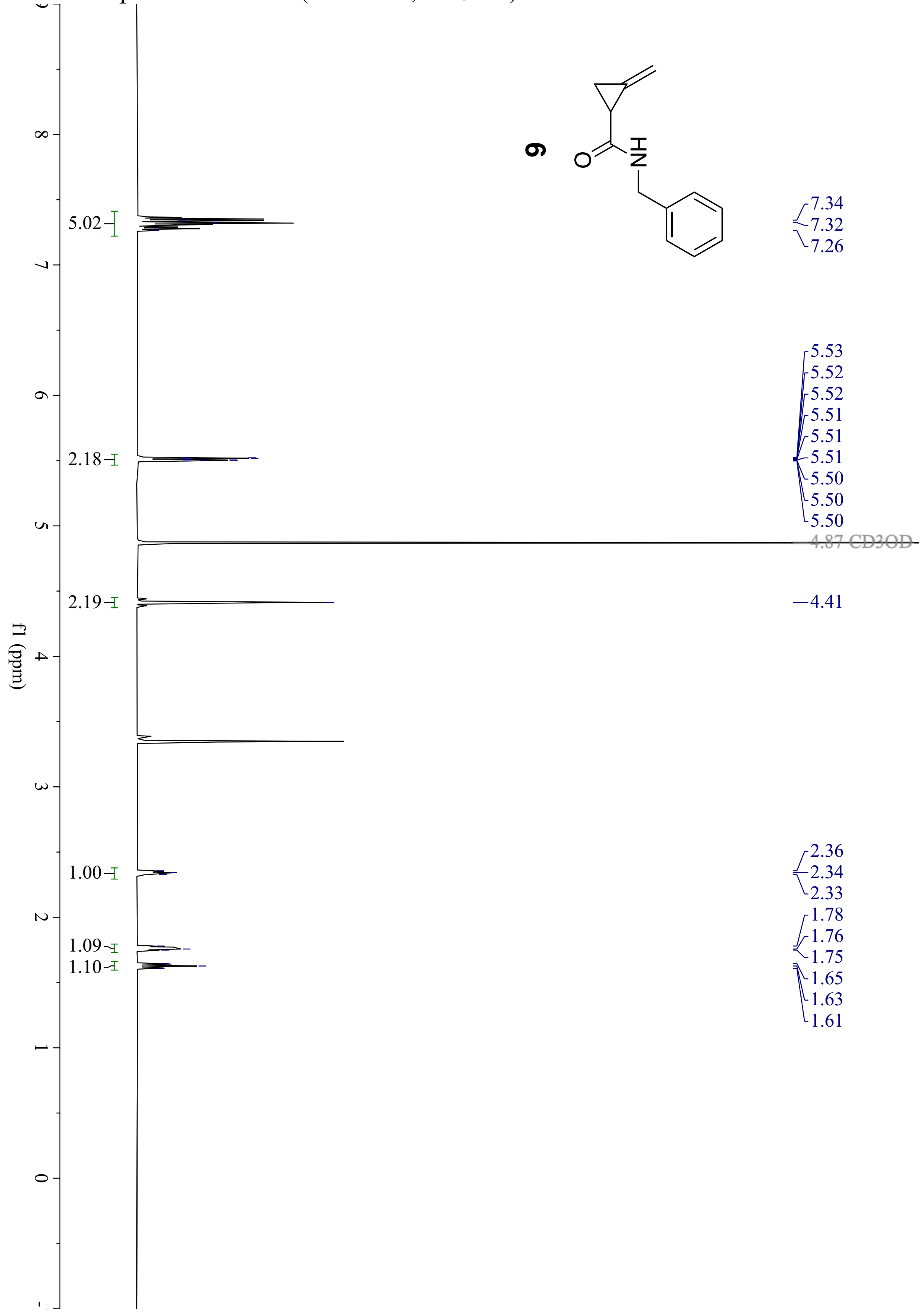

^{13}C NMR spectrum of **6** (101 MHz, CD_3OD)

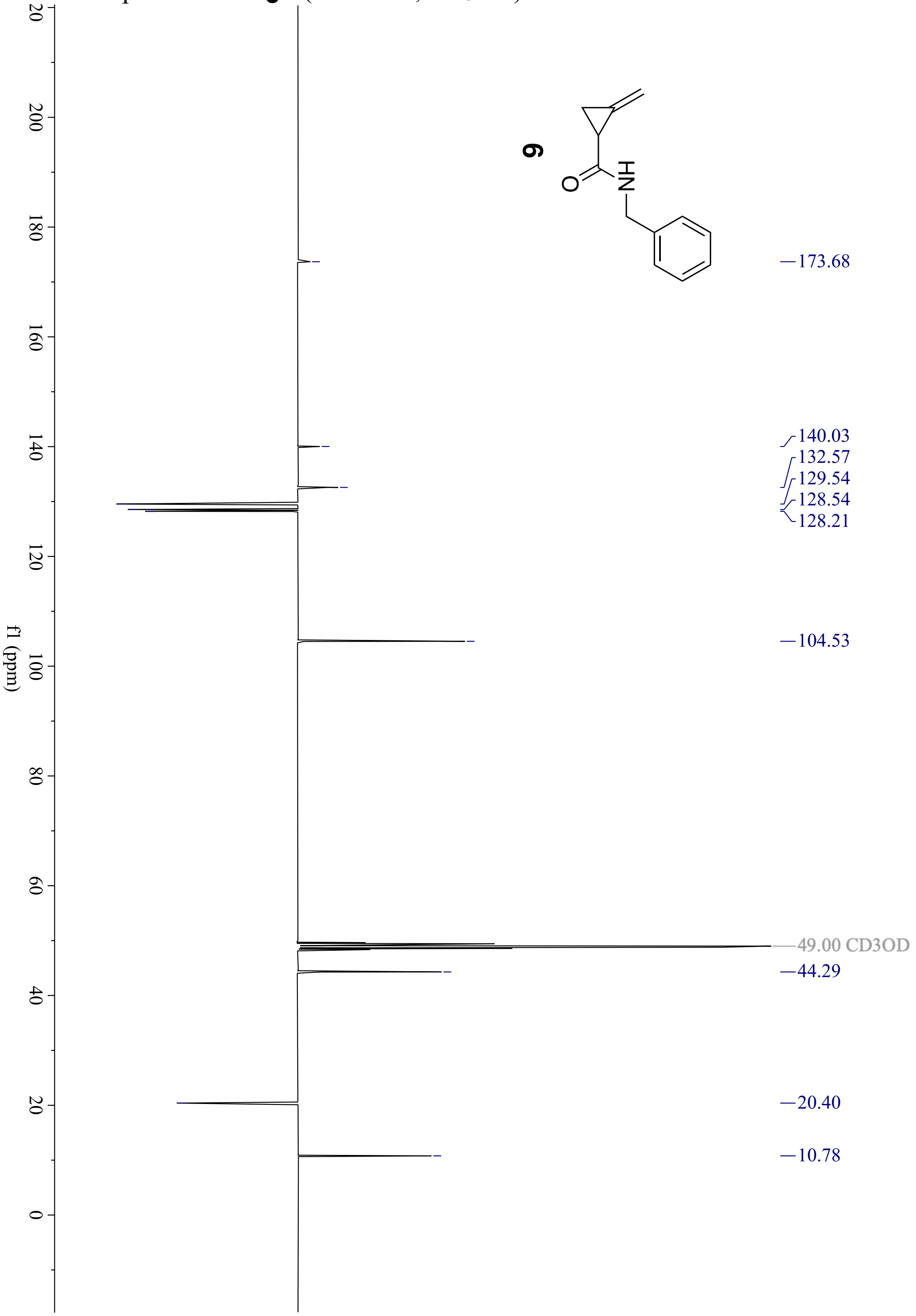

^1H NMR spectrum (400 MHz, $CDCl_3$)

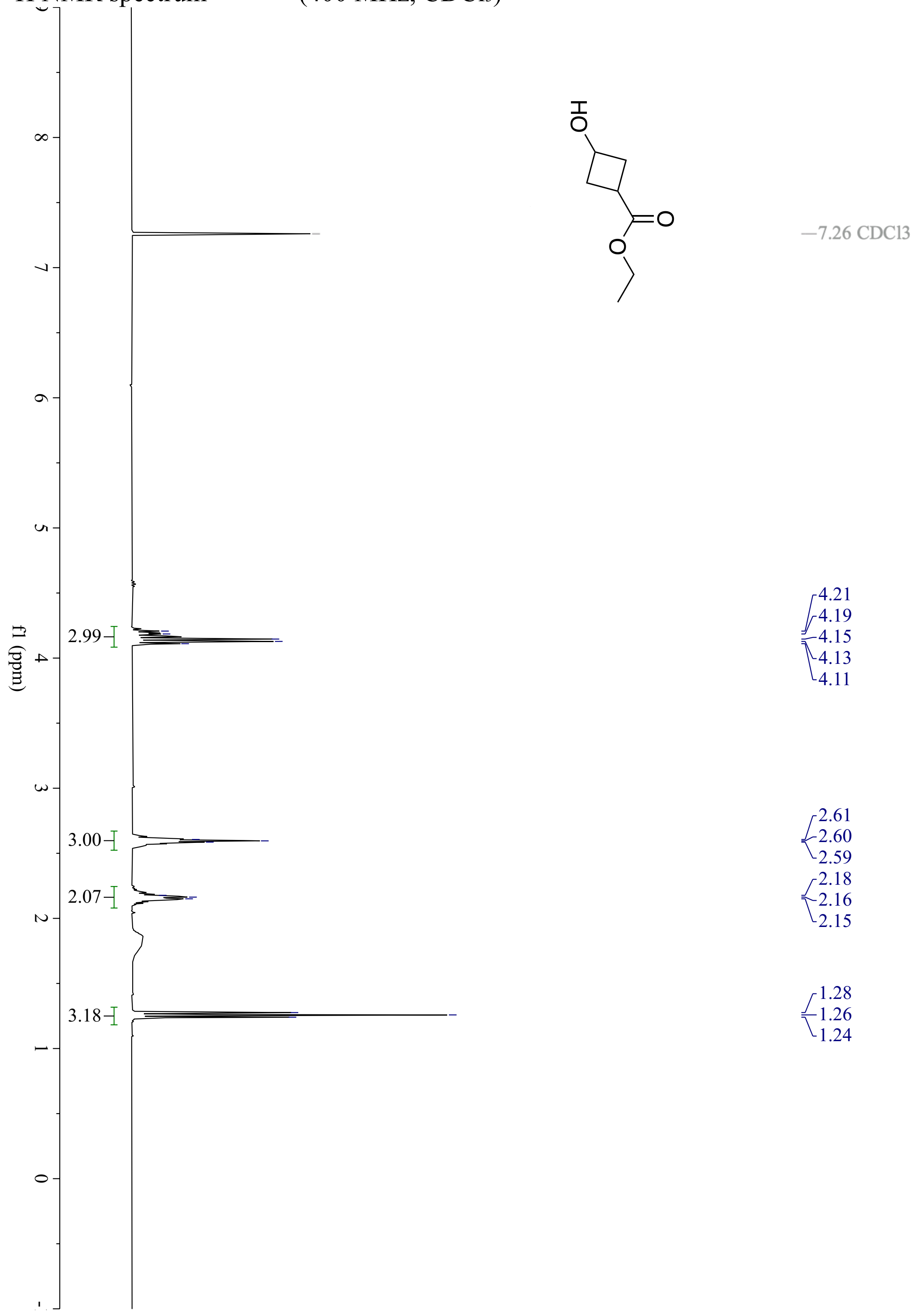

^{13}C NMR spectrum (101 MHz, $CDCl_3$)

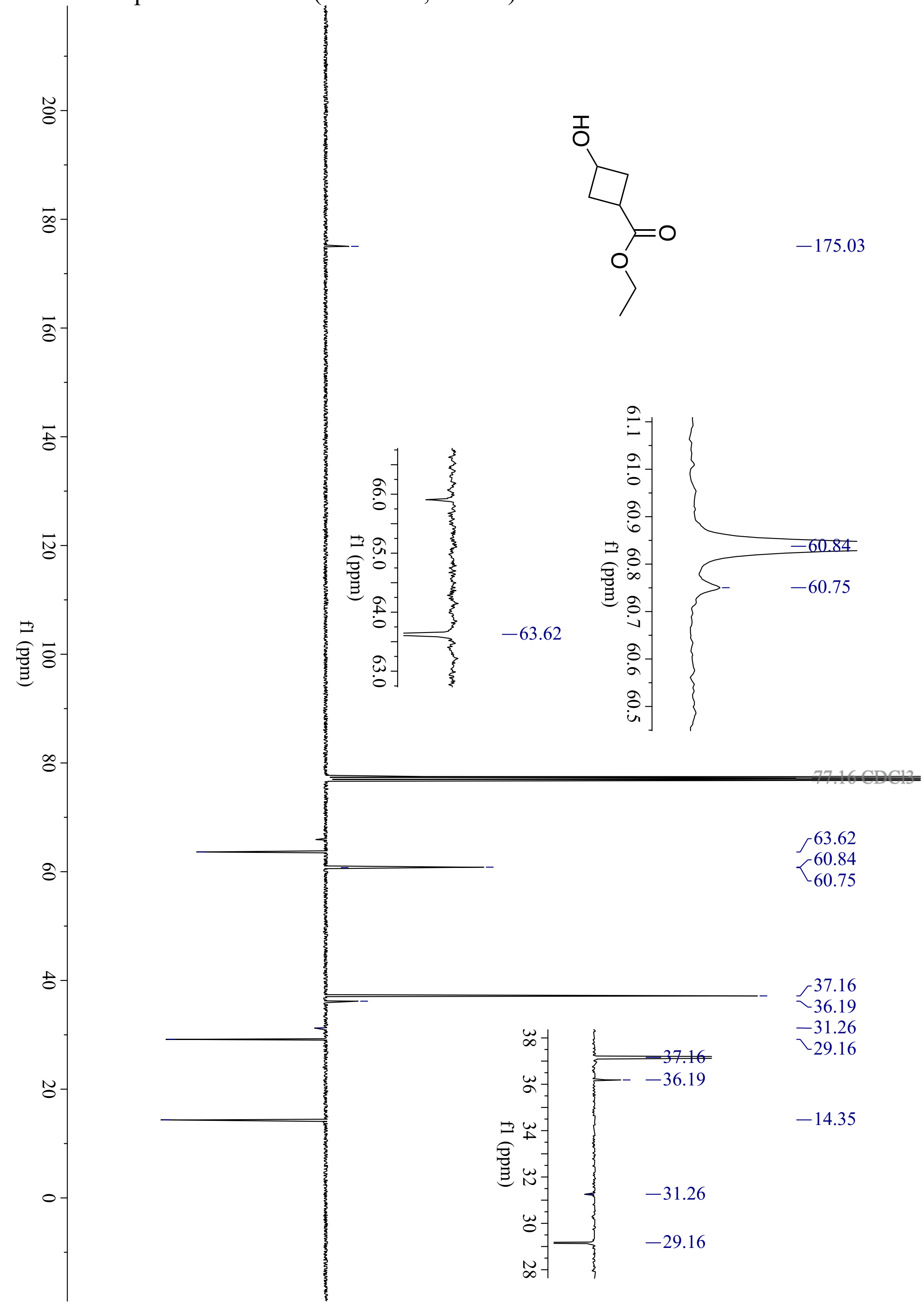

^{1}H NMR spectrum of (600 MHz, $CDCl_3$)

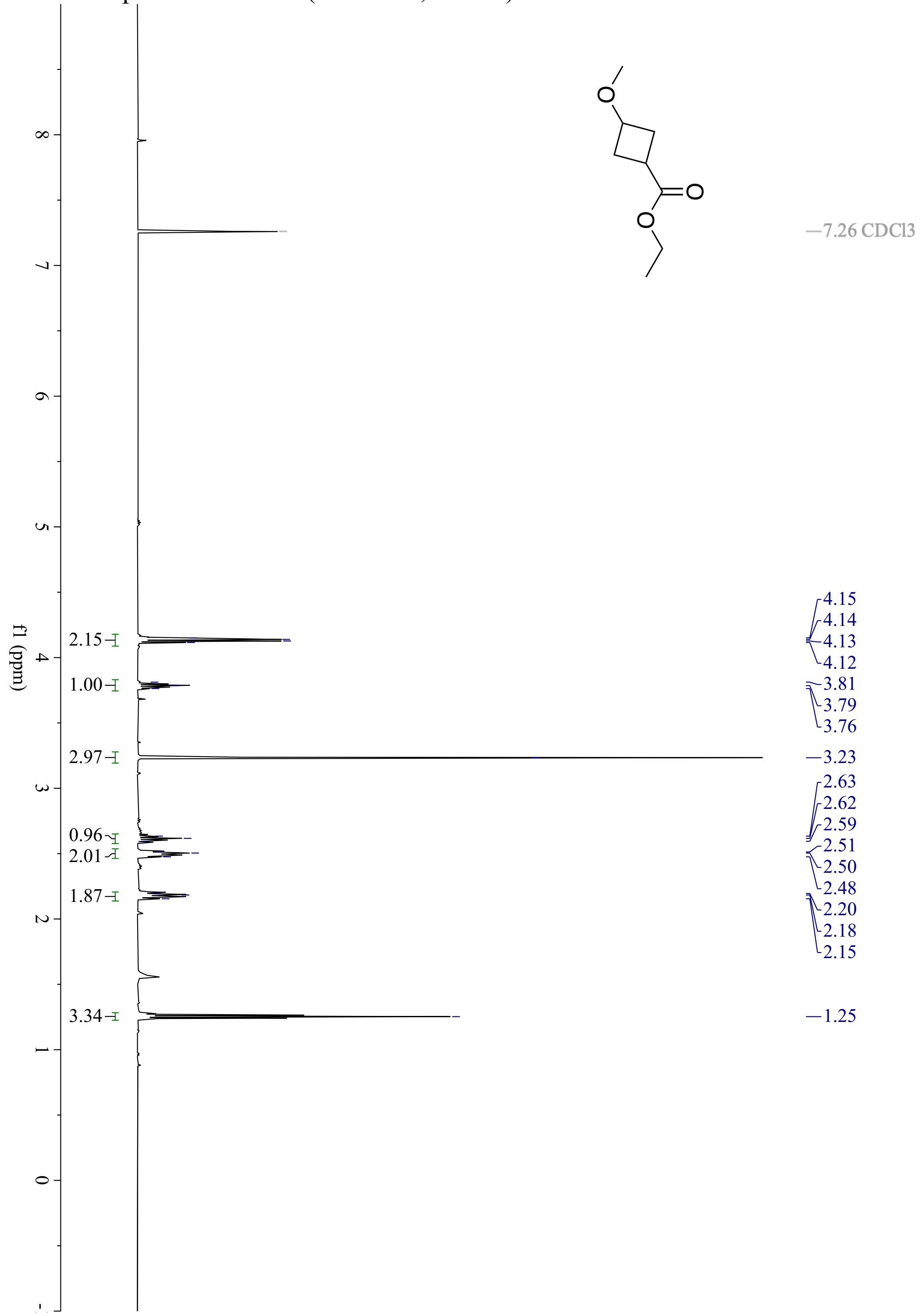

^{13}C NMR spectrum (150 MHz, $CDCl_3$)

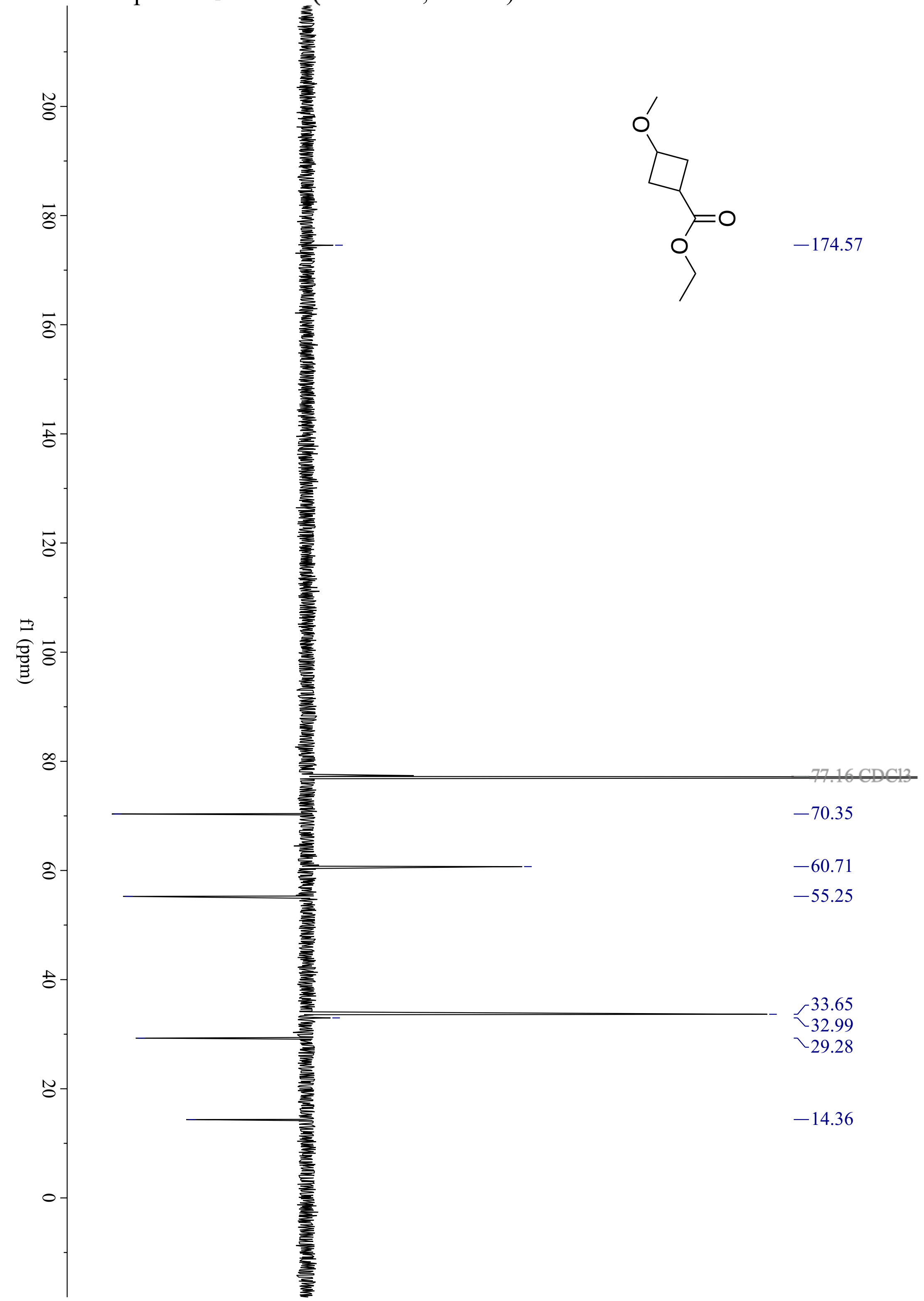

^{1}H NMR spectrum (400 MHz, $CDCl_3$)

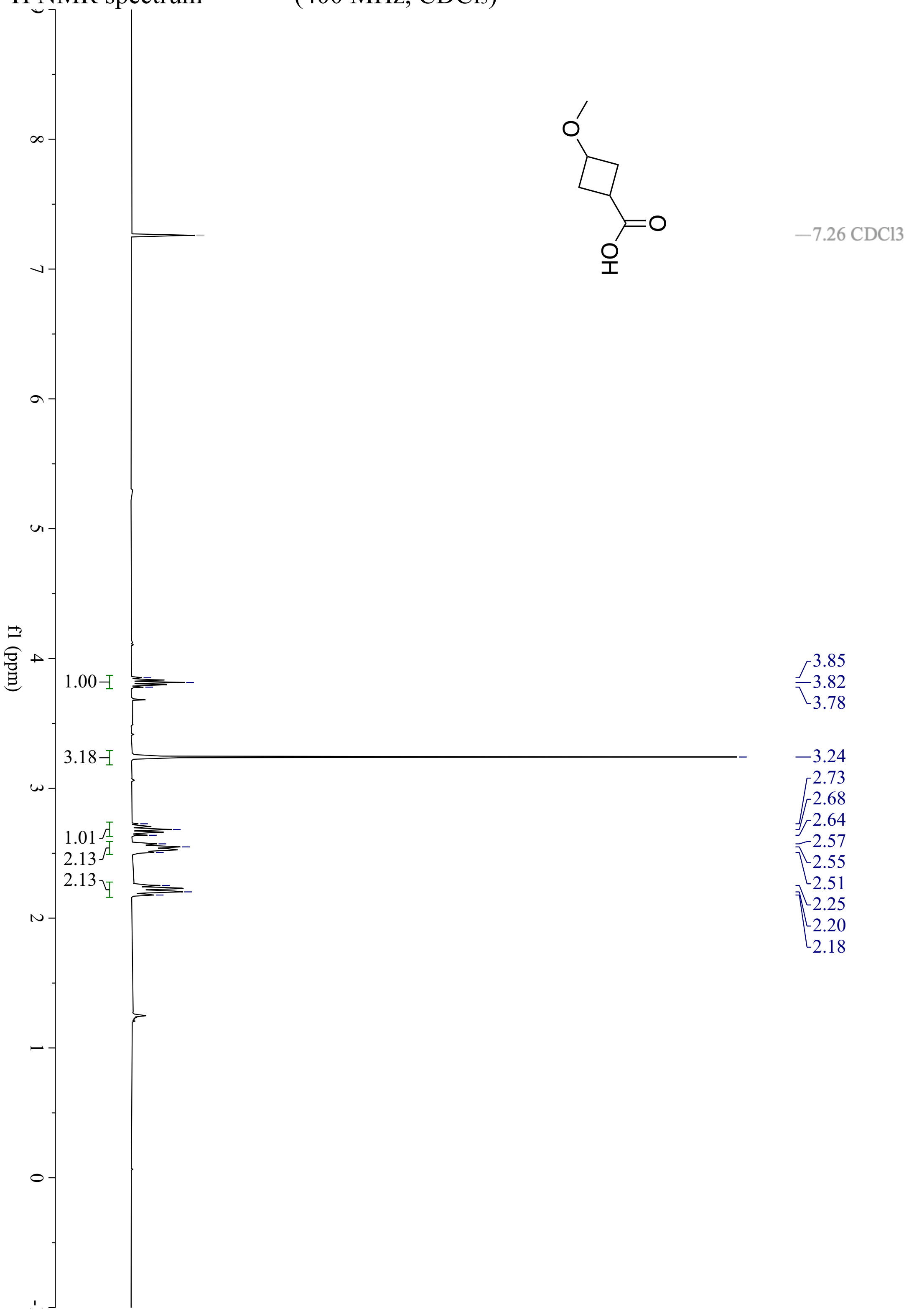

^{13}C NMR spectrum (101 MHz, $CDCl_3$)

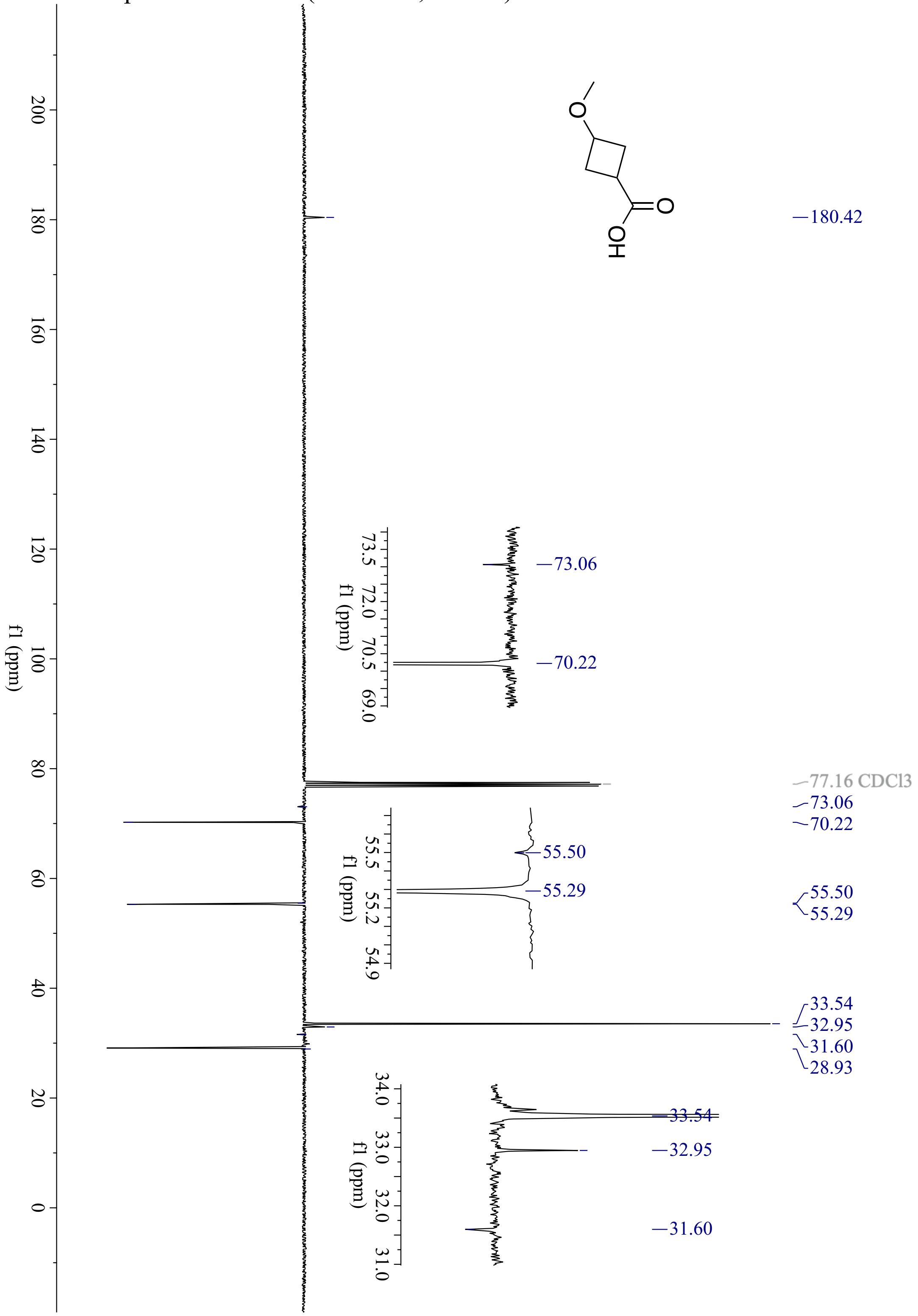

^{1}H NMR spectrum of **11** (600 MHz, CD_3OD)

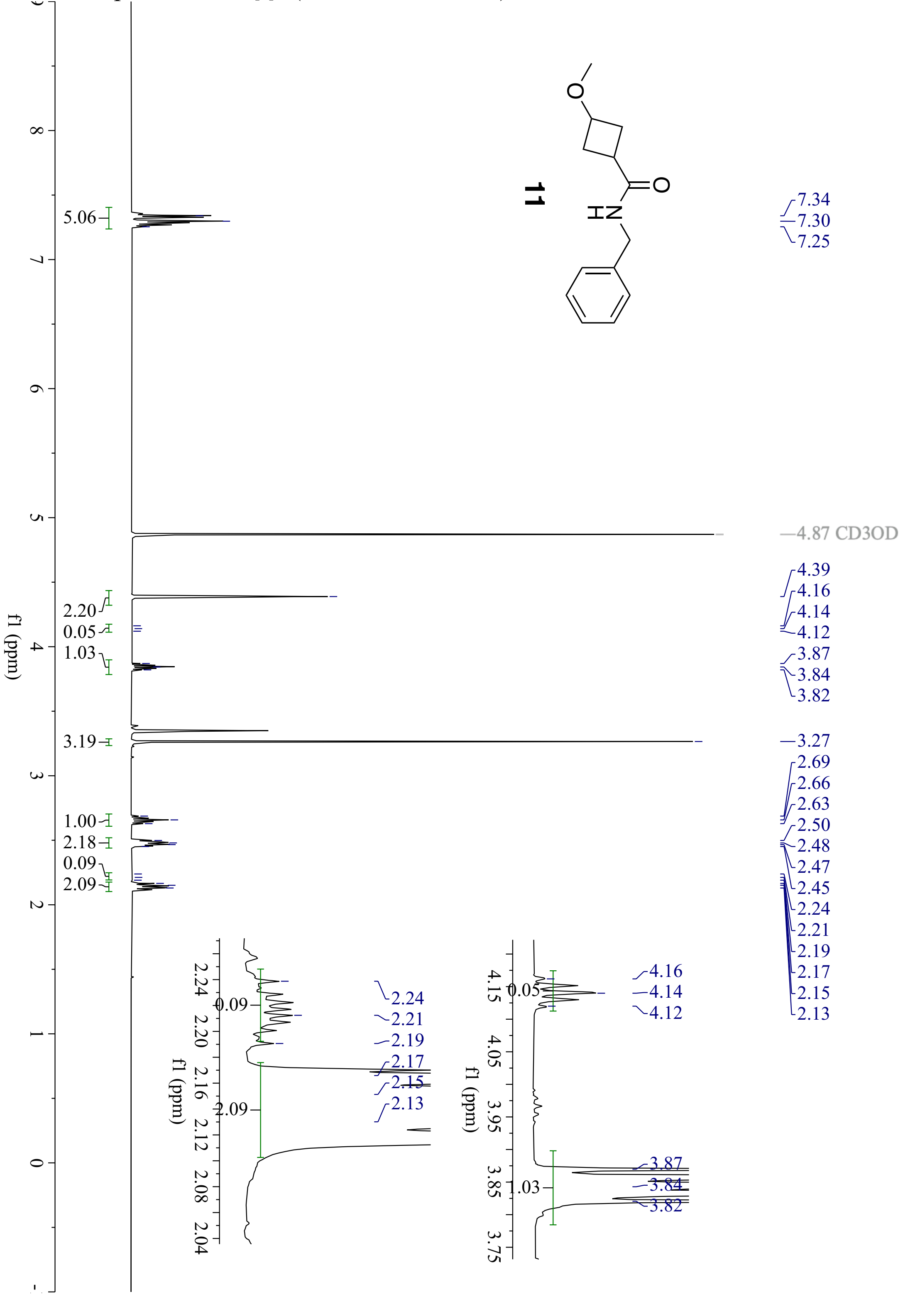

^{13}C NMR spectrum of **11** (101 MHz, $CDCl_3$)

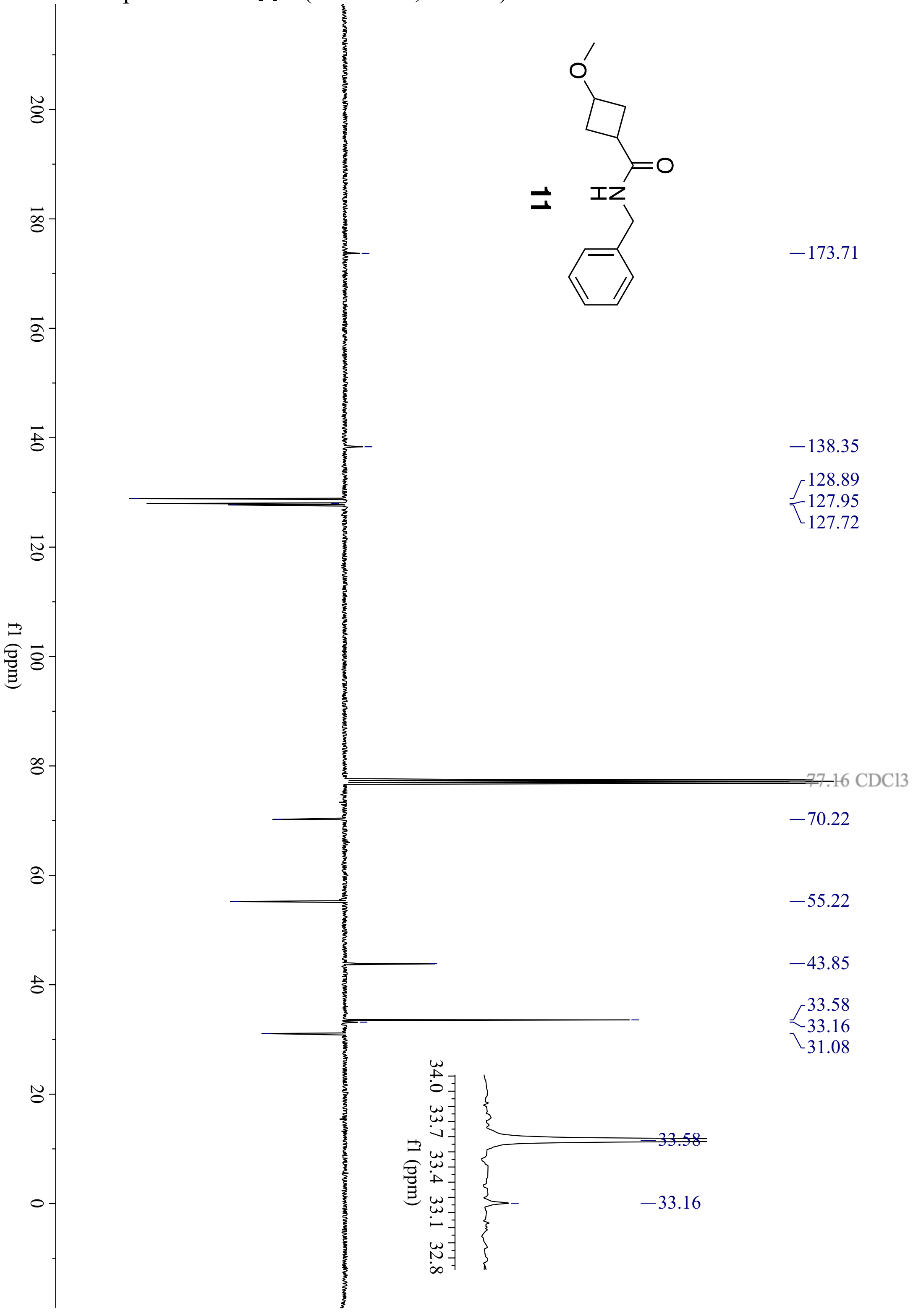

^{1}H NMR spectrum of **30** (600 MHz, CD_3OD)

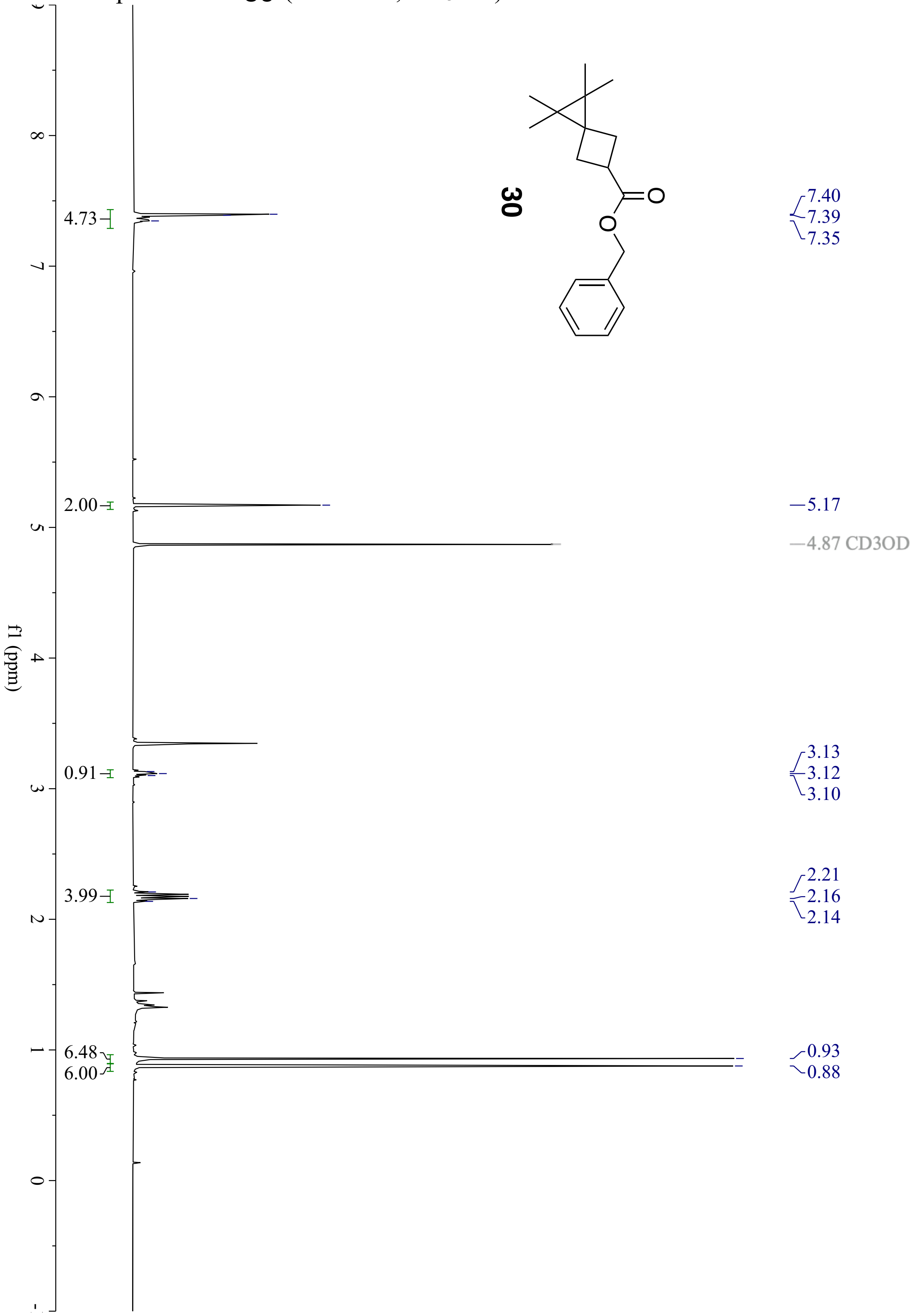

^{13}C NMR spectrum of **30** (150 MHz, CD_3OD)

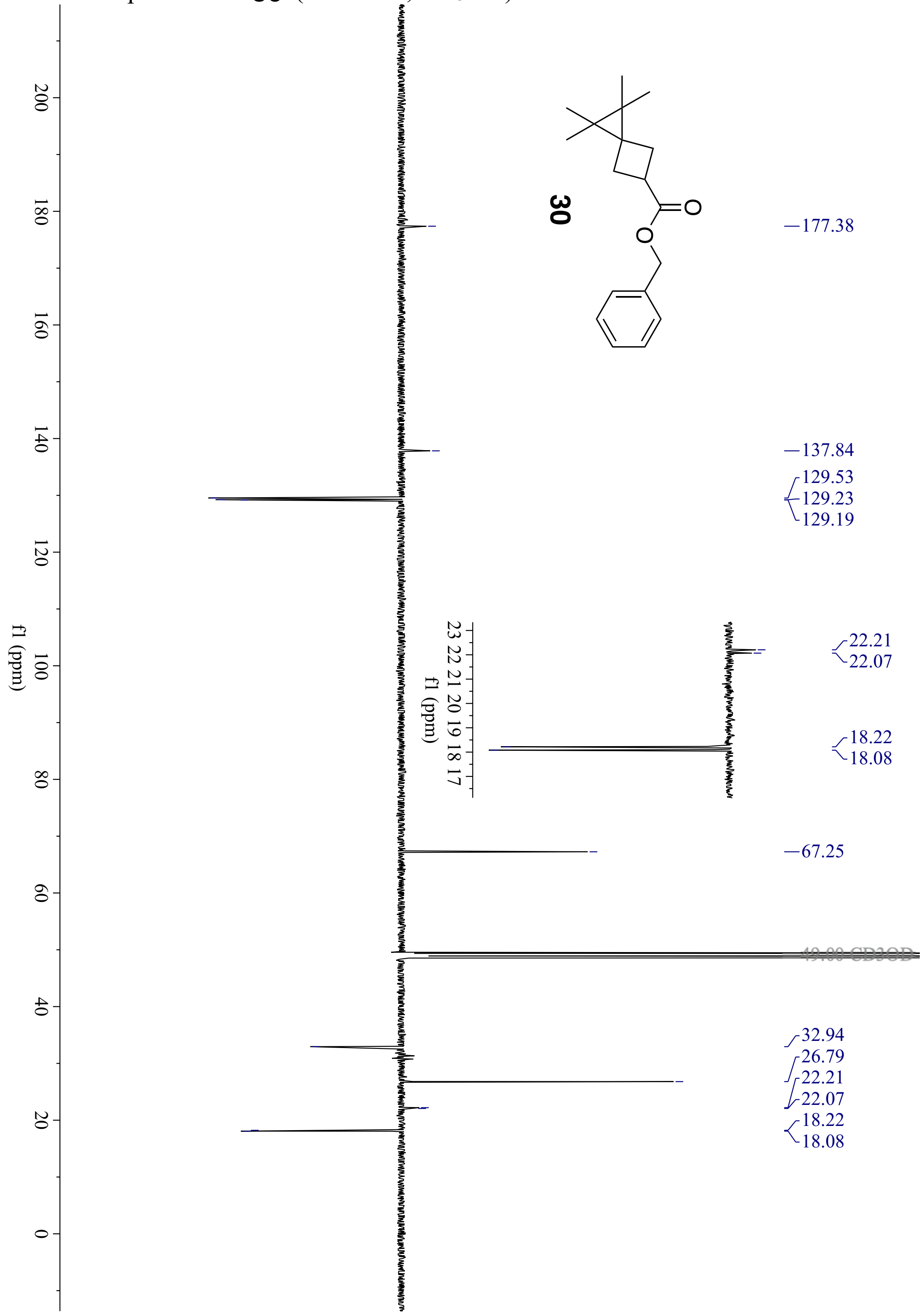

^{1}H NMR spectrum of **31** (400 MHz, $CDCl_3$)

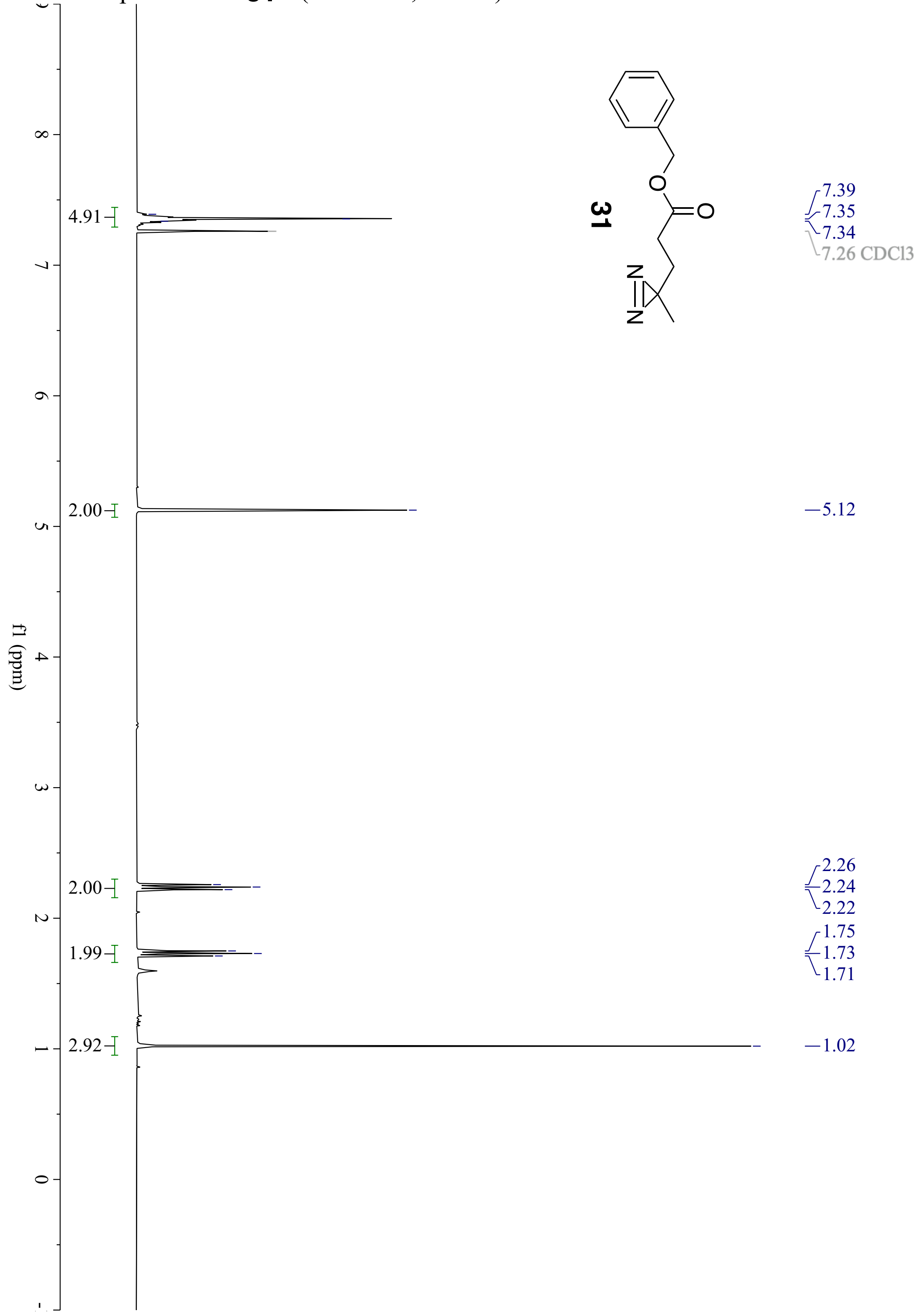

^{13}C NMR spectrum of **31** (101 MHz, $CDCl_3$)

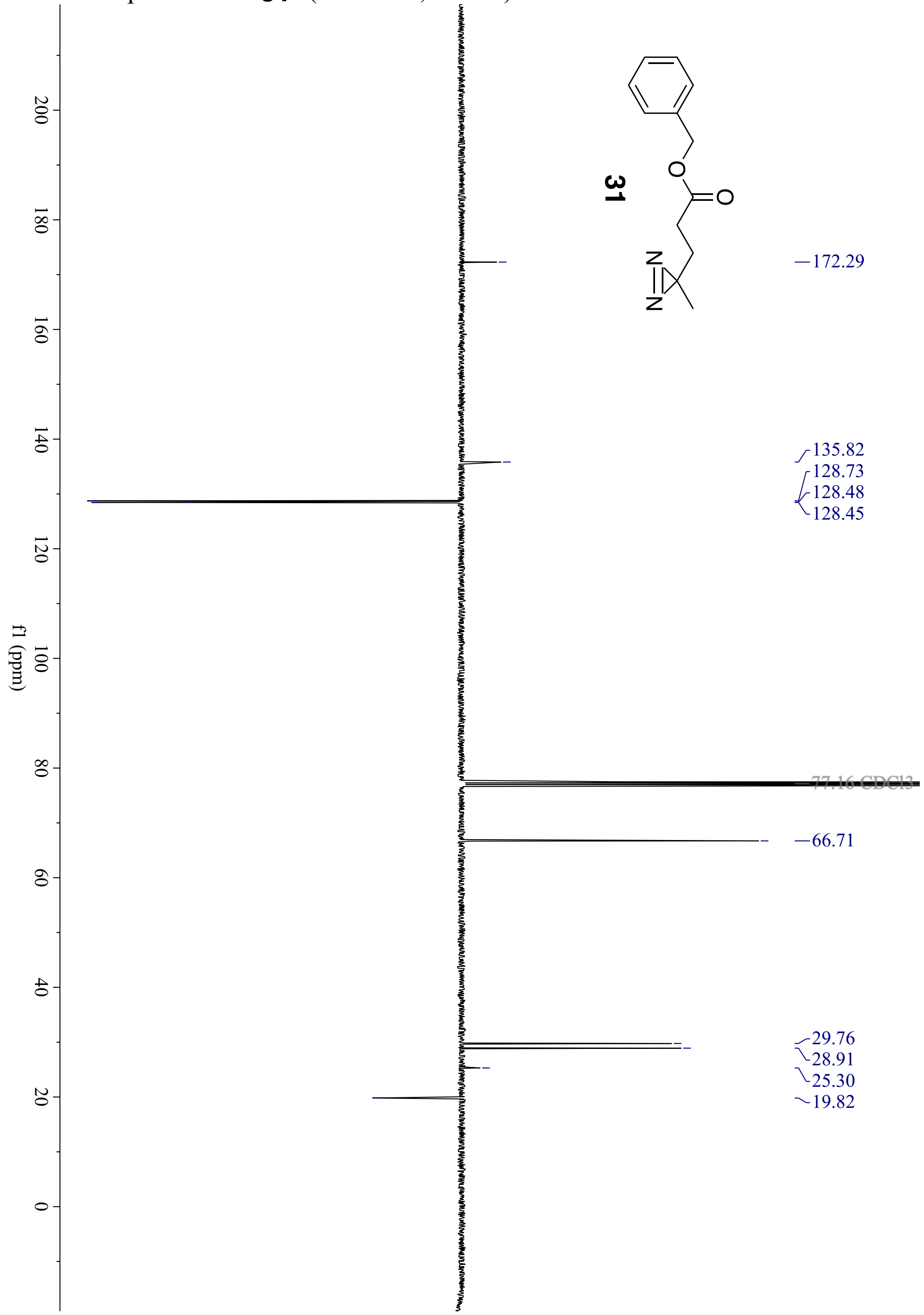

^{1}H NMR spectrum (600 MHz, CD_3OD)

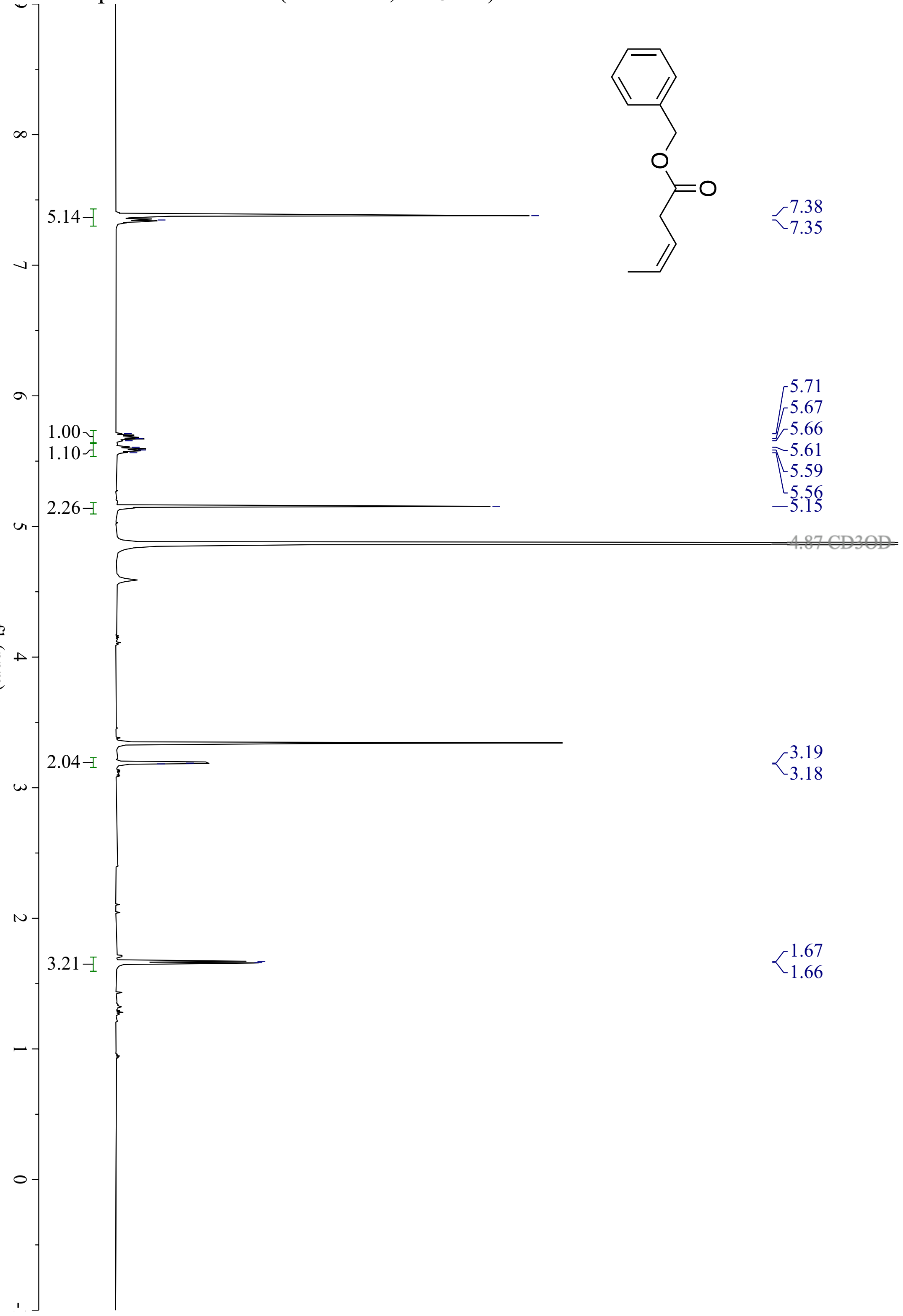

^{13}C NMR spectrum (101 MHz, $CDCl_3$)

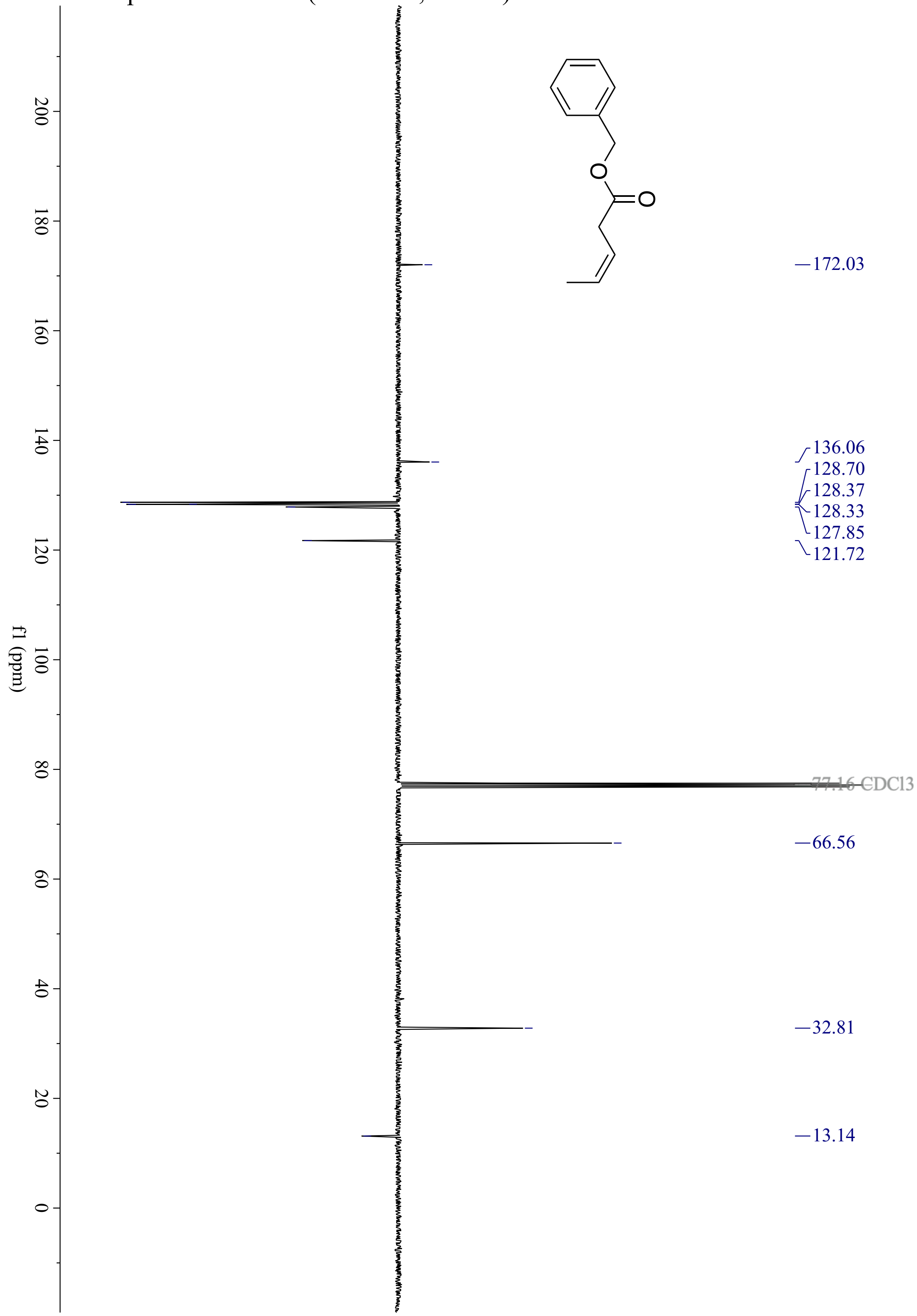

^{1}H NMR spectrum (600 MHz, CD_3OD)

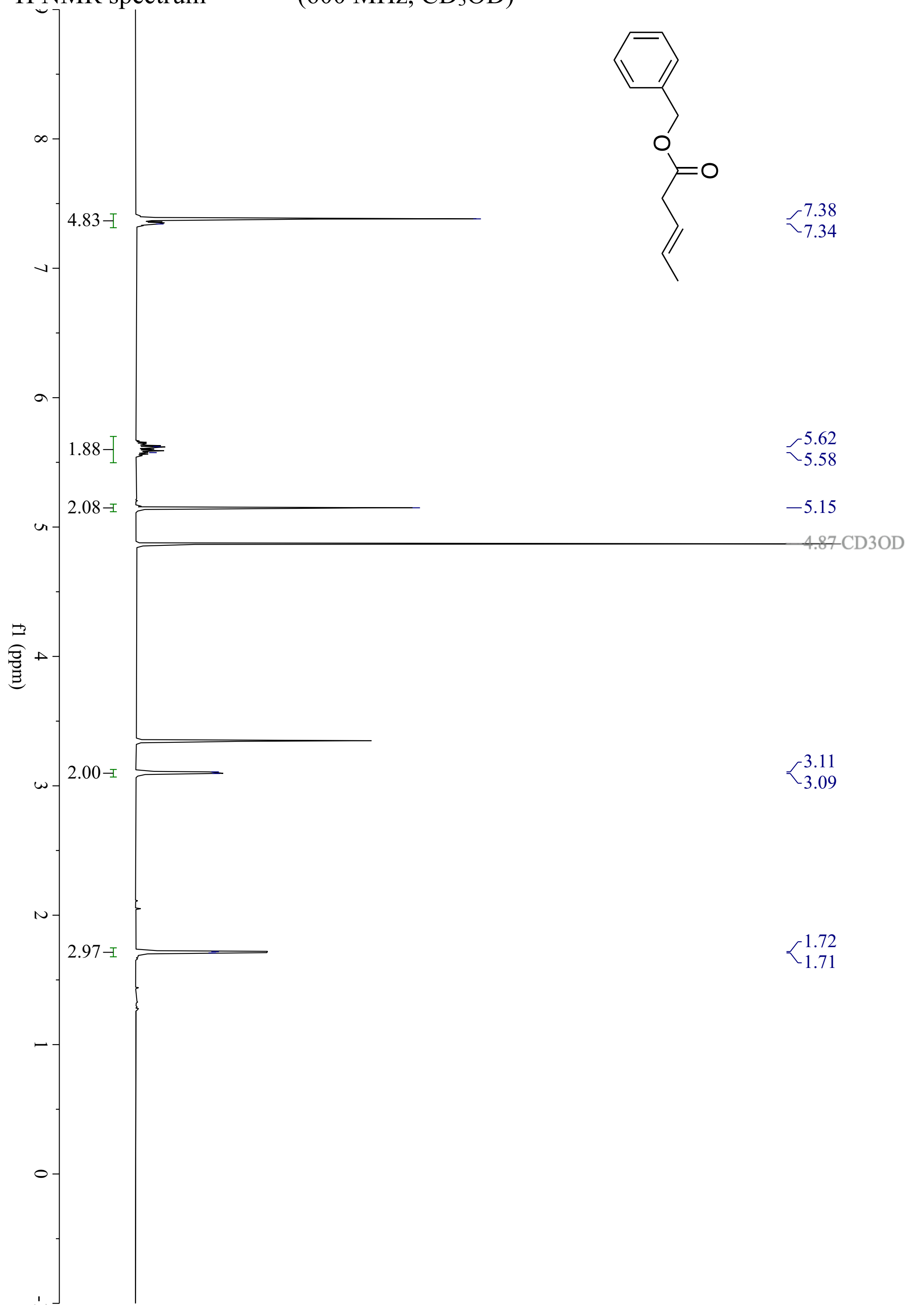

^{13}C NMR spectrum (101 MHz, $CDCl_3$)

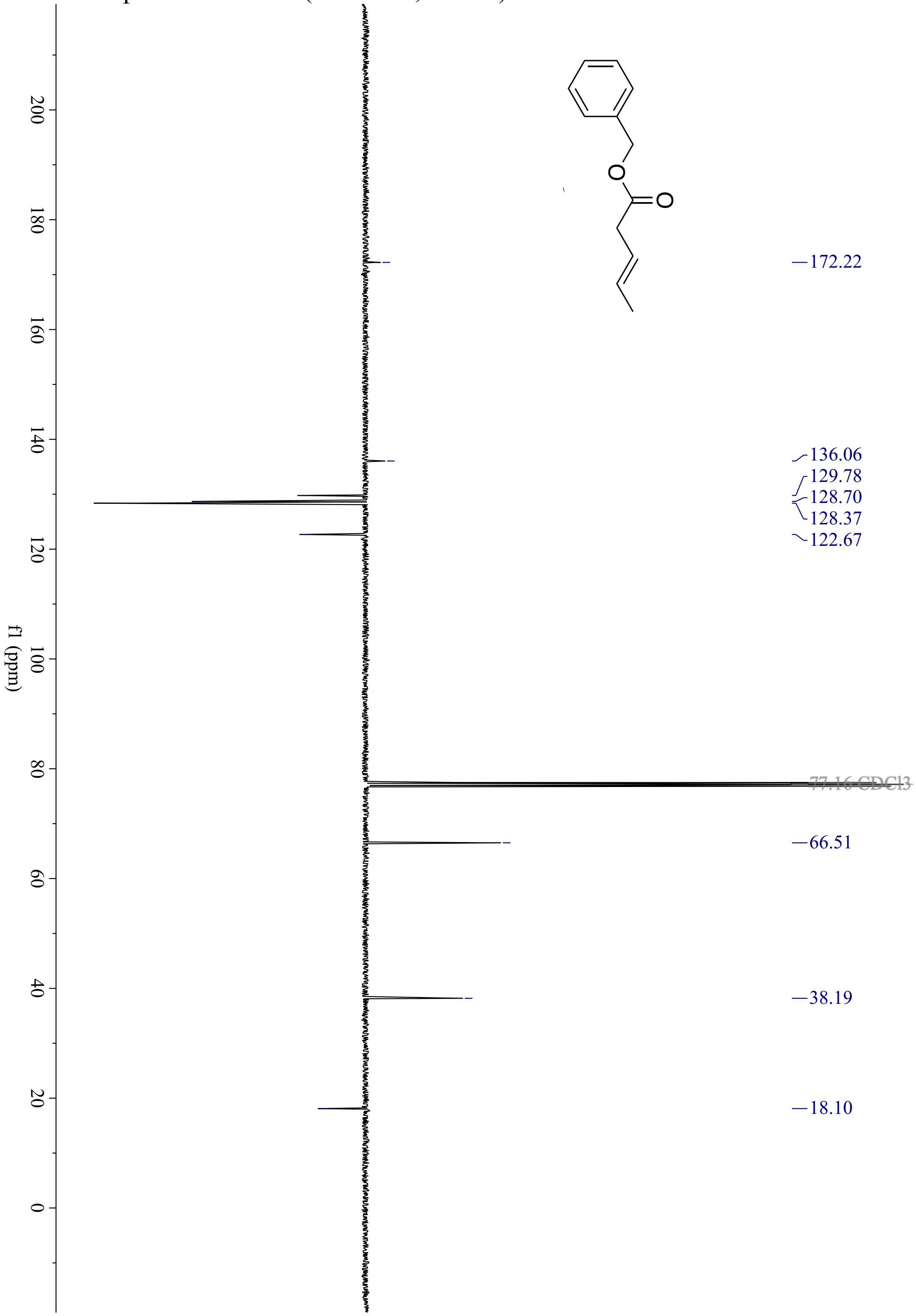

^{1}H NMR spectrum (600 MHz, CD_3OD)

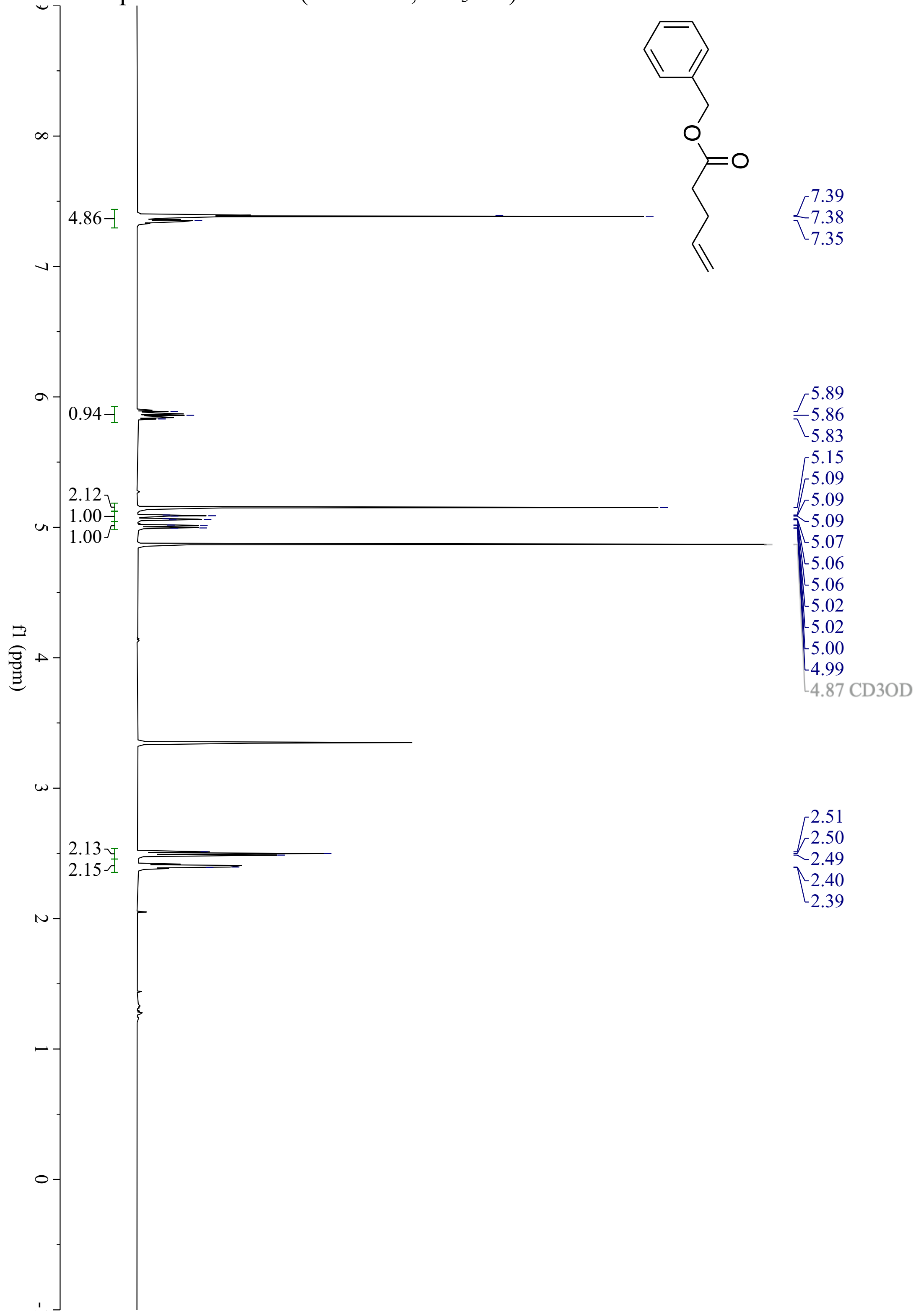

^{13}C NMR spectrum (101 MHz, $CDCl_3$)

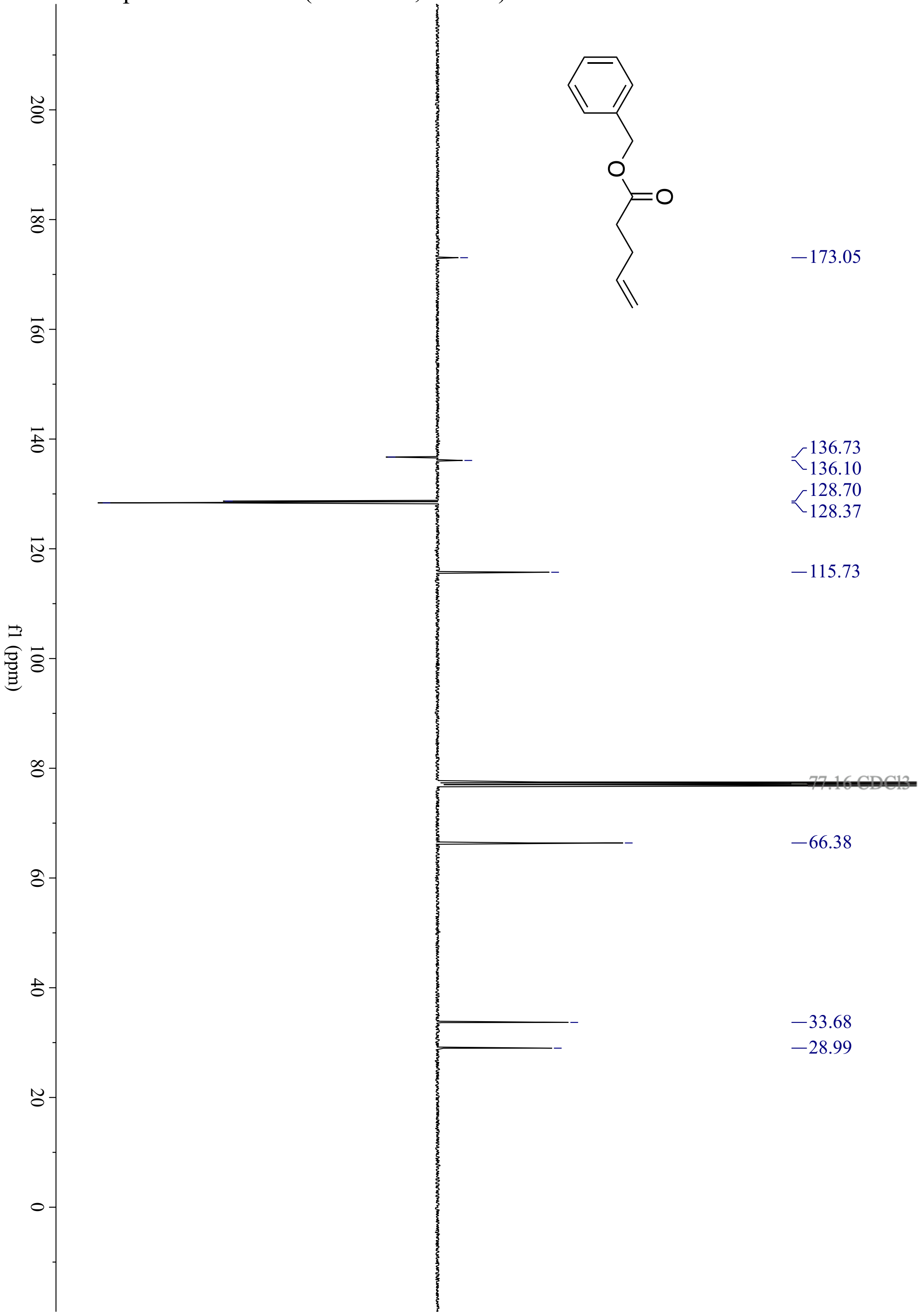

^{1}H NMR spectrum (600 MHz, CD_3OD)

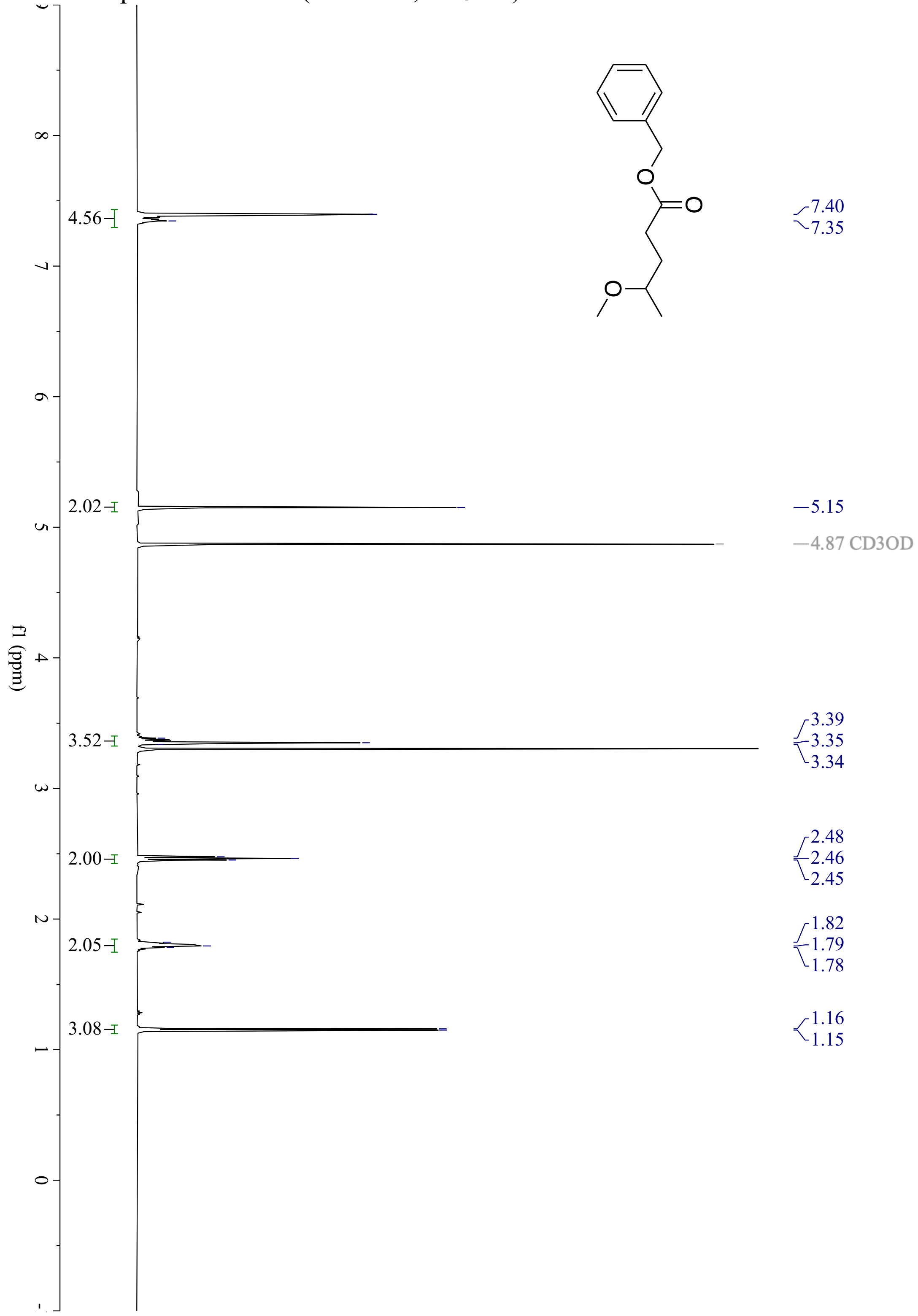

^{13}C NMR spectrum (101 MHz, $CDCl_3$)

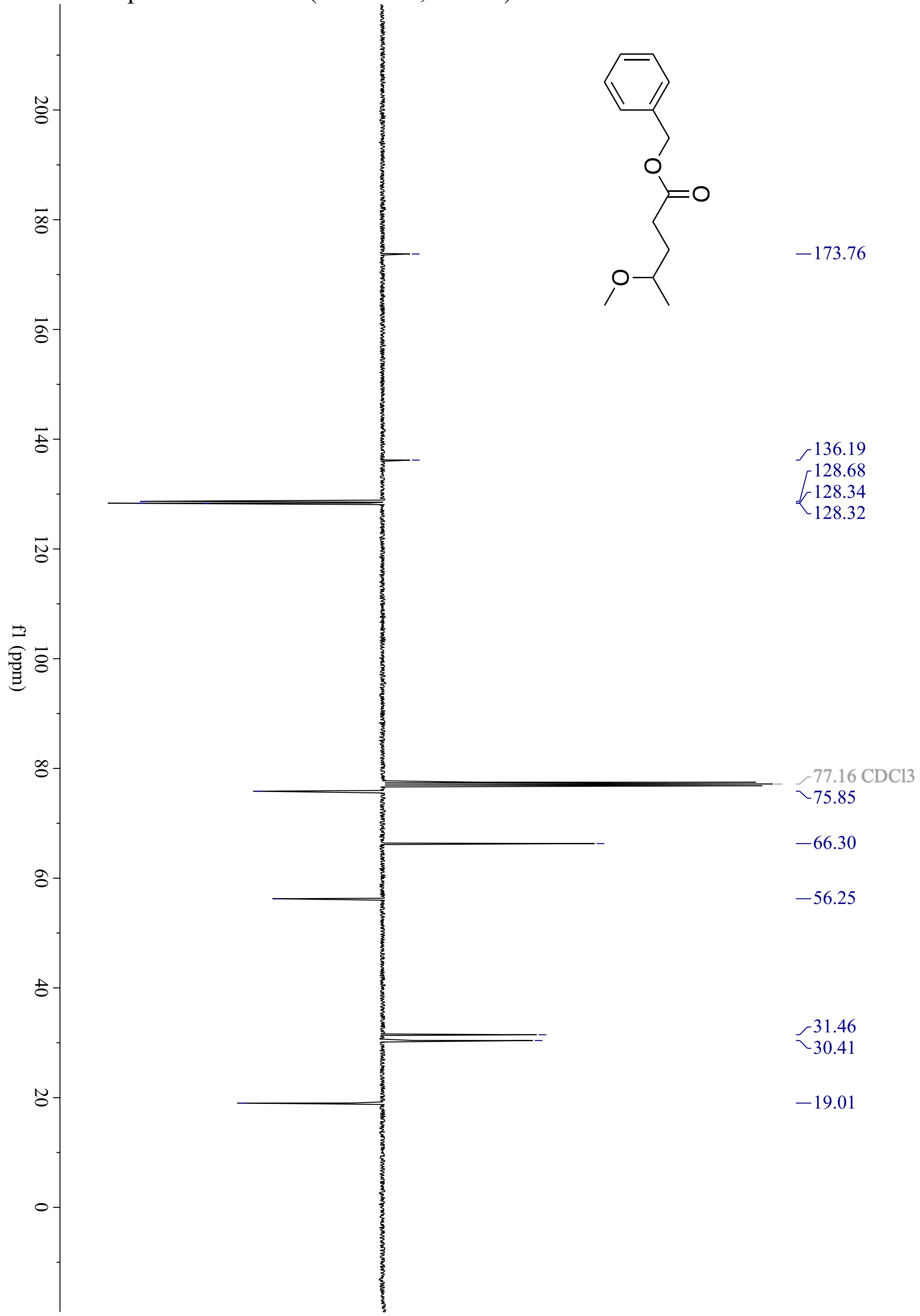

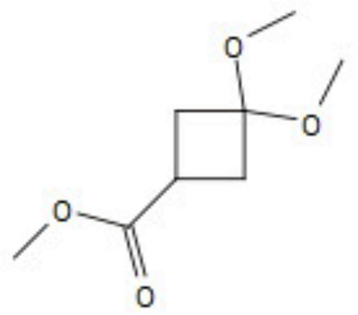

^{1}H NMR(400 MHz,$CDCl_3$)

17

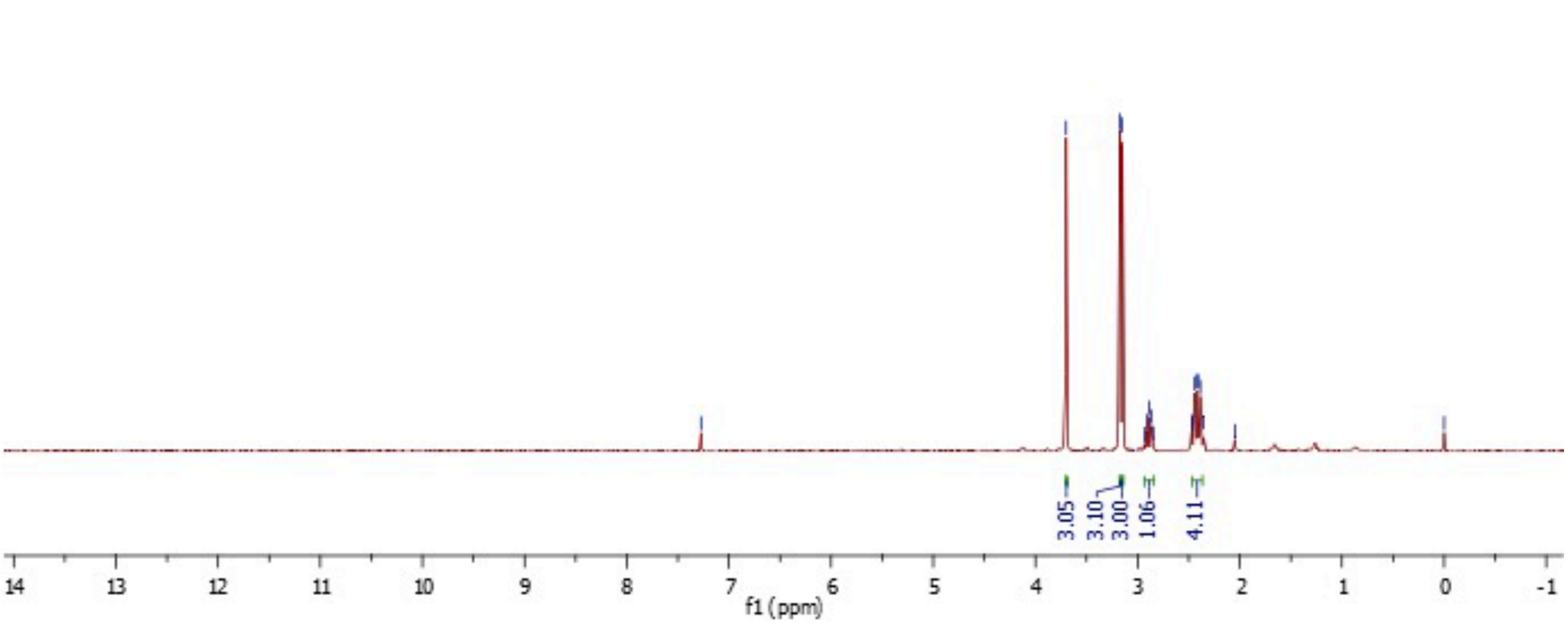

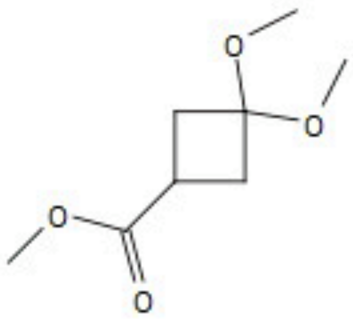

^{13}C NMR(101 MHz,$CDCl_3$)

17

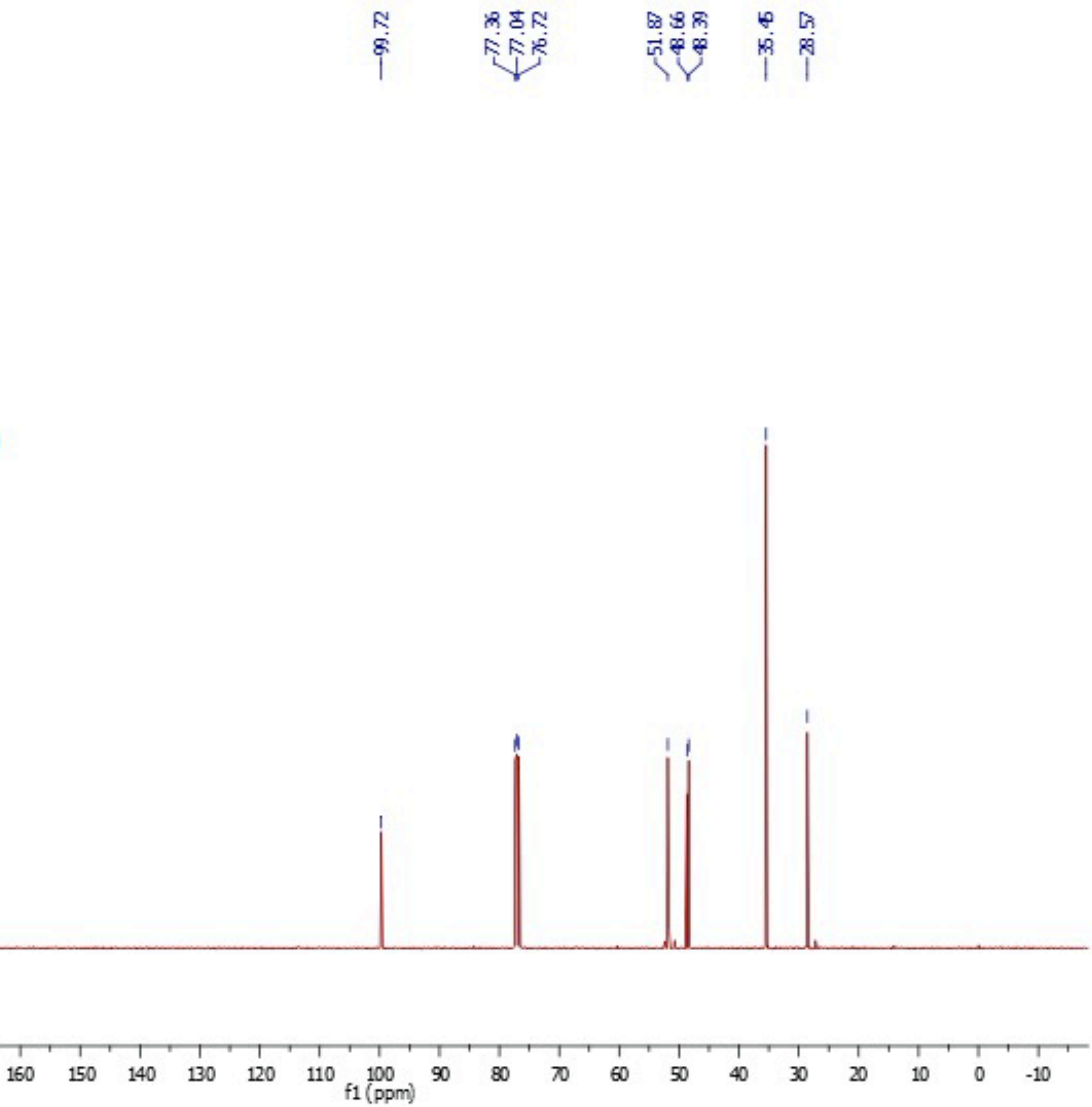

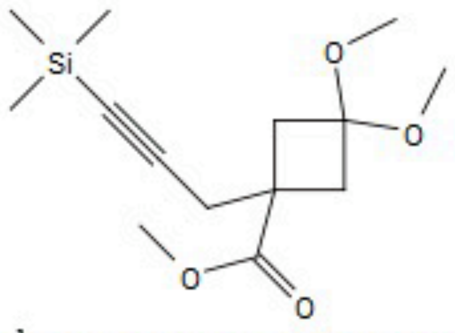

^{1}H NMR(400 MHz,CDCl$_3$)

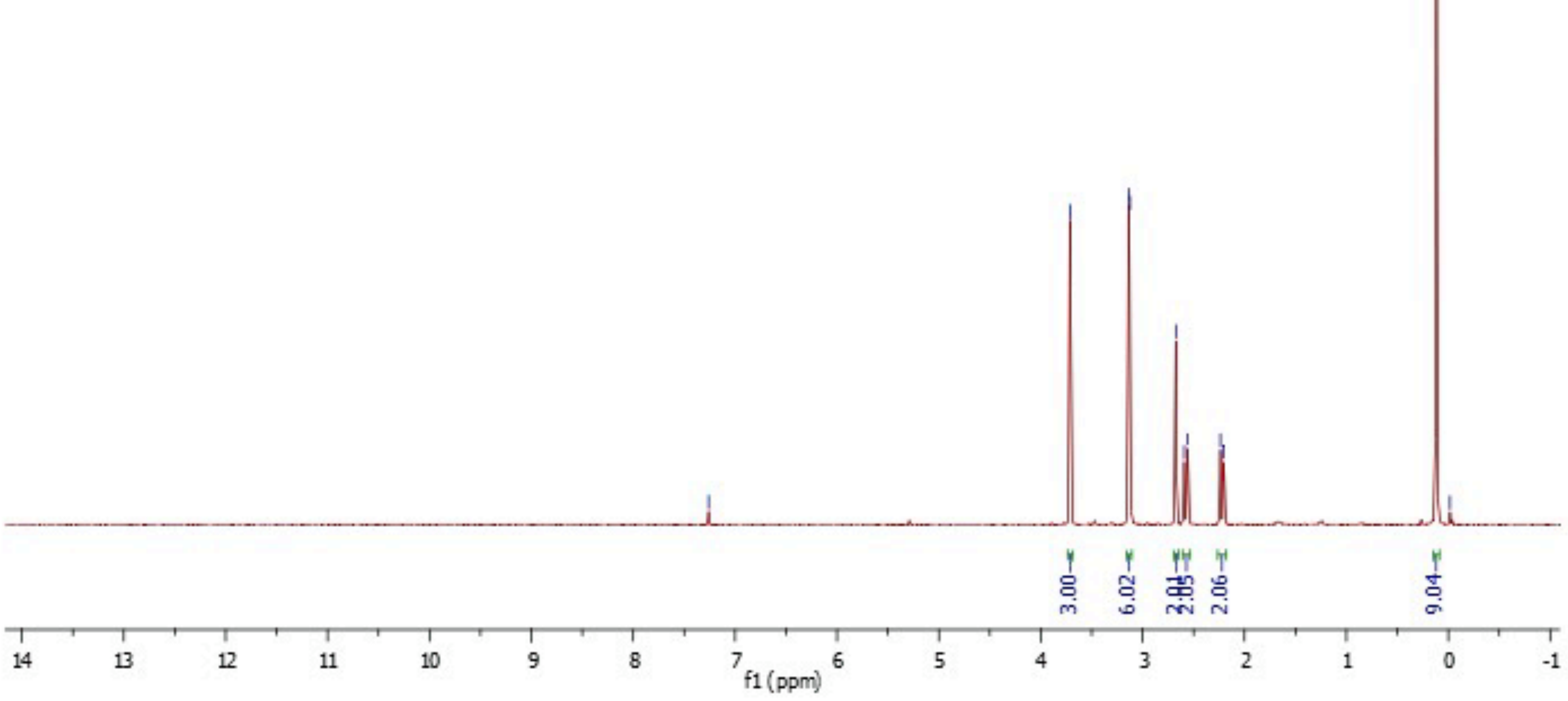

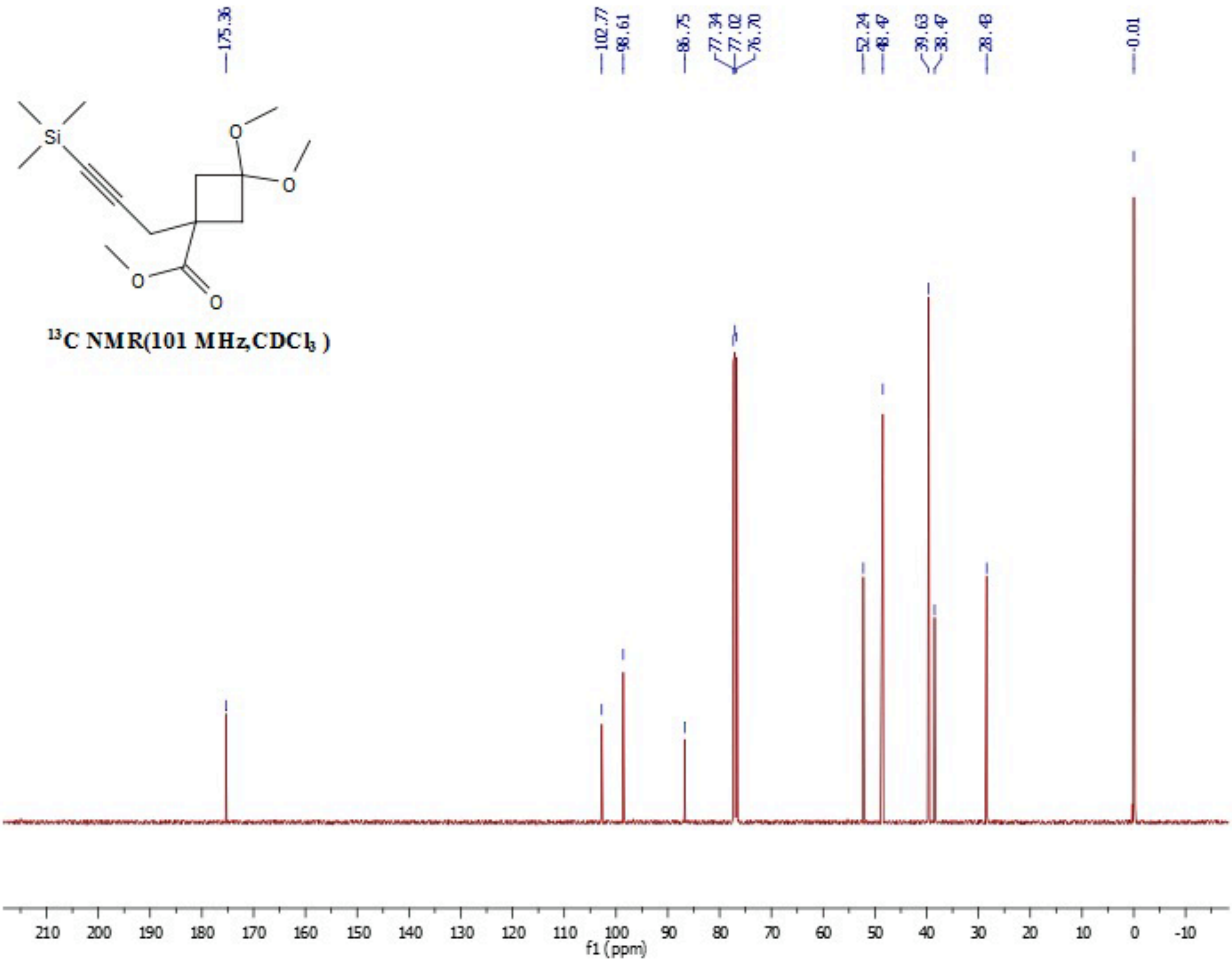

175.36
102.77
98.61
86.75
77.34
77.02
76.70
52.24
48.47
39.63
38.47
28.43
-0.01
Si
O
O
O
O
^{13}C NMR(101 MHz,$CDCl_3$)
210
200
190
180
170
160
150
140
130
120
110
100
90
80
70
60
50
40
30
20
10
0
-10
f1 (ppm)

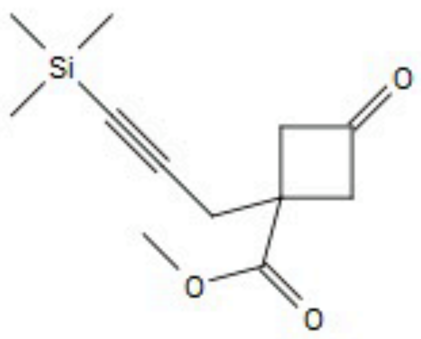

^{1}H NMR(400 MHz,$CDCl_3$)

18

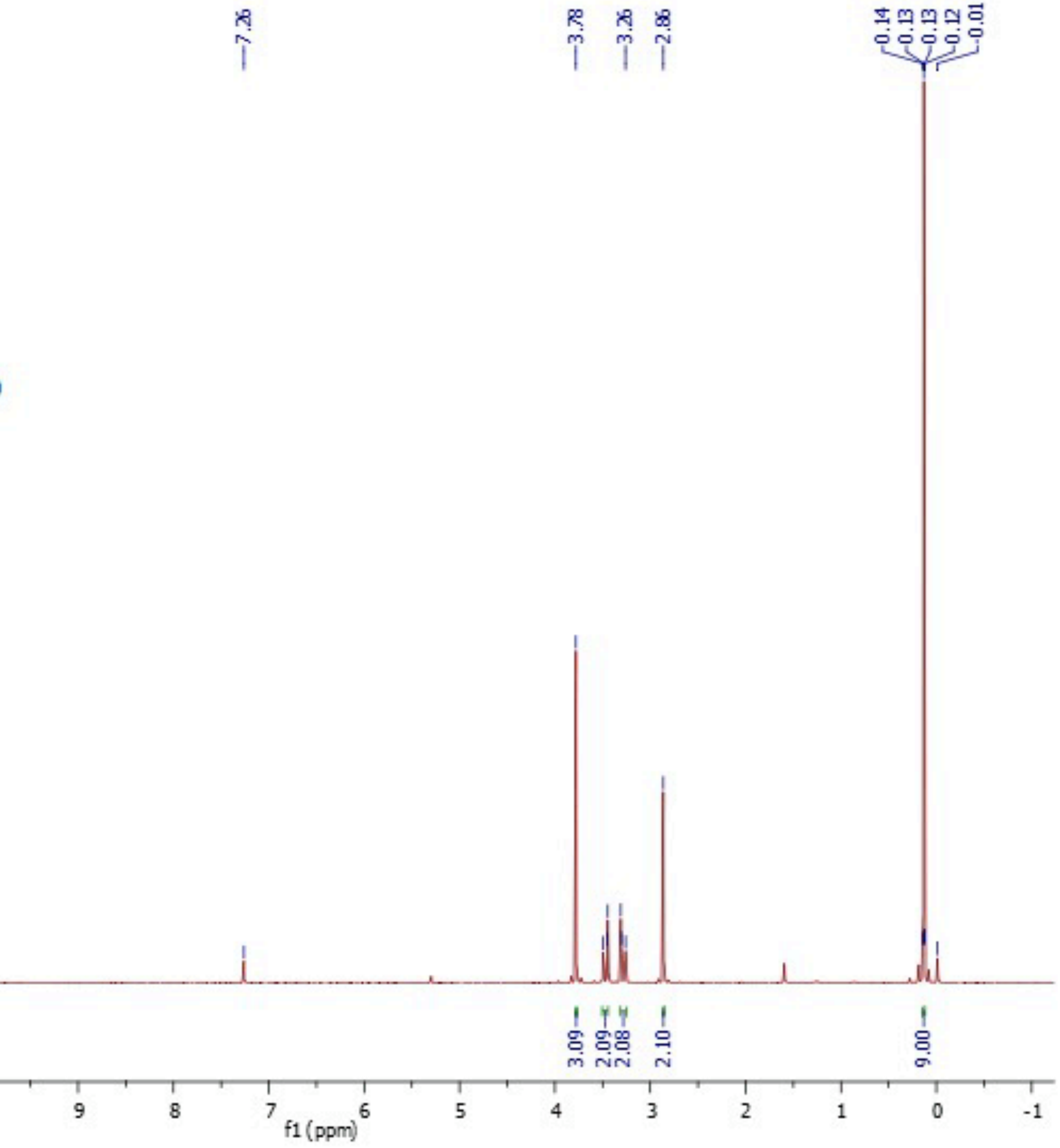

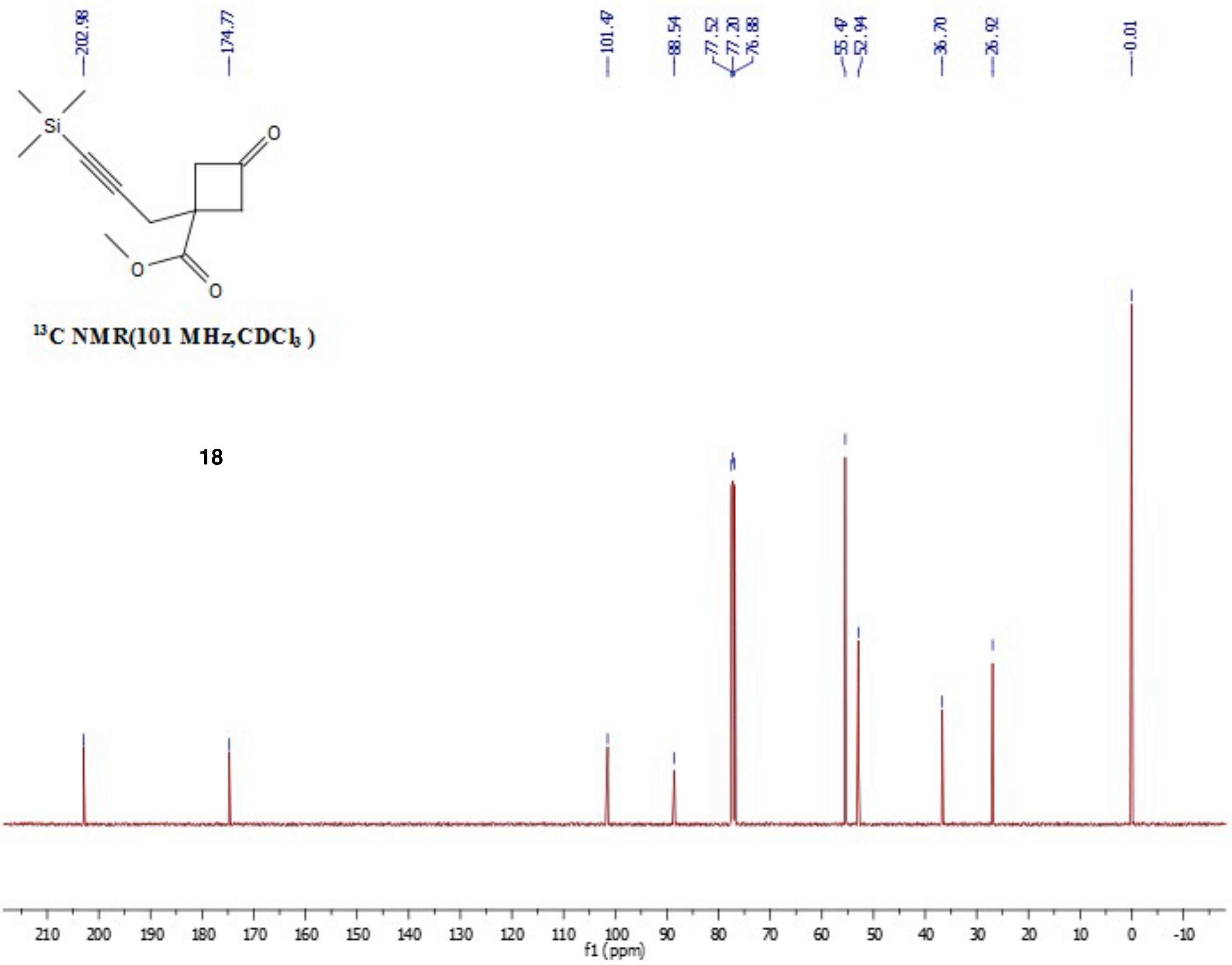

^{13}C NMR(101 MHz,$CDCl_3$)

18

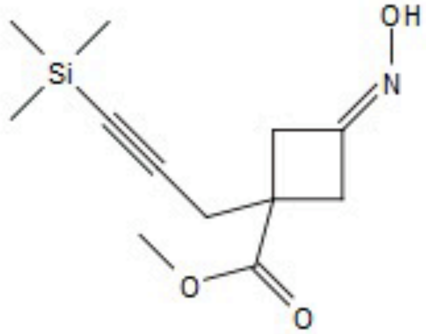

^1H NMR (400 MHz, DMSO-d6)

19

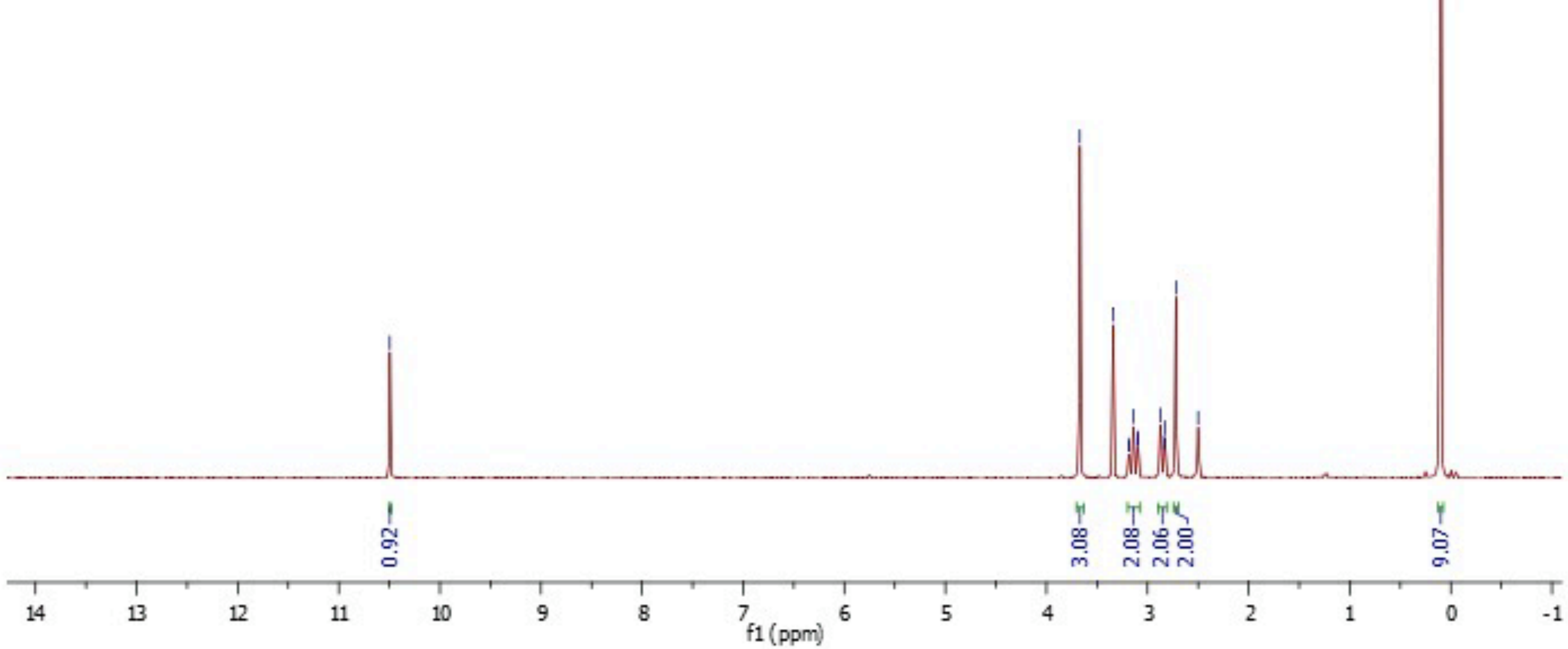

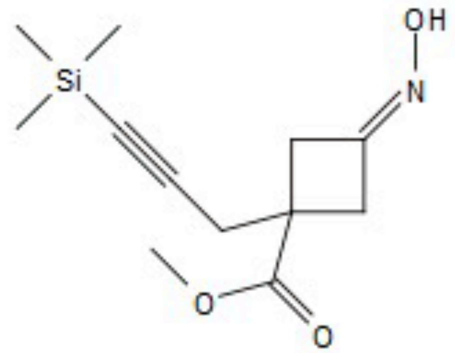

^{13}C NMR (101 MHz, DMSO-d6)

19

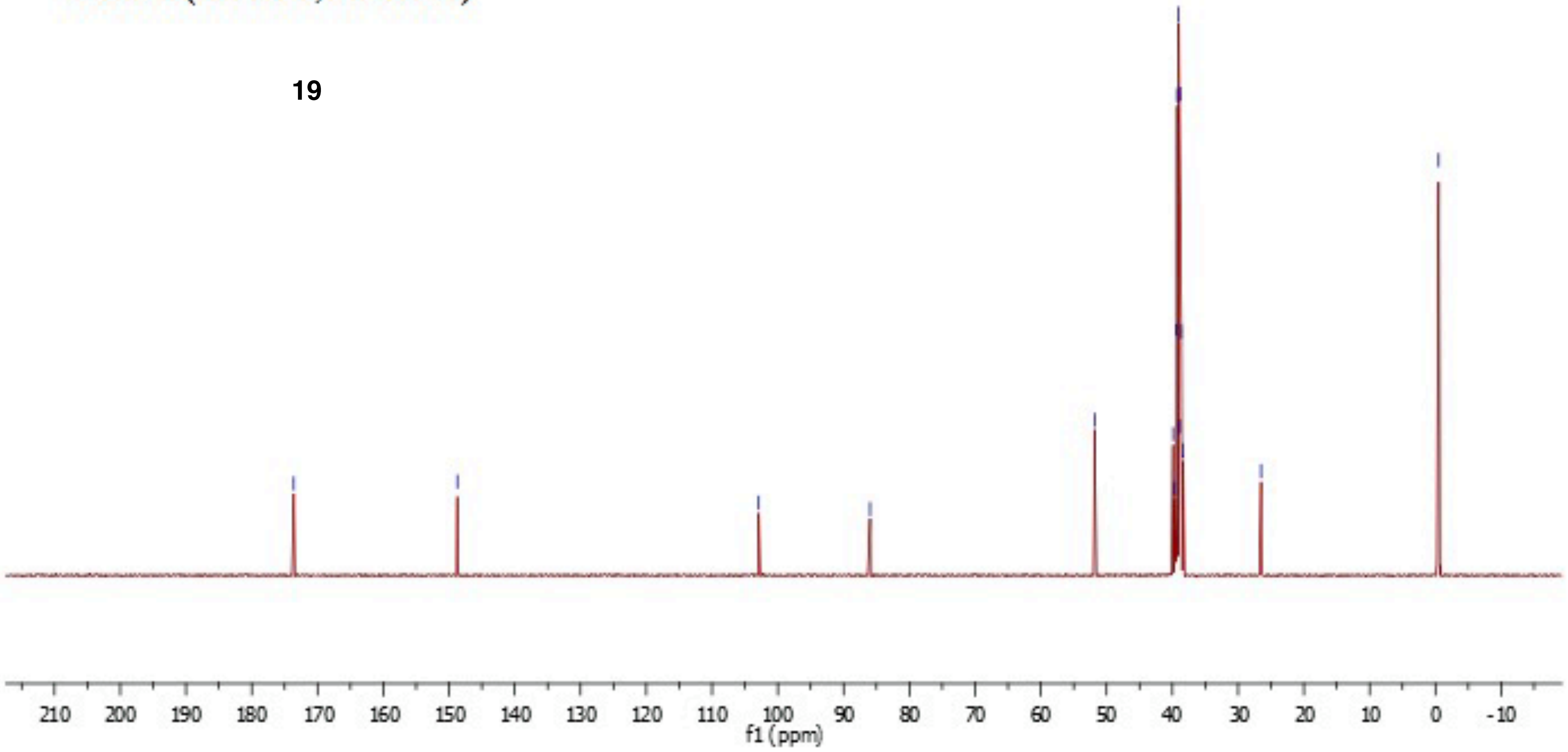

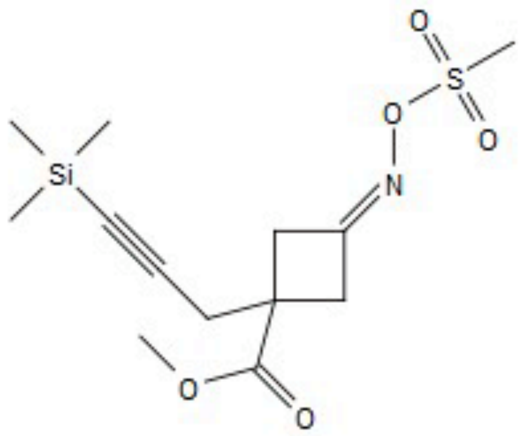

^1H NMR(400 MHz,$CDCl_3$)

20

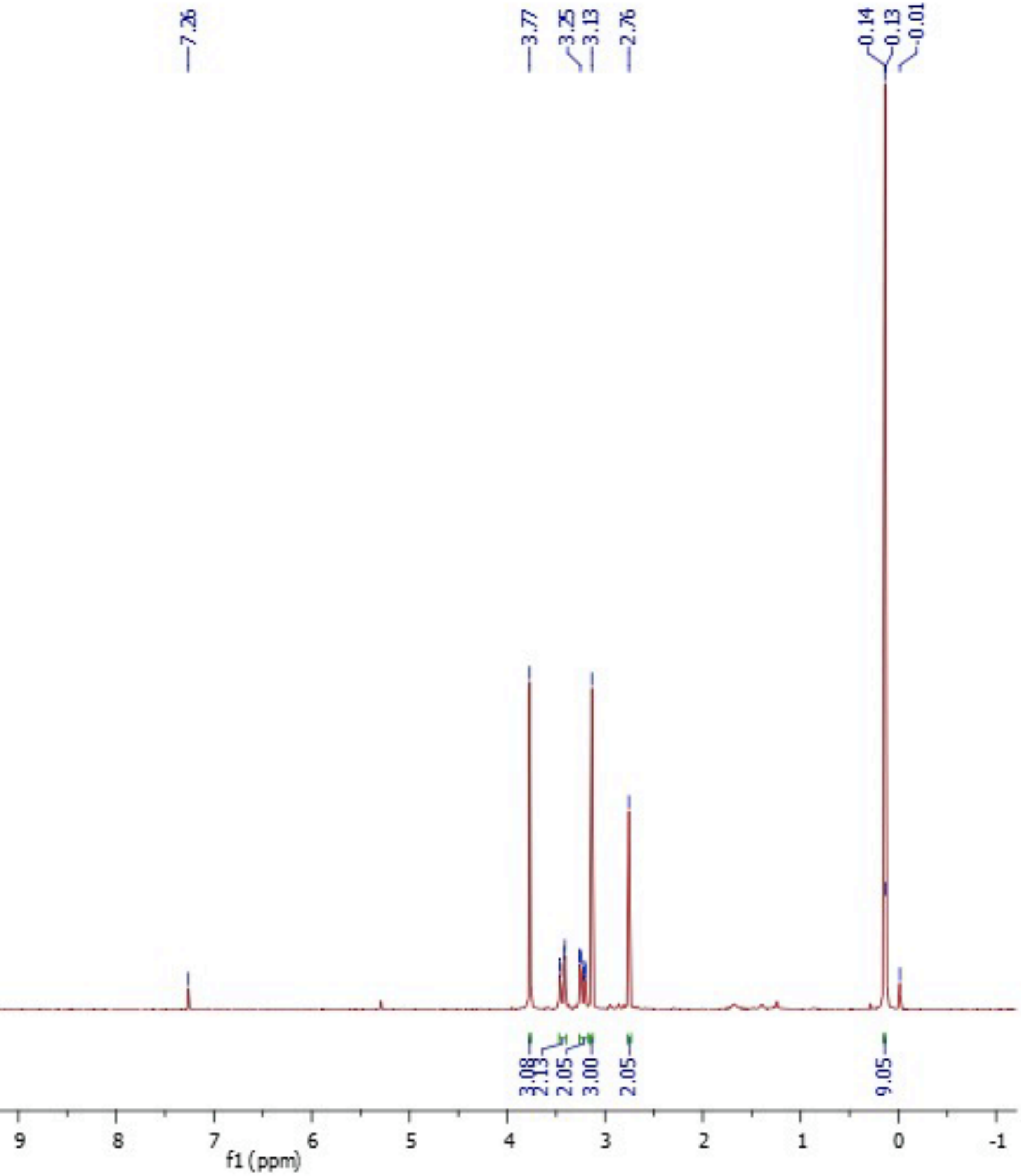

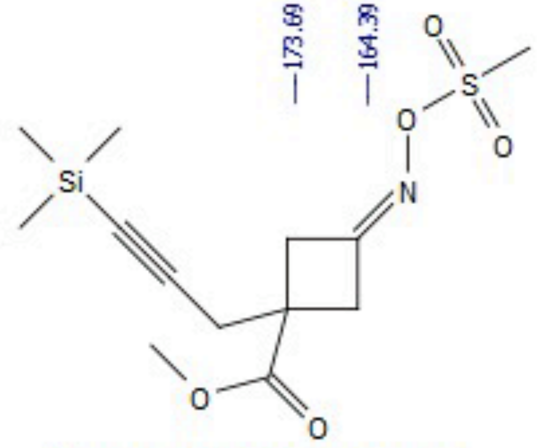

^{13}C NMR(101 MHz,CDCl$_3$)

20

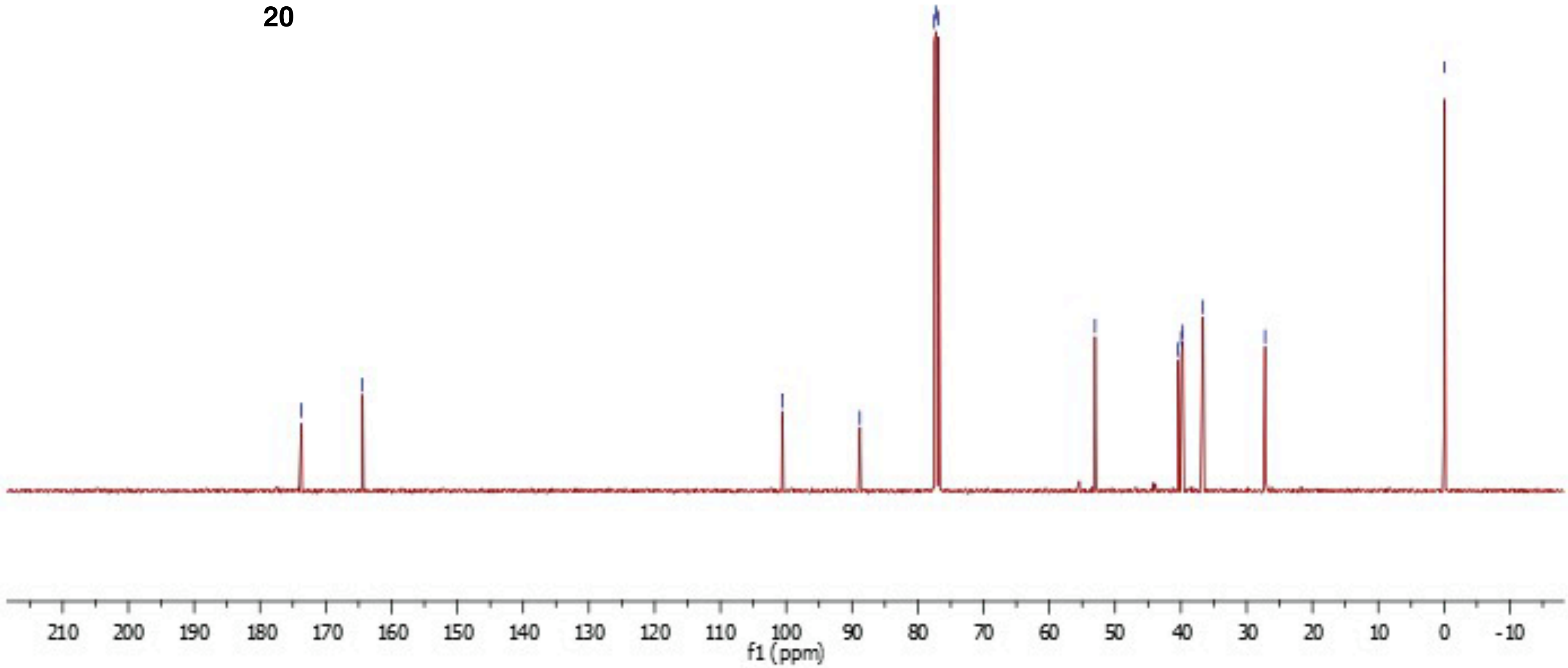

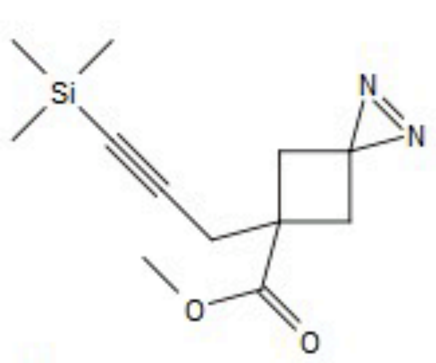

^1H NMR(400 MHz,$CDCl_3$)

21

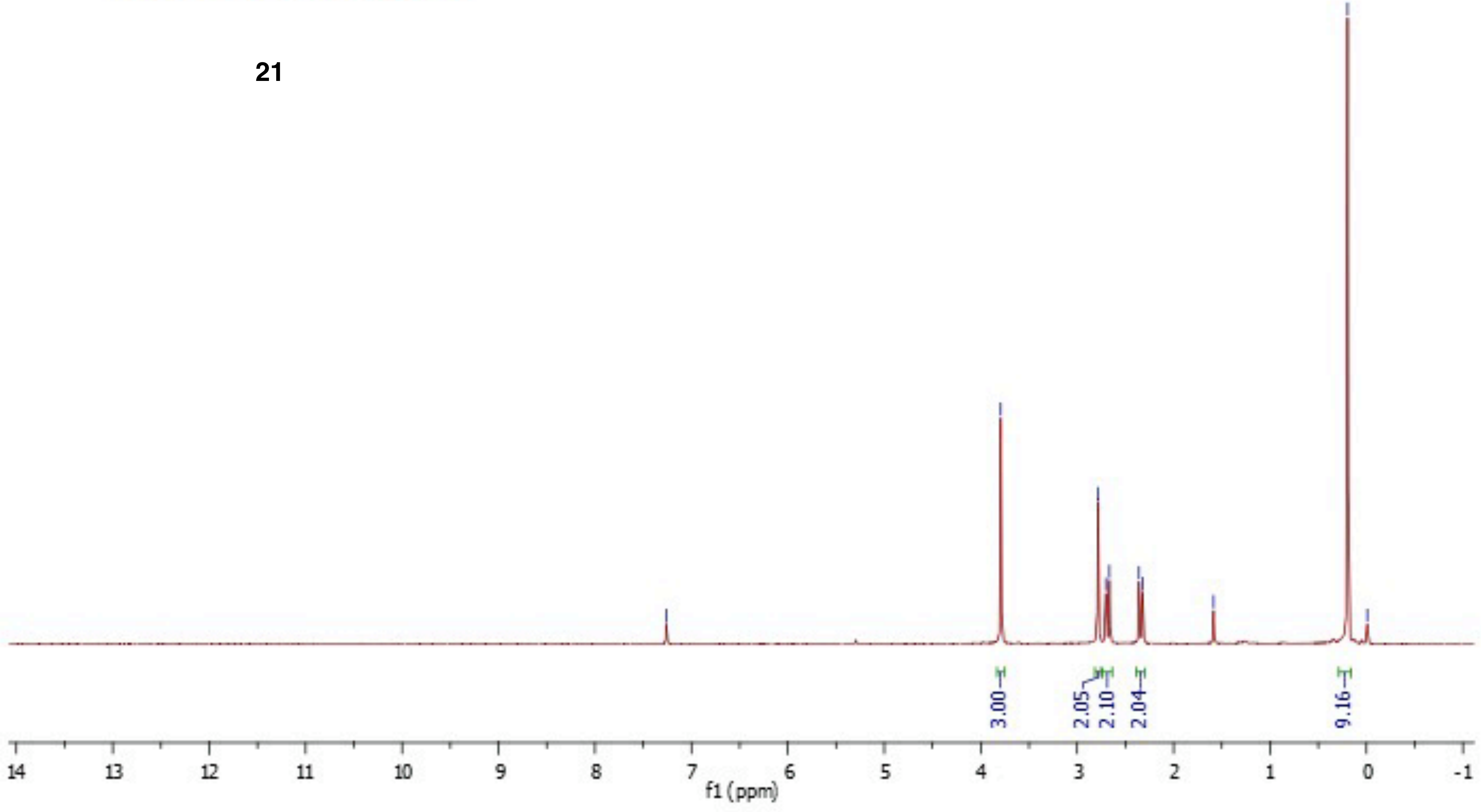

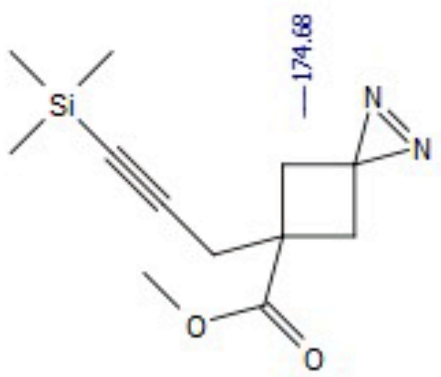

^{13}C NMR(101 MHz,$CDCl_3$)

21

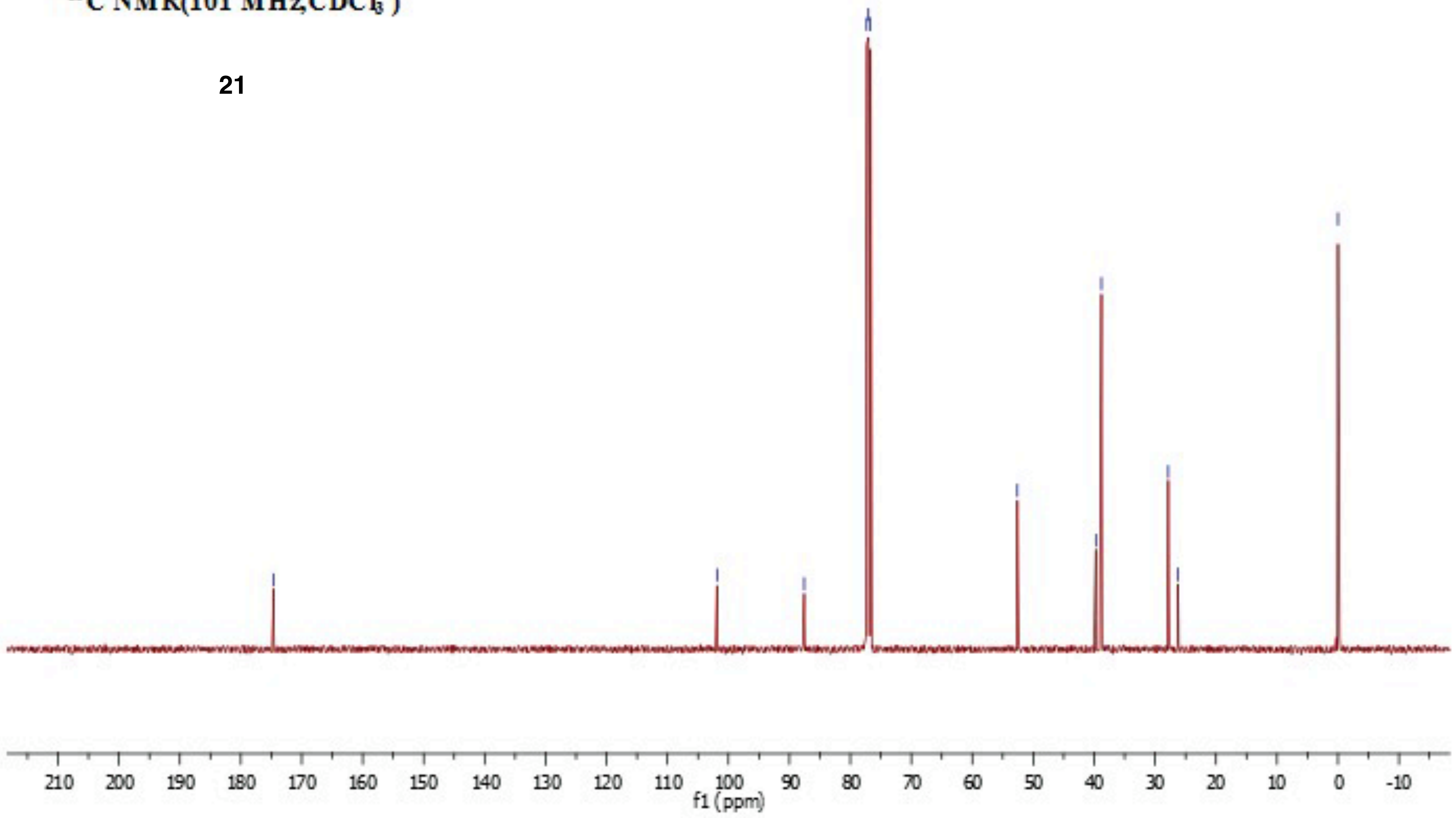

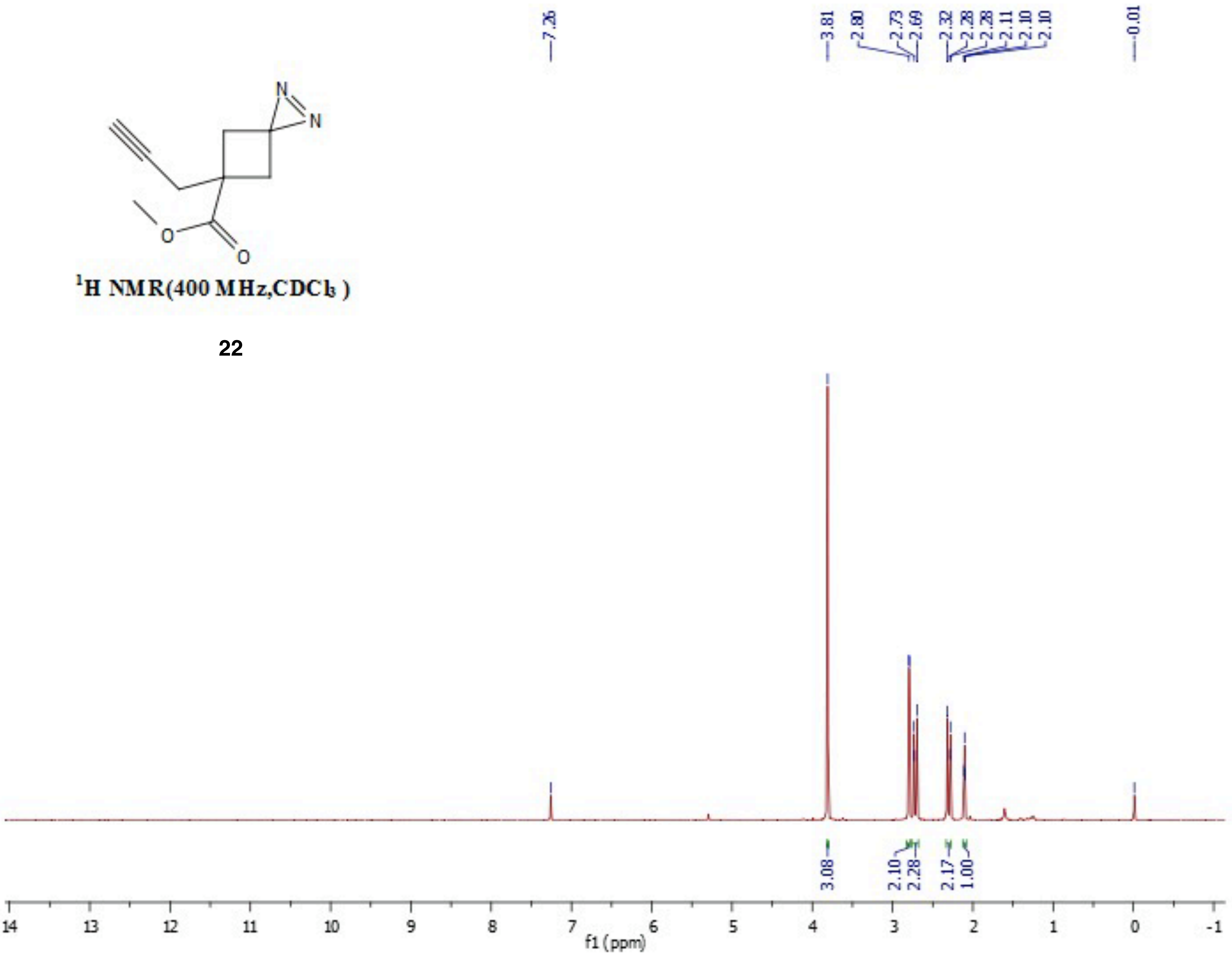

N
N
O
O
¹H NMR(400 MHz,CDCl₃)
22
7.26
3.81
2.80
2.73
2.69
2.32
2.28
2.28
2.11
2.10
2.10
-0.01
3.08
2.10
2.28
2.17
1.00
14
13
12
11
10
9
8
7
6
5
4
3
2
1
0
-1
f1 (ppm)

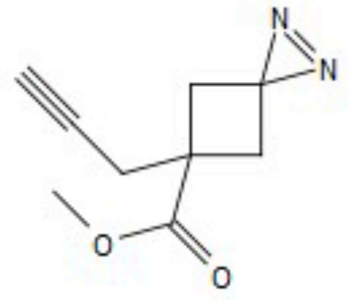

^{13}C NMR(101 MHz,CDCl$_3$)

22

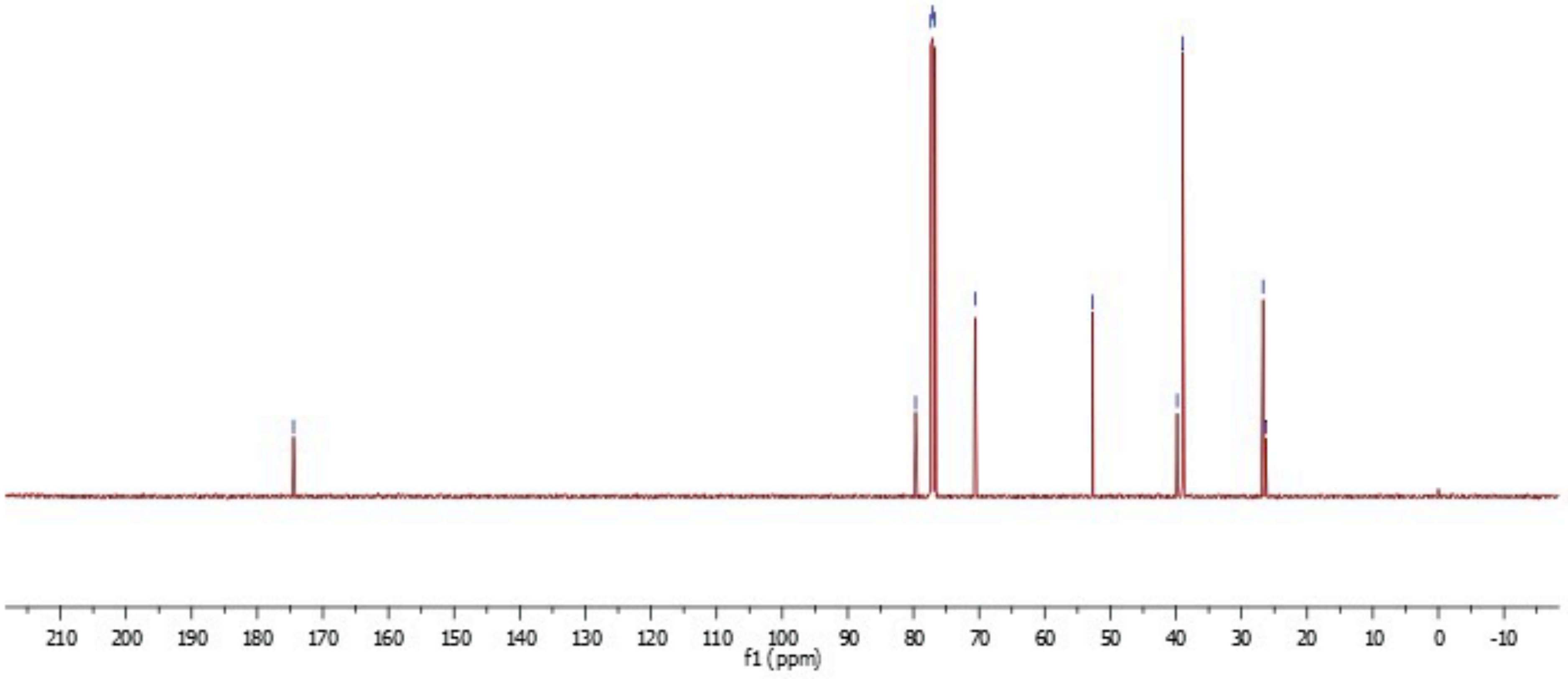

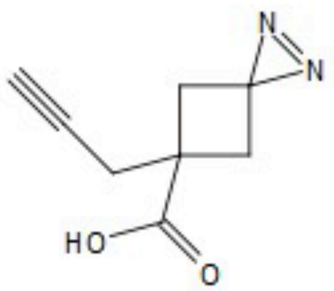

^1H NMR(400 MHz, $CDCl_3$)

23

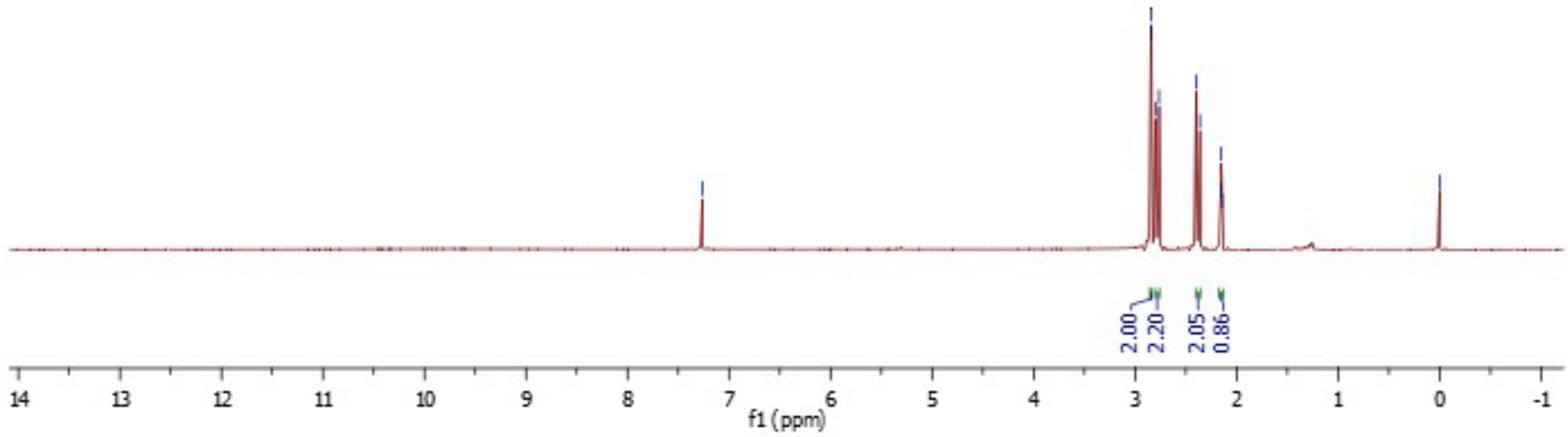

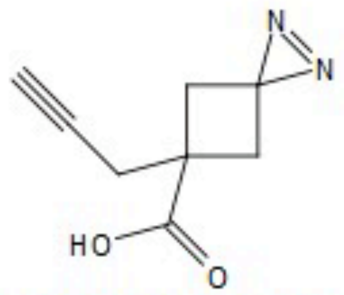

^{13}C NMR(101 MHz,$CDCl_3$)

23

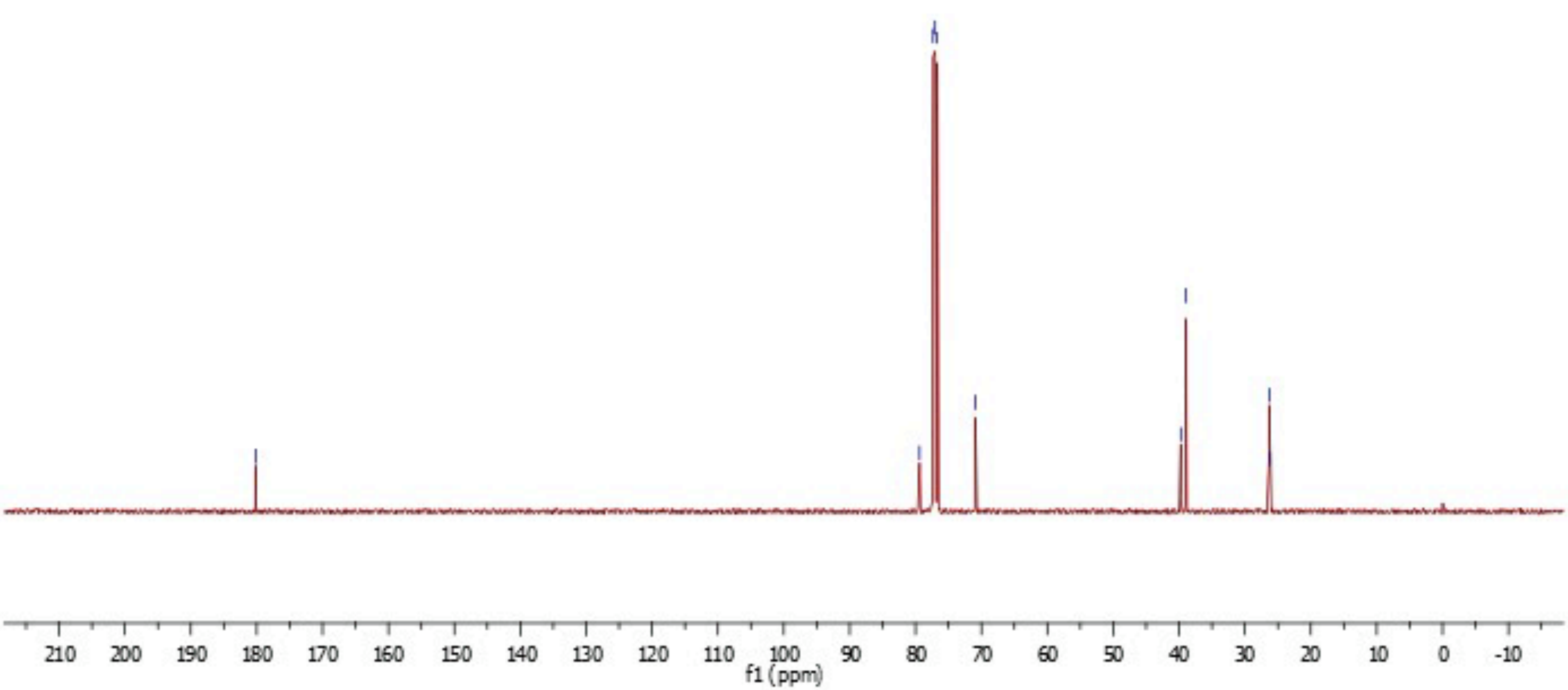

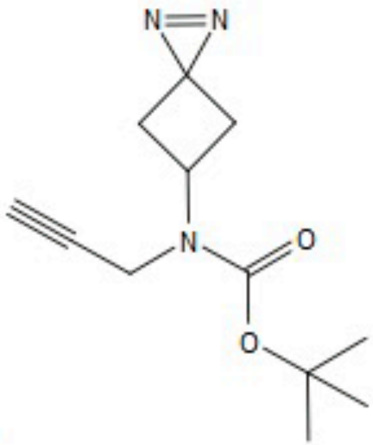

^{1}H NMR(400 MHz,$CDCl_3$)

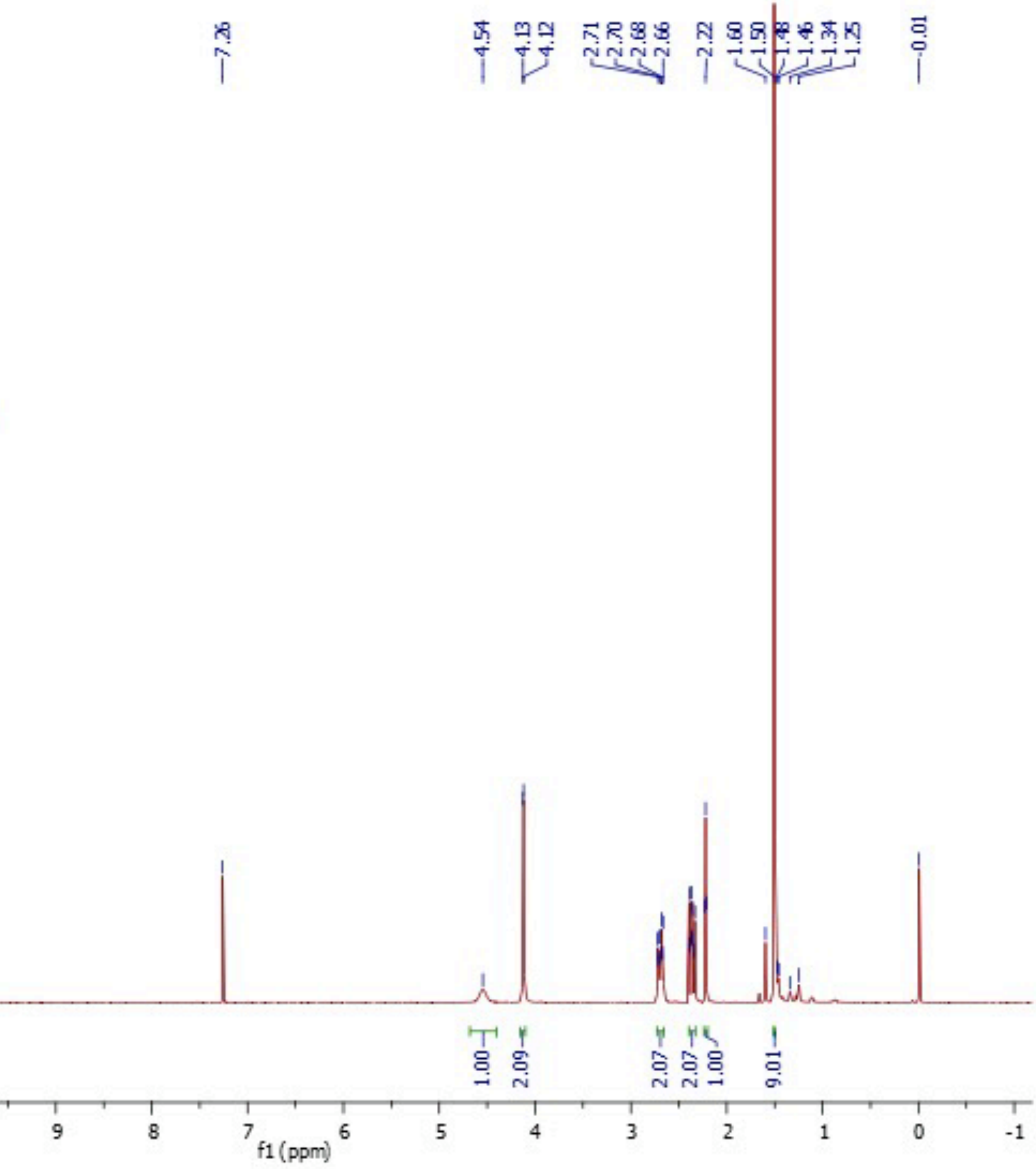

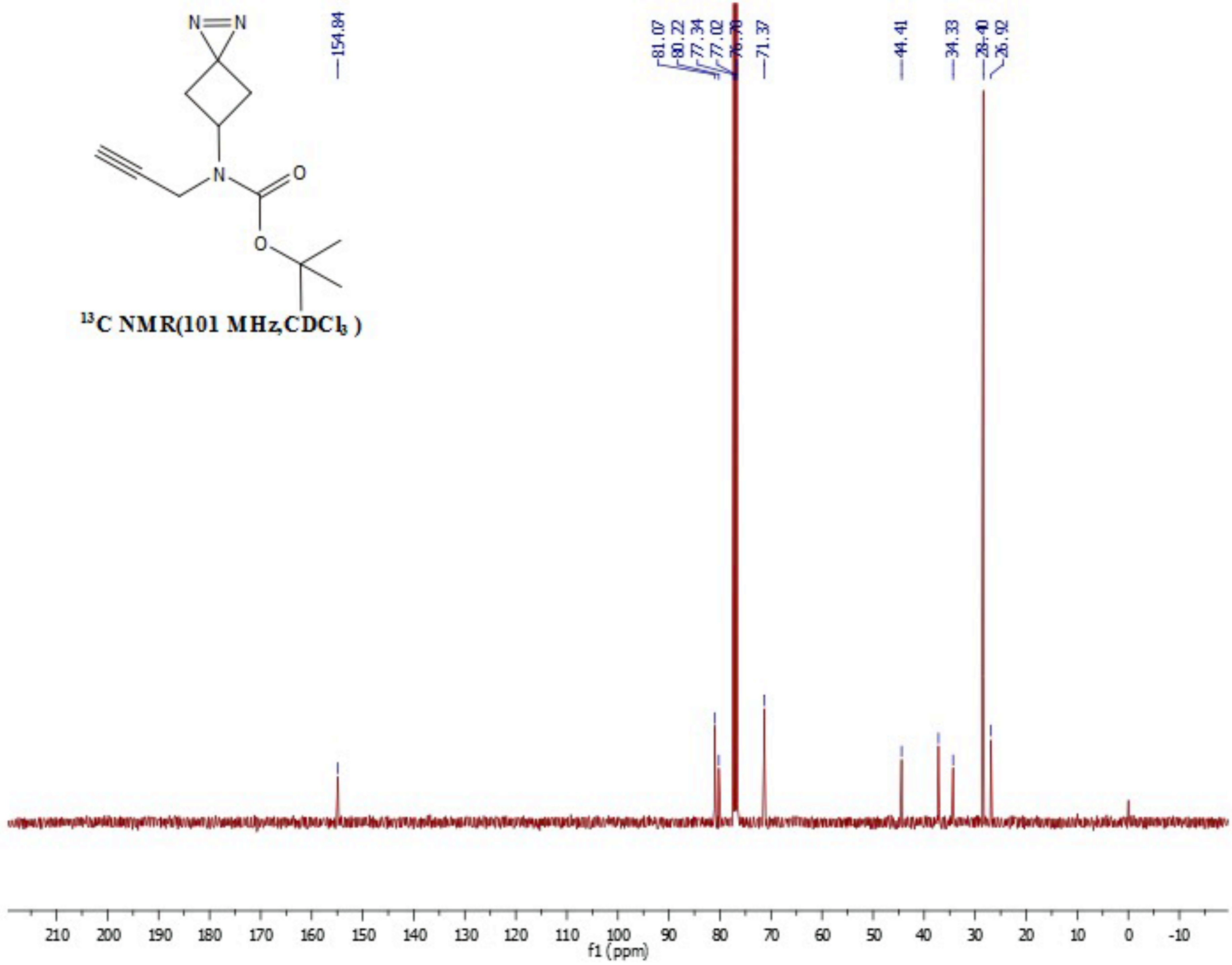

13C NMR(101 MHz,CDCl3)
154.84
81.07
80.22
77.34
77.02
76.70
71.37
44.41
34.33
28.40
26.92
210
200
190
180
170
160
150
140
130
120
110
100
90
80
70
60
50
40
30
20
10
0
-10
f1 (ppm)

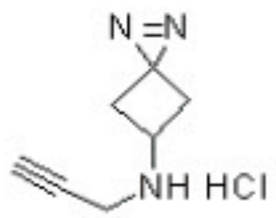

^{1}H NMR (400 MHz, DMSO-d6)

25

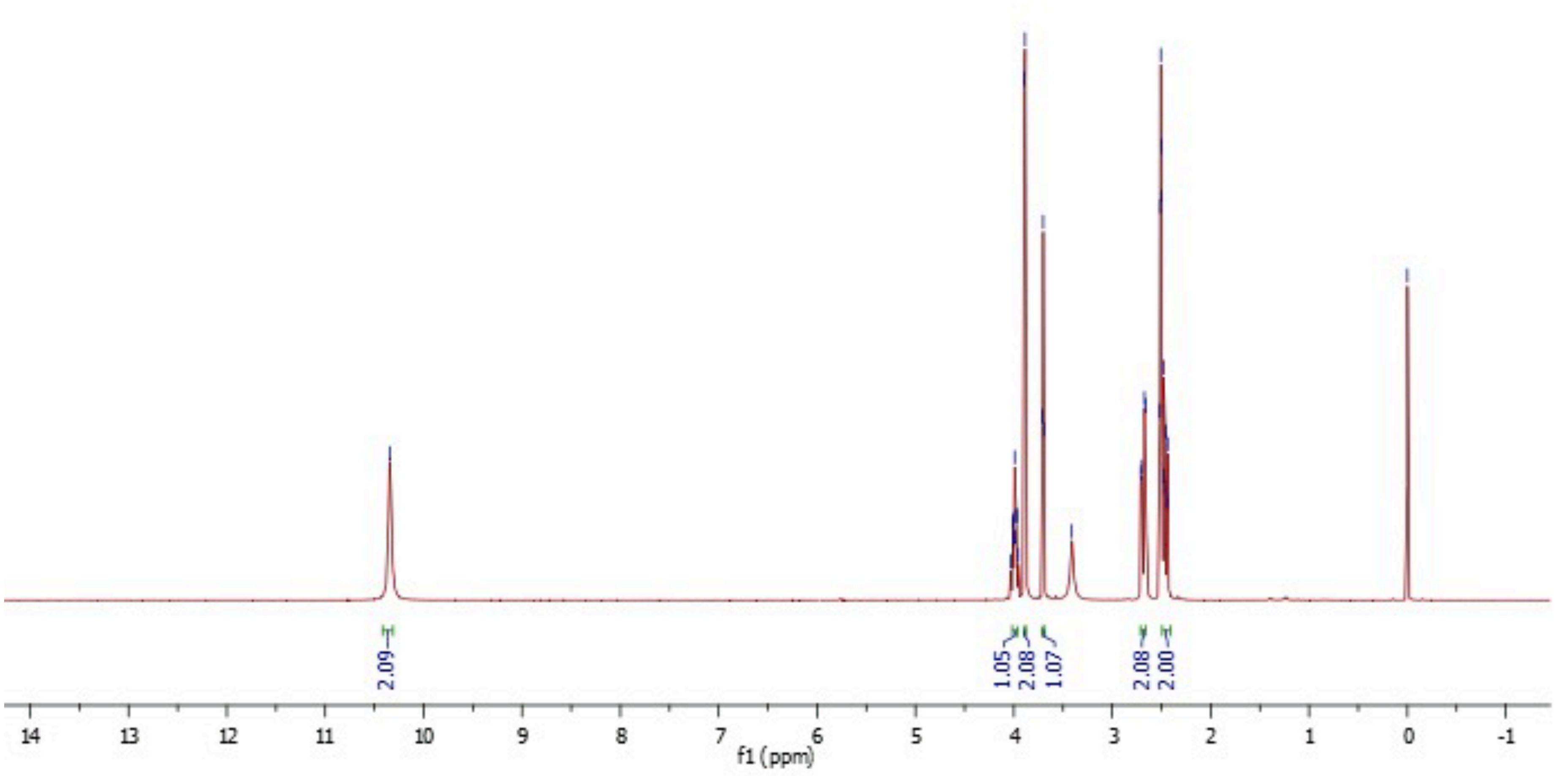

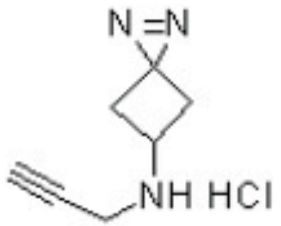

^{13}C NMR (101 MHz, DMSO-d6)

25

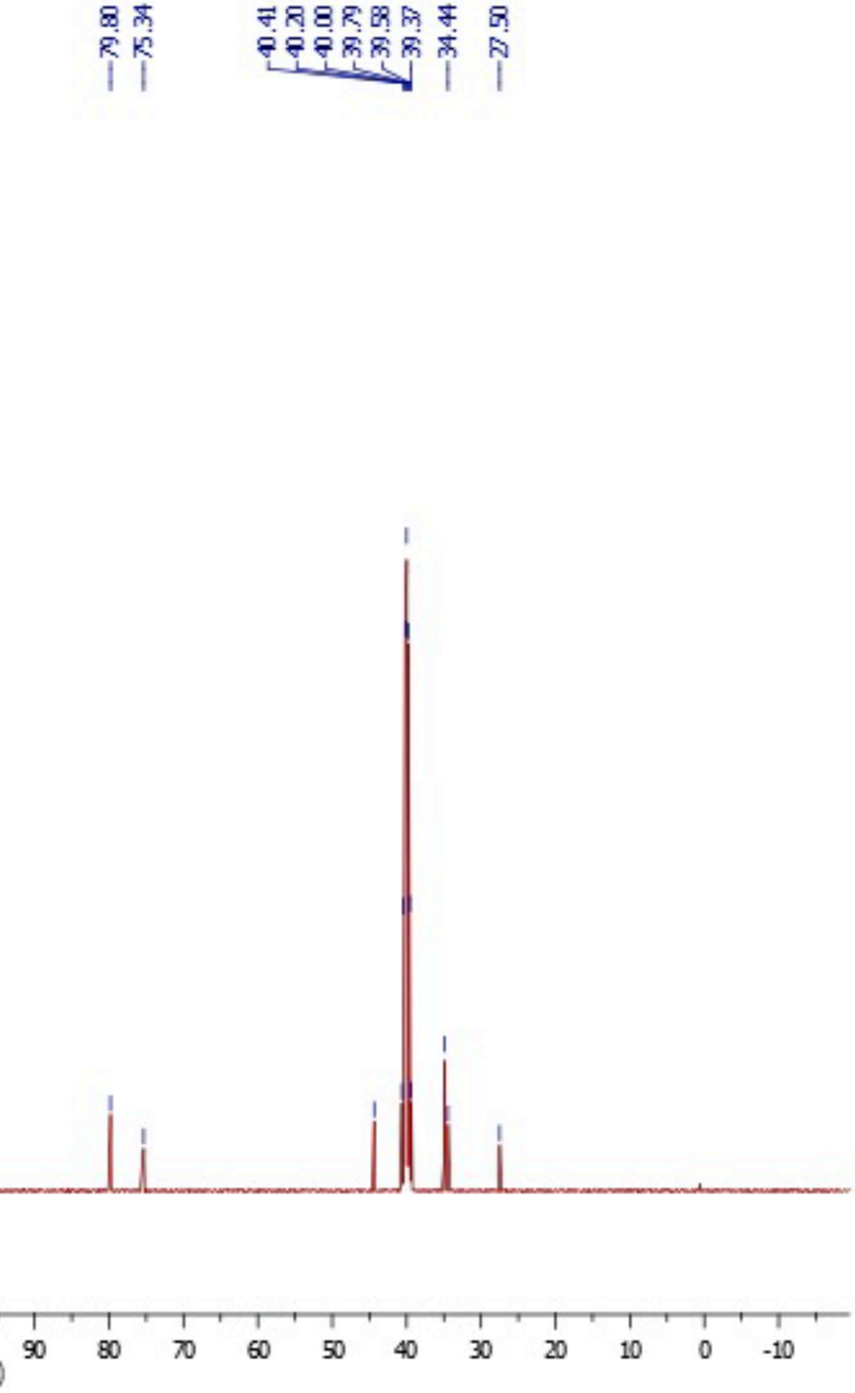

00110030-4016-001a.2.fid
NMR System BNMR_319-1 400 10206243
Pfizer Confidential
proton CD3CN {C:\Bruker\TopSpin3.5pl6} dutrajk 1

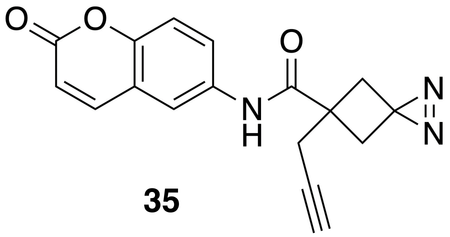

35

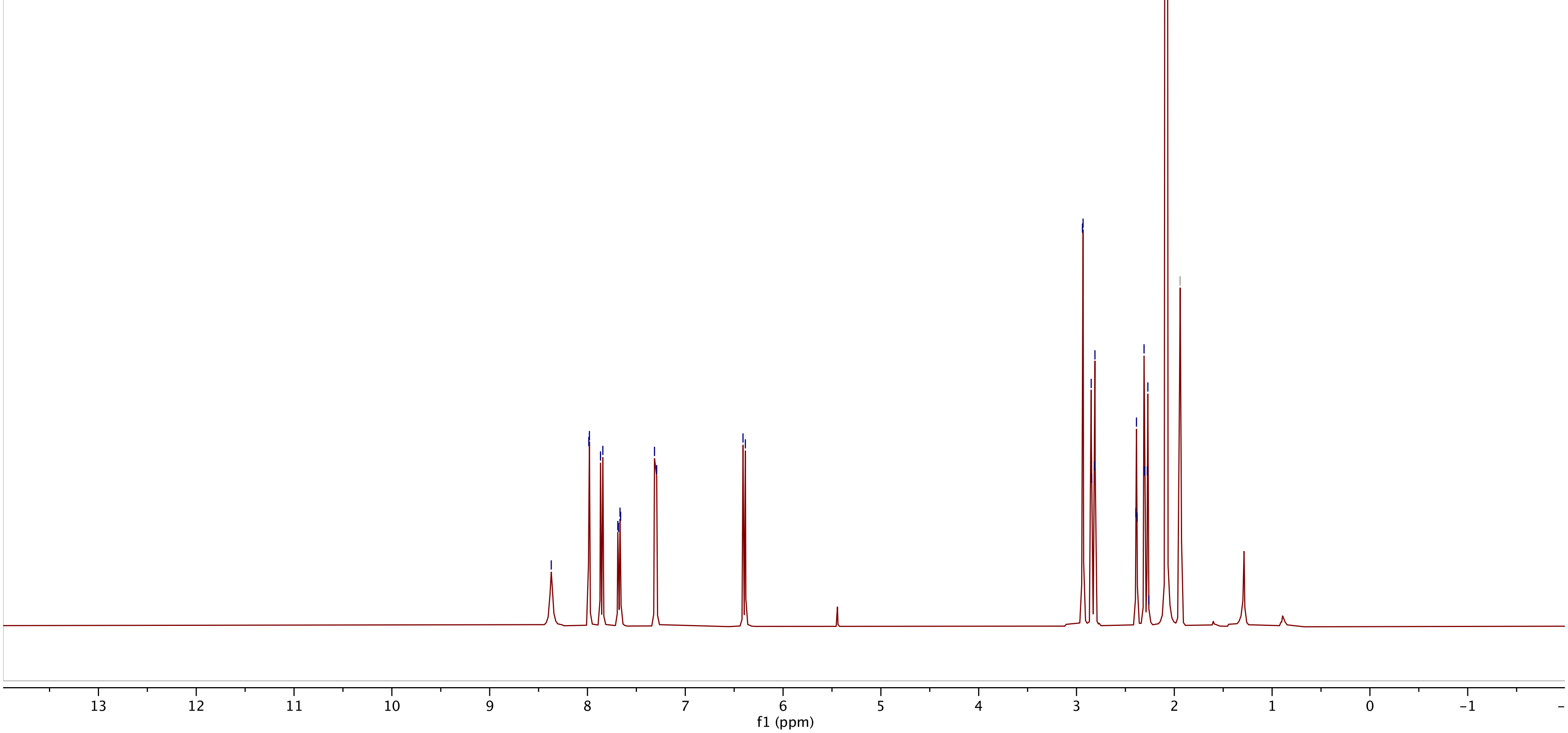

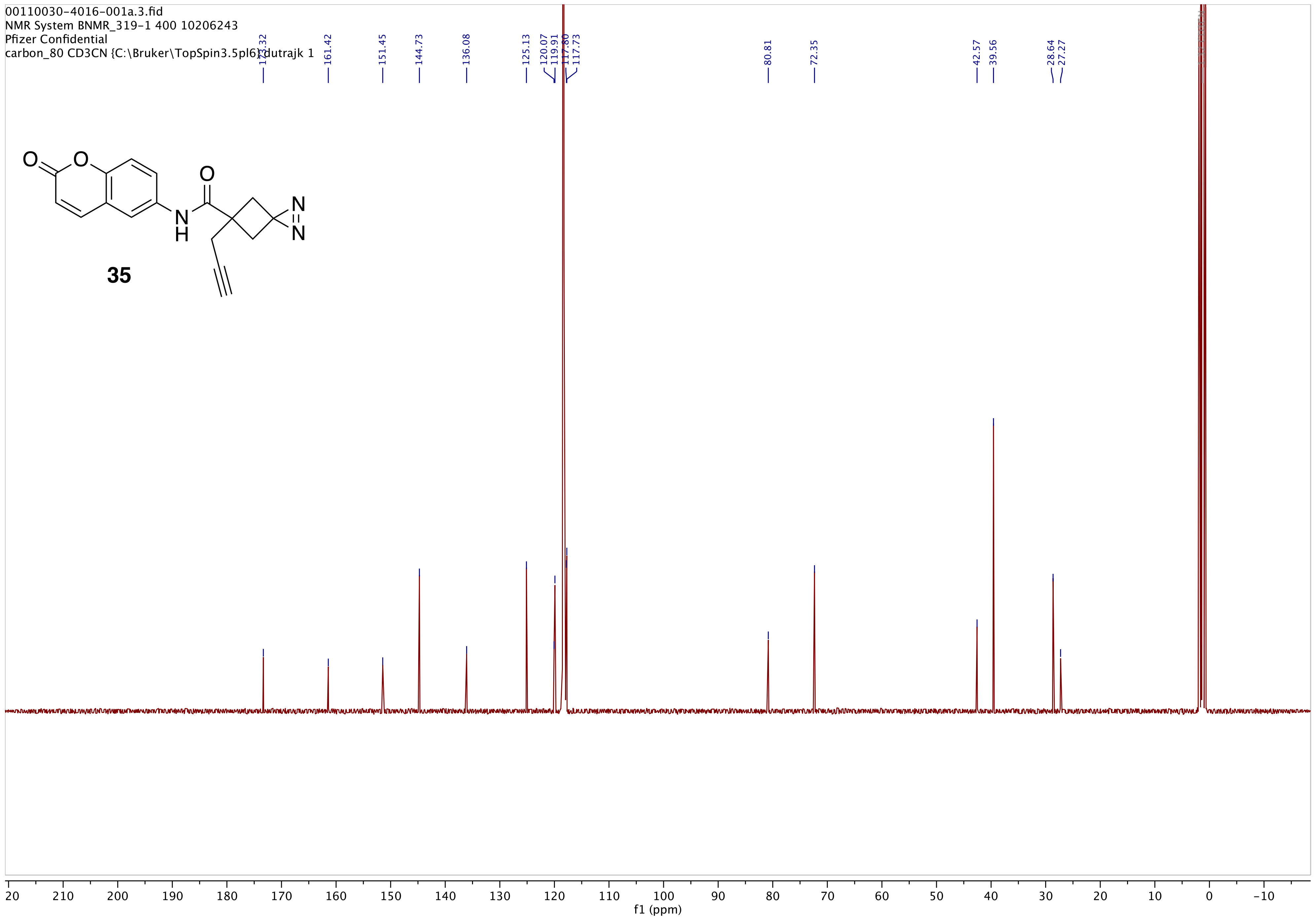
00110030-4016-001a.3.fid
NMR System BNMR_319-1 400 10206243
Pfizer Confidential
carbon_80 CD3CN {C:\Bruker\TopSpin3.5pl6}dutrajk 1
173.32
161.42
151.45
144.73
136.08
125.13
120.07
119.91
117.80
117.73
80.81
72.35
42.57
39.56
28.64
27.27
f1 (ppm)

35

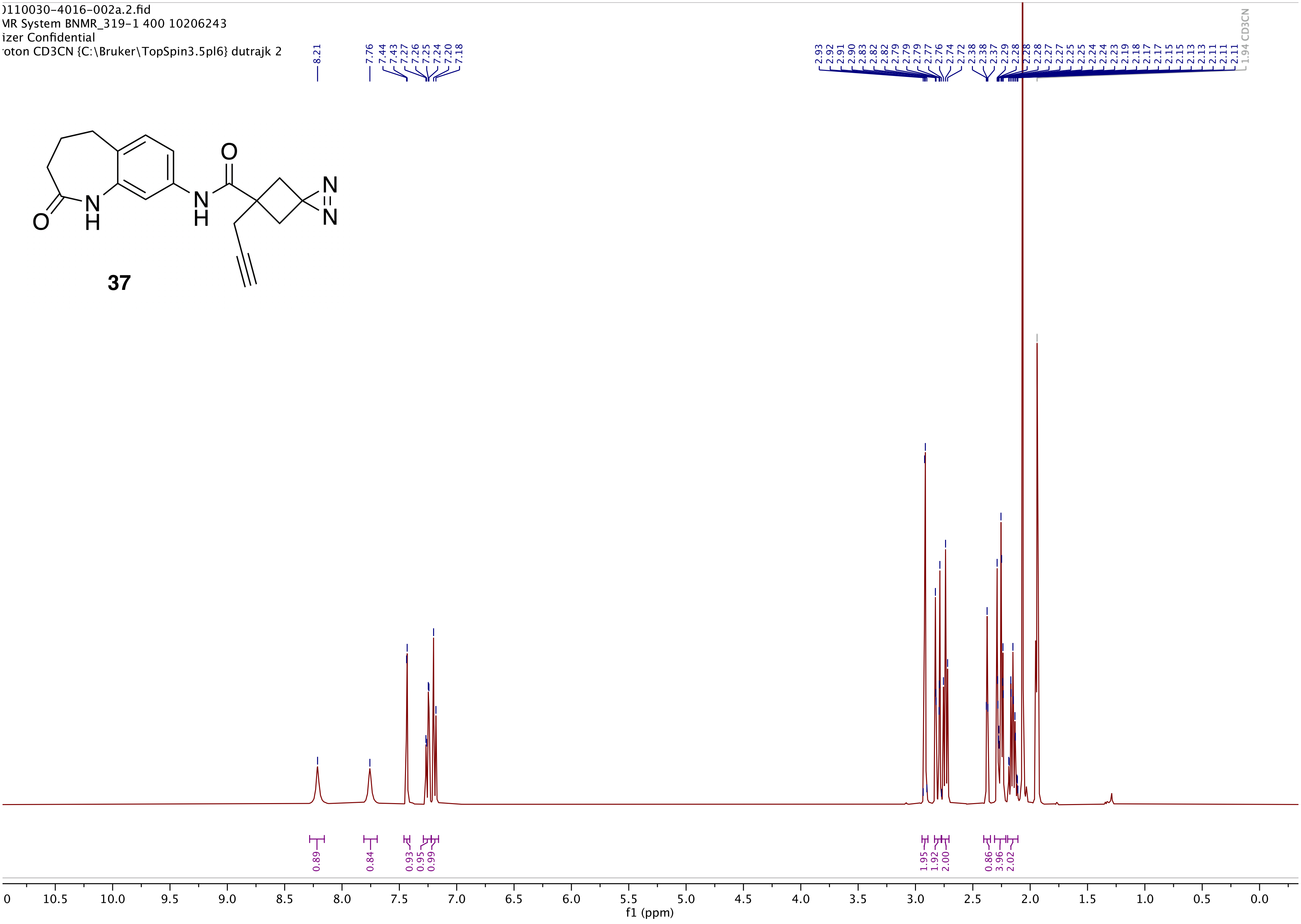
)110030-4016-002a.2.fid
VIR System BNMR_319-1 400 10206243
izer Confidential
roton CD3CN {C:\Bruker\TopSpin3.5pl6} dutrajk 2
8.21
7.76
7.44
7.43
7.27
7.26
7.25
7.24
7.20
7.18
2.93
2.92
2.91
2.90
2.83
2.82
2.82
2.79
2.79
2.79
2.77
2.76
2.74
2.72
2.38
2.38
2.37
2.29
2.28
2.28
2.28
2.27
2.27
2.25
2.25
2.24
2.24
2.23
2.19
2.18
2.17
2.17
2.15
2.15
2.13
2.13
2.11
2.11
2.11
1.94 CD3CN
O
N
H
N
H
O
N
N
0.89
0.84
0.93
0.95
0.99
1.95
1.92
2.00
0.86
3.96
2.02
f1 (ppm)

37

00110030-4016-002a.3.fid
NMR System BNMR_319-1 400 10206243
Pfizer Confidential
carbon_80 CD3CN {C:\Bruker\TopSpin3.5pl[illegible]\dutrajk 2

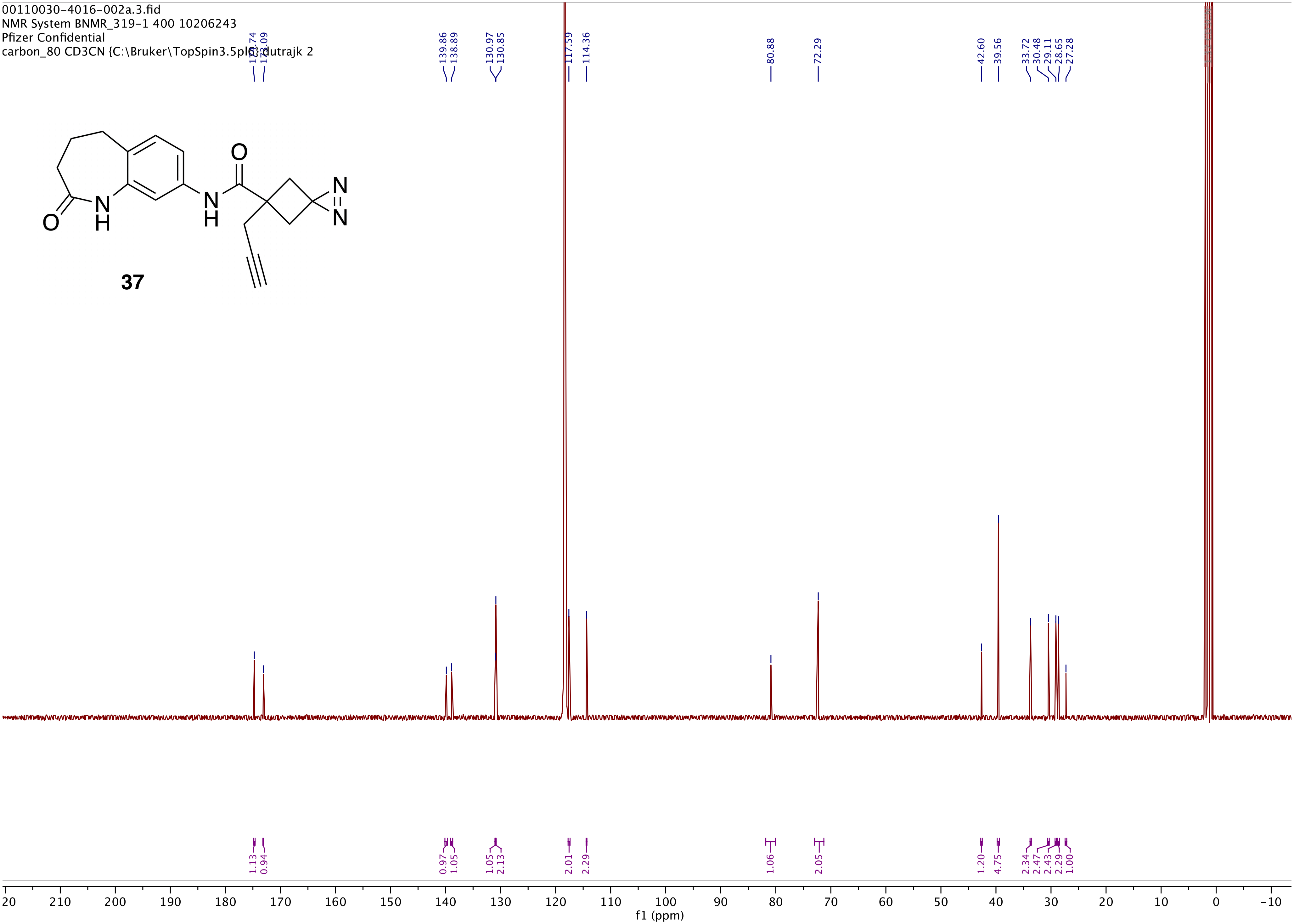

37

^{1}H NMR of **38** (400 MHz, $CDCl_3$)

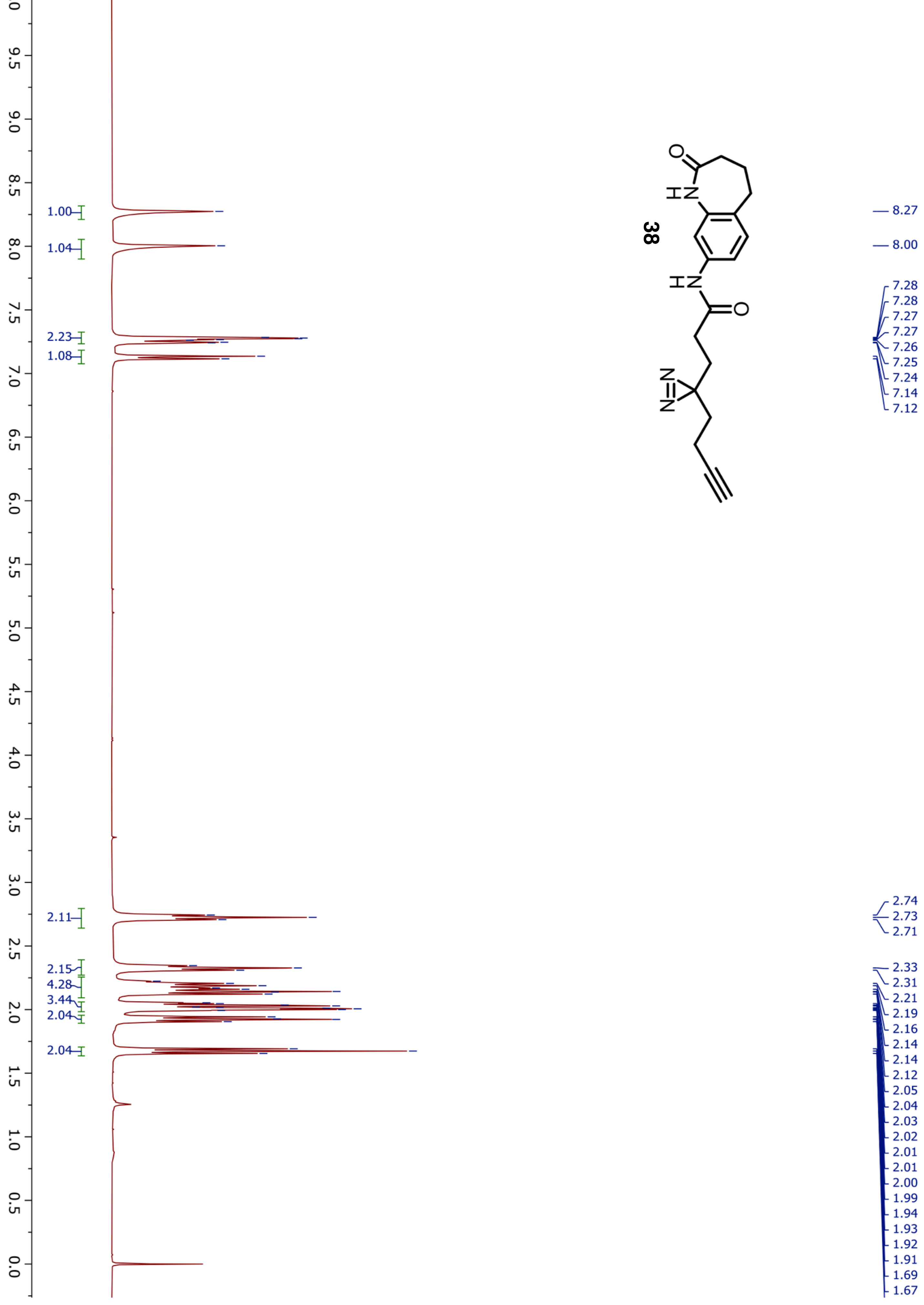

^{13}C NMR of **38** (101 MHz, $CDCl_3$)

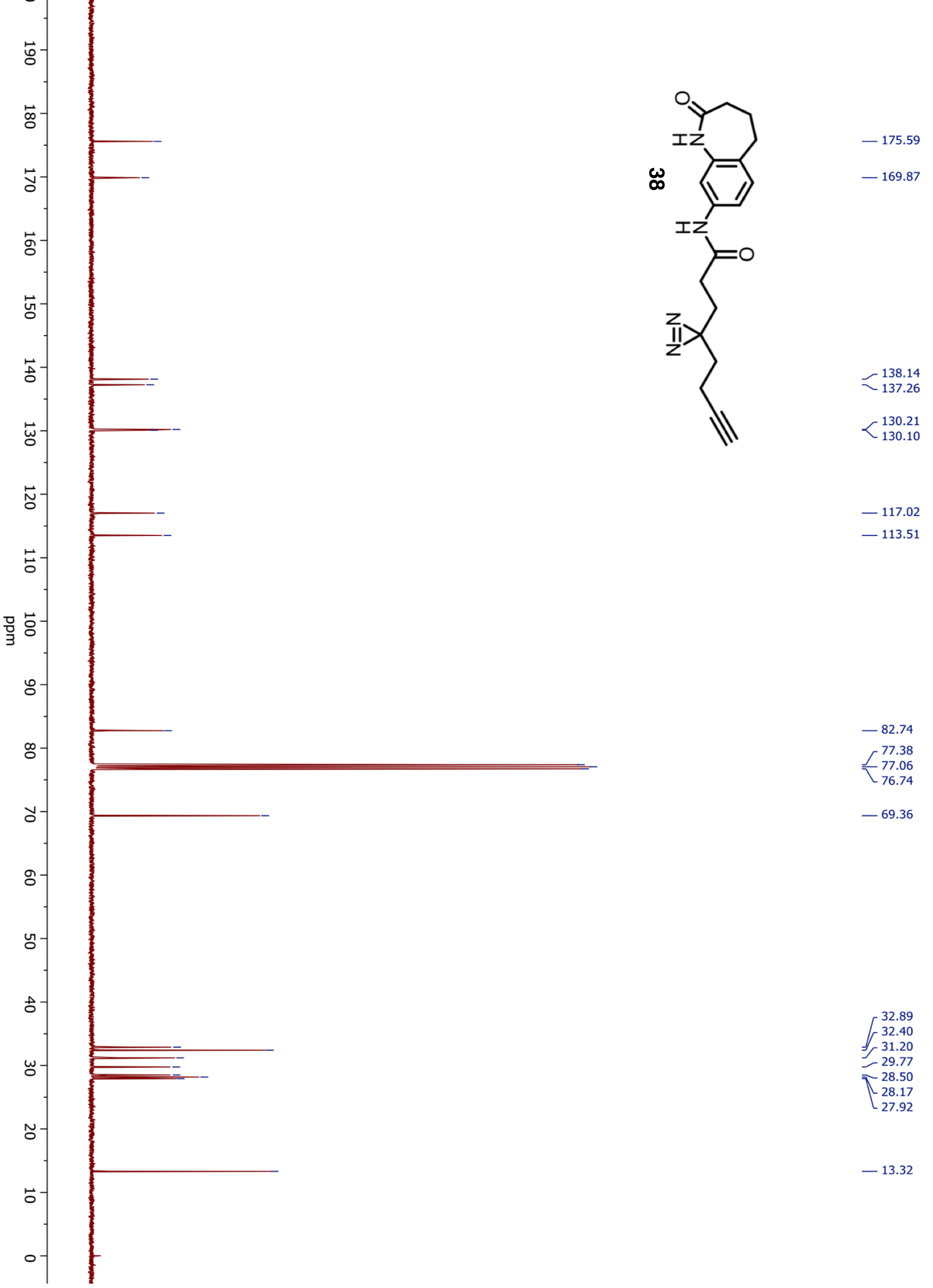

0110030-4016-003.3.fid
IMR System BNMR_319-1 400 10206243
fizer Confidential
roton CD3CN {C:\Bruker\TopSpin3.5pl6} dutrajk 3

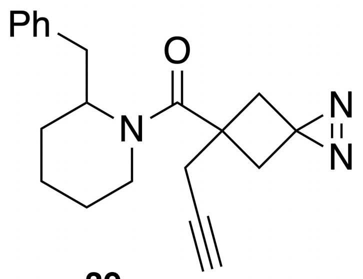

39

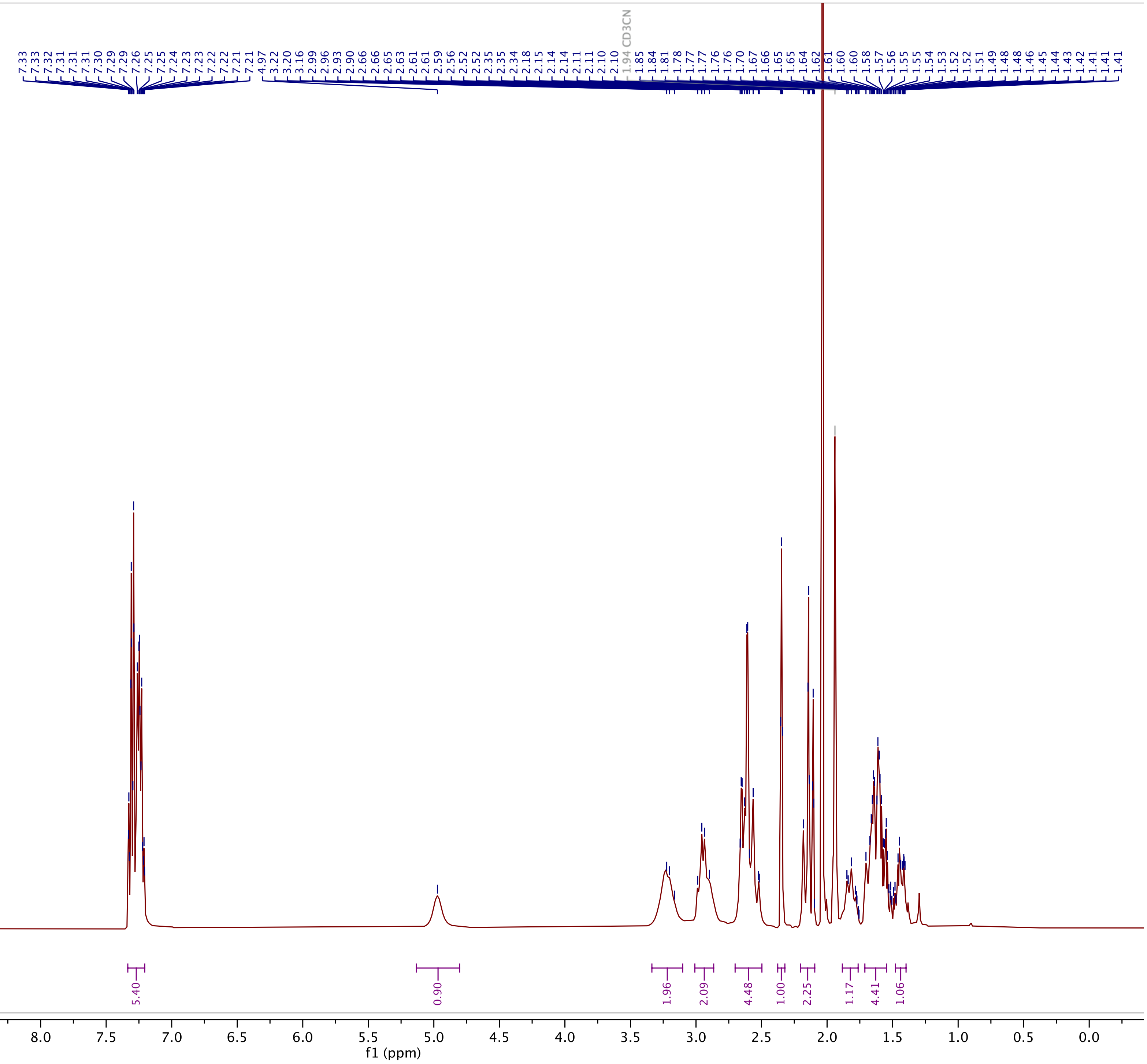

0110030-4016-003.4.fid
MR System BNMR_319-1 400 10206243
ïzer Confidential
ırbon_80 CD3CN {C:\Bruker\TopSpin3.5pl6} dutrajk 3

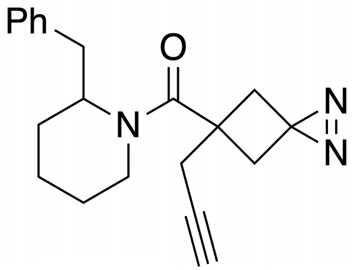

39

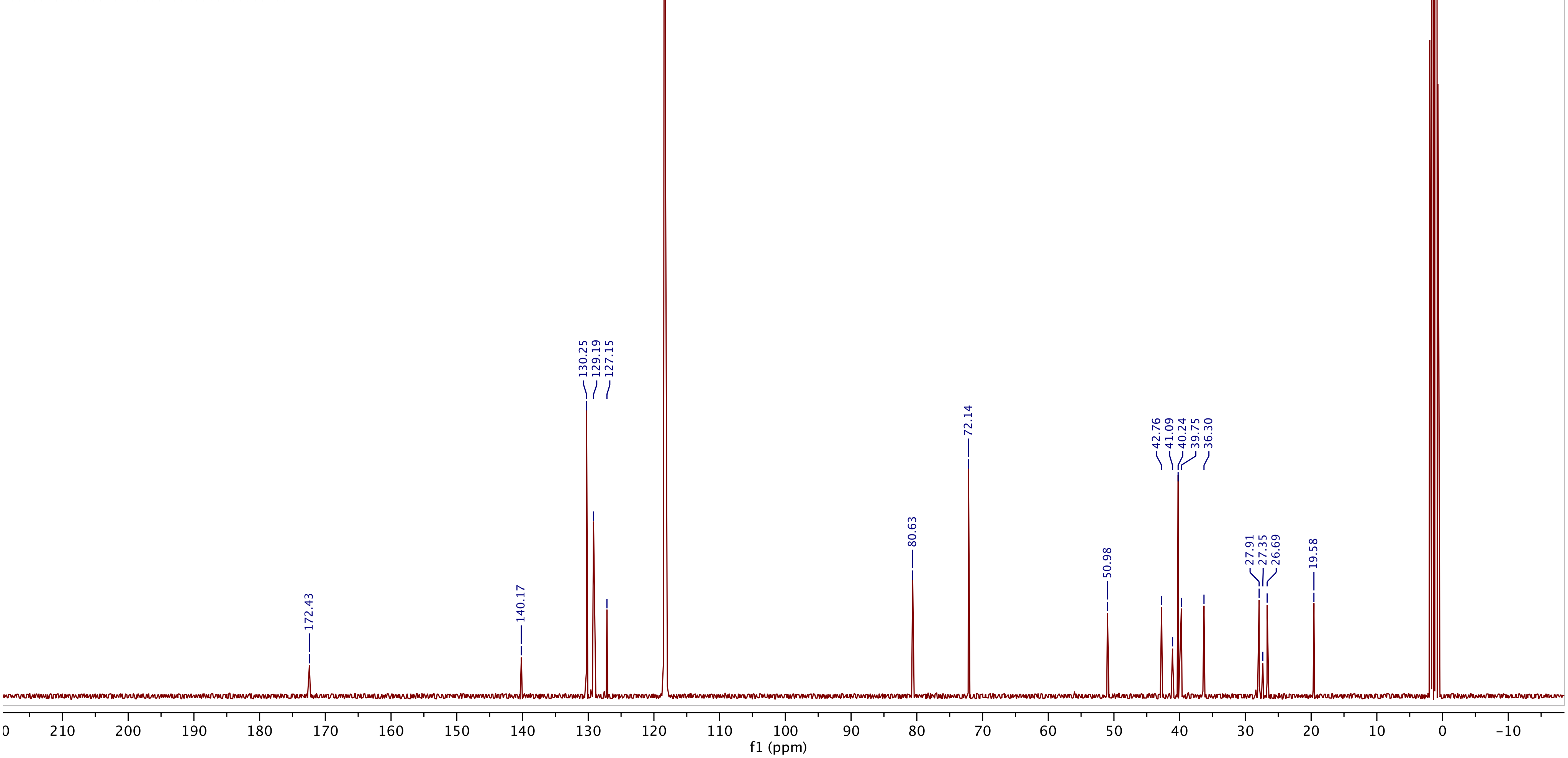

00110030-4015-001.3.fid
NMR System BNMR_319-1 400 10206243
Pfizer Confidential
proton CD3CN {C:\Bruker\TopSpin3.5pl6} dutrajk 10

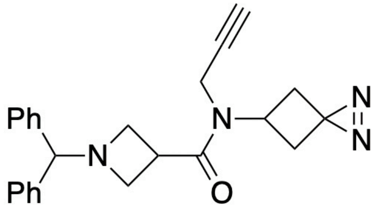

41

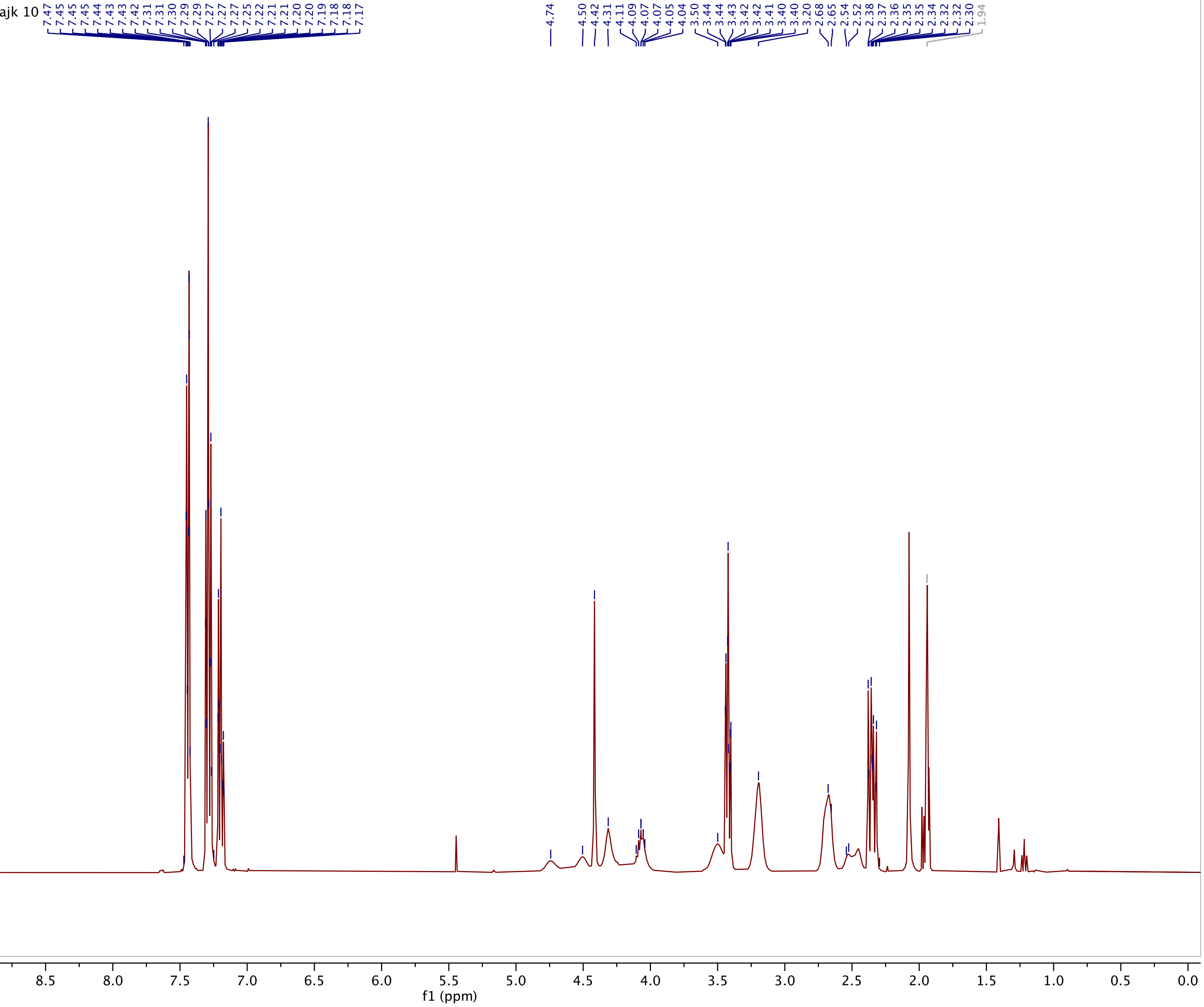

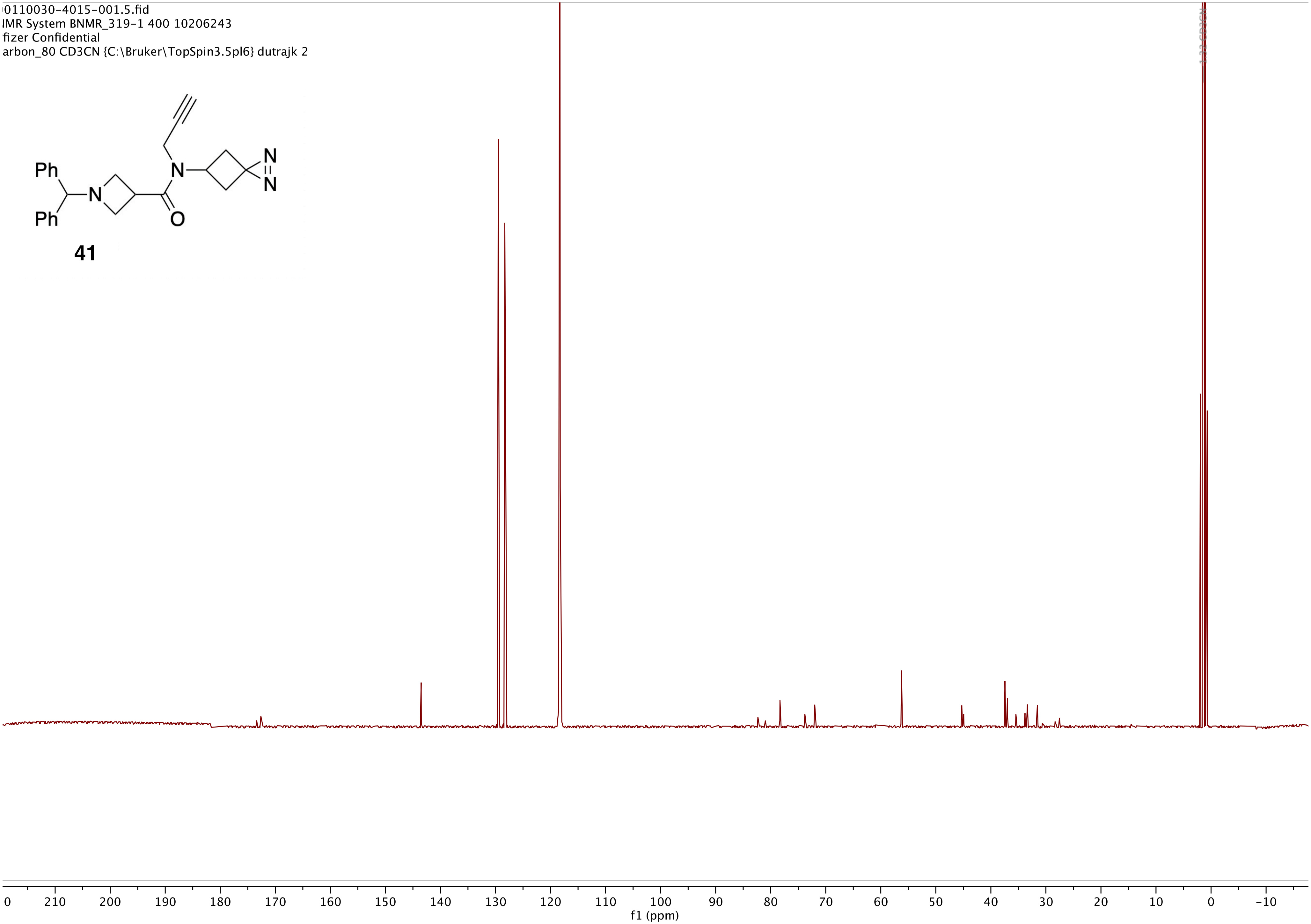

0110030-4015-001.5.fid
IMR System BNMR_319-1 400 10206243
fizer Confidential
arbon_80 CD3CN {C:\Bruker\TopSpin3.5pl6} dutrajk 2
41
0
210
200
190
180
170
160
150
140
130
120
110
100
90
80
70
60
50
40
30
20
10
0
−10
f1 (ppm)

00110030-4016-006.5.fid
NMR System BNMR_319-1 400 10206243
Pfizer Confidential
proton CD3CN {C:\Bruker\TopSpin3.5pl6} dutrajk 6

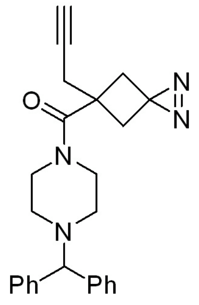

44

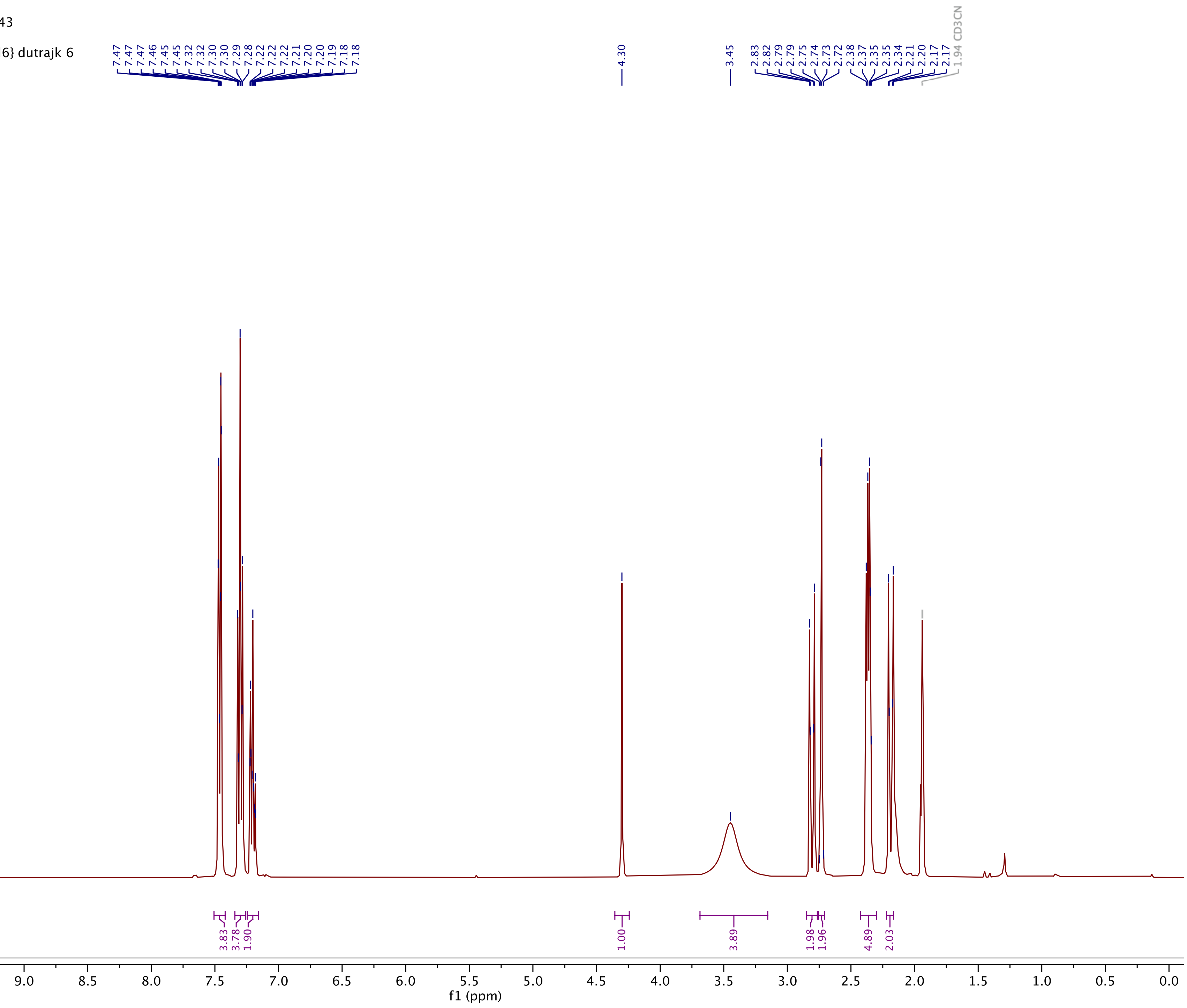

0110030-4016-006.6.fid
IMR System BNMR_319-1 400 10206243
fizer Confidential
arbon_80 CD3CN {C:\Bruker\TopSpin3.5pl6} dutrajk 6

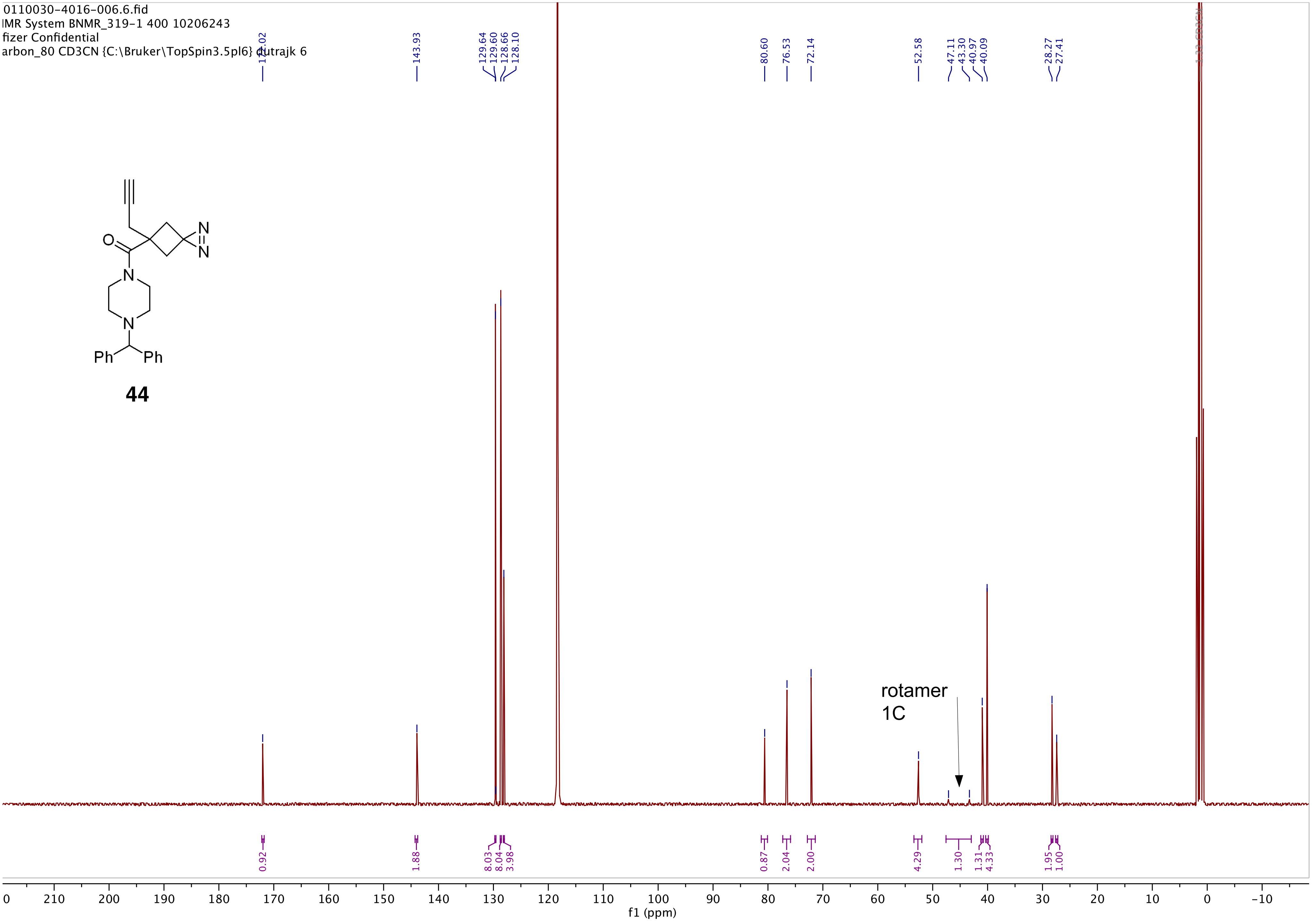

Supplement: SC-015-D4SC04238G-s001 [file SC-015-D4SC04238G-s001.pdf]
